# From bench to *in silico* and backwards: What have we done on genetics of recurrent pregnancy loss and implantation failure and where should we go next?

**DOI:** 10.1590/1678-4685-GMB-2023-0127

**Published:** 2024-08-26

**Authors:** Flavia Gobetti Gomes, Juliano André Boquett, Thayne Woycinck Kowalski, João Matheus Bremm, Marcus Silva Michels, Luiza Pretto, Marília Körbes Rockenbach, Fernanda Sales Luiz Vianna, Lavinia Schuler-Faccini, Maria Teresa Vieira Sanseverino, Lucas Rosa Fraga

**Affiliations:** 1 Universidade Federal do Rio Grande do Sul (UFRGS), Instituto de Biociências, Departamento de Genética, Programa de Pós-Graduação em Genética e Biologia Molecular, Porto Alegre, RS, Brazil.; 2 Universidade Federal do Rio Grande do Sul (UFRGS), Faculdade de Medicina, Programa de Pós-Graduação em Saúde da Criança e do Adolescente, Porto Alegre, RS, Brazil.; 3 University of California, Department of Neurology, San Francisco, CA, EUA.; 4 Hospital de Clínicas de Porto Alegre (HCPA), Centro de Pesquisa Experimental, Laboratório de Medicina Genômica, Porto Alegre, RS, Brazil.; 5 Hospital de Clínicas de Porto Alegre (HCPA), Núcleo de Bioinformática, Porto Alegre, RS, Brazil.; 6 Universidade Federal do Rio Grande do Sul (UFRGS), Programa de Pós-Graduação em Medicina, Ciências Médicas, Porto Alegre, RS, Brazil.; 7 Hospital de Clínicas de Porto Alegre (HCPA), Serviço de Genética Médica, Sistema Nacional de Informação sobre Agentes Teratogênicos (SIAT), Porto Alegre, RS, Brazil.; 8 Pontifícia Universidade Católica do Rio Grande do Sul (PUCRS), Escola de Medicina, Porto Alegre, Brazil.; 9 Universidade Federal do Rio Grande do Sul (UFRGS), Instituto de Ciências Básicas da Saúde, Departamento de Ciências Morfológicas, Porto Alegre, RS, Brazil.

**Keywords:** Embryo loss, embryology, genomics, miscarriage, bioinformatics

## Abstract

Human reproduction goes through many challenges to its success and in many cases it fails. Cases of pregnancy loss are common outcomes for pregnancies, and implantation failures (IF) are common in assisted reproduction attempts. Although several risk factors have already been linked to adverse outcomes in reproduction, many cases remain without a definitive cause. Genetics of female reproduction is a field that may bring some pieces of this puzzle; however, there are no well-defined genes that might be related to the risk for recurrent pregnancy loss (RPL) and IF. Here, we present a literature review of the studies of genetic association in RPL and IF carried out in the Brazilian population and complemented with a database search to explore genes previously related to RPL and IF, where a search for genes previously involved in these conditions was performed in OMIM, HuGE, and CTD databases. Finally, we present the next steps for reproductive genetics investigation, through genomic sequencing analyses and discuss future plans in the study of RPL genetics. The combined strategy of looking for literature and databases is useful to raise hypotheses and to identify underexplored genes related to RPL and IF.

## Introduction

Human reproduction is substantially ineffective, since about 70% of its conceptions do not survive until birth, and approximately 50% are lost before clinical recognition or embryonic cardiac activity (Hyde and Schust, 2015). Despite great advances in the diagnosis and treatment of infertility, its prevalence increases worldwide every decade (Borght and Wyns, 2018). According to the report of the World Health Organization (WHO), one in six couples experiences some type of difficulty when trying to conceive, which corresponds to 80 million people worldwide (Tournaye and Cohlen, 2012). In Brazil, for example, assisted reproduction cycles increased more than 30% from 2020 to 2021 (ANVISA, 2022).

It is estimated that 50% of all conceptions are lost at preclinical stages due to biochemical loss or implantation failure (ESHRE *et al*., 2018). A recent review including studies from Europe and North America found that the pooled risk of miscarriage 15.3% of all recognized pregnancies (Quenby et al., 2021). Considering 130 million births per year approximately, that would account to approximately 23 million miscarriages per year, or 44 per minute. The authors ponder that the number of miscarriages can be considerably higher than reported, since miscarriages and preclinical pregnancy losses are commonly managed at home (Quenby *et al.*, 2021).

Recurrent pregnancy loss can be caused by chromosomal errors, anatomical uterine defects, autoimmune disorders and endometrial dysfunction (Magnus et al., 2019). The reported incidence of early pregnancy loss may vary depending on the method used to detect pregnancy (Dimitriadis et al., 2020). Among 15 and 25% of all recognized pregnancies are lost (Quintero-Ronderos et al., 2017), mainly during the first trimester (Ammon Avalos et al., 2012). It is reported a sharp reduction after 12 weeks of gestation to an incidence of ~1%, suggesting that most pregnancy losses occur soon after implantation (Ammon Avalos *et al.*, 2012). Implantation failures are estimated to be responsible for at least 40% of failures in assisted reproduction cycles of health embryos (Quintero-Ronderos *et al.*, 2017).

Embryonic implantation occurs due to a complex interaction between a viable blastocyst and a receptive endometrium in a timeframe known as the implantation window (Ng et al., 2020). This critical process depends on the proliferation of the trophoblast, its invasion in the endometrium, and local angiogenesis, processes regulated by proteins that control the balance between growth factors and apoptosis (Mojarrad et al., 2013). It requires cellular and molecular events that result in uterine growth and differentiation, adherence to blastocysts, invasion, and formation of the placenta. In this context, advances in assisted reproduction techniques have enabled a broader analysis of embryonic implantations and their possible reasons for failure. Studies suggest that genetic factors involved in the implantation process are decisive in the establishment of pregnancy. Hence, impairments in genes that are related to this process may lead (or at least increase the susceptibility) to implantation failures (Krüssel et al., 2003; Zhai et al., 2021). Implantation failure (IF) is characterized when pregnancy is not reached after transferring at least four good-quality embryos in a minimum of three cycles of *in vitro* fertilization, in women under 40 years old (Coughlan et al., 2014).

Spontaneous pregnancy loss occurs when a clinically established pregnancy is lost before the fetus has developed sufficiently to guarantee its viability, or up to 24 weeks of gestation (van den Berg et al., 2012). The occurrence of two or more spontaneous pregnancy losses, a scenario known as Recurrent Pregnancy Loss (RPL), is a more unusual condition. It is estimated that approximately 5% of women will have two pregnancy losses and only 1% will have three or more (Kaser, 2018). RPL is associated with several causes, such as genetic mechanisms (mainly embryonic chromosomal aneuploidies), thrombophilia, immunological, hormonal, or metabolic alterations, infections, anatomical factors, among others (Kaser, 2018). Nevertheless, approximately half of RPL cases have an unknown etiology (Ford and Schust, 2009).

Due to the many etiologies, previous studies had limited success in identifying genetic susceptibility factors directly related to RPL and IF (Quintero-Ronderos et al., 2017). In addition, the molecular complexity of the reproductive process in mammals makes it difficult to recognize the genomic regions responsible for complex traits, which hampers the strategy for selecting candidate genes for RPL and IF studies (Quintero-Ronderos *et al.*, 2017). A more comprehensive molecular investigation of the factors related to these conditions is necessary to identify potential target genes strongly involved in both RPL and IF. In previous years, efforts have been made to try to identify potential molecular mechanisms and genetic susceptibility factors related to these main adverse pregnancy outcomes. The advance of technologies in genetic screening and the reduction in the cost of them combined with the development of many bioinformatic tools allowed the discovery of several risk factors for RPL and IF. In this sense, our research group has contributed to this field in the last ten years, investigating genetic susceptibility to RPL in the Brazilian population.

Considering the advances in the research of reproductive genetics and the need to identify new disease-causing genes to be investigated in adverse conditions of pregnancy, in this commemorative edition we present a literature review of RPL and IF genetic association studies carried out in the Brazilian population over the past 20 years. In addition, we performed a search on databases of genes and proteins potentially related to these conditions, comparing what has already been related to them, through bioinformatic tools. Finally, we discuss the next steps for the research on reproductive genetics to unravel the idiopathic cases of infertility.

## Methods

### Literature review

Electronic searches were performed in the PubMed/MEDLINE and EMBASE databases for studies on genetic association and RPL or IF carried out in the Brazilian population. The search terms used were: “recurrent pregnancy loss” OR “recurrent spontaneous abortion” OR “recurrent miscarriages” AND “genetics” AND “Brazil” for RPL; and “implantation failure” AND “genetic association” AND “Brazil” for IF. Additional literature was included from references of other studies. The search was conducted between April 14^th^ 2023 and February 14^th^ 2024. A screen on title and abstract was performed and the retrieved references were selected based on the relationship between RPL or IF and gene variants related to pregnancy. All genetic association case-control studies performed in Brazil were included in the review. Studies performed outside Brazil and with different methodology than genetic association case-control were not included. Only studies published in indexed journals were included. Conference papers and abstracts were not included in this literature review.

### Search in databases

The strategy used was based on the study conducted by Trouvé et al. (2017). Initially, a search for genes potentially involved in RPL and IF was carried out in databases relating genotype-phenotype: Online Mendelian Inheritance in Man - OMIM (Amberger and Hamosh, 2017), Comparative Toxicogenomics Database - CTD (Davis et al., 2019) and Human Genome Epidemiology Encyclopedia - HuGE (Yu et al., 2008). In all the databases, the used keywords were: “miscarriage”, “abortion”, “recurrent pregnancy loss” and “recurrent abortion” referring to RPL, and “implantation failure” and “embryo loss” regarding IF. With the search results, a list of genes was created for each database and specific condition. The genes in common were then compared in two forms: the genes in common between the two conditions and the genes in common for each condition in the different databases.

## Systems biology analysis and functional enrichment

To assess the possible interaction between the genes obtained in the database review, an analysis was performed in the STRING tool v.11.0 (Szklarczyk et al., 2019), with the following parameters defined: only the “experimental” and “co-expression” interactions were considered, with a combined confidence level score set of ≥ 0.4 as default (thus selecting medium trust interactions, as it is an exploratory trial). The networks obtained for both RPL and IF conditions were combined using the DyNet v.1.0.0 (Goenawan et al., 2016) application of the Cytoscape v.3.7.2 (Shannon et al., 2003) software.

To assess which biological processes the identified genes were involved, functional enrichment was carried out by accessing the Gene Ontology Consortium (GO) and the Kyoto Encyclopedia of Genes and Genomes (KEGG) repository, through the *clusterprofileR* (Yu et al., 2012) package, in the R software. For all the analyses, P-Value adjustment was performed with the Benjamini-Hochberg method, considering statistically significant results when adjusted P-Value < 0.05.

## Genetic association studies on recurrent pregnancy loss and implantation failure in the Brazilian population

**Figure 1-:**
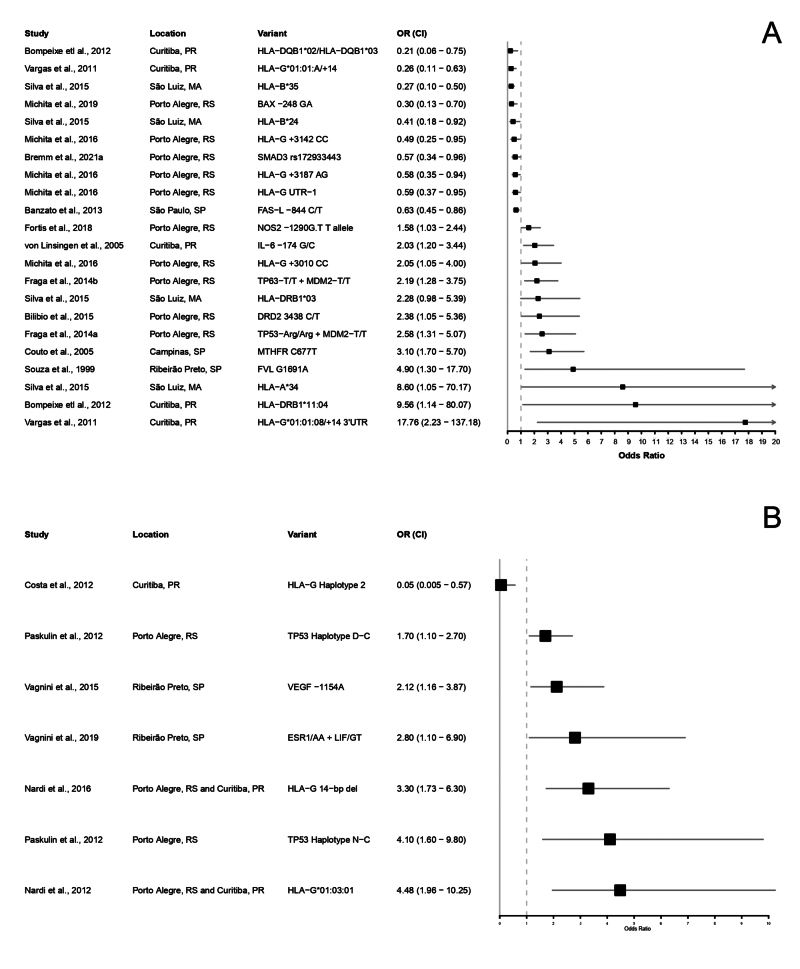
Forest plot presenting all genetic variants significantly associated to Recurrent Pregnancy Loss (**A**) and Implantation Failure (**B**). Only genetic variants with odds ratio and confidence interval available are shown.

Several genetic association studies attempted to find risk genetic variants for RPL in the Brazilian population (Table 1). Our PubMed/MEDLINE search retrieved 48 results, while the search conducted on EMBASE retrieved 43 results for RPL, whereas the search on IF retrieved 68 and 24 results for PubMed/MEDLINE and EMBASE databases respectively. Regarding RPL, 24 studies were included, in which nine have been conducted in Porto Alegre, Rio Grande do Sul (RS), nine in São Paulo (SP) state (four in São Paulo, three in Campinas, one in Ribeirão Preto and one in São José do Rio Preto), three in Curitiba, Paraná (PR), two in Salvador, Bahia (BA) and one in São Luís, Maranhão (MA). All of them were case-control studies, with the sample sizes varying from 20 to 156 RPL cases and from 31 to 384 subjects in control groups (Table 1). Fewer studies were found in our literature search on IF; two were conducted in Ribeirão Preto, SP, two used samples from Curitiba, PR and Porto Alegre, RS, one was conducted in Curitiba, PR and one in Porto Alegre, RS (Table 1). Figure 1 shows all genetic variants significantly associated with RPL and IF.

**Table 1:** Genetic association studies carried out on recurrent pregnancy loss and implantation failure in the Brazilian population.

Study Id / Region	Sample	Genes investigated	Physiological effect / Pathway	Main findings
***RPL***				
Souza et al., 1999 / Ribeirão Preto, SP	56 case × 384 ctrl	*FVL*, *FII*	Thrombophilia	*FVL* G1691A and *FII* G20210A variants were more prevalent in RPL patients in comparison with controls
Daher et al., 2003 / São Paulo, SP	48 case × 108 ctrl	*IFN-γ, TNF-α, IL-6, IL-10*	*IFN-γ* and *TNF-α* inhibit trophoblast growth and differentiation; interleukins may promote embryo development and placentation	Lack of association of the variants investigated in the population studied.
Barbosa et al., 2004 / Campinas, SP	86 case × 86 ctrl	*FXIII*	Blood clotting	The prevalence of the studied variants did not differ between RPL patients and controls
Couto et al., 2005 / Campinas, SP	88 case × 88 ctrl	*MTHFR, FVL, FII*	Thrombophilia	*MTHFR* C677T variant was statistically associated with RPL.
von Linsingen et al., 2005 / Curitiba, PR	57 case × 74 ctrl	*IL6, TGFB1*	Cytokine production	The frequency of the *IL6* (-174G/C) C/C genotype was increased in case women in comparison with controls.
Aléssio et al., 2008 / Campinas, SP	75 case × 179 ctrl	*ESR1, ESR2*	Estrogen receptor	There was no association between RPL and ESR polymorphisms.
Nunes et al., 2011 / São José do Rio Preto, SP	129 case × 182 ctrl	*ADA*	Affects the methylation process, cell growth and differentiation, apoptosis, DNA replication and immune function	*ADA*∗2 allele is associated with a low risk for recurrent spontaneous abortions, but this association is dependent on older age.
Traina et al., 2011 / São Paulo, SP	89 case × 191 ctrl	*PGR (PROGINS), IL-1R1, VEGF*	Implantation, trophoblastic invasion, maternal-fetal immune tolerance, angiogenesis and growth factor	No correlations were found in any of the investigated polymorphisms.
Vargas et al., 2011 / Curitiba, PR	60 case × 68 ctrl	*HLA-G*	Maternal-fetal interface tolerance	Haplotypic combinations of HLA-G alleles and the 14 bp segment (HLA-G*01:01:08/+14 and HLA-G*01:01:A/+14) may be associated with RPL.
Bompeixe et al., 2012 / Curitiba, PR	61 case × 75 ctrl	*HLA-DRB1, HLA-DQB1, IFN-γ, TNF-α, IL-10*	Maternal-fetal interface tolerance, trophoblast growth and differentiation; embryo development and placentation	HLA-DQB1*02:02, 03:01 alleles significantly decreased and HLA-DRB1*11:04 allele significantly increased among patients.
Banzato et al., 2013 / São Paulo, SP	129 case × 235 ctrl	*FAS, FAS-L*	FAS-FAS-L system is one important inducer of apoptosis	Significant differences in the *FAS-L* 844 C/T frequencies between women case and controls, suggesting that FAS-L gene polymorphism is associated with increased susceptibility to RPL.
Dutra et al., 2014 / Porto Alegre, RS	145 case × 135 ctrl	*MTHFR, FVL, FII, eNOS*	Thrombophilia	Lack of association of the variants investigated in the population studied.
Fraga et al., 2014b / Porto Alegre, RS	120 case × 143 ctrl	*TP53, MDM2, LIF*	p53 pathway (apoptosis)	The combination of *TP53* Arg/Arg (rs1042522) and *MDM2* TT (rs2279744) genotypes may be a risk factor for RPL.
Fraga et al., 2014a / Porto Alegre, RS	153 case × 143 ctrl	*TP63, TP73, MDM2*	Cycle cell arrest and apoptosis	Interaction between the *TP63* and *MDM2* variants shown to increase the risk of RPL.
Lino et al., 2015 / São Paulo, SP	112 case × 98 ctrl	*MTHFR, FVL, FXIII, PTM, PAI-1*	Thrombophilia	No correlations were found in any of the investigated polymorphisms.
Bilibio et al., 2015 / Porto Alegre, RS	20 case × 31 ctrl	*DRD2*	Prolactin secretion	An excess homozygosity of the *DRD2* variant (rs6277) suggests a genetic predisposition to RPL, which could result in a mild serum prolactin increase.
Silva *et al.*, 2015 / São Luís, MA	100 case × 100 ctrl	*HLA-A, HLA-B, HLA-DRB1*	Maternal-fetal interface tolerance	HLA-A*34 allele is a risk factor for RPL; HLA-A*24 and HLA-B*35 alleles are associated with protection.
Vilas Boas et al., 2015 / Salvador, BA	89 case × 150 ctrl	*MTHFR, MS, CBS*	Folate and vitamin B12-dependent homocysteine metabolisms	Lack of association of the variants investigated in the population studied
Gonçalves et al., 2016 / Salvador, BA	137 case × 100 ctrl	*MTHFR, FVL, FII*	Thrombophilia	Lack of association of the variants investigated in the population studied
Michita et al., 2016 / Porto Alegre, RS	156 case × 140 ctrl	*HLA-G*	Maternal-fetal interface tolerance	HLA-G 3’UTR plays important role in RPL
Fortis et al., 2018 / Porto Alegre, RS and Salvador, BA	149 case × 208 ctrl	*NOS2, PTGS2, VEGFA*	Angiogenesis and oxidative stress	The variant genotypes of the SNP rs2779249 in the *NOS2* promoter are a potential risk for RPL
Michita et al., 2019 / Porto Alegre, RS	140 case × 156 ctrl	*FAS, FAS-L, BAX, BCL-2*	Extrinsic and intrinsic apoptosis pathways	Association of BAX-248 G/A with RPL susceptibility
Bremm et al., 2021 / Porto Alegre, RS	149 case × 159 ctrl	*SMAD3*	Smad3-dependent signaling pathway (steroid hormone regulation and implantation)	Association of variant rs17293443 with the RPL
Bremm et al., 2021 / Porto Alegre, RS	149 case × 159 ctrl	*TDGF1, CFC1*	EGF-CFC family genes exert function in the formation of the body axes in the embryo	Lack of association of the variants investigated in the population studied
*IF*				
Costa et al., 2012 / Curitiba, PR	38 case × 14 ctrl	*HLA-G*	Maternal-fetal tolerance	No differences were observed in the allelic or genotypic frequencies between the success and failure groups. *HLA-G* Haplotype 2 is significantly presented in case women with failure implantation.
Paskulin et al., 2012 / Porto Alegre, RS	115 case × 134 ctrl	*LIF, TP53*	Blastocyst implantation; p53 pathway (apoptosis)	*TP53* PIN3 and PEX4 variants were associated with IVF when compared with control women. Haplotypes D-C and N-C were related to higher risk for failure of IVF when compared with the fertile group.
Nardi et al., 2012 / Porto Alegre, RS and Curitiba, PR	82 case × 166 ctrl (female) 162 case × 224 ctrl (couples)	*HLA-G*	Maternal-fetal tolerance	Lack of association in HLA-G alleles in case and control females. The frequency of the HLA-G*01:03:01 allele was increased in the IF couples.
Vagnini et al., 2015 / Ribeirão Preto, SP	120 case × 89 ctrl	*HAUSP, LIF, TP53, VEGF, gp130*	Blastocyst implantation; p53 pathway (apoptosis); angiogenesis and vascularization	Association between the VEGF -1154G/A polymorphism and recurrent IF.
Nardi et al., 2016 / Porto Alegre, RS and Curitiba, PR	49 case × 34 ctrl	*HLG-G*	Maternal-fetal tolerance	The 14-bp deletion allele is more frequent in IF women.
Vagnini et al., 2019 / Ribeirão Preto, SP	44 case × 63 ctrl(1) and 65 ctrl(2)	*ESR1, ESR2, LIF, MMP2, TP63, VEGFA*	*ESR1* and *ESR2* are estrogen receptors; *LIF* is important mediator of embryo implantation; *MMP2* play roles in remodeling extracellular matrix during ovarian follicular growth and ovulation; *TP63* is a regulator of the quality and maturation of oocytes; *VEGFA* acts in angiogenesis and vasculogenesis.	The *ESR1*/AA (rs12199722) and *LIF*/GT (rs929271) genotypes was more frequent in the case group.

## Thrombophilia

Different genes involved in signaling pathways and physiological processes that impact reproduction were studied for RPL and IF, being genes related to thrombophilia process the most studied (Souza et al.., 1999; Barbosa et al., 2004; Couto et al., 2005; Dutra et al., 2014; Lino et al., 2015; Vilas Boas et al., 2015; Gonçalves et al., 2016), mainly *FVL*, *FII* and *MTHFR* genes. Souza *et al.* (1999) found that the variants *FVL* G1691A and *FII* G20210A were more prevalent in RPL patients in comparison with controls, while in the study conducted by Couto *et al.* (2005) the *MTHFR* 677C>T (rs1801133) variant was associated with RPL. No association was found in the other studies involving thrombophilia related genes in the studied populations (Dutra *et al.*, 2014; Lino *et al.*, 2015; Gonçalves *et al.*, 2016). As thrombophilia is one of the main causes of spontaneous abortion during the early stages of pregnancy, studying variants in genes related to this process is justified (Kovalevsky et al., 2004). However, the role of polymorphisms that can lead to thromboembolic events with RPL is not well established yet.

## Immune system

As the fetus is semi-allogenic to mother, genes related to the immune system, especially those involved in the maternal-fetal interface tolerance, are amongst the most studied (Daher et al., 2003; von Linsingen et al., 2005, Vargas et al., 2011; Bompeixe et al., 2012; Silva *et al.*, 2015; Michita et al., 2016), as *IFNG*, *TNF*, *IL6*, *IL10*, *TGFB1,* and the HLA genes. Cytokines play an important role not only in tolerance induction, but also in reproductive processes, such as ovulation, implantation, placentation, cervical dilatation, and parturition (Yockey and Iwasaki, 2018). In this way, von Linsingen *et al.* (2005) studied the cytokine gene variant *IL6* -174G>C (rs1800795) and found an association of the -174CC genotype with RPL. This genotype leads to a lower production of the IL-6 cytokine, as for a successful pregnancy the IL-6 production should be higher.

HLA antigens expressed by fetal tissues can induce an alloimmune response in the mother, which is considered beneficial to the implantation process (Kumpel and Manoussaka, 2012). Of note, Vargas et al. (2011) found that the frequencies of haplotypic combinations of HLA-G alleles and the 14 bp segment (HLA-G*01:01:08/+14 and HLA-G*01:01:A/+14) were significantly higher in women with RPL, indicating a possible association with this condition. They also found the HLA-G*01:04:03 allele as risk to RPL whilst the allele HLA-G*01:01A had a protective effect for RPL on the population studied. In the study of Michita et al. (2016) the +3010 CC HLA-G 3’UTR variant was associated with risk to RPL. Both authors believe that the studied polymorphisms can affect the expression of HLA-G, which is related to the establishment of an immuno-tolerogenic environment by the mother. Furthermore, the HLA-DRB1*11:04 allele was significantly more frequent among RPL patients in the study of Bompeixe et al. (2012), and the HLA-A*34 allele was associated with risk for RPL (Silva *et al.*, 2015).

Nardi et al. (2012; 2016) conducted two studies analyzing HLA-G variants in IF patients. In their first study, no association was found when analyzing HLA-G alleles in case and control women, but when the alleles were analyzed between case and control couples, the frequency of HLA-G*01:03:01 allele was significantly higher in the IF group (Nardi *et al.*, 2012). According to the authors hypothesis, the association of a specific allele with IF in a population may result of both HLA-G and its ligand: if the connection of -G*01:03:01 allele and its KIR inhibitory receptor is insufficient, that would lead to an imbalance of signals between the activating and inhibitory receptors, which in turn would activate the cytotoxic action of the natural killer cells, finally leading to IF. Later, the 14-bp ins/del polymorphism located in exon 8 at the 3’UTR of the HLA-G gene was analyzed, in which the 14-bp deletion allele is more frequent in IF women. Moreover, the 14-bp del allele has been associated with higher values of circulating HLA-G molecules, the authors are cautious about the role of this polymorphism as a genetic marker, in which its functional significance influence on HLA-G release pathway needs further investigations (Nardi *et al.*, 2016). HLA-G variants were also studied by Costa et al. (2012). In their study, a specific haplotype (“haplotype 2”) is significantly more frequent in the group composed by women with IF.

## Angiogenesis and oxidative stress

Angiogenesis and oxidative stress are important physiologic processes in which several related genes were studied (Traina et al., 2011; Fortis et al., 2018), mainly the *VEGFA* gene, since it is a key regulator of angiogenesis and an essential growth factor for normal placental development (Zygmunt et al., 2003). The study conducted by Vagnini et al. (2015) showed an association between the VEGF -1154G/A variant and recurrent IF in Brazilian women. Of all gene variants related to angiogenesis and oxidative stress, only the SNP -1290G>T (rs2779249) in the *NOS2* promoter were associated as a potential risk for RPL (Fortis *et al.*, 2018). The placental oxidative stress was implicated as a potential factor for spontaneous pregnancy loss (Burton and Jauniaux, 2011). Thus, the hypothesis supported by Fortis *et al.* (2018) is that an increased *NOS2* expression could lead to excessive nitric oxide production and, in turn, increase the production of peroxynitrite, a potent pro-oxidant, resulting in deleterious effects.

Variants in genes involved in cell death as *FAS*, *FASLG* were investigated (Banzato et al., 2013; Michita et al., 2019), in which the *FASLG* (-844C>T; rs763110) variant was associated with susceptibility to RPL and *FASLG* mRNA expression was higher in RPL women in comparison to the control group (Banzato *et al.*, 2013). The authors hypothesized that RPL patients may have an altered trophoblast invasion due to increased *FASLG* expression. Furthermore, the FAS-FAS-L system is important in the maternal-fetal immune tolerance, in which impaired expression of these molecules could affect trophoblast apoptosis and interfere in gestation. On the other hand, Michita *et al.* (2019) did not find association between the aforementioned *FASLG* variant and RPL. In their study, however, the heterozygous genotype for the *BAX* -248G>A (rs4645878) variant was associated with RPL, which led the authors to speculate that *BAX -*248GA heterozygous subjects could have a better control of *BAX* expression, resulting in a well-balanced proapoptotic and antiapoptotic ratio in comparison to homozygotes (Michita *et al.*, 2019).

## Cell cycle and apoptosis

The p53 protein signaling pathway plays a crucial role in apoptosis, and variants in genes involved in this process (*TP53*, *TP63*, *TP73*, *MDM2* and *LIF*) were investigated in RPL (Fraga et al., 2014a, b). The combination of *TP53* Arg/Arg (rs1042522) and *MDM2* TT (rs2279744) genotypes (Fraga *et al.*, 2014b), as well as the *TP63* TT (rs17506395) and *MDM2* TT (rs2279744) genotypes (Fraga *et al.*, 2014a) showed to increase the risk of RPL. The role of p53 signaling pathway involves embryo selection through post implantation apoptosis induction. In addition, p63 can induce repair and apoptosis in order to protect the quality of the female germ line during meiotic arrest being crucial for proper gestation (Feng et al., 2011). Regarding IF, three *TP53* variants were investigated by Paskulin et al. (2012). In their study, PIN3 (Polymorphism in Intron 3, rs17878362, 16 bp duplication) and PEX4 (same as p.P72R - C/G, rs1042522) variants were associated with IVF when compared with control women.

## Embryo development

Variants in genes involved in embryonic development such as *TDGF1*, *CFC1* and *SMAD3* were also studied (Bremm et al., 2021, 2022). An intronic variant in *SMAD3* variant (C.207-19370T>C; rs17293443) was associated with RPL. *In silico* predictions showed that this variant can affect the expression levels of the gene, which could impact in processes such as steroid hormone regulation and implantation (Bremm *et al.*, 2022). It is worth mentioning studies that investigated genes involved in other processes as in the estrogen receptors (*ESR1*, *ESR2*; Aléssio et al., 2008), prolactin secretion (*DRD2*; Bilibio et al., 2015) and processes related to methylation, cell growth and differentiation (*ADA*; Nunes et al., 2011). Finally, Vagnini et al. (2019) investigated the relationship of genetic variants and IF in six genes also studied in RPL: *ESR1, ESR2, LIF, MMP2, TP63, VEGFA*. Of all variants analyzed, the *ESR1* AA (rs12199722) and *LIF* GT (rs929271) genotypes were more frequent in the IF group, leading to an increase in the chance of women carrying these genotypes presenting IF. The authors suggest that variants in *ESR1* gene could modify estrogen action, modifying the way the endocrine system acts in the implantation process. Additional information can be found in Table 1.

## Connecting hubs to find new candidates: Bioinformatics and systems biology

Through the previous years, advances in genetics, genomics, and computational biology have increased the number of tools available to shed light on many mechanisms involved in medical conditions. Until recently, most of the studies used to select the investigated genes by using mainly literature and known physiological mechanisms involved with pregnancy. Literature searches are extremely useful and essential to understand genetic factors related to infertility. However, bioinformatic tools and database research can be very informative in the search for new candidate genes and proteins potentially involved in different conditions.

In order to complement our literature review, a search in databases was performed and systems biology approaches were applied. A total of 671 registries related to RPL was found in all three databases: 132 from OMIM, 358 from HuGE, and 181 from the CTD database comprising 604 unique genes. Despite these many genes, only 10 were common to the three databases for this specific condition: *AGTR1*, *ANXA5*, *FII*, *FV*, *GPX4*, *JAK2*, *NOS3*, *PRLR*, *PGR*, and *SYCP3* (Table S1).

Of all these 10 genes, *FII*, *FV*, *NOS3,* and *PGR* have been studied in RPL case-control association studies with the Brazilian population. The *AGTR1* gene expresses angiotensin receptors, which is an important effector controlling blood pressure and volume in the cardiovascular system, and was implicated with hypertension in pregnancy (Kobashi et al., 2004). *ANXA5* gene codes for an anticoagulant protein annexin that has been studied in pregnancy, being associated with RPL in German women (Bogdanova et al., 2007). *GPX4* plays a role in pathophysiologic processes, including inflammation and atherogenesis (OMIM, 138322) and *JAK2*, a tyrosine kinase involved in cytokine signaling, has a variant already associated in pregnancy loss (Mercier et al., 2007). *PRLR* and the *SYCP3* genes, which encode prolactin receptor and synaptonemal protein, respectively, are other potential candidates for RPL studies (Bolor et al., 2009; Kim et al., 2018).

Compared to RPL, fewer genes were related to Implantation Failure. In total, 34 entries were found: 9 from OMIM, 16 from HuGE, and 9 from CTD, without any genes in common among the three databases. *FV* was registered both in OMIM and HuGE. Hence, 33 unique genes were annotated (Table S2). When comparing the results obtained for RPL *versus* IF, 26 genes in common were identified (previously associated with both conditions).

**Figure 2 -:**
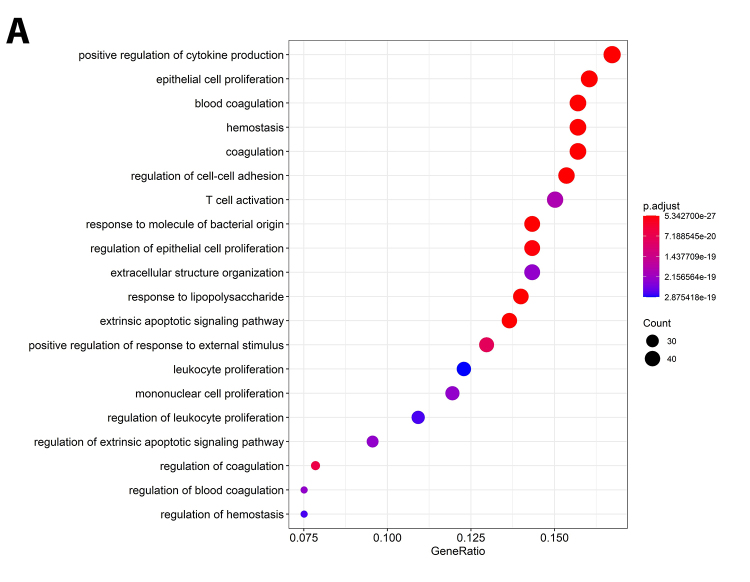
Protein-protein interaction network for the genes obtained from database review (**A**). Genes registered as associated with recurrent pregnancy loss are represented in red, whilst genes registered for both recurrent pregnancy loss and implantation failure are represented in blue (**B**).

**Figure 3-:**
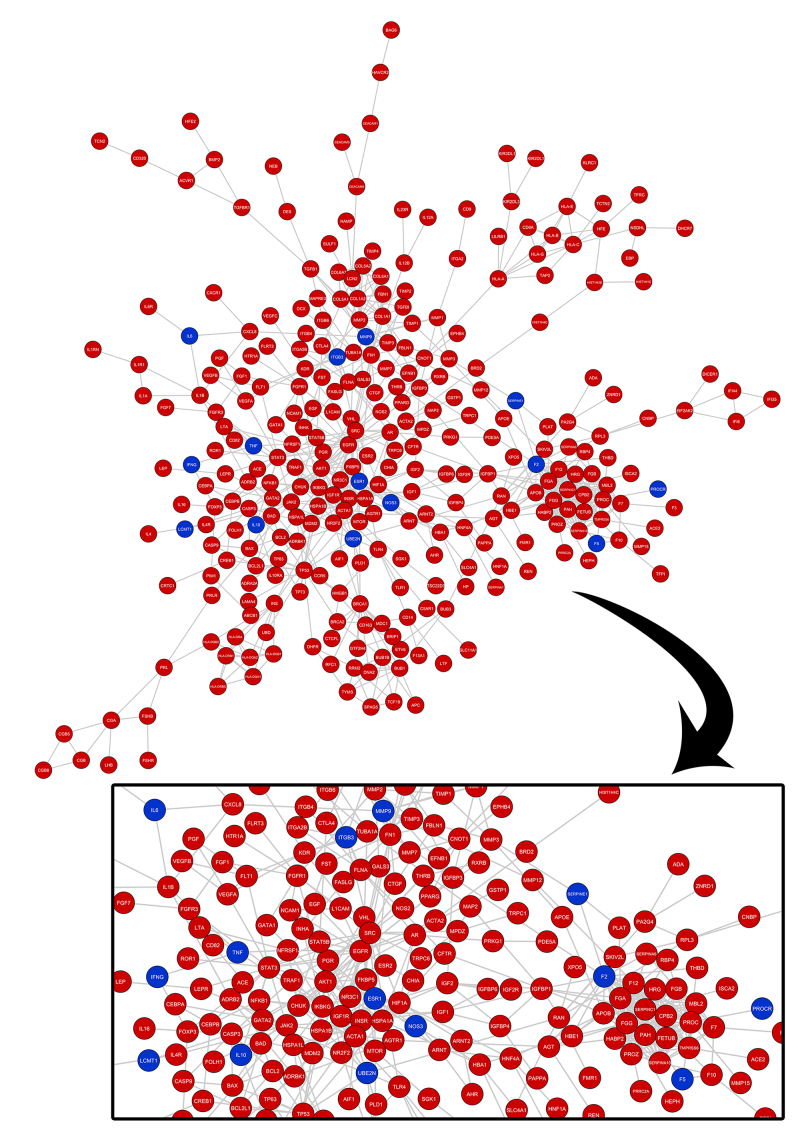
Main enriched Gene Ontologies (GO) by adjusted P-Value.

Further, the lists of candidates obtained from IF or RPL were uploaded on STRING, and two protein-protein interaction (PPI) networks were obtained and combined into one (Figure 2A). The evaluation of the combined network showed that all the proteins of the IF network were present in the RPL network (Figure 2B). Analyses of ontologies and associated pathways showed that the main enriched ontologies include biological processes especially related to coagulation, the immune system, cell-cell adhesion, and regulation of proliferation/apoptosis (Figure 3). Similarly, KEGG pathway enrichment pointed to cancer and autoimmune conditions. The proteins in common between both conditions are depicted in Table S3 and the complete list of enriched processes is available in Table S4 and Table S5, respectively, for GO and KEGG.

Bioinformatics and systems biology tools are very useful in the search for genes and proteins with specific physiological functions in the search for new candidates to be investigated in a given outcome. Complementarily, the search for variants with biological impact includes effect prediction tools and an extensive literature review. The combination of different tools and research methods are of great value in formulating a good hypothesis to be tested, then, in a case-control study.

## Beyond gene candidate analyses: Genomic sequencing analyses

Nowadays, many techniques are available to evaluate genome analysis. Among them, whole exome sequencing (WES), through next-generation sequencing (NGS), allows the analysis of several coding genes, enabling the identification of variants that may be related to a given condition (Petersen et al., 2017). Thus, instead of a gene-specific approach, which disregards the other genes indirectly involved in a disease, a large number of genes is considered, increasing the chances of identifying variants related to the condition of interest. In addition, the generated data allow a better understanding of the mechanisms involved, since they point to candidate genes. In this way, NGS has revolutionized the diagnosis of genetic diseases, however its use in reproductive conditions remains little explored (Rajcan-Separovic, 2020). Considering investigations on RPL, some approaches involve WES to identify gene variants in coding regions, as well as chromosomal microarray analysis to detect chromosomal imbalances and copy number variations (Rajcan-Separovic, 2020).

Despite chromosomal alterations in the conceptus being recognized as the most common causes of pregnancy loss and greater knowledge of genes associated with RPL, several events remain without a defined cause, as already mentioned. Therefore, studies considering a large number of genes, such as the WES, represent a promising way to identify the genetic causes involved in cases of idiopathic RPL. Some genes have already been associated with RPL through NGS, mainly those involved in important biological processes for the implantation and maintenance of pregnancy, as well as for embryonic development, such as cell division, differentiation, migration and adhesion, ciliary movements, immune functions and processes related to the coagulation cascade (Quintero-Ronderos et al., 2017; Rajcan-Separovic, 2020). Furthermore, knowing the genes associated with this condition, it may be possible to develop genetic panels for diagnostic clinical application. Recently, the first results of a genetic panel for the diagnosis of female and male infertility in Latin America were published (Lorenzi et al., 2020), contributing to advances in the area and to more precise approaches in the management of infertility. Still, investments in NGS of cases of unexplained infertility, particularly RPL and IF, should be done in order to improve the understanding of many idiopathic cases. In this sense, our research group is now taking the studies on RPL to the genomic era, in which, exomic sequencing will be performed in unexplained cases of RPL.

## Conclusion and Perspectives

In this review we aimed to provide a broader view on what has been done on genetics of RPL and IF in Brazilian population. In this sense, we gathered data from all studies that carried out genetic association analyses on this subject over the past 20 years. For the most part, studies have focused on genes related to the following physiological pathways: thrombophilia (Couto et al., 2005; Dutra et al., 2014; Lino et al., 2015; Gonçalves et al., 2016), immune system (Daher et al., 2003; von Linsingen et al., 2005; Vargas et al., 2011; Bompeixe et al., 2012; Nardi et al., 2012; Silva *et al.*, 2015; Michita et al., 2016; Nardi *et al.*, 2016), angiogenesis and oxidative stress (Traina et al., 2011; Fortis et al., 2018; Vagnini et al., 2019), cell death (Banzato et al., 2013; Michita *et al.*, 2019), apoptosis (Fraga et al., 2014a, b; Vagnini *et al.*, 2019) and embryonic development (Vagnini *et al.*, 2019; Bremm et al., 2021, 2022). Interestingly, no variant was associated with RPL in more than one study with the Brazilian population (Table 1). This result could be explained by several reasons. The majority of the studies are concentrated in the South and Southeast regions of Brazil, with few studies conducted in the Northeast and none in Midwest and North regions. The Brazilian population is known by its admixture, which can lead to differences in genetic variant frequencies and biases related to the self-perceptions based on skin color (Kehdy et al., 2015). Additionally, differences in the study design and phenotypes evaluated, as well as the small size effect that these common variants can have on the RPL become challenging the reproducibility of the findings among different studies.

It is noteworthy that almost half of the retrieved studies have been conducted by our research group, showing expertise and contribution to the field. Throughout the years, we have used different approaches on the investigation of genetic factors related to RPL, such as computational analysis with systems biology, and analyses of gene expression studies from public databases. Future studies on reproductive genetics and investigations on genetic susceptibility of RPL should also combine different methods to evaluate potential risk factors to this condition. In this sense, the use of bioinformatic tools may be extremely useful to give clues on how reproductive conditions may be affected by genetic alterations. The combination of literature review, genetic investigation and search on genetic and genomic databases are important to find genes and proteins potentially related to RPL and IF.

Complementarily to the literature review, genomic databases OMIM, HuGE, and CTD were evaluated, and genes previously associated with RPL or IF were annotated. The candidate genes obtained in both strategies were compared, for both clinical conditions. Few genes were common between the methods used. This can be explained due the fact that they are different entities in terms of pregnancy, having distinct molecular control. Still, comparing them is important to improve the knowledge of the molecular mechanisms involving pregnancy on its different stages and how they might be disturbed. Of the 10 genes common to the three databases for RPL, variants in *FII*, *FV*, *NOS3* and *PGR* genes were studied in the Brazilian population for this outcome. Interestingly, no associations were found with the variants studied in these four genes. The other six genes (*AGTR1*, *ANXA5*, *GPX4*, *JAK2*, *PRLR* and *SYCP3*) were not studied in Brazilian RPL or IF patients to date, and seem to be good candidates for future case-control RPL and/or IF studies. When performing the database review, the lack of information for IF, in comparison to RPL, is evident.

Finally, here we reviewed all genetic association studies for RPL and IF carried out in Brazil in the last twenty years. In addition, we present alternatives in the search for new candidate genes, through bioinformatics and systems biology tools. Yet, genome-wide sequencing studies can be an interesting option in obtaining more comprehensive results than studies with a candidate gene/variant. The complex etiology of RPL and IF demonstrates the need for assessing *omics* data, which evaluate several genes that might interact and result in both. This knowledge might represent an important tool for clinical practice, as it may identify the etiology of the RPL or IF and the couple’s prognosis, thus reducing the suffering and the costs involved in the journey until conception. From this perspective, the comprehension of the molecular mechanisms, allied with precision medicine, can help to improve the success rate of IVF cycles and the management of adverse events of pregnancy.

## Supplementary Material

The following online material is available for this article:

Table S1 - Database research for recurrent pregnancy loss

Table S2 - Database research for implantation failure

Table S3 - Comparison of the common proteins obtained from database research for recurrent pregnancy loss and implantation failure

Table S4 - Enriched gene ontologies for recurrent pregnancy loss + implantation failure network

Table S5 - Enriched KEGG pathways for recurrent pregnancy loss + implantation failure network
