# Erratum: From bench to *in silico* and backwards: What have we done on genetics of recurrent pregnancy loss and implantation failure and where should we go next?

**DOI:** 10.1590/1678-4685-GMB-2023-0127er

**Published:** 2024-11-22

In the article “From bench to in silico and backwards: What have we done on genetics of recurrent pregnancy loss and implantation failure and where should we go next?”, with DOI code number https://doi.org/10.1590/1678-4685-GMB-2023-0127, published in the Genetics and Molecular Biology, 46(3 Suppl 1): e20230127 (2023):

**Where it was written:**

**Figure 2 -:**
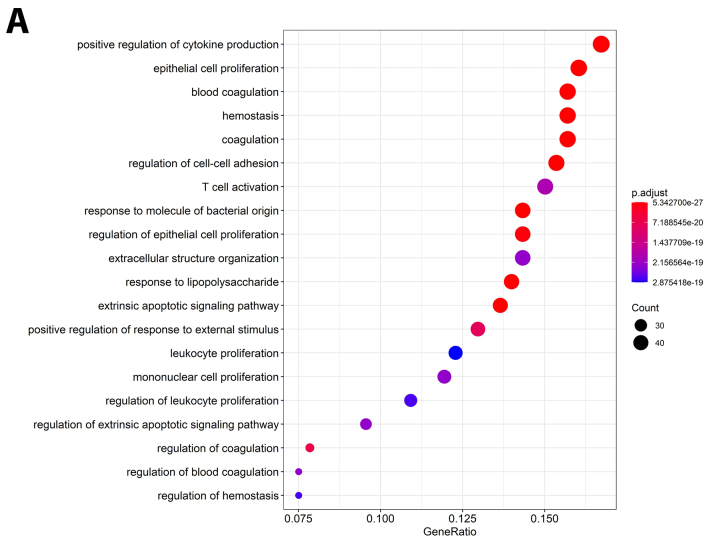
Protein-protein interaction network for the genes obtained from database review (**A**). Genes registered as associated with recurrent pregnancy loss are represented in red, whilst genes registered for both recurrent pregnancy loss and implantation failure are represented in blue (**B**).

**Figure 3 -:**
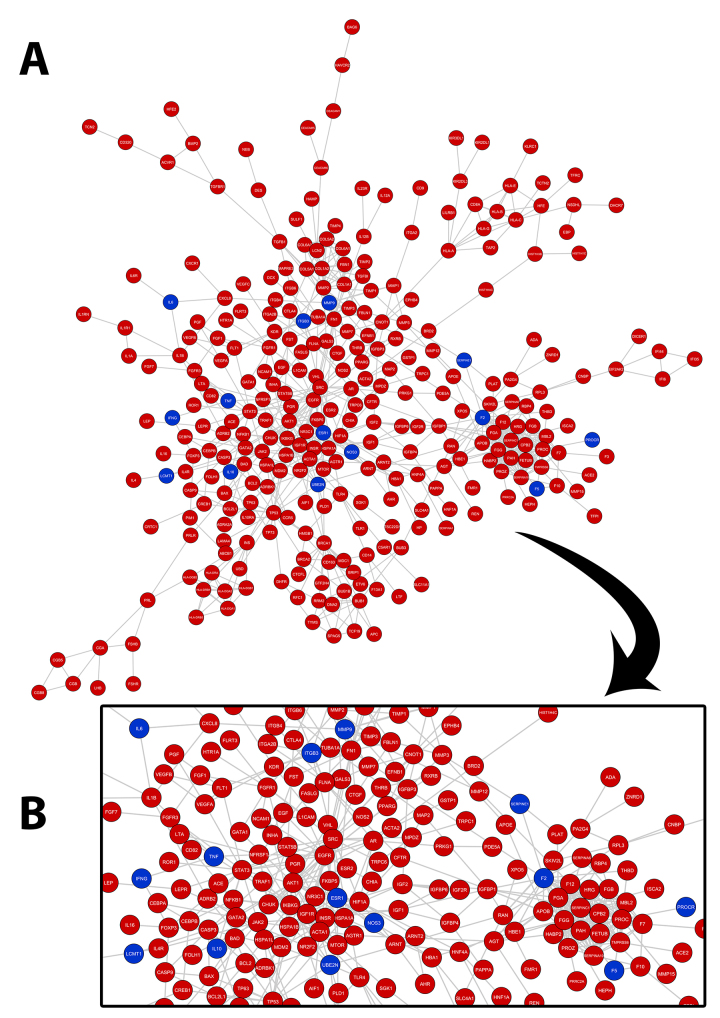
Main enriched Gene Ontologies (GO) by adjusted P-Value.

**Should read:**

**Figure 2 -:**
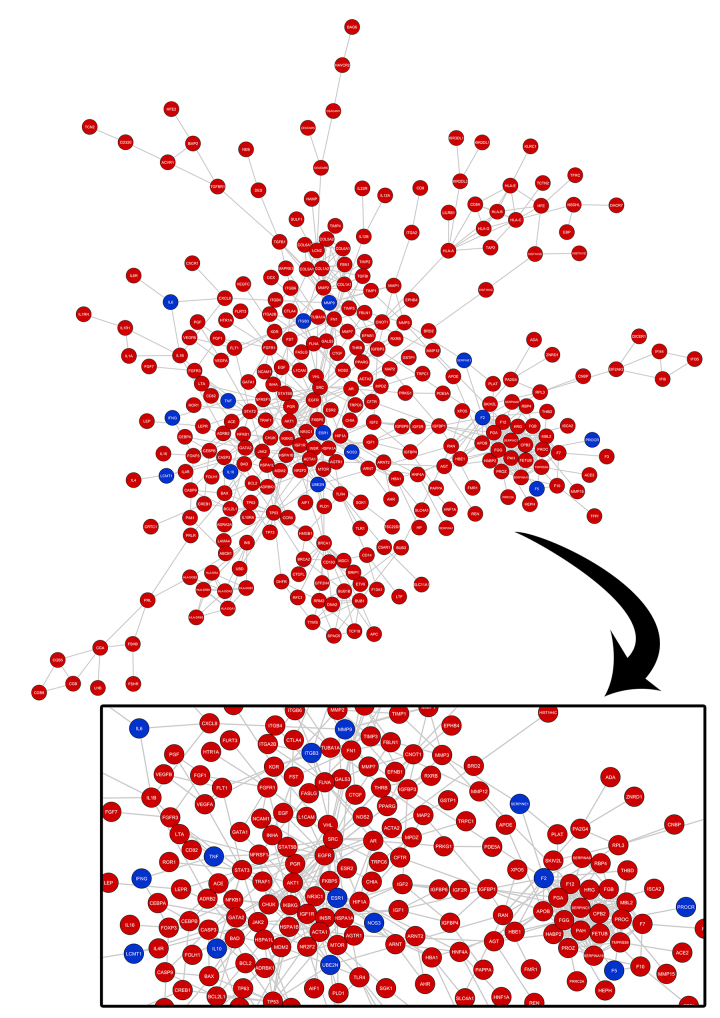
Protein-protein interaction network for the genes obtained from database review (**A**). Genes registered as associated with recurrent pregnancy loss are represented in red, whilst genes registered for both recurrent pregnancy loss and implantation failure are represented in blue (**B**).

**Figure 3 -:**
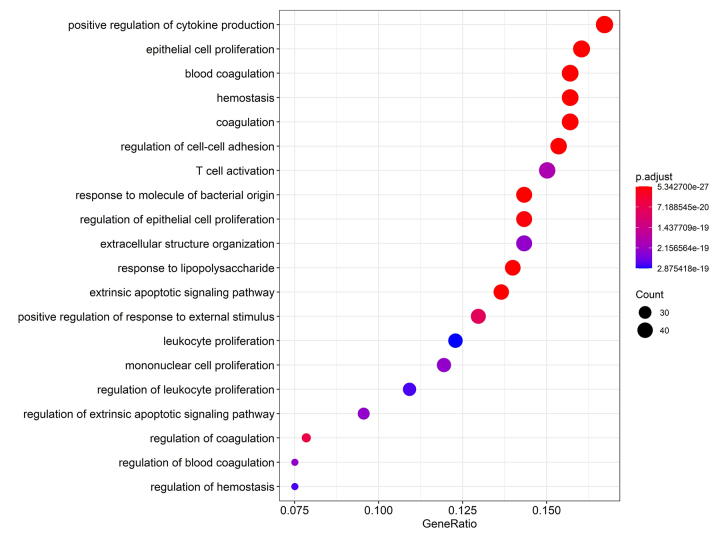
Main enriched Gene Ontologies (GO) by adjusted P-Value.

