# Supplementary material for: From bench to in silico and backwards: What have we done on genetics of recurrent pregnancy loss and implantation failure and where should we go next?
Source: Genet Mol Biol. 2024 Aug 26;46(3 Suppl 1):e20230127. doi: 10.1590/1678-4685-GMB-2023-0127 (PMC11346592; doi:10.1590/1678-4685-GMB-2023-0127)
Supplement: Table S1 - [file 1415-4757-GMB-46-03-s1-e20230127-s1.pdf]

Genetics and Molecular Biology

DOI: https://doi.org/10.1590/1678-4685-GMB-2023-0127

Supplementary Material to "From bench to *in silico* and backwards: what have we done on genetics of recurrent pregnancy loss and implantation failure and where should we go next?"

Table S1 - Database research for recurrent pregnancy loss.

OMIM	HuGE	CTD
A4GALT	ABCB1	ACE2
ABCG2	ACE	ACKR4
ACE	ACHE	ACTA2
ADRA2A	ACP1	ADIPOQ
AGT	ACTA1	AGT
AGTR1	ACVR1	AGTR1
ALG6	ADA	AHR
ANXA5	ADAD1	AKT1
ARNT2	ADH1B	ALPG
ATPAF2	ADIPOQ	ANGPT1
B3GALNT1	ADRA2B	ANXA5
B3GALT3	ADRB2	APOE
BCOR	ADRB3	ARG1
BUB1	AGTR1	ARNT
BUB1B	AIF1	BAD
BUB3	ALDH2	BAX
C5AR1	ALPP	BCL2
CBS	ANGPT2	BCL2L1
CCDC88C	ANXA5	CASP3
CD46	APC	CASP9
CD9	APOB	CAT
CEP120	APOE	CD163
CGA	AR	CD164
CGB3	ARNT	CD226
CHUK	BAT2	CD68
CNGB3	BAT3	CD69
COX7B	BMP2	CD7
CPT1B	BMP6	CD82
CREB3L1	BRCA1	CD84
CSPP1	BRCA2	CD8A
DACT1	BRD2	CEACAM1
DCX	BRIP1	CEACAM5
DGKZ	BTNL2	CEACAM6
DHCR7	C4BPA	CEBPA

OMIM	HuGE	CTD
DIH1	C6orf10	CEBPB
DLEU2L	C6orf221	CFTR
DNA2	CAT	CHIA
EBP	CBS	COL13A1
EFNB1	CCR5	COL1A1
EPHB4	CD14	COL1A2
ETV6	CD320	COL4A6
EXT1	CD46	COL5A1
F10	CD55	COL5A2
F12	CDKAL1	COL6A1
F13A1	CETP	COL6A3
F13A1	CFB	COL9A2
F2	CGB	CREB1
F3	CGB5	CRISP3
F5	CGB8	CSF3
FBN1	CLIC1	CSRNP3
FGA	CNOT1	CTGF
FGB	COL1A1	CXCL10
FGFR1	COMT	CYP1A1
FGG	COX2	CYP24A1
FLNA	COX3	CYP3A4
FMN2	CPB2	CYP3A7
FMR1	CRP	DES
FOXD3	Crtc1	DHFR
FRA16B	CTLA4	DIAPH2-AS1
GATA1	CXCR1	ECM1
GATA2	CYBRD1	ECM2
GDF-15	CYP17A1	EDN1
GLA	CYP19A1	EMP1
GPHN	CYP1A1	F2
GPX4	CYP1A2	F5
GPX5	CYP1B1	FABP4
GUSB	CYP21A2	FASN
HABP2	CYP2D6	FETUB
HCCS	CYP7A1	FGF1
HLA -G	CYTB	FGF7
HLA-DQA1	DDR1	FGFR3
HPS6	DICER1	FKBP5
HTR1A	DNMT3L	FLRT3
IKBKG	DRD2	FN1
INSR	DROSHA	FSHB
ISCA2	ECA1	FST
ITGB3BP	EDN1	GLS
JAK2	EGF	GPX4

OMIM	HuGE	CTD
KAOGS	EGFL8	GRK2
KHDC3L	EGFR	HABP2
KIAA0586	EIF2AK2	HMOX1
KIAA1109	ESR1	HSD17B1
KIAA1799	ESR2	IFI35
L1CAM	F12	IFI44
LMNA	F13A1	IFI6
MME	F2	IFNA10
MPDZ	F5	IGF1
MTHFR	F7	IGF2
NEB	F8	IGFBP1
NEU1	FASLG	IGFBP3
NF1	FAZ	IGFBP6
NLRP5	FBLN1	IL11
NLRP7	FGA	IL12B
NOS3	FGB	IL16
NPHP3	FLT1	IL1B
NR2F2	FMR1	IL20RA
NSDHL	FOLH1	IL24
PAH	FOXP3	IL4
PCOS1	FSHR	IL4RA
PEE1	FTO	IL5
PGM1	FVT1	IL5RA
PGR	GCK	IL6
PLA2R1	GCLC	IL9
PLD1	GOLPH3	INHA
POMT1	GP6	INS1
PORCN	GPX1	ITGB4
PPP2R2C	GPX4	ITGB6
PRL	GSTA1	JAK2
PRLR	GSTK1	KLK10
PROM1	GSTM1	LAMA4
PROZ	GSTO1	LCMT1
REN	GSTO2	LGALS14
ROR1	GSTP1	LGALS3
SCO2	GSTT1	LHB
SGK1	GTF2H4	LIF
SHOX	H19	MAP2
SHOXY	HAMP	MAPRE3
SLC35D1	HAVCR2	MGAT2
SLC4A1	HBA1	MGP
SPAST	HBE1	MMP11
STAT5B	HEPH	MMP12
STEAP3	HFE	MMP15

OMIM	HuGE	CTD
SYCP3	HFE2	MMP19
TCTN2	HIF1A	MMP26
TDO2	HIST1H1C	MMP7
THBD	HIST1H1T	MPO
TMCO1	HIST1H3B	MUC4
TRAPPC12	HIST1H4C	NCAM1
TSEN54	HLA-A	NOS1
TSHR	HLA-B	NOS2
XIC	HLA-C	NOS3
ZNF9	HLA-DPB2	NPPA
	HLA-DQA1	NPPB
	HLA-DQA2	NR3C1
	HLA-DQB1	PADI6
	HLA-DQB2	PAEP
	HLA-DRA	PARG
	HLA-DRB1	PCDHA3
	HLA-DRB3	PDE5
	HLA-DRB4	PDE5A
	HLA-DRB5	PDE8B
	HLA-E	PGF
	HLA-G	PGR
	HMGB1	PPARG
	HMOX1	PRKG1
	HNF1A	PRLR
	HNF4A	PTGIS
	HP	PTHLH
	HRG	RBP4
	HSD17B1	REN
	HSD17B8	SDF2L1
	HSD3B1	SERPINB3
	HSPA1A	SERPINB4
	HSPA1B	SLC22A2
	HSPA1L	SLC2A4
	IDO1	SLC31A1
	IER3	SPAG5
	IFGF2	SST
	IFNG	SYCP3
	IGF1	TFRC
	IGF1R	TGFB1
	IGF2	TGFBI
	IGF2R	TGFBR1
	IGFBP3	TIMP2
	IGFBP4	TIMP3
	IL10	TLE6

OMIM	HuGE	CTD
	IL10RA	TNF
	IL12A	TNFSF10
	IL17A	TNFSF13
	IL17F	TNR
	IL18	TP53
	IL19	TRAF1
	IL1A	TRPC1
	IL1B	TRPC6
	IL1R1	TSC22D3
	IL1RN	TUBA1A
	IL21	TXN1
	IL23R	UBE2N
	IL4	VEGFA
	IL4R	VEGFB
	IL6	VEGFC
	IL6R	
	IL8	
	INHA	
	INS	
	IREB2	
	IRF4	
	ITBG3	
	ITGA2	
	ITGA2B	
	ITGB3	
	JAK2	
	JAZF1	
	KDR	
	KIAA0319L	
	KIR2DL1	
	KIR2DL2	
	KIR2DL3	
	KIR2DL4	
	KIR2DL5A	
	KIR2DP1	
	KIR2DS1	
	KIR2DS2	
	KIR2DS3	
	KIR2DS4	
	KIR2DS5	
	KIR3DL1	
	KIR3DL2	
	KIR3DL3	
	KIR3DP1	

OMIM	HuGE	CTD
	KIR3DS1	
	KIR3DS5	
	KIRD3DL1	
	KIRD3DS1	
	KLRC1	
	KLRC4	
	KLRC4-KLRK1	
	KLRK1	
	LCN2	
	LCT	
	LEP	
	LEPR	
	LHCGR	
	LIF	
	LTA	
	LTF	
	LY6G6F	
	MBL2	
	MDC1	
	MDM2	
	MICA	
	MIR125A	
	MIR146A	
	MIR149	
	MIR196A2	
	MIR423	
	MIR499	
	MMP1	
	MMP2	
	MMP3	
	MMP9	
	MSH4	
	MSH5	
	MTHFD1	
	MTHFR	
	MTOR	
	MTR	
	MTRR	
	NCR3	
	ND1	
	ND2	
	ND4	
	ND6	
	NFE2L2	

OMIM	HuGE	CTD
	NFKB1	
	NFKBIL1	
	NLRP2	
	NLRP7	
	NOS3	
	NOTCH4	
	NR3C1	
	NR4A3	
	OGG1	
	OSR1	
	PA2G4	
	PAPPA	
	PBX2	
	PC	
	PDE8B	
	PGR	
	PIM1	
	PLAT	
	PLAUR	
	PON1	
	POU5F1	
	PPARG	
	PRL	
	PRLR	
	PROC	
	PROCR	
	PROK1	
	PROKR1	
	PROKR2	
	PROS1	
	PROZ	
	PXK	
	RAN	
	RETN	
	RFC1	
	RHOF	
	RPL3	
	RRM2	
	RXRB	
	SELP	
	SERPINA1	
	SERPINA10	
	SERPINA6	
	SERPINB2	

OMIM	HuGE	CTD
	SERPINC1	
	SERPINE1	
	SHBG	
	SIPA1	
	SKIV2L	
	SLC11A1	
	SLC11A2	
	SLC17A3	
	SLC19A1	
	SLC39A14	
	SLC39A4	
	SLC39A7	
	SLC40A1	
	SLC6A4	
	SOD2	
	SPINK1	
	SRC	
	SRI	
	STAT3	
	STAT4	
	STEAP3	
	SULF1	
	SYCP3	
	TAP2	
	TCF19	
	TCN1	
	TCN2	
	TFF3	
	TFPI	
	TFR2	
	TFRC	
	TGFB1	
	THARB	
	THBD	
	THRB	
	TIMP1	
	TIMP2	
	TIMP3	
	TIMP4	
	TLR1	
	TLR4	
	TMPRSS6	
	TNF	
	TNFRSF1A	

OMIM	HuGE	CTD
	TNXB	
	TP53	
	TP63	
	TP73	
	TPH1	
	TSHR	
	TYMS	
	UBD	
	UCP2	
	USP26	
	UTS2R	
	VEGF	
	VEGFA	
	VHL	
	XPO5	
	ZNRD1	
