# Supplementary material for: From bench to in silico and backwards: What have we done on genetics of recurrent pregnancy loss and implantation failure and where should we go next?
Source: Genet Mol Biol. 2024 Aug 26;46(3 Suppl 1):e20230127. doi: 10.1590/1678-4685-GMB-2023-0127 (PMC11346592; doi:10.1590/1678-4685-GMB-2023-0127)
Supplement: Table S2 - [file 1415-4757-GMB-46-03-s1-e20230127-s2.pdf]

Genetics and Molecular Biology

DOI: https://doi.org/10.1590/1678-4685-GMB-2023-0127

Supplementary Material to "From bench to *in silico* and backwards: what have we done on genetics of recurrent pregnancy loss and implantation failure and where should we go next?"

Table S2 - Database research for implantation failure.

**OMIM**	**HuGE**	**CTD**
CD46	ESR1	GPX4
CHKA	F2	GRK2
F5	F5	LCMT1
FKBP4	IFNG	LIF
PRLR	IL10	PADI6
UBTFL1	IL6	PARG
FRAS1	ITGB3	SLC31A1
POFUT2	MMP9	TLE6
FBXO43	MTHFR	UBE2N
	MTRR	
	ND1	
	NOS3	
	PROCR	
	SERPINE1	
	TGB1	
	TNF	
