# Supplementary material for: From bench to in silico and backwards: What have we done on genetics of recurrent pregnancy loss and implantation failure and where should we go next?
Source: Genet Mol Biol. 2024 Aug 26;46(3 Suppl 1):e20230127. doi: 10.1590/1678-4685-GMB-2023-0127 (PMC11346592; doi:10.1590/1678-4685-GMB-2023-0127)
Supplement: Table S3 - [file 1415-4757-GMB-46-03-s1-e20230127-s3.pdf]

Genetics and Molecular Biology
DOI: https://doi.org/10.1590/1678-4685-GMB-2023-0127

Supplementary Material to "From bench to *in silico* and backwards: what have we done on genetics of recurrent pregnancy loss and implantation failure and where should we go next?"

Table S3 - Comparison of the common proteins obtained from database research for recurrent pregnancy loss and implantation failure.

Gene	**RPL**			**IF**		
	**CTD**	**HuGE**	**OMIM**	**CTD**	**HuGE**	**OMIM**
F5	x	x	x		x	x
GPX4	x	x	x	x		
PRLR	x	x	x			x
NOS3	x	x	x		x	
F2	x	x	x		x	
LIF	x	x		x		
CD46		x	x			x
MTHFR		x	x		x	
TNF	x	x			x	
IL6	x	x			x	
SLC31A1	x			x		
LCMT1	x			x		
UBE2N	x			x		
PARG	x			x		
TLE6	x			x		
PADI6	x			x		
GRK2	x			x		
ND1		x			x	
IL10		x			x	
MTRR		x			x	
IFNG		x			x	
SERPINE1		x			x	
ITGB3		x			x	
ESR1		x			x	
PROCR		x			x	
MMP9		x			x	
