# Supplementary material for: From bench to in silico and backwards: What have we done on genetics of recurrent pregnancy loss and implantation failure and where should we go next?
Source: Genet Mol Biol. 2024 Aug 26;46(3 Suppl 1):e20230127. doi: 10.1590/1678-4685-GMB-2023-0127 (PMC11346592; doi:10.1590/1678-4685-GMB-2023-0127)
Supplement: Table S4 - [file 1415-4757-GMB-46-03-s1-e20230127-s4.pdf]

Genetics and Molecular Biology

DOI: https://doi.org/10.1590/1678-4685-GMB-2023-0127

Supplementary Material to "From bench to *in silico* and backwards: what have we done on genetics of recurrent pregnancy loss and implantation failure and where should we go next?"

Table S4 - Enriched gene ontologies for recurrent pregnancy loss + implantation failure network.

ID	Description	GeneRatio	BgRatio	pvalue	p.adjust	qvalue	geneID	Count
GO:0097191	extrinsic apoptotic signaling pathway	40/293	224/18670	1,13E-30	5,34E-27	2,35E-27	*SRC/AKT1/TRAF1/CASP3/NOS3/AR/GATA1/FGFR1/BAD/TNFRSF1A/LGALS3/HSPA1B/HSPA1A/JAK2/GSTP1/ACVR1/TGFBR1/IL1B/TNF/AGT/FGG/CASP9/BAX/BCL2/BCL2L1/FGB/FGA/TIMP3/SERPINE1/BRCA1/LCN2/TGFB1/IFI6/FASLG/FGFR3/IFNG/IGF1/IL12A/IL1A/IL6R*	40
GO:0007596	blood coagulation	46/293	336/18670	6,59E-30	1,56E-26	6,86E-27	*TMPRSS6/SRC/TRPC6/TLR4/NOS3/GATA2/GATA1/JAK2/ADRA2A/IL6/FGG/F2/PROC/HRG/SERPINA10/HBE1/SERPINC1/PROZ/HNF4A/FLNA/PRKG1/CPB2/F12/FGB/F7/FGA/APOE/SERPINA1/PLAT/ITGA2B/ITGB3/SERPINE1/F13A1/CD9/ITGA2/CEACAM1/COL1A1/FBLN1/COL1A2/PROCR/THBD/F10/TFPI/F5/F3/SLC4A1*	46
GO:0007599	hemostasis	46/293	341/18670	1,28E-29	1,72E-26	7,59E-27	*TMPRSS6/SRC/TRPC6/TLR4/NOS3/GATA2/GATA1/JAK2/ADRA2A/IL6/FGG/F2/PROC/HRG/SERPINA10/HBE1/SERPINC1/PROZ/HNF4A/FLNA/PRKG1/CPB2/F12/FGB/F7/FGA/APOE/SERPINA1/PLAT/ITGA2B/ITGB3/SERPINE1/F13A1/CD9/ITGA2/CEACAM1/COL1A1/FBLN1/COL1A2/PROCR/THBD/F10/TFPI/F5/F3/SLC4A1*	46
GO:0050817	coagulation	46/293	342/18670	1,46E-29	1,72E-26	7,59E-27	*TMPRSS6/SRC/TRPC6/TLR4/NOS3/GATA2/GATA1/JAK2/ADRA2A/IL6/FGG/F2/PROC/HRG/SERPINA10/HBE1/SERPINC1/PROZ/HNF4A/FLNA/PRKG1/CPB2/F12/FGB/F7/FGA/APOE/SERPINA1/PLAT/ITGA2B/ITGB3/SERPINE1/F13A1/CD9/ITGA2/CEACAM1/COL1A1/FBLN1/COL1A2/PROCR/THBD/F10/TFPI/F5/F3/SLC4A1*	46
GO:0001819	positive regulation of cytokine production	49/293	464/18670	1,42E-26	1,34E-23	5,90E-24	*NFKB1/AIF1/SRC/STAT3/TLR4/CHUK/HSPA1B/HSPA1A/JAK2/ADRA2A/IL1B/TNF/IL6/IL1R1/AGT/ARNT/HIF1A/MMP12/SERPINE1/HAVCR2/BRCA1/HMGB1/C5AR1/SLC11A1/CD14/HLA-E/HLA-G/LILRB1/CEBPB/CREB1/CHIA/TGFB1/IL12B/SULF1/EIF2AK2/INS/IL23R/FOXP3/IFNG/IL12A/IL1RN/IL1A/IL4R/IL4/LEP/LTA/TLR1/IL10/IL6R*	49

ID	Description	GeneRatio	BgRatio	pvalue	p.adjust	qvalue	geneID	Count
GO:0050673	epithelial cell proliferation	47/293	434/18670	5,60E-26	4,41E-23	1,94E-23	*AKT1/STAT3/EGFR/GATA2/MTOR/AR/NR2F2/FGFR1/BAD/ESR1/IGFBP3/PPARG/TGFBR1/BMP2/AGTR1/TNF/TP63/ARNT/HIF1A/BAX/CPB2/APOE/MMP12/IGFBP4/ITGB3/BRCA2/HMGB1/C5AR1/CEACAM1/CEBPB/VEGFA/PRL/FSHB/TGFB1/SULF1/PGR/F3/FGF1/FGF7/KDR/VEGFC/FLT1/VEGFB/PGF/IGF1/LEP/IL10*	47
GO:0022407	regulation of cell-cell adhesion	45/293	403/18670	1,90E-25	1,29E-22	5,66E-23	*AIF1/SRC/AKT1/CASP3/BAD/LGALS3/JAK2/BMP2/ADA/IL1B/TNF/IL6/FGG/PRKG1/FGB/FGA/HAVCR2/HMGB1/HLA-E/HLA-G/LILRB1/CD9/CEACAM1/CEACAM6/CEBPB/VEGFA/TGFB1/IL12B/CTLA4/EFNB1/IGF2/IL23R/FOXP3/IFNG/HFE/TFRC/IGF1/IL12A/IL1RN/IL4R/IL4/LEP/PDE5A/IL10/IL6R*	45
GO:0002237	response to molecule of bacterial origin	42/293	343/18670	2,26E-25	1,33E-22	5,87E-23	*NFKB1/SRC/AKT1/TLR4/CASP3/NOS3/CHUK/JAK2/GSTP1/IL1B/CCR5/TNF/IL6/IL10RA/REN/CASP9/APOB/SERPINE1/HAVCR2/HMGB1/C5AR1/SLC11A1/CD14/LTF/LILRB1/LCN2/CEBPB/TGFB1/TIMP4/IL12B/THBD/CXCL8/TFPI/FASLG/IL23R/HAMP/IL12A/IL1RN/LTA/NOS2/TLR1/IL10*	42
GO:0032496	response to lipopolysaccharide	41/293	330/18670	4,98E-25	2,61E-22	1,15E-22	*NFKB1/SRC/AKT1/TLR4/CASP3/NOS3/CHUK/JAK2/GSTP1/IL1B/CCR5/TNF/IL6/IL10RA/REN/CASP9/APOB/SERPINE1/HAVCR2/HMGB1/C5AR1/SLC11A1/CD14/LTF/LILRB1/LCN2/CEBPB/TGFB1/TIMP4/IL12B/THBD/CXCL8/TFPI/FASLG/IL23R/HAMP/IL12A/IL1RN/LTA/NOS2/IL10*	41
GO:0050678	regulation of epithelial cell proliferation	42/293	378/18670	1,07E-23	5,07E-21	2,24E-21	*AKT1/STAT3/EGFR/GATA2/MTOR/AR/NR2F2/FGFR1/BAD/PPARG/TGFBR1/BMP2/AGTR1/TNF/TP63/ARNT/HIF1A/BAX/CPB2/APOE/MMP12/ITGB3/BRCA2/HMGB1/C5AR1/CEACAM1/VEGFA/PRL/TGFB1/SULF1/PGR/F3/FGF1/FGF7/KDR/VEGFC/FLT1/VEGFB/PGF/IGF1/LEP/IL10*	42
GO:0050818	regulation of coagulation	23/293	84/18670	1,43E-22	6,13E-20	2,70E-20	*TMPRSS6/TLR4/NOS3/FGG/F2/PROC/HRG/SERPINC1/PRKG1/CPB2/F12/FGB/F7/FGA/APOE/PLAT/SERPINE1/CD9/CEACAM1/PROCR/THBD/TFPI/F3*	23
GO:0032103	positive regulation of response to external stimulus	38/293	323/18670	2,19E-22	8,60E-20	3,79E-20	*AIF1/TLR4/EGFR/FGFR1/TNFRSF1A/JAK2/IL1B/AGTR1/TNF/IL6/AGT/F2/HRG/IL16/CPB2/F12/F7/SERPINE1/HAVCR2/HMGB1/C5AR1/HLA-E/ITGA2/CEBPA/CEBPB/VEGFA/TGFB1/IL12B/THBD/CXCL8/F3/KDR/VEGFC/VEGFB/PGF/IL12A/LTA/IL6R*	38
GO:0042110	T cell activation	44/293	464/18670	5,26E-22	1,91E-19	8,43E-20	*TP53/AIF1/SRC/AKT1/STAT3/CASP3/MTOR/BAD/LGALS3/ADA/IL1B/IL6/LEPR/PRLR/BAX/BCL2/HAVCR2/HMGB1/SLC11A1/CD8A/HLA-E/HLA-G/LILRB1/CEACAM1/CEBPB/TGFB1/IL12B/CTLA4/EFNB1/INS/IGF2/IL23R/FOXP3/IFNG/HFE/TFRC/IGF1/IL12A/IL4R/IL4/LEP/PDE5A/IL10/IL6R*	44
GO:0032943	mononuclear cell proliferation	35/293	274/18670	7,14E-22	2,24E-19	9,88E-20	*ACE/TP53/AIF1/TLR4/CASP3/LGALS3/CD320/ADA/IL1B/IL6/AHR/BAX/BCL2/HAVCR2/HMGB1/SLC11A1/HLA-E/HLA-*	35

ID	Description	GeneRatio	BgRatio	pvalue	p.adjust	qvalue	geneID	Count
							G/LILRB1/CEBPB/TGFB1/IL12B/CTLA4/EFNB1/IGF2/IL23R/FOXP3/TFRC/IGF1/IL12A/IL4/LEP/PDE5A/IL10/IL6R	
GO:2001236	regulation of extrinsic apoptotic signaling pathway	28/293	155/18670	7,30E-22	2,24E-19	9,88E-20	*SRC/AKT1/TRAF1/NOS3/AR/GATA1/FGFR1/LGALS3/HSPA1B/HSPA1A/GSTP1/ACVR1/TGFBR1/IL1B/TNF/AGT/FGG/BCL2/BCL2L1/FGB/FGA/TIMP3/SERPINE1/BRCA1/IFI6/FASLG/IGF1/IL1A*	28
GO:0043062	extracellular structure organization	42/293	422/18670	7,81E-22	2,24E-19	9,88E-20	*TMPRSS6/TNFRSF1A/FN1/TGFBR1/AGTR1/TNF/IL6/AGT/FGG/MMP2/CPB2/FGB/FGA/APOB/APOE/MMP12/MMP3/ITGB4/ITGB6/ITGA2B/ITGB3/TIMP1/SERPINE1/MMP9/ITGA2/MMP1/MMP7/TGFB1/TIMP2/COL1A1/FBLN1/TGFBI/FBN1/COL5A1/COL6A3/COL5A2/COL6A1/COL1A2/SULF1/KDR/LAMA4/MMP15*	42
GO:0030193	regulation of blood coagulation	22/293	79/18670	8,06E-22	2,24E-19	9,88E-20	*TMPRSS6/TLR4/NOS3/FGG/F2/PROC/HRG/SERPINC1/PRKG1/CPB2/F12/FGB/F7/FGA/APOE/PLAT/SERPINE1/CD9/CEACAM1/THBD/TFPI/F3*	22
GO:0070663	regulation of leukocyte proliferation	32/293	222/18670	1,05E-21	2,73E-19	1,20E-19	*AIF1/TLR4/CASP3/LGALS3/GSTP1/CD320/ADA/IL1B/IL6/AHR/BCL2/HAVCR2/HMGB1/HLA-E/HLA-G/LILRB1/CEBPB/TGFB1/IL12B/CTLA4/EFNB1/IGF2/IL23R/FOXP3/TFRC/IGF1/IL12A/IL4/LEP/PDE5A/IL10/IL6R*	32
GO:1900046	regulation of hemostasis	22/293	80/18670	1,10E-21	2,73E-19	1,20E-19	*TMPRSS6/TLR4/NOS3/FGG/F2/PROC/HRG/SERPINC1/PRKG1/CPB2/F12/FGB/F7/FGA/APOE/PLAT/SERPINE1/CD9/CEACAM1/THBD/TFPI/F3*	22
GO:0070661	leukocyte proliferation	36/293	298/18670	1,22E-21	2,88E-19	1,27E-19	*ACE/TP53/AIF1/TLR4/CASP3/LGALS3/GSTP1/CD320/ADA/IL1B/IL6/AHR/BAX/BCL2/HAVCR2/HMGB1/SLC11A1/HLA-E/HLA-G/LILRB1/CEBPB/TGFB1/IL12B/CTLA4/EFNB1/IGF2/IL23R/FOXP3/TFRC/IGF1/IL12A/IL4/LEP/PDE5A/IL10/IL6R*	36
GO:2001237	negative regulation of extrinsic apoptotic signaling pathway	24/293	104/18670	1,46E-21	3,28E-19	1,44E-19	*SRC/AKT1/NOS3/AR/GATA1/LGALS3/HSPA1B/HSPA1A/GSTP1/ACVR1/TGFBR1/IL1B/TNF/FGG/BCL2/BCL2L1/FGB/FGA/SERPINE1/BRCA1/IFI6/FASLG/IGF1/IL1A*	24
GO:0018108	peptidyl-tyrosine phosphorylation	39/293	363/18670	1,63E-21	3,44E-19	1,52E-19	*ACE/TP53/SRC/STAT3/EGFR/MTOR/GATA1/FGFR1/TNFRSF1A/IGF1R/JAK2/ACVR1/ADRA2A/TNF/IL6/AGT/HRG/PRLR/ITGB3/VEGFA/TGFB1/IL12B/EIF2AK2/EPHB4/EGF/INSR/IGF2/FGFR3/FGF7/KDR/FLT1/IL23R/IFNG/IGF1/IL12A/IL4/LEP/ROR1/IL6R*	39
GO:0050670	regulation of lymphocyte proliferation	31/293	208/18670	1,69E-21	3,44E-19	1,52E-19	*AIF1/TLR4/CASP3/LGALS3/CD320/ADA/IL1B/IL6/AHR/BCL2/HAVCR2/HMGB1/HLA-E/HLA-G/LILRB1/CEBPB/TGFB1/IL12B/CTLA4/EFNB1/IGF2/IL23R/FOXP3/TFRC/IGF1/IL12A/IL4/LEP/PDE5A/IL10/IL6R*	31
GO:0002822	regulation of adaptive immune response based on somatic recombination of immune receptors	27/293	145/18670	1,75E-21	3,44E-19	1,52E-19	*ADA/IL1B/TNF/IL6/IL1R1/HAVCR2/HMGB1/SLC11A1/HLA-E/HLA-C/HLA-G/HLA-A/HLA-B/LILRB1/CEACAM1/TGFB1/IL12B/IL23R/FOXP3/HFE/TFRC/IL12A/IL4R/IL4/LTA/IL10/IL6R*	27

ID	Description	GeneRatio	BgRatio	pvalue	p.adjust	qvalue	geneID	Count
	built from immunoglobulin superfamily domains							
GO:0032944	regulation of mononuclear cell proliferation	31/293	209/18670	1,96E-21	3,71E-19	1,63E-19	*AIF1/TLR4/CASP3/LGALS3/CD320/ADA/IL1B/IL6/AHR/BCL2/HAVCR2/HMGB1/HLA-E/HLA-G/LILRB1/CEBPB/TGFB1/IL12B/CTLA4/EFNB1/IGF2/IL23R/FOXP3/TFRC/IGF1/IL12A/IL4/LEP/PDE5A/IL10/IL6R*	31
GO:0018212	peptidyl-tyrosine modification	39/293	366/18670	2,19E-21	3,98E-19	1,76E-19	*ACE/TP53/SRC/STAT3/EGFR/MTOR/GATA1/FGFR1/TNFRSF1A/IGF1R/JAK2/ACVR1/ADRA2A/TNF/IL6/AGT/HRG/PRLR/ITGB3/VEGFA/TGFB1/IL12B/EIF2AK2/EPHB4/EGF/INSR/IGF2/FGFR3/FGF7/KDR/FLT1/IL23R/IFNG/IGF1/IL12A/IL4/LEP/ROR1/IL6R*	39
GO:0048732	gland development	42/293	434/18670	2,29E-21	4,01E-19	1,77E-19	*SRC/AKT1/THRB/EGFR/GATA2/AR/FGFR1/ESR1/JAK2/TGFBR1/BMP2/ADA/TNF/TP63/SERPINC1/PRLR/HIF1A/HNF4A/BAX/BCL2/TYMS/CPB2/BRCA2/ITGA2/CEACAM1/CEBPA/CEBPB/VEGFA/CREB1/PRL/TGFB1/SULF1/EGF/INSR/IGF2/PGR/FGF7/HAMP/HFE/HNF1A/IGF2R/IL10*	42
GO:0030198	extracellular matrix organization	39/293	368/18670	2,67E-21	4,51E-19	1,99E-19	*TMPRSS6/TNFRSF1A/FN1/TGFBR1/TNF/IL6/AGT/FGG/MMP2/CPB2/FGB/FGA/MMP12/MMP3/ITGB4/ITGB6/ITGA2B/ITGB3/TIMP1/SERPINE1/MMP9/ITGA2/MMP1/MMP7/TGFB1/TIMP2/COL1A1/FBLN1/TGFBI/FBN1/COL5A1/COL6A3/COL5A2/COL6A1/COL1A2/SULF1/KDR/LAMA4/MMP15*	39
GO:0046651	lymphocyte proliferation	34/293	272/18670	5,77E-21	9,40E-19	4,14E-19	*TP53/AIF1/TLR4/CASP3/LGALS3/CD320/ADA/IL1B/IL6/AHR/BAX/BCL2/HAVCR2/HMGB1/SLC11A1/HLA-E/HLA-G/LILRB1/CEBPB/TGFB1/IL12B/CTLA4/EFNB1/IGF2/IL23R/FOXP3/TFRC/IGF1/IL12A/IL4/LEP/PDE5A/IL10/IL6R*	34
GO:0042098	T cell proliferation	29/293	184/18670	7,14E-21	1,12E-18	4,96E-19	*TP53/AIF1/CASP3/LGALS3/IL1B/IL6/BAX/HAVCR2/HMGB1/SLC11A1/HLA-E/HLA-G/LILRB1/CEBPB/TGFB1/IL12B/CTLA4/EFNB1/IGF2/IL23R/FOXP3/TFRC/IGF1/IL12A/IL4/LEP/PDE5A/IL10/IL6R*	29
GO:0022409	positive regulation of cell-cell adhesion	33/293	255/18670	7,49E-21	1,14E-18	5,03E-19	*AIF1/SRC/AKT1/BAD/ADA/IL1B/TNF/IL6/FGG/FGB/FGA/HAVCR2/HMGB1/HLA-E/HLA-G/LILRB1/CEACAM6/TGFB1/IL12B/CTLA4/EFNB1/IGF2/IL23R/FOXP3/IFNG/TFRC/IGF1/IL12A/IL4R/IL4/LEP/IL10/IL6R*	33
GO:0045785	positive regulation of cell adhesion	40/293	403/18670	8,85E-21	1,31E-18	5,76E-19	*AIF1/SRC/AKT1/BAD/JAK2/FN1/ADA/IL1B/TNF/IL6/FGG/HRG/FLNA/FGB/FGA/HAVCR2/HMGB1/HLA-E/HLA-G/LILRB1/ITGA2/CEACAM6/VEGFA/TGFB1/IL12B/CTLA4/EFNB1/IGF2/KDR/IL23R/FOXP3/IFNG/TFRC/IGF1/IL12A/IL4R/IL4/LEP/IL10/IL6R*	40
GO:0050819	negative regulation of coagulation	19/293	57/18670	1,09E-20	1,56E-18	6,85E-19	*TMPRSS6/NOS3/FGG/F2/PROC/HRG/PRKG1/CPB2/F12/FGB/FGA/APOE/PLAT/SERPINE1/CD9/CEACAM1/PROCR/THBD/TFPI*	19

ID	Description	GeneRatio	BgRatio	pvalue	p.adjust	qvalue	geneID	Count
GO:1903037	regulation of leukocyte cell-cell adhesion	35/293	304/18670	2,27E-20	3,15E-18	1,39E-18	*AIF1/SRC/AKT1/CASP3/BAD/LGALS3/ADA/IL1B/TNF/IL6/HAVCR2/HMGB1/HLA-E/HLA-G/LILRB1/CEACAM1/CEBPB/TGFB1/IL12B/CTLA4/EFNB1/IGF2/IL23R/FOXP3/IFNG/HFE/TFRC/IGF1/IL12A/IL4R/IL4/LEP/PDE5A/IL10/IL6R*	35
GO:0002819	regulation of adaptive immune response	27/293	160/18670	2,62E-20	3,54E-18	1,56E-18	*ADA/IL1B/TNF/IL6/IL1R1/HAVCR2/HMGB1/SLC11A1/HLA-E/HLA-C/HLA-G/HLA-A/HLA-B/LILRB1/CEACAM1/TGFB1/IL12B/IL23R/FOXP3/HFE/TFRC/IL12A/IL4R/IL4/LTA/IL10/IL6R*	27
GO:1903532	positive regulation of secretion by cell	39/293	399/18670	4,86E-20	6,38E-18	2,81E-18	*AIF1/SRC/TLR4/EGFR/GATA2/CHUK/FGFR1/BAD/JAK2/IL1B/TNF/IL6/AGT/FGG/RBP4/HIF1A/INHA/CPB2/FGB/FGA/MMP12/HAVCR2/HMGB1/CD14/HLA-E/CREB1/CFTR/CHIA/TGFB1/INS/VEGFC/IFNG/HFE/IGF1/IL1A/IL4R/LEP/TLR1/IL10*	39
GO:0050900	leukocyte migration	43/293	499/18670	6,53E-20	8,34E-18	3,67E-18	*AIF1/SRC/AKT1/LGALS3/FN1/L1CAM/ADA/IL1B/CCR5/TNF/IL6/IL1R1/F2/IL16/F7/APOB/ITGA2B/ITGB3/SERPINE1/HMGB1/C5AR1/CD9/ITGA2/CEACAM1/CEACAM6/MMP1/CEACAM5/VEGFA/TGFB1/COL1A1/COL1A2/THBD/CXCL8/CXCR1/VEGFC/FLT1/VEGFB/PGF/IL12A/IL1RN/LEP/IL10/IL6R*	43
GO:0030195	negative regulation of blood coagulation	18/293	53/18670	7,79E-20	9,57E-18	4,22E-18	*TMPRSS6/NOS3/FGG/F2/PROC/HRG/PRKG1/CPB2/F12/FGB/FGA/APOE/PLAT/SERPINE1/CD9/CEACAM1/THBD/TFPI*	18
GO:0051047	positive regulation of secretion	40/293	428/18670	7,90E-20	9,57E-18	4,22E-18	*AIF1/SRC/TLR4/EGFR/GATA2/CHUK/FGFR1/BAD/JAK2/IL1B/TNF/IL6/AGT/FGG/RBP4/HIF1A/INHA/CPB2/FGB/FGA/MMP12/HAVCR2/HMGB1/CD14/HLA-E/CREB1/CFTR/PRL/CHIA/TGFB1/INS/VEGFC/IFNG/HFE/IGF1/IL1A/IL4R/LEP/TLR1/IL10*	40
GO:1900047	negative regulation of hemostasis	18/293	54/18670	1,15E-19	1,36E-17	6,00E-18	*TMPRSS6/NOS3/FGG/F2/PROC/HRG/PRKG1/CPB2/F12/FGB/FGA/APOE/PLAT/SERPINE1/CD9/CEACAM1/THBD/TFPI*	18
GO:0050679	positive regulation of epithelial cell proliferation	29/293	206/18670	1,81E-19	2,08E-17	9,18E-18	*AKT1/STAT3/EGFR/GATA2/MTOR/AR/FGFR1/BAD/TGFBR1/BMP2/AGTR1/TP63/ARNT/HIF1A/MMP12/ITGB3/HMGB1/C5AR1/VEGFA/TGFB1/F3/FGF1/FGF7/KDR/VEGFC/VEGFB/PGF/IGF1/IL10*	29
GO:0042129	regulation of T cell proliferation	26/293	156/18670	1,87E-19	2,10E-17	9,26E-18	*AIF1/CASP3/LGALS3/IL1B/IL6/HAVCR2/HMGB1/HLA-E/HLA-G/LILRB1/CEBPB/TGFB1/IL12B/CTLA4/EFNB1/IGF2/IL23R/FOXP3/TFRC/IGF1/IL12A/IL4/LEP/PDE5A/IL10/IL6R*	26
GO:0050731	positive regulation of peptidyl-tyrosine phosphorylation	28/293	192/18670	2,97E-19	3,26E-17	1,44E-17	*ACE/TP53/SRC/STAT3/MTOR/GATA1/TNFRSF1A/JAK2/ACVR1/ADRA2A/TNF/IL6/AGT/ITGB3/VEGFA/TGFB1/IL12B/EGF/IGF2/FGFR3/FGF7/IL23R/IFNG/IGF1/IL12A/IL4/LEP/IL6R*	28

ID	Description	GeneRatio	BgRatio	pvalue	p.adjust	qvalue	geneID	Count
GO:0001936	regulation of endothelial cell proliferation	27/293	176/18670	3,44E-19	3,69E-17	1,63E-17	*AKT1/STAT3/GATA2/MTOR/NR2F2/FGFR1/PPARG/TGFBR1/BMP2/AGTR1/TNF/ARNT/HIF1A/APOE/ITGB3/HMGB1/VEGFA/PRL/SULF1/F3/KDR/VEGFC/FLT1/VEGFB/PGF/LEP/IL10*	27
GO:0050863	regulation of T cell activation	34/293	314/18670	5,76E-19	6,05E-17	2,67E-17	*AIF1/SRC/AKT1/CASP3/BAD/LGALS3/ADA/IL1B/IL6/HAVCR2/HMGB1/HLA-E/HLA-G/LILRB1/CEACAM1/CEBPB/TGFB1/IL12B/CTLA4/EFNB1/IGF2/IL23R/FOXP3/IFNG/HFE/TFRC/IGF1/IL12A/IL4R/IL4/LEP/PDE5A/IL10/IL6R*	34
GO:0007159	leukocyte cell-cell adhesion	35/293	337/18670	6,54E-19	6,72E-17	2,96E-17	*AIF1/SRC/AKT1/CASP3/BAD/LGALS3/ADA/IL1B/TNF/IL6/HAVCR2/HMGB1/HLA-E/HLA-G/LILRB1/CEACAM1/CEBPB/TGFB1/IL12B/CTLA4/EFNB1/IGF2/IL23R/FOXP3/IFNG/HFE/TFRC/IGF1/IL12A/IL4R/IL4/LEP/PDE5A/IL10/IL6R*	35
GO:2001233	regulation of apoptotic signaling pathway	38/293	406/18670	6,77E-19	6,81E-17	3,00E-17	*TP53/SRC/AKT1/TRAF1/MDM2/NOS3/AR/GATA1/FGFR1/BAD/LGALS3/HSPA1B/HSPA1A/JAK2/GSTP1/ACVR1/TGFBR1/IL1B/TNF/TP63/TP73/AGT/FGG/HIF1A/BAX/BCL2/BCL2L1/FGB/FGA/TIMP3/SERPINE1/MMP9/BRCA1/IFI6/INS/FASLG/IGF1/IL1A*	38
GO:1904951	positive regulation of establishment of protein localization	40/293	456/18670	7,67E-19	7,55E-17	3,33E-17	*HSPA1L/TP53/AIF1/SRC/TLR4/EGFR/MDM2/CHUK/BAD/JAK2/RAN/IL1B/TNF/IL6/TP63/TP73/FGG/RBP4/HIF1A/FLNA/BCL2/FGB/FGA/MMP12/HAVCR2/HMGB1/CD14/HLA-E/CFTR/CHIA/TGFB1/INS/VEGFC/IFNG/IGF1/IL1A/IL4R/LEP/TLR1/IL10*	40
GO:0072593	reactive oxygen species metabolic process	32/293	284/18670	2,02E-18	1,95E-16	8,59E-17	*ACE2/TP53/AIF1/AKT1/STAT3/TLR4/EGFR/NOS3/MTOR/JAK2/GSTP1/IL1B/AGTR1/TNF/AGT/F2/HBE1/HIF1A/BCL2/MMP3/BRCA1/TGFB1/DHFR/IFI6/INSR/INS/IFNG/HBA1/HP/LEP/NOS2/IL10*	32
GO:0001935	endothelial cell proliferation	27/293	191/18670	3,02E-18	2,85E-16	1,25E-16	*AKT1/STAT3/GATA2/MTOR/NR2F2/FGFR1/PPARG/TGFBR1/BMP2/AGTR1/TNF/ARNT/HIF1A/APOE/ITGB3/HMGB1/VEGFA/PRL/SULF1/F3/KDR/VEGFC/FLT1/VEGFB/PGF/LEP/IL10*	27
GO:0002793	positive regulation of peptide secretion	32/293	288/18670	3,07E-18	2,85E-16	1,25E-16	*AIF1/SRC/TLR4/EGFR/CHUK/BAD/JAK2/IL1B/TNF/IL6/FGG/RBP4/HIF1A/FGB/FGA/MMP12/HAVCR2/HMGB1/CD14/HLA-E/CFTR/CHIA/TGFB1/INS/VEGFC/IFNG/HFE/IGF1/IL1A/IL4R/TLR1/IL10*	32
GO:0050714	positive regulation of protein secretion	31/293	268/18670	3,31E-18	3,01E-16	1,32E-16	*AIF1/SRC/TLR4/EGFR/CHUK/BAD/JAK2/IL1B/TNF/IL6/FGG/RBP4/HIF1A/FGB/FGA/MMP12/HAVCR2/HMGB1/CD14/HLA-E/CFTR/CHIA/TGFB1/INS/VEGFC/IFNG/IGF1/IL1A/IL4R/TLR1/IL10*	31

ID	Description	GeneRatio	BgRatio	pvalue	p.adjust	qvalue	geneID	Count
GO:2001234	negative regulation of apoptotic signaling pathway	29/293	230/18670	3,97E-18	3,54E-16	1,56E-16	*SRC/AKT1/MDM2/NOS3/AR/GATA1/LGALS3/HSPA1B/HSPA1A/GSTP1/ACVR1/TGFBR1/IL1B/TNF/FGG/HIF1A/BAX/BCL2/BCL2L1/FGB/FGA/SERPINE1/MMP9/BRCA1/IFI6/INS/FASLG/IGF1/IL1A*	29
GO:0071219	cellular response to molecule of bacterial origin	28/293	212/18670	4,42E-18	3,87E-16	1,70E-16	*NFKB1/SRC/AKT1/TLR4/NOS3/GSTP1/IL1B/CCR5/TNF/IL6/SERPINE1/HAVCR2/HMGB1/CD14/LTF/LILRB1/LCN2/CEBPB/TGFB1/IL12B/CXCL8/TFPI/HAMP/IL12A/IL1RN/NOS2/TLR1/IL10*	28
GO:0048608	reproductive structure development	38/293	431/18670	5,19E-18	4,39E-16	1,94E-16	*SRC/AKT1/EGFR/CASP3/NOS3/GATA2/AR/NR2F2/GATA1/ESR1/PPARG/TGFBR1/ADA/TP63/REN/RBP4/ARNT/HIF1A/FLNA/BAX/BCL2/BCL2L1/INHA/BRIP1/BRCA2/NSDHL/CEBPA/CEBPB/VEGFA/LHB/FSHB/FSHR/SULF1/INSR/IGF2/PGR/LEP/IL10*	38
GO:2000377	regulation of reactive oxygen species metabolic process	27/293	195/18670	5,21E-18	4,39E-16	1,94E-16	*ACE2/TP53/AIF1/AKT1/STAT3/TLR4/EGFR/MTOR/JAK2/GSTP1/IL1B/AGTR1/TNF/AGT/F2/HIF1A/BCL2/MMP3/BRCA1/TGFB1/DHFR/INSR/INS/IFNG/HP/LEP/IL10*	27
GO:0046427	positive regulation of JAK-STAT cascade	20/293	89/18670	6,43E-18	5,33E-16	2,35E-16	*STAT3/TNFRSF1A/JAK2/TNF/IL6/IL10RA/AGT/F2/PRLR/PRL/IL12B/FGFR3/IL23R/IFNG/IGF1/IL12A/IL4/LEP/IL10/IL6R*	20
GO:0061458	reproductive system development	38/293	434/18670	6,56E-18	5,35E-16	2,36E-16	*SRC/AKT1/EGFR/CASP3/NOS3/GATA2/AR/NR2F2/GATA1/ESR1/PPARG/TGFBR1/ADA/TP63/REN/RBP4/ARNT/HIF1A/FLNA/BAX/BCL2/BCL2L1/INHA/BRIP1/BRCA2/NSDHL/CEBPA/CEBPB/VEGFA/LHB/FSHB/FSHR/SULF1/INSR/IGF2/PGR/LEP/IL10*	38
GO:0071216	cellular response to biotic stimulus	29/293	236/18670	8,09E-18	6,47E-16	2,85E-16	*NFKB1/TP53/SRC/AKT1/TLR4/NOS3/GSTP1/IL1B/CCR5/TNF/IL6/SERPINE1/HAVCR2/HMGB1/CD14/LTF/LILRB1/LCN2/CEBPB/TGFB1/IL12B/CXCL8/TFPI/HAMP/IL12A/IL1RN/NOS2/TLR1/IL10*	29
GO:0050730	regulation of peptidyl-tyrosine phosphorylation	30/293	256/18670	8,22E-18	6,47E-16	2,85E-16	*ACE/TP53/SRC/STAT3/EGFR/MTOR/GATA1/TNFRSF1A/JAK2/ACVR1/ADRA2A/TNF/IL6/AGT/HRG/ITGB3/VEGFA/TGFB1/IL12B/EGF/IGF2/FGFR3/FGF7/IL23R/IFNG/IGF1/IL12A/IL4/LEP/IL6R*	30
GO:0006953	acute-phase response	16/293	47/18670	8,73E-18	6,76E-16	2,98E-16	*STAT3/FN1/IL1B/TNF/IL6/CD163/F2/MBL2/SERPINA1/CEBPB/INS/HAMP/HP/HFE/IL1A/IL6R*	16
GO:0061041	regulation of wound healing	24/293	148/18670	9,29E-18	7,03E-16	3,10E-16	*TMPRSS6/TLR4/NOS3/MTOR/ADRA2A/FGG/F2/PROC/HRG/SERPINC1/PRKG1/CPB2/F12/FGB/F7/FGA/APOE/PLAT/SERPINE1/CD9/CEACAM1/THBD/TFPI/F3*	24
GO:1903039	positive regulation of leukocyte cell-cell adhesion	28/293	218/18670	9,38E-18	7,03E-16	3,10E-16	*AIF1/SRC/AKT1/BAD/ADA/IL1B/TNF/IL6/HAVCR2/HMGB1/HLA-E/HLA-G/LILRB1/TGFB1/IL12B/CTLA4/EFNB1/IGF2/IL23R/FOXP3/IFNG/TFRC/IGF1/IL12A/IL4R/IL4/LEP/IL6R*	28
GO:0002706	regulation of lymphocyte mediated immunity	24/293	149/18670	1,09E-17	8,02E-16	3,54E-16	*IL1B/TNF/IL6/IL1R1/HAVCR2/HMGB1/HLA-E/HLA-C/HLA-G/HLA-A/HLA-B/LILRB1/CEACAM1/TGFB1/IL12B/IL23R/FOXP3/HFE/TFRC/IL12A/IL4/LEP/LTA/IL10*	24

ID	Description	GeneRatio	BgRatio	pvalue	p.adjust	qvalue	geneID	Count
GO:0030099	myeloid cell differentiation	37/293	416/18670	1,10E-17	8,02E-16	3,54E-16	*SRC/STAT3/STAT5B/TLR4/CASP3/GATA2/MTOR/GATA1/LGALS3/HSPA1B/HSPA1A/PPARG/JAK2/TNF/ARNT/HIF1A/CASP9/INHA/ITGA2B/MMP9/UBD/LTF/LILRB1/CEACAM1/CEBPA/CEBPB/VEGFA/CREB1/FSHB/TGFB1/FBN1/IL12B/IL23R/IFNG/SLC4A1/TFRC/IL4*	37
GO:0002703	regulation of leukocyte mediated immunity	27/293	201/18670	1,15E-17	8,24E-16	3,63E-16	*TLR4/GATA2/IL1B/TNF/IL6/IL1R1/HAVCR2/HMGB1/HLA-E/HLA-C/HLA-G/HLA-A/HLA-B/LILRB1/CEACAM1/TGFB1/IL12B/IL23R/FOXP3/HFE/TFRC/IL12A/IL4R/IL4/LEP/LTA/IL10*	27
GO:0051222	positive regulation of protein transport	37/293	418/18670	1,29E-17	8,98E-16	3,96E-16	*HSPA1L/TP53/AIF1/SRC/TLR4/EGFR/MDM2/CHUK/BAD/JAK2/RAN/IL1B/TNF/IL6/FGG/RBP4/HIF1A/FLNA/FGB/FGA/MMP12/HAVCR2/HMGB1/CD14/HLA-E/CFTR/CHIA/TGFB1/INS/VEGFC/IFNG/IGF1/IL1A/IL4R/LEP/TLR1/IL10*	37
GO:1904894	positive regulation of STAT cascade	20/293	92/18670	1,30E-17	8,98E-16	3,96E-16	*STAT3/TNFRSF1A/JAK2/TNF/IL6/IL10RA/AGT/F2/PRLR/PRL/IL12B/FGFR3/IL23R/IFNG/IGF1/IL12A/IL4/LEP/IL10/IL6R*	20
GO:0050870	positive regulation of T cell activation	27/293	202/18670	1,31E-17	8,98E-16	3,96E-16	*AIF1/SRC/AKT1/BAD/ADA/IL1B/IL6/HAVCR2/HMGB1/HLA-E/HLA-G/LILRB1/TGFB1/IL12B/CTLA4/EFNB1/IGF2/IL23R/FOXP3/IFNG/TFRC/IGF1/IL12A/IL4R/IL4/LEP/IL6R*	27
GO:0050708	regulation of protein secretion	39/293	472/18670	1,71E-17	1,16E-15	5,09E-16	*AIF1/SRC/TLR4/EGFR/CHUK/BAD/JAK2/FN1/ADRA2A/IL1B/TNF/IL6/FGG/RBP4/HIF1A/HNF4A/FGB/FGA/APOE/MMP12/HAVCR2/HMGB1/CD14/HLA-E/LILRB1/CFTR/CHIA/TGFB1/INS/VEGFC/FOXP3/IFNG/IGF1/IL1A/IL4R/LEP/NOS2/TLR1/IL10*	39
GO:0071222	cellular response to lipopolysaccharide	27/293	205/18670	1,92E-17	1,28E-15	5,64E-16	*NFKB1/SRC/AKT1/TLR4/NOS3/GSTP1/IL1B/CCR5/TNF/IL6/SERPINE1/HAVCR2/HMGB1/CD14/LTF/LILRB1/LCN2/CEBPB/TGFB1/IL12B/CXCL8/TFPI/HAMP/IL12A/IL1RN/NOS2/IL10*	27
GO:0002791	regulation of peptide secretion	40/293	500/18670	1,97E-17	1,29E-15	5,70E-16	*AIF1/SRC/TLR4/EGFR/CHUK/BAD/JAK2/FN1/ADRA2A/IL1B/TNF/IL6/FGG/RBP4/HIF1A/HNF4A/FGB/FGA/APOE/MMP12/HAVCR2/HMGB1/CD14/HLA-E/LILRB1/CFTR/CHIA/TGFB1/INS/VEGFC/FOXP3/IFNG/HFE/IGF1/IL1A/IL4R/LEP/NOS2/TLR1/IL10*	40
GO:0051249	regulation of lymphocyte activation	39/293	485/18670	4,32E-17	2,80E-15	1,23E-15	*AIF1/SRC/AKT1/TLR4/CASP3/BAD/LGALS3/CD320/ADA/IL1B/IL6/AHR/BCL2/INHA/HAVCR2/HMGB1/HLA-E/HLA-G/LILRB1/CEACAM1/CEBPB/TGFB1/IL12B/CTLA4/EFNB1/IGF2/IL23R/FOXP3/IFNG/HFE/TFRC/IGF1/IL12A/IL4R/IL4/LEP/PDE5A/IL10/IL6R*	39
GO:0002576	platelet degranulation	22/293	128/18670	6,18E-17	3,95E-15	1,74E-15	*FN1/FGG/HRG/FLNA/FGB/FGA/SERPINA1/ITGA2B/ITGB3/TIMP1/TIMP3/SERPINE1/F13A1/CD9/VEGFA/TGFB1/EGF/IGF2/F5/VEGFC/VEGFB/IGF1*	22

ID	Description	GeneRatio	BgRatio	pvalue	p.adjust	qvalue	geneID	Count
GO:0002824	positive regulation of adaptive immune response based on somatic recombination of immune receptors built from immunoglobulin superfamily domains	20/293	100/18670	7,44E-17	4,68E-15	2,06E-15	*ADA/IL1B/TNF/IL6/IL1R1/SLC11A1/HLA-E/HLA-C/HLA-G/HLA-A/HLA-B/TGFB1/IL12B/IL23R/FOXP3/TFRC/IL12A/IL4/LTA/IL6R*	20
GO:1903034	regulation of response to wounding	25/293	179/18670	7,53E-17	4,68E-15	2,06E-15	*TMPRSS6/TLR4/NOS3/MTOR/ADRA2A/FGG/F2/PROC/HRG/SERPINC1/PRKG1/CPB2/F12/FGB/F7/FGA/APOE/PLAT/SERPINE1/CD9/CEACAM1/THBD/TFPI/F3/IL10*	25
GO:0033002	muscle cell proliferation	28/293	239/18670	1,09E-16	6,62E-15	2,92E-15	*PIM1/AIF1/AKT1/STAT3/EGFR/MDM2/MTOR/FGFR1/IGFBP3/PPARG/JAK2/GSTP1/TGFBR1/TNF/IL6/TP73/AGT/RBP4/PRKG1/MMP2/MMP9/ITGA2/IL12B/IFNG/IGF1/IL12A/IL10/IL6R*	28
GO:0061045	negative regulation of wound healing	18/293	76/18670	1,09E-16	6,62E-15	2,92E-15	*TMPRSS6/NOS3/FGG/F2/PROC/HRG/PRKG1/CPB2/F12/FGB/FGA/APOE/PLAT/SERPINE1/CD9/CEACAM1/THBD/TFPI*	18
GO:1903706	regulation of hemopoiesis	38/293	475/18670	1,34E-16	8,03E-15	3,54E-15	*PIM1/ACE/STAT3/STAT5B/TLR4/GATA2/MTOR/GATA1/BAD/LGALS3/HSPA1B/HSPA1A/ADA/TNF/TP73/ARNT/HIF1A/INHA/ITGA2B/HMGB1/LTF/HLA-G/LILRB1/CEACAM1/CEBPB/CREB1/FSHB/TGFB1/FBN1/IL12B/CTLA4/EIF2AK2/IL23R/FOXP3/IFNG/IL12A/IL4R/IL4*	38
GO:0002821	positive regulation of adaptive immune response	20/293	105/18670	2,04E-16	1,21E-14	5,32E-15	*ADA/IL1B/TNF/IL6/IL1R1/SLC11A1/HLA-E/HLA-C/HLA-G/HLA-A/HLA-B/TGFB1/IL12B/IL23R/FOXP3/TFRC/IL12A/IL4/LTA/IL6R*	20
GO:0032147	activation of protein kinase activity	32/293	333/18670	2,21E-16	1,29E-14	5,67E-15	*SRC/AKT1/TLR4/EGFR/MTOR/IKBKG/JAK2/TGFBR1/BMP2/ADRA2A/ADRB2/IL1B/TNF/UBE2N/TP73/AGT/PRLR/ITGB3/C5AR1/SLC11A1/VEGFA/IL12B/EIF2AK2/EGF/INSR/INS/FGF1/IL23R/IGF1/IL4/LEP/IL6R*	32
GO:0007162	negative regulation of cell adhesion	30/293	289/18670	2,44E-16	1,40E-14	6,18E-15	*SRC/AKT1/CASP3/LGALS3/JAK2/BMP2/FGG/HRG/PRKG1/MMP12/SERPINE1/HAVCR2/HMGB1/HLA-G/LILRB1/CD9/CEACAM1/CEBPB/VEGFA/TGFB1/COL1A1/FBLN1/TGFBI/CTLA4/FOXP3/HFE/IL1RN/IL4R/PDE5A/IL10*	30
GO:0050727	regulation of inflammatory response	38/293	485/18670	2,67E-16	1,52E-14	6,70E-15	*ACE2/NFKB1/TLR4/EGFR/ESR1/TNFRSF1A/PPARG/JAK2/GSTP1/ADA/IL1B/AGTR1/TNF/IL6/IL1R1/AGT/F2/PROC/CPB2/F12/APOE/MMP3/SERPINE1/MMP9/C5AR1/HLA-E/ITGA2/CEBPA/CEBPB/IL12B/INS/FOXP3/IGF1/IL4/LEP/LTA/NOS2/IL10*	38
GO:0046425	regulation of JAK-STAT cascade	22/293	137/18670	2,78E-16	1,56E-14	6,88E-15	*STAT3/VHL/TNFRSF1A/JAK2/TNF/IL6/IL10RA/AGT/F2/PRLR/PRL/IL12B/EGF/FGFR3/IL23R/IFNG/IGF1/IL12A/IL4/LEP/IL10/IL6R*	22

ID	Description	GeneRatio	BgRatio	pvalue	p.adjust	qvalue	geneID	Count
GO:0048545	response to steroid hormone	34/293	385/18670	3,16E-16	1,75E-14	7,73E-15	*ACTA1/AIF1/SRC/THRB/NR3C1/EGFR/MDM2/CASP3/ESR2/AR/NR2F2/BAD/ESR1/PPARG/JAK2/GSTP1/RAN/TNF/IL6/TP63/HNF4A/CASP9/BCL2/TYMS/BRCA1/CNOT1/RXRB/TGFB1/COL1A1/PGR/TFPI/PAPPA/IL1RN/IL10*	34
GO:0002460	adaptive immune response based on somatic recombination of immune receptors built from immunoglobulin superfamily domains	33/293	361/18670	3,22E-16	1,77E-14	7,78E-15	*STAT3/TLR4/MTOR/ADA/IL1B/TNF/IL6/IL1R1/MBL2/HAVCR2/HMGB1/SLC11A1/CD8A/HLA-E/HLA-C/HLA-G/HLA-A/HLA-B/LILRB1/CEACAM1/TGFB1/IL12B/IL23R/FOXP3/HFE/TFRC/HLA-DQB1/IL12A/IL4R/IL4/LTA/IL10/IL6R*	33
GO:0007259	JAK-STAT cascade	23/293	156/18670	4,00E-16	2,17E-14	9,58E-15	*STAT3/STAT5B/VHL/TNFRSF1A/JAK2/TNF/IL6/IL10RA/AGT/F2/PRLR/PRL/IL12B/EGF/FGFR3/IL23R/IFNG/IGF1/IL12A/IL4/LEP/IL10/IL6R*	23
GO:0007565	female pregnancy	25/293	192/18670	4,10E-16	2,20E-14	9,71E-15	*AKT1/MTOR/AR/NR2F2/ESR1/IL1B/AGT/PRLR/MMP2/BCL2/TIMP1/MMP9/HAVCR2/ITGA2/PRL/FSHB/MMP7/TGFB1/FBLN1/THBD/PGR/PGF/HFE/PAPPA/LEP*	25
GO:0032102	negative regulation of response to external stimulus	33/293	365/18670	4,45E-16	2,36E-14	1,04E-14	*TMPRSS6/NFKB1/AIF1/NOS3/TNFRSF1A/PPARG/GSTP1/ADA/FGG/F2/PROC/HRG/PRKG1/CPB2/F12/FGB/FGA/APOE/PLAT/SERPINE1/HAVCR2/LTF/CD9/CEACAM1/IL12B/THBD/INS/TFPI/FOXP3/IGF1/IL4/LEP/IL10*	33
GO:0050867	positive regulation of cell activation	34/293	394/18670	6,34E-16	3,33E-14	1,47E-14	*AIF1/SRC/AKT1/TLR4/GATA2/BAD/JAK2/CD320/ADA/IL1B/IL6/BCL2/HAVCR2/HMGB1/HLA-E/HLA-G/LILRB1/CEBPA/TGFB1/IL12B/CTLA4/EFNB1/IGF2/IL23R/FOXP3/IFNG/TFRC/IGF1/IL12A/IL4R/IL4/LEP/IL10/IL6R*	34
GO:0048771	tissue remodeling	24/293	179/18670	8,26E-16	4,29E-14	1,89E-14	*ACE/TP53/SRC/EGFR/MDM2/NOS3/ADRB2/IL6/AGT/HRG/LEPR/HIF1A/MMP2/BAX/ITGB3/TIMP1/HMGB1/CEACAM1/FSHB/TGFB1/IL12B/TFRC/IL1A/LEP*	24
GO:1904892	regulation of STAT cascade	22/293	146/18670	1,11E-15	5,72E-14	2,52E-14	*STAT3/VHL/TNFRSF1A/JAK2/TNF/IL6/IL10RA/AGT/F2/PRLR/PRL/IL12B/EGF/FGFR3/IL23R/IFNG/IGF1/IL12A/IL4/LEP/IL10/IL6R*	22
GO:0050671	positive regulation of lymphocyte proliferation	21/293	130/18670	1,19E-15	6,03E-14	2,66E-14	*AIF1/TLR4/CD320/ADA/IL1B/IL6/BCL2/HAVCR2/HMGB1/HLA-E/IL12B/EFNB1/IGF2/IL23R/FOXP3/TFRC/IGF1/IL12A/IL4/LEP/IL6R*	21
GO:0032946	positive regulation of mononuclear cell proliferation	21/293	131/18670	1,39E-15	7,01E-14	3,09E-14	*AIF1/TLR4/CD320/ADA/IL1B/IL6/BCL2/HAVCR2/HMGB1/HLA-E/IL12B/EFNB1/IGF2/IL23R/FOXP3/TFRC/IGF1/IL12A/IL4/LEP/IL6R*	21
GO:0002696	positive regulation of leukocyte activation	33/293	380/18670	1,45E-15	7,22E-14	3,18E-14	*AIF1/SRC/AKT1/TLR4/GATA2/BAD/CD320/ADA/IL1B/IL6/BCL2/HAVCR2/HMGB1/HLA-E/HLA-G/LILRB1/CEBPA/TGFB1/IL12B/CTLA4/EFNB1/IGF2/IL2*	33

ID	Description	GeneRatio	BgRatio	pvalue	p.adjust	qvalue	geneID	Count
							3R/FOXP3/IFNG/TFRC/IGF1/IL12A/IL4R/IL4/LEP/IL10/IL6R	
GO:0048871	multicellular organismal homeostasis	37/293	485/18670	1,60E-15	7,85E-14	3,46E-14	SRC/STAT3/TLR4/EGFR/NOS3/GATA2/GATA1/IGF1R/JAK2/ADRB2/IL1B/TNF/IL6/TP63/RBP4/LEPR/PRLR/BAX/BCL2/APC/ITGB3/SLC11A1/LTF/LCN2/CEBPB/VEGFA/CFTR/CRTC1/FSHB/HAMP/HFE/TFRC/IL1A/IL4R/IL4/LEP/LAMA4	37
GO:0097696	STAT cascade	23/293	166/18670	1,62E-15	7,88E-14	3,47E-14	STAT3/STAT5B/VHL/TNFRSF1A/JAK2/TNF/IL6/IL10RA/AGT/F2/PRLR/PRL/IL12B/EGF/FGFR3/IL23R/IFNG/IGF1/IL12A/IL4/LEP/IL10/IL6R	23
GO:0062012	regulation of small molecule metabolic process	36/293	459/18670	1,68E-15	8,11E-14	3,57E-14	NFKB1/TP53/SRC/AKT1/STAT3/NOS3/MTOR/BAD/IGFBP3/PPARG/CD320/BMP2/RAN/IL1B/TNF/ARNT/LEPR/HIF1A/LCMT1/APOB/APOE/IGFBP4/BRCA1/CEACAM1/TGFB1/DHCR7/EGF/INSR/INS/IGF2/FGF1/IFNG/IGF1/IL4/LEP/NOS2	36
GO:0002573	myeloid leukocyte differentiation	25/293	204/18670	1,75E-15	8,33E-14	3,67E-14	SRC/TLR4/GATA2/MTOR/GATA1/PPARG/TNF/INHA/MMP9/UBD/LTF/LILRB1/CEACAM1/CEBPA/CEBPB/VEGFA/CREB1/FSHB/TGFB1/FBN1/IL12B/IL23R/IFNG/TFRC/IL4	25
GO:0072378	blood coagulation, fibrin clot formation	12/293	27/18670	2,51E-15	1,19E-13	5,23E-14	FGG/F2/SERPINC1/F12/FGB/F7/FGA/F13A1/FBLN1/F10/TFPI/F3	12
GO:1903035	negative regulation of response to wounding	18/293	90/18670	2,71E-15	1,27E-13	5,60E-14	TMPRSS6/NOS3/FGG/F2/PROC/HRG/PRKG1/CPB2/F12/FGB/FGA/APOE/PLAT/SERPINE1/CD9/CEACAM1/THBD/TFPI	18
GO:0030168	platelet activation	22/293	153/18670	3,06E-15	1,42E-13	6,25E-14	SRC/TRPC6/TLR4/NOS3/GATA1/ADRA2A/IL6/FGG/F2/HRG/FLNA/PRKG1/FGB/FGA/APOE/ITGA2B/ITGB3/CD9/CEACAM1/COL1A1/COL1A2/THBD	22
GO:0001101	response to acid chemical	31/293	343/18670	3,69E-15	1,69E-13	7,46E-14	SRC/AKT1/EGFR/CASP3/CHUK/MTOR/BAD/PPARG/GSTP1/TNF/RBP4/MMP2/BCL2L1/TYMS/F7/APOB/CD9/ITGA2/CEBPB/VEGFA/CREB1/RXRB/COL1A1/COL5A2/COL6A1/COL1A2/DHFR/KDR/HAMP/IGF2R/LEP	31
GO:0002456	T cell mediated immunity	19/293	106/18670	3,83E-15	1,74E-13	7,66E-14	IL1B/IL6/IL1R1/HMGB1/SLC11A1/CD8A/HLA-E/HLA-C/HLA-G/HLA-A/HLA-B/LILRB1/CEACAM1/IL12B/IL23R/FOXP3/HFE/IL12A/IL4	19
GO:0070665	positive regulation of leukocyte proliferation	21/293	139/18670	4,80E-15	2,16E-13	9,51E-14	AIF1/TLR4/CD320/ADA/IL1B/IL6/BCL2/HAVCR2/HMGB1/HLA-E/IL12B/EFNB1/IGF2/IL23R/FOXP3/TFRC/IGF1/IL12A/IL4/LEP/IL6R	21
GO:0051091	positive regulation of DNA-binding transcription factor activity	27/293	261/18670	8,92E-15	3,97E-13	1,75E-13	NFKB1/AKT1/STAT3/TRAF1/TLR4/ESR2/CHUK/AR/ESR1/IKBKG/HSPA1B/HSPA1A/PPARG/JAK2/IL1B/TNF/IL6/UBE2N/AGT/LTF/VEGFA/CRTC1/TGFB1/EIF2AK2/INS/ROR1/IL10	27
GO:0002697	regulation of immune effector process	35/293	458/18670	9,36E-15	4,11E-13	1,81E-13	TLR4/GATA2/LGALS3/IL1B/TNF/IL6/IL1R1/F2/RBP4/CPB2/MMP12/HAVCR2/HMGB1/C5AR1/HLA-E/HLA-C/HLA-G/HLA-A/HLA-	35

ID	Description	GeneRatio	BgRatio	pvalue	p.adjust	qvalue	geneID	Count
							B/LILRB1/CEACAM1/TGFB1/IL12B/INS/IL23R/FOXP3/IFNG/HFE/TFRC/IL12A/IL4R/IL4/LEP/LTA/IL10	
GO:0034341	response to interferon-gamma	24/293	199/18670	9,40E-15	4,11E-13	1,81E-13	*TP53/AIF1/TLR4/NCAM1/PPARG/JAK2/UBD/SLC11A1/HLA-E/HLA-C/HLA-G/HLA-A/HLA-B/IL12B/IL23R/IFNG/HLA-DQA1/HLA-DRB5/HLA-DRA/HLA-DQB2/HLA-DRB1/HLA-DQB1/HLA-DQA2/NOS2*	24
GO:0002526	acute inflammatory response	25/293	220/18670	1,03E-14	4,47E-13	1,97E-13	*STAT3/PPARG/FN1/GSTP1/ACVR1/IL1B/TNF/IL6/CD163/F2/MBL2/SERPINC1/CPB2/F12/SERPINA1/C5AR1/HLA-E/CEBPB/INS/F3/HAMP/HP/HFE/IL1A/IL6R*	25
GO:0002709	regulation of T cell mediated immunity	16/293	70/18670	1,04E-14	4,47E-13	1,97E-13	*IL1B/IL6/IL1R1/HMGB1/HLA-E/HLA-C/HLA-G/HLA-A/HLA-B/LILRB1/CEACAM1/IL12B/IL23R/FOXP3/HFE/IL12A*	16
GO:0022408	negative regulation of cell-cell adhesion	23/293	181/18670	1,10E-14	4,66E-13	2,05E-13	*AKT1/CASP3/LGALS3/JAK2/BMP2/FGG/PRKG1/HAVCR2/HMGB1/HLA-G/LILRB1/CD9/CEACAM1/CEBPB/VEGFA/TGFB1/CTLA4/FOXP3/HFE/IL1RN/IL4R/PDE5A/IL10*	23
GO:0001938	positive regulation of endothelial cell proliferation	19/293	112/18670	1,10E-14	4,66E-13	2,05E-13	*AKT1/STAT3/GATA2/MTOR/FGFR1/TGFBR1/BMP2/AGTR1/ARNT/HIF1A/ITGB3/HMGB1/VEGFA/F3/KDR/VEGFC/VEGFB/PGF/IL10*	19
GO:0051251	positive regulation of lymphocyte activation	30/293	334/18670	1,24E-14	5,20E-13	2,29E-13	*AIF1/SRC/AKT1/TLR4/BAD/CD320/ADA/IL1B/IL6/BCL2/HAVCR2/HMGB1/HLA-E/HLA-G/LILRB1/TGFB1/IL12B/CTLA4/EFNB1/IGF2/IL23R/FOXP3/IFNG/TFRC/IGF1/IL12A/IL4R/IL4/LEP/IL6R*	30
GO:0044706	multi-multicellular organism process	25/293	222/18670	1,28E-14	5,30E-13	2,34E-13	*AKT1/MTOR/AR/NR2F2/ESR1/IL1B/AGT/PRLR/MMP2/BCL2/TIMP1/MMP9/HAVCR2/ITGA2/PRL/FSHB/MMP7/TGFB1/FBLN1/THBD/PGR/PGF/HFE/PAPPA/LEP*	25
GO:0038034	signal transduction in absence of ligand	16/293	72/18670	1,68E-14	6,85E-13	3,02E-13	*AKT1/CASP3/GATA1/FGFR1/BAD/HSPA1B/HSPA1A/IL1B/TNF/CASP9/BAX/BCL2/BCL2L1/LCN2/IFI6/IL1A*	16
GO:0097192	extrinsic apoptotic signaling pathway in absence of ligand	16/293	72/18670	1,68E-14	6,85E-13	3,02E-13	*AKT1/CASP3/GATA1/FGFR1/BAD/HSPA1B/HSPA1A/IL1B/TNF/CASP9/BAX/BCL2/BCL2L1/LCN2/IFI6/IL1A*	16
GO:0001906	cell killing	22/293	168/18670	2,25E-14	9,10E-13	4,01E-13	*BAD/F2/HRG/MBL2/HAVCR2/LTF/HLA-E/HLA-C/HLA-G/HLA-A/HLA-B/LILRB1/CEACAM1/IL12B/IL23R/IFNG/HAMP/KIR3DL1/IL12A/IL4/LEP/NOS2*	22
GO:0048660	regulation of smooth muscle cell proliferation	22/293	169/18670	2,55E-14	1,02E-12	4,51E-13	*AIF1/AKT1/EGFR/MDM2/MTOR/IGFBP3/PPARG/JAK2/GSTP1/TNF/IL6/AGT/PRKG1/MMP2/MMP9/ITGA2/IL12B/IFNG/IGF1/IL12A/IL10/IL6R*	22
GO:2000379	positive regulation of reactive oxygen species metabolic process	18/293	102/18670	2,74E-14	1,08E-12	4,77E-13	*ACE2/TP53/AIF1/AKT1/TLR4/EGFR/MTOR/JAK2/GSTP1/IL1B/AGTR1/TNF/AGT/F2/TGFB1/INSR/IFNG/LEP*	18
GO:0045637	regulation of myeloid cell differentiation	26/293	251/18670	2,77E-14	1,08E-12	4,77E-13	*STAT3/STAT5B/TLR4/GATA2/MTOR/GATA1/LGALS3/HSPA1B/HSPA1A/TNF/ARNT/HIF1A/INHA/ITGA2B/LTF/LILRB1*	26

ID	Description	GeneRatio	BgRatio	pvalue	p.adjust	qvalue	geneID	Count
							/CEACAM1/CEBPB/CREB1/FSHB/TGFB1/FBN1/IL12B/IL23R/IFNG/IL4	
GO:0032355	response to estradiol	20/293	134/18670	2,77E-14	1,08E-12	4,77E-13	*STAT3/STAT5B/EGFR/CASP3/NR2F2/BAD/ESR1/GSTP1/AGT/ARNT2/CASP9/F7/APOB/ITGA2/TGFB1/COL1A1/TFPI/LEP/MMP15/IL10*	20
GO:0048659	smooth muscle cell proliferation	22/293	171/18670	3,27E-14	1,27E-12	5,59E-13	*AIF1/AKT1/EGFR/MDM2/MTOR/IGFBP3/PPARG/JAK2/GSTP1/TNF/IL6/AGT/PRKG1/MMP2/MMP9/ITGA2/IL12B/IFNG/IGF1/IL12A/IL10/IL6R*	22
GO:0006979	response to oxidative stress	34/293	451/18670	3,45E-14	1,33E-12	5,84E-13	*TP53/AIF1/SRC/AKT1/TRPC6/TLR4/EGFR/MDM2/CASP3/NOS3/CHUK/BAD/HSPA1B/HSPA1A/JAK2/GSTP1/ADA/TNF/IL6/MBL2/ARNT/HIF1A/MMP2/BCL2/APOE/MMP3/MMP9/LCN2/COL1A1/DHFR/INS/HBA1/HP/IL10*	34
GO:0052547	regulation of peptidase activity	34/293	452/18670	3,68E-14	1,40E-12	6,18E-13	*SRC/AKT1/STAT3/MDM2/BAD/PPARG/JAK2/FN1/TNF/TP63/AGT/HRG/SERPINA10/SERPINA6/SERPINC1/CASP9/BAX/FETUB/SERPINA1/TIMP1/TIMP3/SERPINE1/MMP9/HMGB1/LTF/VEGFA/TIMP4/TIMP2/FBLN1/COL6A3/IFI6/TFPI/FASLG/F3*	34
GO:0003018	vascular process in circulatory system	22/293	173/18670	4,18E-14	1,58E-12	6,97E-13	*ACE/ACE2/SRC/AKT1/EGFR/NOS3/ACTA2/ADRA2A/ADRB2/AGTR1/AGT/FGG/PRKG1/HTR1A/FGB/FGA/APOE/CEACAM1/VEGFA/TGFB1/INS/LEP*	22
GO:0002449	lymphocyte mediated immunity	30/293	352/18670	5,02E-14	1,88E-12	8,29E-13	*IL1B/TNF/IL6/IL1R1/MBL2/HAVCR2/HMGB1/SLC11A1/CD8A/HLA-E/HLA-C/HLA-G/HLA-A/HLA-B/LILRB1/CEACAM1/TGFB1/IL12B/IL23R/FOXP3/HFE/TFRC/KIR3DL1/HLA-DQB1/IL12A/IL4R/IL4/LEP/LTA/IL10*	30
GO:0060333	interferon-gamma-mediated signaling pathway	17/293	91/18670	5,41E-14	2,01E-12	8,86E-13	*TP53/NCAM1/PPARG/JAK2/HLA-E/HLA-C/HLA-G/HLA-A/HLA-B/IFNG/HLA-DQA1/HLA-DRB5/HLA-DRA/HLA-DQB2/HLA-DRB1/HLA-DQB1/HLA-DQA2*	17
GO:0043434	response to peptide hormone	33/293	436/18670	7,55E-14	2,79E-12	1,23E-12	*NFKB1/SRC/AKT1/STAT3/STAT5B/MDM2/CHUK/MTOR/IGF1R/PPARG/JAK2/GSTP1/IL1B/AGTR1/AGT/IGFBP1/PRLR/APC/F7/TIMP1/BRIP1/CEACAM1/CREB1/PRL/TGFB1/TIMP4/COL1A1/FBN1/INSR/INS/IGF2/LEP/IL10*	33
GO:0071902	positive regulation of protein serine/threonine kinase activity	29/293	334/18670	8,43E-14	3,09E-12	1,36E-12	*SRC/AKT1/TLR4/EGFR/FGFR1/IKBKG/JAK2/TGFBR1/BMP2/ADRA2A/ADRB2/IL1B/TNF/UBE2N/TP73/C5AR1/LTF/VEGFA/TGFB1/EIF2AK2/EGF/INSR/IGF2/FGF1/FLT1/IFNG/IGF1/MAPRE3/PDE5A*	29
GO:0050866	negative regulation of cell activation	23/293	199/18670	8,69E-14	3,16E-12	1,39E-12	*CASP3/NOS3/LGALS3/FGG/F2/PRKG1/INHA/APOE/HAVCR2/HMGB1/HLA-G/LILRB1/CD9/CEACAM1/CEBPB/TGFB1/THBD/CTLA4/FOXP3/HFE/IL4R/PDE5A/IL10*	23
GO:0046879	hormone secretion	28/293	312/18670	1,02E-13	3,67E-12	1,62E-12	*EGFR/FGFR1/BAD/JAK2/ADRA2A/IL1B/AGTR1/TNF/IL6/AGT/REN/FGG/RBP4/HIF1A/HNF4A/HTR1A/INHA/FGB/FGA/CREB1/CFTR/INS/IFNG/HFE/HNF1A/IL1RN/LEP/NOS2*	28

ID	Description	GeneRatio	BgRatio	pvalue	p.adjust	qvalue	geneID	Count
GO:0042102	positive regulation of T cell proliferation	17/293	95/18670	1,14E-13	4,08E-12	1,80E-12	*AIF1/IL1B/IL6/HAVCR2/HMGB1/HLA-E/IL12B/EFNB1/IGF2/IL23R/FOXP3/TFRC/IGF1/IL12A/IL4/LEP/IL6R*	17
GO:0002687	positive regulation of leukocyte migration	19/293	128/18670	1,38E-13	4,90E-12	2,16E-12	*AIF1/LGALS3/TNF/IL6/IL1R1/F7/ITGA2B/SERPINE1/HMGB1/C5AR1/ITGA2/VEGFA/TGFB1/CXCL8/VEGFC/VEGFB/PGF/IL12A/IL6R*	19
GO:0045834	positive regulation of lipid metabolic process	20/293	146/18670	1,48E-13	5,22E-12	2,30E-12	*SRC/AKT1/MTOR/TNFRSF1A/PPARG/IL1B/AGTR1/TNF/AGT/F2/APOE/CREB1/CGA/FSHB/TGFB1/INS/FGF1/FGFR3/FLT1/IFNG*	20
GO:0007548	sex differentiation	26/293	270/18670	1,56E-13	5,46E-12	2,40E-12	*SRC/CASP3/NOS3/AR/GATA1/ESR1/TGFBR1/TP63/REN/RBP4/HNF4A/FLNA/BAX/BCL2/BCL2L1/INHA/BRIP1/BRCA2/CEBPB/VEGFA/LHB/FSHB/FSHR/INSR/PGR/LEP*	26
GO:0070482	response to oxygen levels	31/293	394/18670	1,61E-13	5,59E-12	2,46E-12	*TP53/SRC/AKT1/TRPC6/VHL/MDM2/CASP3/MTOR/BAD/PPARG/BMP2/ADA/ARNT/ARNT2/HIF1A/MMP2/BCL2/F7/PLAT/BRIP1/ITGA2/VEGFA/CREB1/TGFB1/COL1A1/VEGFC/VEGFB/PGF/LEP/LTA/NOS2*	31
GO:1903708	positive regulation of hemopoiesis	22/293	185/18670	1,70E-13	5,85E-12	2,58E-12	*STAT3/STAT5B/GATA2/GATA1/BAD/HSPA1B/HSPA1A/ADA/TNF/ARNT/HIF1A/HMGB1/HLA-G/CREB1/TGFB1/IL12B/IL23R/FOXP3/IFNG/IL12A/IL4R/IL4*	22
GO:0042730	fibrinolysis	11/293	28/18670	2,00E-13	6,84E-12	3,02E-12	*TMPRSS6/FGG/F2/HRG/CPB2/F12/FGB/FGA/PLAT/SERPINE1/THBD*	11
GO:0052548	regulation of endopeptidase activity	32/293	425/18670	2,13E-13	7,25E-12	3,19E-12	*SRC/AKT1/STAT3/MDM2/BAD/PPARG/JAK2/TNF/TP63/AGT/HRG/SERPINA10/SERPINA6/SERPINC1/CASP9/BAX/FETUB/SERPINA1/TIMP1/TIMP3/SERPINE1/MMP9/HMGB1/LTF/VEGFA/TIMP4/TIMP2/COL6A3/IFI6/TFPI/FASLG/F3*	32
GO:0009914	hormone transport	28/293	322/18670	2,23E-13	7,52E-12	3,31E-12	*EGFR/FGFR1/BAD/JAK2/ADRA2A/IL1B/AGTR1/TNF/IL6/AGT/REN/FGG/RBP4/HIF1A/HNF4A/HTR1A/INHA/FGB/FGA/CREB1/CFTR/INS/IFNG/HFE/HNF1A/IL1RN/LEP/NOS2*	28
GO:0046660	female sex differentiation	18/293	115/18670	2,39E-13	8,02E-12	3,53E-12	*SRC/CASP3/NOS3/ESR1/TP63/RBP4/BAX/BCL2/BCL2L1/INHA/BRCA2/CEBPB/VEGFA/FSHB/FSHR/INSR/PGR/LEP*	18
GO:0002705	positive regulation of leukocyte mediated immunity	19/293	133/18670	2,81E-13	9,36E-12	4,13E-12	*GATA2/IL1B/TNF/IL6/IL1R1/HLA-E/HLA-C/HLA-G/HLA-A/HLA-B/TGFB1/IL12B/IL23R/FOXP3/TFRC/IL12A/IL4R/IL4/LTA*	19
GO:0000302	response to reactive oxygen species	24/293	232/18670	2,91E-13	9,61E-12	4,24E-12	*SRC/AKT1/TRPC6/EGFR/MDM2/CASP3/NOS3/CHUK/BAD/GSTP1/ADA/TNF/IL6/MMP2/BCL2/APOE/MMP3/MMP9/LCN2/COL1A1/DHFR/HBA1/HP/IL10*	24
GO:0032649	regulation of interferon-gamma production	17/293	101/18670	3,27E-13	1,07E-11	4,73E-12	*TLR4/IL1B/TNF/IL1R1/INHA/HAVCR2/HMGB1/SLC11A1/CD14/LILRB1/IL12B/IL23R/FOXP3/IL12A/LTA/IL10/IL6R*	17
GO:0051090	regulation of DNA-binding transcription factor activity	32/293	432/18670	3,33E-13	1,09E-11	4,78E-12	*PIM1/NFKB1/AKT1/STAT3/TRAF1/TLR4/ESR2/CHUK/AR/ESR1/IKBKG/HSPA1B/HSPA1A/PPARG/JAK2/IL1B/TNF/IL*	32

ID	Description	GeneRatio	BgRatio	pvalue	p.adjust	qvalue	geneID	Count
							6/UBE2N/AGT/SGK1/FLNA/HAVCR2/LTF/VEGFA/CRTC1/TGFB1/EIF2AK2/INS/FOXP3/ROR1/IL10	
GO:0060326	cell chemotaxis	27/293	304/18670	3,68E-13	1,19E-11	5,25E-12	*AIF1/FGFR1/LGALS3/GSTP1/IL1B/AGTR1/CCR5/IL6/HRG/IL16/F7/SERPINE1/HMGB1/C5AR1/VEGFA/CXCL8/CXCR1/FGF1/KDR/VEGFC/FLT1/VEGFB/PGF/IL12A/IL1RN/IL10/IL6R*	27
GO:0050921	positive regulation of chemotaxis	19/293	135/18670	3,71E-13	1,19E-11	5,25E-12	*AIF1/FGFR1/IL6/IL16/F7/SERPINE1/HMGB1/C5AR1/ITGA2/VEGFA/TGFB1/CXCL8/F3/KDR/VEGFC/VEGFB/PGF/IL12A/IL6R*	19
GO:0045765	regulation of angiogenesis	30/293	383/18670	4,56E-13	1,46E-11	6,41E-12	*STAT3/NOS3/GATA2/PPARG/IL1B/AGTR1/IL6/AGT/HRG/HIF1A/SERPINE1/BRCA1/C5AR1/HLA-G/CEACAM1/VEGFA/PRL/SULF1/CXCL8/FASLG/F3/FGF1/KDR/VEGFC/FLT1/VEGFB/PGF/IL1A/LEP/IL10*	30
GO:0002699	positive regulation of immune effector process	23/293	216/18670	5,02E-13	1,59E-11	7,02E-12	*TLR4/GATA2/IL1B/TNF/IL6/IL1R1/RBP4/HLA-E/HLA-C/HLA-G/HLA-A/HLA-B/LILRB1/TGFB1/IL12B/IL23R/FOXP3/IFNG/TFRC/IL12A/IL4R/IL4/LTA*	23
GO:1901653	cellular response to peptide	30/293	385/18670	5,22E-13	1,64E-11	7,24E-12	*NFKB1/TP53/SRC/AKT1/STAT3/STAT5B/TLR4/MDM2/IGF1R/PPARG/JAK2/GSTP1/ADRB2/IL1B/AGTR1/AGT/IGFBP1/PRLR/APC/BRIP1/CEACAM1/CREB1/PRL/TGFB1/FBN1/INSR/INS/IGF2/IGF1/LEP*	30
GO:0001666	response to hypoxia	29/293	359/18670	5,28E-13	1,65E-11	7,28E-12	*TP53/SRC/AKT1/TRPC6/VHL/MDM2/CASP3/MTOR/BAD/BMP2/ADA/ARNT/ARNT2/HIF1A/MMP2/BCL2/F7/PLAT/BRIP1/ITGA2/VEGFA/CREB1/TGFB1/VEGFC/VEGFB/PGF/LEP/LTA/NOS2*	29
GO:0008406	gonad development	23/293	217/18670	5,54E-13	1,72E-11	7,59E-12	*SRC/CASP3/NOS3/AR/GATA1/ESR1/TGFBR1/REN/FLNA/BAX/BCL2/BCL2L1/INHA/BRIP1/BRCA2/CEBPB/VEGFA/LHB/FSHB/FSHR/INSR/PGR/LEP*	23
GO:0002685	regulation of leukocyte migration	22/293	196/18670	5,59E-13	1,73E-11	7,61E-12	*AIF1/AKT1/LGALS3/ADA/TNF/IL6/IL1R1/F7/ITGA2B/SERPINE1/HMGB1/C5AR1/CD9/ITGA2/VEGFA/TGFB1/CXCL8/VEGFC/VEGFB/PGF/IL12A/IL6R*	22
GO:0090130	tissue migration	29/293	360/18670	5,66E-13	1,74E-11	7,66E-12	*ACTA1/SRC/AKT1/NOS3/GATA2/MTOR/NR2F2/FGFR1/PPARG/ACTA2/TGFBR1/AGT/HRG/HIF1A/APOE/ITGB3/MMP9/HMGB1/ITGA2/CEACAM1/VEGFA/TGFB1/EPHB4/FGF1/FGF7/KDR/VEGFC/IFNG/IL4*	29
GO:0050663	cytokine secretion	24/293	240/18670	6,12E-13	1,87E-11	8,22E-12	*AIF1/SRC/TLR4/CHUK/FN1/IL1B/TNF/AGT/MMP12/ITGB6/HAVCR2/HMGB1/CD14/LILRB1/CHIA/INS/FOXP3/IFNG/IL1A/IL4R/LEP/NOS2/TLR1/IL10*	24
GO:0002708	positive regulation of lymphocyte mediated immunity	17/293	105/18670	6,33E-13	1,92E-11	8,45E-12	*IL1B/TNF/IL6/IL1R1/HLA-E/HLA-C/HLA-G/HLA-A/HLA-B/TGFB1/IL12B/IL23R/FOXP3/TFRC/IL12A/IL4/LTA*	17
GO:0045861	negative regulation of proteolysis	29/293	363/18670	6,98E-13	2,10E-11	9,25E-12	*TP53/SRC/AKT1/MDM2/AGT/F2/HRG/SERPINA10/SERPINA6/SERPINC1/CPB2/FETUB/SERPINA1/PLAT/TIMP1/TIMP3/SERPINE1/MMP9/BAG6/LTF/VEGFA/TIMP4/TIMP2/COL6A3/IFI6/INS/TFPI/HFE/IL10*	29

ID	Description	GeneRatio	BgRatio	pvalue	p.adjust	qvalue	geneID	Count
GO:0051043	regulation of membrane protein ectodomain proteolysis	10/293	23/18670	7,43E-13	2,22E-11	9,79E-12	*ADRA2A/IL1B/TNF/APOE/TIMP1/TIMP3/TIMP4/TIMP2/IFNG/IL10*	10
GO:0046883	regulation of hormone secretion	25/293	266/18670	8,06E-13	2,40E-11	1,06E-11	*EGFR/FGFR1/BAD/JAK2/ADRA2A/IL1B/AGTR1/TNF/AGT/REN/FGG/RBP4/HIF1A/HNF4A/HTR1A/INHA/FGB/FGA/CREB1/CFTR/INS/IFNG/HFE/LEP/NOS2*	25
GO:0071346	cellular response to interferon-gamma	21/293	180/18670	8,95E-13	2,64E-11	1,16E-11	*TP53/AIF1/TLR4/NCAM1/PPARG/JAK2/HLA-E/HLA-C/HLA-G/HLA-A/HLA-B/IL12B/IFNG/HLA-DQA1/HLA-DRB5/HLA-DRA/HLA-DQB2/HLA-DRB1/HLA-DQB1/HLA-DQA2/NOS2*	21
GO:0045137	development of primary sexual characteristics	23/293	223/18670	9,87E-13	2,89E-11	1,28E-11	*SRC/CASP3/NOS3/AR/GATA1/ESR1/TGFBR1/REN/FLNA/BAX/BCL2/BCL2L1/INHA/BRIP1/BRCA2/CEBPB/VEGFA/LHB/FSHB/FSHR/INSR/PGR/LEP*	23
GO:1901342	regulation of vasculature development	31/293	422/18670	9,95E-13	2,89E-11	1,28E-11	*STAT3/NOS3/GATA2/PPARG/IL1B/AGTR1/IL6/AGT/HRG/HIF1A/SERPINE1/BRCA1/C5AR1/HLA-G/CEACAM1/VEGFA/PRL/SULF1/CXCL8/FASLG/F3/FGF1/KDR/VEGFC/FLT1/VEGFB/PGF/IL1A/LEP/IL10/IL6R*	31
GO:0002285	lymphocyte activation involved in immune response	21/293	181/18670	9,99E-13	2,89E-11	1,28E-11	*TP53/STAT3/TLR4/MTOR/LGALS3/ADA/IL6/HAVCR2/HMGB1/SLC11A1/LILRB1/CEACAM1/TGFB1/IL12B/IL23R/FOXP3/IFNG/TFRC/IL4R/IL4/IL10*	21
GO:0043491	protein kinase B signaling	25/293	269/18670	1,04E-12	2,99E-11	1,32E-11	*SRC/AKT1/EGFR/MTOR/FGFR1/ESR1/IGF1R/TGFBR1/IL1B/TNF/F7/HLA-G/TGFB1/EGF/INSR/INS/IGF2/F10/F3/FGF1/FGFR3/FGF7/KDR/IGF1/LEP*	25
GO:0036293	response to decreased oxygen levels	29/293	370/18670	1,13E-12	3,22E-11	1,42E-11	*TP53/SRC/AKT1/TRPC6/VHL/MDM2/CASP3/MTOR/BAD/BMP2/ADA/ARNT/ARNT2/HIF1A/MMP2/BCL2/F7/PLAT/BRIP1/ITGA2/VEGFA/CREB1/TGFB1/VEGFC/VEGFB/PGF/LEP/LTA/NOS2*	29
GO:0001818	negative regulation of cytokine production	26/293	296/18670	1,32E-12	3,76E-11	1,66E-11	*NFKB1/TLR4/FGFR1/FN1/GSTP1/TNF/IL6/F2/INHA/HAVCR2/HMGB1/SLC11A1/LTF/LILRB1/CEACAM1/TGFB1/IL12B/DICER1/IL23R/FOXP3/IFNG/HFE/IGF1/IL12A/IL4/IL10*	26
GO:1902105	regulation of leukocyte differentiation	25/293	272/18670	1,33E-12	3,76E-11	1,66E-11	*TLR4/GATA2/MTOR/BAD/ADA/TNF/INHA/HMGB1/LTF/HLA-G/LILRB1/CEACAM1/CEBPB/CREB1/FSHB/TGFB1/FBN1/IL12B/CTLA4/IL23R/FOXP3/IFNG/IL12A/IL4R/IL4*	25
GO:0042088	T-helper 1 type immune response	12/293	43/18670	1,77E-12	4,98E-11	2,20E-11	*TLR4/MTOR/IL1B/IL1R1/HAVCR2/HMGB1/SLC11A1/IL12B/IL23R/IL12A/IL4R/IL6R*	12
GO:0008585	female gonad development	16/293	96/18670	1,93E-12	5,39E-11	2,38E-11	*SRC/CASP3/NOS3/ESR1/BAX/BCL2/BCL2L1/INHA/BRCA2/CEBPB/VEGFA/FSHB/FSHR/INSR/PGR/LEP*	16
GO:0050927	positive regulation of positive chemotaxis	10/293	25/18670	2,06E-12	5,74E-11	2,53E-11	*IL16/F7/ITGA2/VEGFA/CXCL8/F3/KDR/VEGFC/VEGFB/PGF*	10

ID	Description	GeneRatio	BgRatio	pvalue	p.adjust	qvalue	geneID	Count
GO:0046677	response to antibiotic	27/293	327/18670	2,08E-12	5,76E-11	2,54E-11	*TP53/SRC/STAT3/TRPC6/MDM2/CASP3/CHUK/BAD/JAK2/GSTP1/ADA/IL6/RBP4/AHR/CASP9/BCL2/BCL2L1/TYMS/CD14/LCN2/CFTR/COL1A1/HAMP/HBA1/HP/LEP/IL10*	27
GO:0034599	cellular response to oxidative stress	26/293	302/18670	2,10E-12	5,76E-11	2,54E-11	*TP53/AIF1/SRC/AKT1/TRPC6/TLR4/EGFR/MDM2/NOS3/CHUK/HSPA1B/HSPA1A/JAK2/GSTP1/TNF/IL6/ARNT/HIF1A/MMP2/BCL2/MMP3/MMP9/LCN2/DHFR/INS/IL10*	26
GO:0032609	interferon-gamma production	17/293	113/18670	2,18E-12	5,96E-11	2,63E-11	*TLR4/IL1B/TNF/IL1R1/INHA/HAVCR2/HMGB1/SLC11A1/CD14/LILRB1/IL12B/IL23R/FOXP3/IL12A/LTA/IL10/IL6R*	17
GO:0030316	osteoclast differentiation	16/293	97/18670	2,28E-12	6,19E-11	2,73E-11	*SRC/TLR4/MTOR/TNF/LTF/LILRB1/CEBPB/CREB1/FSHB/TGFB1/FBN1/IL12B/IL23R/IFNG/TFRC/IL4*	16
GO:0042035	regulation of cytokine biosynthetic process	17/293	114/18670	2,53E-12	6,81E-11	3,00E-11	*NFKB1/STAT3/TLR4/JAK2/IL1B/TNF/IL6/INHA/LILRB1/CEBPB/IL12B/FOXP3/IFNG/IL1A/IL4/TLR1/IL10*	17
GO:0030522	intracellular receptor signaling pathway	25/293	280/18670	2,54E-12	6,81E-11	3,00E-11	*PIM1/SRC/STAT3/THRB/TLR4/NR3C1/ESR2/AR/NR2F2/ESR1/IKBKG/HSPA1B/HSPA1A/PPARG/JAK2/RAN/TP63/UBE2N/AHR/ARNT/BRCA1/CNOT1/RXRB/PGR/LEP*	25
GO:0001655	urogenital system development	27/293	330/18670	2,58E-12	6,90E-11	3,04E-11	*ACE/GATA2/AR/FGFR1/ESR1/ACTA2/TGFBR1/BMP2/AGTR1/TP63/TP73/AGT/REN/RBP4/CASP9/BAX/BCL2/ITGB4/MMP9/BAG6/VEGFA/TGFB1/FBN1/SULF1/FGF1/PGF/IL6R*	27
GO:0019216	regulation of lipid metabolic process	30/293	410/18670	2,61E-12	6,94E-11	3,06E-11	*NFKB1/SRC/AKT1/MTOR/TNFRSF1A/PPARG/BMP2/ADRA2A/RAN/IL1B/AGTR1/TNF/AGT/F2/HNF4A/APOB/APOE/BRCA1/CEACAM1/CREB1/CGA/FSHB/TGFB1/DHCR7/INS/FGF1/FGFR3/FLT1/IFNG/LEP*	30
GO:0031341	regulation of cell killing	16/293	98/18670	2,68E-12	7,09E-11	3,12E-11	*BAD/HAVCR2/HLA-E/HLA-C/HLA-G/HLA-A/HLA-B/LILRB1/CEACAM1/IL12B/IL23R/IFNG/IL12A/IL4/LEP/NOS2*	16
GO:0008202	steroid metabolic process	27/293	331/18670	2,77E-12	7,28E-11	3,21E-11	*NFKB1/FGFR1/ESR1/BMP2/RAN/IL1B/AGTR1/TNF/AGT/SERPINA6/LEPR/PRLR/APOB/APOE/NSDHL/CEBPA/CFTR/CGA/LHB/FSHB/CNBP/DHCR7/EBP/FGF1/IFNG/IL4/LEP*	27
GO:0032963	collagen metabolic process	17/293	115/18670	2,93E-12	7,64E-11	3,37E-11	*TMPRSS6/PPARG/IL6/F2/HIF1A/MMP2/MMP12/MMP3/MMP9/ITGA2/MMP1/MMP7/TGFB1/COL1A1/COL5A1/COL1A2/MMP15*	17
GO:0050926	regulation of positive chemotaxis	10/293	26/18670	3,31E-12	8,59E-11	3,79E-11	*IL16/F7/ITGA2/VEGFA/CXCL8/F3/KDR/VEGFC/VEGFB/PGF*	10
GO:0062013	positive regulation of small molecule metabolic process	19/293	153/18670	3,66E-12	9,44E-11	4,16E-11	*NFKB1/SRC/AKT1/STAT3/NOS3/PPARG/IL1B/TNF/ARNT/HIF1A/EGF/INSR/INS/IGF2/FGF1/IFNG/IGF1/IL4/NOS2*	19
GO:0002761	regulation of myeloid leukocyte differentiation	17/293	117/18670	3,90E-12	1,00E-10	4,42E-11	*TLR4/GATA2/MTOR/TNF/INHA/LTF/LILRB1/CEACAM1/CEBPB/CREB1/FSHB/TGFB1/FBN1/IL12B/IL23R/IFNG/IL4*	17
GO:0046545	development of primary female sexual characteristics	16/293	101/18670	4,34E-12	1,11E-10	4,87E-11	*SRC/CASP3/NOS3/ESR1/BAX/BCL2/BCL2L1/INHA/BRCA2/CEBPB/VEGFA/FSHB/FSHR/INSR/PGR/LEP*	16

ID	Description	GeneRatio	BgRatio	pvalue	p.adjust	qvalue	geneID	Count
GO:0042531	positive regulation of tyrosine phosphorylation of STAT protein	14/293	71/18670	4,35E-12	1,11E-10	4,87E-11	*STAT3/TNFRSF1A/JAK2/TNF/IL6/IL12B/FGFR3/IL23R/IFNG/IGF1/IL12A/IL4/LEP/IL6R*	14
GO:0050920	regulation of chemotaxis	22/293	217/18670	4,42E-12	1,12E-10	4,92E-11	*AIF1/FGFR1/GSTP1/IL6/HRG/IL16/F7/SERPINE1/HMGB1/C5AR1/ITGA2/VEGFA/TGFB1/CXCL8/F3/FGF1/KDR/VEGFC/VEGFB/PGF/IL12A/IL6R*	22
GO:0008625	extrinsic apoptotic signaling pathway via death domain receptors	15/293	86/18670	4,84E-12	1,22E-10	5,36E-11	*NOS3/BAD/TNFRSF1A/LGALS3/TNF/FGG/BAX/BCL2/BCL2L1/FGB/FGA/TIMP3/SERPINE1/BRCA1/FASLG*	15
GO:0002367	cytokine production involved in immune response	16/293	102/18670	5,08E-12	1,27E-10	5,59E-11	*TLR4/IL1B/TNF/IL6/IL1R1/SLC11A1/HLA-E/HLA-G/LILRB1/TGFB1/IL12B/FOXP3/HFE/IL12A/IL4/IL10*	16
GO:0051897	positive regulation of protein kinase B signaling	20/293	176/18670	5,19E-12	1,29E-10	5,69E-11	*SRC/EGFR/MTOR/FGFR1/ESR1/IGF1R/TGFBR1/TNF/F7/TGFB1/EGF/INSR/INS/IGF2/F10/F3/FGF1/FGFR3/FGF7/LEP*	20
GO:0033619	membrane protein proteolysis	13/293	59/18670	5,70E-12	1,41E-10	6,21E-11	*TMPRSS6/NFKB1/ADRA2A/IL1B/TNF/APOE/TIMP1/TIMP3/TGFB1/TIMP4/TIMP2/IFNG/IL10*	13
GO:0010632	regulation of epithelial cell migration	25/293	291/18670	5,95E-12	1,46E-10	6,42E-11	*SRC/AKT1/NOS3/GATA2/MTOR/NR2F2/FGFR1/PPARG/AGT/HRG/HIF1A/APOE/ITGB3/MMP9/HMGB1/ITGA2/CEACAM1/VEGFA/TGFB1/FGF1/FGF7/KDR/VEGFC/IFNG/IL4*	25
GO:0045088	regulation of innate immune response	31/293	452/18670	5,95E-12	1,46E-10	6,42E-11	*NFKB1/SRC/TLR4/CHUK/ESR1/IKBKG/HSPA1B/HSPA1A/PPARG/JAK2/UBE2N/FGG/FGB/FGA/APOB/APOE/MMP12/HAVCR2/HMGB1/CD14/LTF/HLA-E/HLA-G/LILRB1/CEACAM1/IL12B/INS/IFNG/IL12A/LEP/TLR1*	31
GO:0070371	ERK1 and ERK2 cascade	26/293	317/18670	6,33E-12	1,54E-10	6,79E-11	*SRC/TLR4/EGFR/FN1/GSTP1/BMP2/IL1B/TNF/AGT/FGG/FGB/FGA/APOE/TIMP3/HAVCR2/HMGB1/C5AR1/CEACAM1/FSHR/TGFB1/FBLN1/EGF/FGFR3/KDR/FLT1/IGF1*	26
GO:0031100	animal organ regeneration	14/293	73/18670	6,50E-12	1,58E-10	6,94E-11	*EGFR/PPARG/GSTP1/TYMS/CPB2/F7/C5AR1/CEBPB/TGFB1/PGF/HAMP/HFE/IGF2R/IL10*	14
GO:0034103	regulation of tissue remodeling	15/293	88/18670	6,85E-12	1,65E-10	7,27E-11	*TP53/SRC/EGFR/IL6/AGT/HRG/LEPR/BAX/ITGB3/CEACAM1/FSHB/TGFB1/IL12B/TFRC/LEP*	15
GO:0002711	positive regulation of T cell mediated immunity	12/293	48/18670	7,51E-12	1,80E-10	7,93E-11	*IL1B/IL6/IL1R1/HLA-E/HLA-C/HLA-G/HLA-A/HLA-B/IL12B/IL23R/FOXP3/IL12A*	12
GO:0048872	homeostasis of number of cells	23/293	246/18670	7,65E-12	1,83E-10	8,04E-11	*AKT1/STAT3/STAT5B/CASP3/NOS3/GATA2/GATA1/HSPA1B/HSPA1A/JAK2/ADA/IL6/ARNT/HIF1A/BAX/BCL2/INHA/HMGB1/VEGFA/TGFB1/FOXP3/SLC4A1/TSC22D3*	23
GO:0071375	cellular response to peptide hormone stimulus	26/293	321/18670	8,41E-12	2,00E-10	8,80E-11	*NFKB1/SRC/AKT1/STAT3/STAT5B/MDM2/IGF1R/PPARG/JAK2/GSTP1/IL1B/AGTR1/AGT/IGFBP1/PRLR/APC/BRIP1/CEACAM1/CREB1/PRL/TGFB1/FBN1/INSR/INS/IGF2/LEP*	26

ID	Description	GeneRatio	BgRatio	pvalue	p.adjust	qvalue	geneID	Count
GO:0042089	cytokine biosynthetic process	17/293	123/18670	8,93E-12	2,11E-10	9,30E-11	*NFKB1/STAT3/TLR4/JAK2/IL1B/TNF/IL6/INHA/LILRB1/CEBPB/IL12B/FOXP3/IFNG/IL1A/IL4/TLR1/IL10*	17
GO:0001667	ameboidal-type cell migration	31/293	461/18670	9,88E-12	2,32E-10	1,02E-10	*SRC/AKT1/NOS3/GATA2/MTOR/NR2F2/FGFR1/PPARG/FN1/ACVR1/TGFBR1/AGT/HRG/HIF1A/APOE/ITGB3/TIMP1/MMP9/HMGB1/ITGA2/CEACAM1/VEGFA/TGFB1/EFNB1/EPHB4/FGF1/FGF7/KDR/VEGFC/IFNG/IL4*	31
GO:0042107	cytokine metabolic process	17/293	124/18670	1,02E-11	2,39E-10	1,05E-10	*NFKB1/STAT3/TLR4/JAK2/IL1B/TNF/IL6/INHA/LILRB1/CEBPB/IL12B/FOXP3/IFNG/IL1A/IL4/TLR1/IL10*	17
GO:0030879	mammary gland development	18/293	143/18670	1,09E-11	2,49E-10	1,10E-10	*SRC/AKT1/AR/ESR1/JAK2/SERPINC1/PRLR/HIF1A/BAX/BRCA2/ITGA2/CEBPB/VEGFA/CREB1/PRL/TGFB1/EGF/PGR*	18
GO:0035296	regulation of tube diameter	18/293	143/18670	1,09E-11	2,49E-10	1,10E-10	*ACE/ACE2/AKT1/EGFR/NOS3/ACTA2/ADRA2A/ADRB2/AGTR1/AGT/FGG/PRKG1/HTR1A/FGB/FGA/APOE/INS/LEP*	18
GO:0050880	regulation of blood vessel size	18/293	143/18670	1,09E-11	2,49E-10	1,10E-10	*ACE/ACE2/AKT1/EGFR/NOS3/ACTA2/ADRA2A/ADRB2/AGTR1/AGT/FGG/PRKG1/HTR1A/FGB/FGA/APOE/INS/LEP*	18
GO:0097746	regulation of blood vessel diameter	18/293	143/18670	1,09E-11	2,49E-10	1,10E-10	*ACE/ACE2/AKT1/EGFR/NOS3/ACTA2/ADRA2A/ADRB2/AGTR1/AGT/FGG/PRKG1/HTR1A/FGB/FGA/APOE/INS/LEP*	18
GO:0010631	epithelial cell migration	27/293	351/18670	1,09E-11	2,49E-10	1,10E-10	*SRC/AKT1/NOS3/GATA2/MTOR/NR2F2/FGFR1/PPARG/TGFBR1/AGT/HRG/HIF1A/APOE/ITGB3/MMP9/HMGB1/ITGA2/CEACAM1/VEGFA/TGFB1/EPHB4/FGF1/FGF7/KDR/VEGFC/IFNG/IL4*	27
GO:0002683	negative regulation of immune system process	31/293	463/18670	1,10E-11	2,51E-10	1,10E-10	*AKT1/TLR4/CASP3/GATA2/LGALS3/PPARG/ADA/TNF/INHA/MMP12/HAVCR2/HMGB1/CD14/LTF/HLA-E/HLA-G/LILRB1/CEACAM1/CEBPB/TGFB1/FBN1/IL12B/CTLA4/INS/FOXP3/HFE/IL4R/IL4/TSC22D3/PDE5A/IL10*	31
GO:0035150	regulation of tube size	18/293	144/18670	1,22E-11	2,77E-10	1,22E-10	*ACE/ACE2/AKT1/EGFR/NOS3/ACTA2/ADRA2A/ADRB2/AGTR1/AGT/FGG/PRKG1/HTR1A/FGB/FGA/APOE/INS/LEP*	18
GO:0006109	regulation of carbohydrate metabolic process	21/293	206/18670	1,24E-11	2,78E-10	1,23E-10	*NFKB1/TP53/SRC/AKT1/STAT3/MTOR/BAD/IGFBP3/ARNT/LEPR/HIF1A/LCMT1/IGFBP4/TGFB1/EGF/INSR/INS/IGF2/IFNG/IGF1/LEP*	21
GO:0090132	epithelium migration	27/293	354/18670	1,33E-11	2,97E-10	1,31E-10	*SRC/AKT1/NOS3/GATA2/MTOR/NR2F2/FGFR1/PPARG/TGFBR1/AGT/HRG/HIF1A/APOE/ITGB3/MMP9/HMGB1/ITGA2/CEACAM1/VEGFA/TGFB1/EPHB4/FGF1/FGF7/KDR/VEGFC/IFNG/IL4*	27
GO:1904018	positive regulation of vasculature development	22/293	230/18670	1,41E-11	3,15E-10	1,39E-10	*STAT3/NOS3/GATA2/IL1B/AGTR1/HIF1A/SERPINE1/BRCA1/C5AR1/CEACAM1/VEGFA/CXCL8/F3/FGF1/KDR/VEGFC/FLT1/VEGFB/PGF/IL1A/IL10/IL6R*	22
GO:0045670	regulation of osteoclast differentiation	13/293	64/18670	1,72E-11	3,83E-10	1,69E-10	*TLR4/MTOR/TNF/LTF/LILRB1/CEBPB/CREB1/FSHB/FBN1/IL12B/IL23R/IFNG/IL4*	13

ID	Description	GeneRatio	BgRatio	pvalue	p.adjust	qvalue	geneID	Count
GO:1904019	epithelial cell apoptotic process	16/293	111/18670	1,92E-11	4,25E-10	1,87E-10	*GATA2/MTOR/BAD/JAK2/TNF/IL6/FGG/BCL2L1/FGB/FGA/SERPINE1/HLA-G/FASLG/KDR/IL4/IL10*	16
GO:0001558	regulation of cell growth	29/293	416/18670	2,01E-11	4,43E-10	1,95E-10	*TP53/AKT1/EGFR/ESR2/MTOR/HSPA1B/HSPA1A/IGFBP3/PPARG/FN1/L1CAM/TGFBR1/AGTR1/AGT/F2/HRG/IGFBP1/HNF4A/SGK1/BCL2/APOE/MAP2/IGFBP4/CEACAM1/VEGFA/TGFB1/INS/HAMP/IGF1*	29
GO:0043406	positive regulation of MAP kinase activity	23/293	258/18670	2,03E-11	4,45E-10	1,96E-10	*SRC/TLR4/EGFR/FGFR1/IKBKG/JAK2/TGFBR1/BMP2/ADRA2A/IL1B/TNF/UBE2N/TP73/C5AR1/VEGFA/TGFB1/EIF2AK2/EGF/INSR/FGF1/FLT1/IGF1/PDE5A*	23
GO:0009895	negative regulation of catabolic process	25/293	308/18670	2,06E-11	4,48E-10	1,97E-10	*TP53/AKT1/STAT3/EGFR/MTOR/ADRA2A/IL1B/TNF/IL10RA/LEPR/FLNA/BCL2/TIMP1/TIMP3/BAG6/SLC11A1/TIMP4/TIMP2/INS/FMR1/HP/HFE/LEP/NOS2/IL10*	25
GO:0031331	positive regulation of cellular catabolic process	27/293	361/18670	2,08E-11	4,51E-10	1,99E-10	*AKT1/MDM2/BAD/IKBKG/HSPA1B/HSPA1A/ADRA2A/ADRB2/IL1B/TNF/IL6/ARNT/HIF1A/BAX/APOE/BAG6/HMGB1/CEBPA/CNOT1/EGF/INSR/INS/KDR/FMR1/IFNG/IGF1/IL4*	27
GO:0051092	positive regulation of NF-kappaB transcription factor activity	18/293	149/18670	2,19E-11	4,73E-10	2,08E-10	*NFKB1/STAT3/TRAF1/TLR4/CHUK/AR/IKBKG/HSPA1B/HSPA1A/IL1B/TNF/UBE2N/AGT/LTF/TGFB1/EIF2AK2/INS/ROR1*	18
GO:0043405	regulation of MAP kinase activity	26/293	337/18670	2,50E-11	5,38E-10	2,37E-10	*SRC/TLR4/EGFR/FGFR1/IKBKG/IGF1R/JAK2/GSTP1/TGFBR1/BMP2/ADRA2A/IL1B/TNF/UBE2N/TP73/APOE/C5AR1/VEGFA/TGFB1/EIF2AK2/EGF/INSR/FGF1/FLT1/IGF1/PDE5A*	26
GO:0043200	response to amino acid	16/293	113/18670	2,54E-11	5,43E-10	2,39E-10	*EGFR/CASP3/CHUK/MTOR/BAD/GSTP1/TNF/MMP2/BCL2L1/F7/CEBPB/CREB1/COL1A1/COL5A2/COL6A1/COL1A2*	16
GO:0010543	regulation of platelet activation	10/293	31/18670	2,58E-11	5,49E-10	2,42E-10	*TLR4/NOS3/FGG/F2/HRG/PRKG1/APOE/CD9/CEACAM1/THBD*	10
GO:0010634	positive regulation of epithelial cell migration	19/293	171/18670	2,67E-11	5,65E-10	2,49E-10	*SRC/AKT1/NOS3/GATA2/MTOR/FGFR1/AGT/HIF1A/ITGB3/MMP9/HMGB1/ITGA2/VEGFA/TGFB1/FGF1/FGF7/KDR/VEGFC/IFNG*	19
GO:0097193	intrinsic apoptotic signaling pathway	24/293	289/18670	3,27E-11	6,90E-10	3,04E-10	*TP53/SRC/AKT1/MDM2/CASP3/BAD/TNFRSF1A/HSPA1A/JAK2/TNF/TP63/TP73/HIF1A/CASP9/BAX/BCL2/BCL2L1/MMP9/BAG6/BRCA1/BRCA2/CEBPB/IFI6/INS*	24
GO:0032642	regulation of chemokine production	14/293	82/18670	3,41E-11	7,16E-10	3,15E-10	*AIF1/TLR4/GSTP1/IL1B/TNF/IL6/HIF1A/HAVCR2/CHIA/EIF2AK2/IFNG/IL4R/IL10/IL6R*	14
GO:0050918	positive chemotaxis	13/293	68/18670	3,89E-11	8,14E-10	3,59E-10	*LGALS3/IL16/F7/HMGB1/ITGA2/VEGFA/CXCL8/F3/FGF7/KDR/VEGFC/VEGFB/PGF*	13
GO:0042509	regulation of tyrosine phosphorylation of STAT protein	14/293	83/18670	4,04E-11	8,41E-10	3,71E-10	*STAT3/TNFRSF1A/JAK2/TNF/IL6/IL12B/FGFR3/IL23R/IFNG/IGF1/IL12A/IL4/LEP/IL6R*	14
GO:0051896	regulation of protein kinase B signaling	22/293	244/18670	4,52E-11	9,37E-10	4,13E-10	*SRC/AKT1/EGFR/MTOR/FGFR1/ESR1/IGF1R/TGFBR1/TNF/F7/HLA-*	22

ID	Description	GeneRatio	BgRatio	pvalue	p.adjust	qvalue	geneID	Count
							G/TGFB1/EGF/INSR/INS/IGF2/F10/F3/FGF1/FGFR3/FGF7/LEP	
GO:0031099	regeneration	20/293	198/18670	4,57E-11	9,39E-10	4,14E-10	*EGFR/PPARG/JAK2/GSTP1/IGFBP1/BCL2/TYMS/CPB2/F7/C5AR1/CD9/CEBPB/TGFB1/DHFR/PGF/HAMP/HFE/IGF1/IGF2R/IL10*	20
GO:0046890	regulation of lipid biosynthetic process	20/293	198/18670	4,57E-11	9,39E-10	4,14E-10	*NFKB1/AKT1/MTOR/TNFRSF1A/BMP2/RAN/IL1B/TNF/APOB/APOE/BRCA1/CEACAM1/CREB1/CGA/FSHB/DHCR7/INS/FGF1/IFNG/LEP*	20
GO:0097756	negative regulation of blood vessel diameter	14/293	84/18670	4,78E-11	9,78E-10	4,31E-10	*ACE/ACE2/AKT1/EGFR/ACTA2/ADRA2A/AGTR1/AGT/FGG/HTR1A/FGB/FGA/INS/LEP*	14
GO:0001914	regulation of T cell mediated cytotoxicity	10/293	33/18670	5,23E-11	1,07E-09	4,70E-10	*HLA-E/HLA-C/HLA-G/HLA-A/HLA-B/LILRB1/CEACAM1/IL12B/IL23R/IL12A*	10
GO:0048661	positive regulation of smooth muscle cell proliferation	15/293	101/18670	5,35E-11	1,08E-09	4,78E-10	*AIF1/AKT1/EGFR/MDM2/MTOR/JAK2/TNF/IL6/AGT/MMP2/MMP9/ITGA2/IGF1/IL10/IL6R*	15
GO:0046631	alpha-beta T cell activation	17/293	138/18670	5,83E-11	1,18E-09	5,19E-10	*STAT3/MTOR/ADA/IL6/BCL2/HMGB1/HLA-E/LILRB1/IL12B/INS/IL23R/FOXP3/IFNG/HFE/IL12A/IL4R/IL6R*	17
GO:0030595	leukocyte chemotaxis	21/293	224/18670	6,09E-11	1,22E-09	5,39E-10	*AIF1/LGALS3/IL1B/CCR5/IL6/IL16/F7/SERPINE1/HMGB1/C5AR1/VEGFA/CXCL8/CXCR1/VEGFC/FLT1/VEGFB/PGF/IL12A/IL1RN/IL10/IL6R*	21
GO:0022612	gland morphogenesis	16/293	120/18670	6,45E-11	1,29E-09	5,69E-10	*SRC/EGFR/AR/FGFR1/ESR1/TNF/TP63/BAX/BCL2/CPB2/CEACAM1/CEBPB/TGFB1/SULF1/PGR/FGF7*	16
GO:0050715	positive regulation of cytokine secretion	17/293	139/18670	6,55E-11	1,31E-09	5,76E-10	*AIF1/SRC/TLR4/CHUK/IL1B/TNF/MMP12/HAVCR2/HMGB1/CD14/CHIA/INS/IFNG/IL1A/IL4R/TLR1/IL10*	17
GO:0007260	tyrosine phosphorylation of STAT protein	14/293	86/18670	6,65E-11	1,32E-09	5,82E-10	*STAT3/TNFRSF1A/JAK2/TNF/IL6/IL12B/FGFR3/IL23R/IFNG/IGF1/IL12A/IL4/LEP/IL6R*	14
GO:0032680	regulation of tumor necrosis factor production	18/293	160/18670	7,31E-11	1,44E-09	6,36E-10	*TLR4/JAK2/GSTP1/HAVCR2/HMGB1/CD14/LTF/HLA-E/LILRB1/IL12B/DICER1/FOXP3/IFNG/IGF1/IL4/LEP/TLR1/IL10*	18
GO:0014068	positive regulation of phosphatidylinositol 3-kinase signaling	14/293	87/18670	7,82E-11	1,54E-09	6,77E-10	*SRC/FGFR1/IGF1R/JAK2/TNF/AGT/F2/FSHR/INSR/INS/KDR/FLT1/IGF1/LEP*	14
GO:0035265	organ growth	20/293	204/18670	7,87E-11	1,54E-09	6,77E-10	*PIM1/AKT1/MTOR/AR/FGFR1/ESR1/TGFBR1/TP73/AGT/MBL2/RBP4/LEPR/BCL2/COL6A3/COL6A1/FGFR3/FGF7/HAMP/IGF1/LEP*	20
GO:0045766	positive regulation of angiogenesis	20/293	204/18670	7,87E-11	1,54E-09	6,77E-10	*STAT3/NOS3/GATA2/IL1B/AGTR1/HIF1A/SERPINE1/BRCA1/C5AR1/VEGFA/CXCL8/F3/FGF1/KDR/VEGFC/FLT1/VEGFB/PGF/IL1A/IL10*	20
GO:0061138	morphogenesis of a branching epithelium	19/293	182/18670	7,97E-11	1,55E-09	6,83E-10	*SRC/AR/FGFR1/ESR1/ACVR1/BMP2/TNF/TP63/AGT/BCL2/VEGFA/TGFB1/SULF1/EGF/PGR/FGF1/FGF7/PGF/IL10*	19

ID	Description	GeneRatio	BgRatio	pvalue	p.adjust	qvalue	geneID	Count
GO:0032722	positive regulation of chemokine production	12/293	58/18670	8,35E-11	1,62E-09	7,13E-10	*AIF1/TLR4/IL1B/TNF/IL6/HIF1A/HAVCR2/CHIA/EIF2AK2/IFNG/IL4R/IL6R*	12
GO:0042742	defense response to bacterium	25/293	330/18670	9,08E-11	1,75E-09	7,72E-10	*TLR4/TNFRSF1A/TNF/IL6/F2/MBL2/FGB/FGA/SERPINE1/HAVCR2/C5AR1/SLC11A1/LTF/HLA-E/LCN2/CEBPB/IL12B/IL23R/HAMP/HP/IL12A/LTA/NOS2/IL10/IL6R*	25
GO:1904035	regulation of epithelial cell apoptotic process	14/293	88/18670	9,16E-11	1,76E-09	7,76E-10	*GATA2/MTOR/BAD/JAK2/TNF/IL6/FGG/FGB/FGA/SERPINE1/HLA-G/FASLG/KDR/IL4*	14
GO:0032640	tumor necrosis factor production	18/293	163/18670	9,97E-11	1,90E-09	8,37E-10	*TLR4/JAK2/GSTP1/HAVCR2/HMGB1/CD14/LTF/HLA-E/LILRB1/IL12B/DICER1/FOXP3/IFNG/IGF1/IL4/LEP/TLR1/IL10*	18
GO:1903555	regulation of tumor necrosis factor superfamily cytokine production	18/293	163/18670	9,97E-11	1,90E-09	8,37E-10	*TLR4/JAK2/GSTP1/HAVCR2/HMGB1/CD14/LTF/HLA-E/LILRB1/IL12B/DICER1/FOXP3/IFNG/IGF1/IL4/LEP/TLR1/IL10*	18
GO:0014066	regulation of phosphatidylinositol 3-kinase signaling	16/293	124/18670	1,07E-10	2,03E-09	8,93E-10	*SRC/EGFR/FGFR1/IGF1R/JAK2/TNF/AGT/F2/CEACAM1/FSHR/INSR/INS/KDR/FLT1/IGF1/LEP*	16
GO:0032602	chemokine production	14/293	89/18670	1,07E-10	2,03E-09	8,93E-10	*AIF1/TLR4/GSTP1/IL1B/TNF/IL6/HIF1A/HAVCR2/CHIA/EIF2AK2/IFNG/IL4R/IL10/IL6R*	14
GO:0002286	T cell activation involved in immune response	15/293	106/18670	1,09E-10	2,05E-09	9,01E-10	*TP53/STAT3/MTOR/LGALS3/IL6/HAVCR2/HMGB1/SLC11A1/LILRB1/CEACAM1/IL12B/IL23R/FOXP3/IFNG/IL4R*	15
GO:2001239	regulation of extrinsic apoptotic signaling pathway in absence of ligand	11/293	47/18670	1,24E-10	2,32E-09	1,02E-09	*AKT1/GATA1/FGFR1/HSPA1B/HSPA1A/IL1B/TNF/BCL2/BCL2L1/IFI6/IL1A*	11
GO:0001909	leukocyte mediated cytotoxicity	15/293	107/18670	1,25E-10	2,32E-09	1,02E-09	*F2/HAVCR2/HLA-E/HLA-C/HLA-G/HLA-A/HLA-B/LILRB1/CEACAM1/IL12B/IL23R/KIR3DL1/IL12A/LEP/NOS2*	15
GO:0051341	regulation of oxidoreductase activity	15/293	107/18670	1,25E-10	2,32E-09	1,02E-09	*NFKB1/AKT1/EGFR/NOS3/IL1B/AGTR1/TNF/AGT/HIF1A/APOE/DHFR/INS/IFNG/HP/LEP*	15
GO:0030194	positive regulation of blood coagulation	9/293	26/18670	1,26E-10	2,33E-09	1,03E-09	*TLR4/F2/HRG/CPB2/F12/F7/SERPINE1/THBD/F3*	9
GO:1900048	positive regulation of hemostasis	9/293	26/18670	1,26E-10	2,33E-09	1,03E-09	*TLR4/F2/HRG/CPB2/F12/F7/SERPINE1/THBD/F3*	9
GO:0045862	positive regulation of proteolysis	26/293	363/18670	1,29E-10	2,37E-09	1,05E-09	*SRC/AKT1/STAT3/MDM2/BAD/HSPA1B/HSPA1A/PPARG/JAK2/FN1/ADRA2A/IL1B/TNF/CASP9/BAX/F12/APOE/BAG6/HMGB1/CEBPA/FBLN1/EGF/FASLG/F3/FMR1/IFNG*	26
GO:0050707	regulation of cytokine secretion	20/293	210/18670	1,33E-10	2,43E-09	1,07E-09	*AIF1/SRC/TLR4/CHUK/FN1/IL1B/TNF/MMP12/HAVCR2/HMGB1/CD14/LILRB1/CHIA/INS/FOXP3/IFNG/IL1A/IL4R/TLR1/IL10*	20

ID	Description	GeneRatio	BgRatio	pvalue	p.adjust	qvalue	geneID	Count
GO:1901099	negative regulation of signal transduction in absence of ligand	10/293	36/18670	1,38E-10	2,50E-09	1,10E-09	*AKT1/GATA1/HSPA1B/HSPA1A/IL1B/TNF/BCL2/BCL2L1/IFI6/IL1A*	10
GO:2001240	negative regulation of extrinsic apoptotic signaling pathway in absence of ligand	10/293	36/18670	1,38E-10	2,50E-09	1,10E-09	*AKT1/GATA1/HSPA1B/HSPA1A/IL1B/TNF/BCL2/BCL2L1/IFI6/IL1A*	10
GO:0014909	smooth muscle cell migration	14/293	91/18670	1,46E-10	2,63E-09	1,16E-09	*ACE/AIF1/SRC/MDM2/IGFBP3/GSTP1/AGT/PRKG1/BCL2/PLAT/ITGB3/SERPINE1/ITGA2/IGF1*	14
GO:0045639	positive regulation of myeloid cell differentiation	14/293	91/18670	1,46E-10	2,63E-09	1,16E-09	*STAT3/STAT5B/GATA2/GATA1/HSPA1B/HSPA1A/TNF/ARNT/HIF1A/CREB1/TGFB1/IL12B/IL23R/IFNG*	14
GO:0009755	hormone-mediated signaling pathway	21/293	235/18670	1,50E-10	2,69E-09	1,18E-09	*SRC/THRB/NR3C1/ESR2/AR/NR2F2/ESR1/PPARG/JAK2/RAN/TP63/REN/PRLR/HNF4A/BRCA1/LHB/FSHB/FSHR/CNOT1/RXRB/PGR*	21
GO:0009896	positive regulation of catabolic process	28/293	423/18670	1,52E-10	2,71E-09	1,20E-09	*AKT1/MDM2/BAD/IKBKG/HSPA1B/HSPA1A/ADRA2A/ADRB2/IL1B/TNF/IL6/ARNT/HIF1A/BAX/APC/APOE/BAG6/HMGB1/CEBPA/CNOT1/EGF/INSR/INS/KDR/FMR1/IFNG/IGF1/IL4*	28
GO:0016049	cell growth	30/293	484/18670	1,60E-10	2,85E-09	1,25E-09	*TP53/AKT1/EGFR/ESR2/MTOR/HSPA1B/HSPA1A/IGFBP3/PPARG/FN1/L1CAM/TGFBR1/AGTR1/AGT/F2/HRG/IGFBP1/HNF4A/SGK1/FLRT3/BCL2/APOE/MAP2/IGFBP4/CEACAM1/VEGFA/TGFB1/INS/HAMP/IGF1*	30
GO:0002262	myeloid cell homeostasis	17/293	147/18670	1,61E-10	2,85E-09	1,25E-09	*STAT3/STAT5B/CASP3/GATA2/GATA1/HSPA1B/HSPA1A/JAK2/IL6/ARNT/HIF1A/BAX/INHA/HMGB1/VEGFA/FOXP3/SLC4A1*	17
GO:0071496	cellular response to external stimulus	25/293	339/18670	1,61E-10	2,85E-09	1,25E-09	*PIM1/NFKB1/TP53/AIF1/AKT1/TLR4/EGFR/MDM2/MTOR/BAD/TNFRSF1A/PPARG/GSTP1/IL1B/AGT/BCL2/BRIP1/ITGA2/LCN2/MMP7/TGFB1/COL1A1/EIF2AK2/HFE/LEP*	25
GO:0071706	tumor necrosis factor superfamily cytokine production	18/293	168/18670	1,65E-10	2,91E-09	1,28E-09	*TLR4/JAK2/GSTP1/HAVCR2/HMGB1/CD14/LTF/HLA-E/LILRB1/IL12B/DICER1/FOXP3/IFNG/IGF1/IL4/LEP/TLR1/IL10*	18
GO:0042310	vasoconstriction	13/293	76/18670	1,69E-10	2,98E-09	1,31E-09	*ACE/ACE2/AKT1/EGFR/ACTA2/ADRA2A/AGTR1/AGT/FGG/HTR1A/FGB/FGA/LEP*	13
GO:0014065	phosphatidylinositol 3-kinase signaling	17/293	148/18670	1,79E-10	3,13E-09	1,38E-09	*SRC/AKT1/EGFR/FGFR1/IGF1R/JAK2/TNF/AGT/F2/CEACAM1/FSHR/INSR/INS/KDR/FLT1/IGF1/LEP*	17
GO:0050820	positive regulation of coagulation	9/293	27/18670	1,87E-10	3,26E-09	1,44E-09	*TLR4/F2/HRG/CPB2/F12/F7/SERPINE1/THBD/F3*	9
GO:1903038	negative regulation of leukocyte cell-cell adhesion	16/293	129/18670	1,95E-10	3,39E-09	1,49E-09	*AKT1/CASP3/LGALS3/HAVCR2/HMGB1/HLA-G/LILRB1/CEACAM1/CEBPB/TGFB1/CTLA4/FOXP3/HFE/IL4R/PDE5A/IL10*	16
GO:0033273	response to vitamin	14/293	93/18670	1,97E-10	3,41E-09	1,50E-09	*PIM1/EGFR/MDM2/PPARG/GSTP1/ADA/TYMS/F7/BRIP1/ITGA2/TGFB1/COL1A1/HAMP/LEP*	14
GO:0006809	nitric oxide biosynthetic process	13/293	77/18670	2,01E-10	3,46E-09	1,53E-09	*AIF1/AKT1/TLR4/NOS3/MTOR/JAK2/IL1B/TNF/AGT/INSR/IFNG/NOS2/IL10*	13

ID	Description	GeneRatio	BgRatio	pvalue	p.adjust	qvalue	geneID	Count
GO:0043112	receptor metabolic process	19/293	192/18670	2,02E-10	3,46E-09	1,53E-09	*ACE2/PPARG/JAK2/TNF/HIF1A/APOE/ITGB3/LILRB1/CD9/CEACAM1/VEGFA/TGFB1/CXCL8/CXCR1/EGF/FMR1/IFNG/TFRC/IL10*	19
GO:0010544	negative regulation of platelet activation	8/293	19/18670	2,18E-10	3,73E-09	1,64E-09	*NOS3/FGG/F2/PRKG1/APOE/CD9/CEACAM1/THBD*	8
GO:0030217	T cell differentiation	21/293	240/18670	2,21E-10	3,76E-09	1,66E-09	*TP53/STAT3/MTOR/BAD/ADA/IL6/LEPR/BCL2/HMGB1/CD8A/HLA-G/TGFB1/IL12B/CTLA4/IL23R/FOXP3/IFNG/IL12A/IL4R/IL4/LEP*	21
GO:0050777	negative regulation of immune response	17/293	150/18670	2,21E-10	3,76E-09	1,66E-09	*LGALS3/PPARG/TNF/MMP12/HAVCR2/HLA-E/HLA-G/LILRB1/CEACAM1/TGFB1/IL12B/CTLA4/INS/FOXP3/HFE/IL4R/IL10*	17
GO:0001701	in utero embryonic development	26/293	373/18670	2,33E-10	3,94E-09	1,73E-09	*TP53/AKT1/EGFR/NOS3/GATA2/AR/NR2F2/GATA1/FGFR1/ACVR1/TGFBR1/BMP2/ADA/ARNT/ARNT2/HIF1A/BCL2L1/APOB/BRCA2/NSDHL/CEBPA/CEBPB/VEGFA/IGF2/IGF1/IL10*	26
GO:0051098	regulation of binding	26/293	373/18670	2,33E-10	3,94E-09	1,73E-09	*ACE/SRC/AKT1/GATA1/LGALS3/PPARG/JAK2/TGFBR1/BMP2/RAN/ADRB2/BAX/BCL2/APOE/MAP2/MMP9/HMGB1/ITGA2/TGFB1/EGF/FMR1/IFNG/HFE/IGF1/MAPRE3/IL10*	26
GO:0001910	regulation of leukocyte mediated cytotoxicity	13/293	78/18670	2,38E-10	4,00E-09	1,76E-09	*HAVCR2/HLA-E/HLA-C/HLA-G/HLA-A/HLA-B/LILRB1/CEACAM1/IL12B/IL23R/IL12A/LEP/NOS2*	13
GO:0050868	negative regulation of T cell activation	15/293	112/18670	2,42E-10	4,05E-09	1,79E-09	*CASP3/LGALS3/HAVCR2/HMGB1/HLA-G/LILRB1/CEACAM1/CEBPB/TGFB1/CTLA4/FOXP3/HFE/IL4R/PDE5A/IL10*	15
GO:0023061	signal release	29/293	462/18670	2,45E-10	4,10E-09	1,81E-09	*EGFR/FGFR1/BAD/JAK2/ADRA2A/IL1B/AGTR1/TNF/IL6/AGT/REN/FGG/RBP4/HIF1A/HNF4A/HTR1A/INHA/FGB/FGA/CREB1/CFTR/INS/FMR1/IFNG/HFE/HNF1A/IL1RN/LEP/NOS2*	29
GO:0048009	insulin-like growth factor receptor signaling pathway	10/293	38/18670	2,49E-10	4,15E-09	1,83E-09	*AKT1/AR/IGF1R/IGFBP3/BMP2/IGFBP1/IGFBP4/IGF1/IGFBP6/IGF2R*	10
GO:0072001	renal system development	23/293	293/18670	2,62E-10	4,35E-09	1,92E-09	*ACE/FGFR1/ACTA2/TGFBR1/BMP2/AGTR1/TP73/AGT/REN/RBP4/CASP9/BAX/BCL2/ITGB4/MMP9/BAG6/VEGFA/TGFB1/FBN1/SULF1/FGF1/PGF/IL6R*	23
GO:0048638	regulation of developmental growth	25/293	347/18670	2,63E-10	4,35E-09	1,92E-09	*PIM1/AKT1/STAT3/STAT5B/MTOR/AR/FGFR1/FN1/L1CAM/TGFBR1/ADRB2/TP73/RBP4/BCL2/APOE/MAP2/VEGFA/CREB1/PRL/INSR/IGF2/FGFR3/HAMP/IGF1/LEP*	25
GO:0001659	temperature homeostasis	18/293	173/18670	2,68E-10	4,41E-09	1,95E-09	*STAT3/TLR4/IGF1R/JAK2/ADRB2/IL1B/TNF/LEPR/PRLR/APC/LCN2/CEBPB/VEGFA/IL1A/IL4R/IL4/LEP/LAMA4*	18
GO:0032768	regulation of monooxygenase activity	12/293	64/18670	2,83E-10	4,64E-09	2,05E-09	*NFKB1/AKT1/EGFR/NOS3/IL1B/TNF/HIF1A/APOE/DHFR/INS/IFNG/LEP*	12

ID	Description	GeneRatio	BgRatio	pvalue	p.adjust	qvalue	geneID	Count
GO:0001763	morphogenesis of a branching structure	19/293	196/18670	2,87E-10	4,68E-09	2,06E-09	*SRC/AR/FGFR1/ESR1/ACVR1/BMP2/TNF/TP63/AGT/BCL2/VEGFA/TGFB1/SULF1/EGF/PGR/FGF1/FGF7/PGF/IL10*	19
GO:0006694	steroid biosynthetic process	19/293	196/18670	2,87E-10	4,68E-09	2,06E-09	*NFKB1/BMP2/RAN/IL1B/TNF/PRLR/APOB/APOE/NSDHL/CFTR/CGA/LHB/FSHB/CNBP/DHCR7/EBP/FGF1/IFNG/LEP*	19
GO:0048511	rhythmic process	23/293	295/18670	3,00E-10	4,88E-09	2,15E-09	*TP53/SRC/EGFR/CASP3/NOS3/MTOR/ESR1/PPARG/ADA/AHR/HNF4A/TYMS/F7/SERPINE1/CREB1/CRTC1/FSHB/FSHR/MMP7/TIMP4/PGR/LEP/NOS2*	23
GO:0050729	positive regulation of inflammatory response	17/293	153/18670	3,03E-10	4,90E-09	2,16E-09	*TLR4/EGFR/TNFRSF1A/JAK2/IL1B/AGTR1/TNF/IL6/AGT/F12/SERPINE1/HLA-E/ITGA2/CEBPA/CEBPB/IL12B/LTA*	17
GO:0031667	response to nutrient levels	30/293	499/18670	3,34E-10	5,38E-09	2,37E-09	*PIM1/TP53/SRC/AKT1/EGFR/MDM2/MTOR/PPARG/GSTP1/ADA/ADRB2/IL1B/SERPINC1/BCL2/TYMS/F7/APOE/BRIP1/ITGA2/LCN2/CREB1/PRL/MMP7/TGFB1/COL1A1/EIF2AK2/HAMP/HFE/LEP/LTA*	30
GO:0032729	positive regulation of interferon-gamma production	12/293	65/18670	3,42E-10	5,50E-09	2,42E-09	*TLR4/IL1B/TNF/IL1R1/HAVCR2/SLC11A1/CD14/IL12B/IL23R/IL12A/LTA/IL6R*	12
GO:0030098	lymphocyte differentiation	25/293	353/18670	3,77E-10	6,04E-09	2,66E-09	*TP53/STAT3/MTOR/BAD/ADA/IL6/LEPR/BAX/BCL2/INHA/HMGB1/CD8A/HLA-G/TGFB1/IL12B/CTLA4/IL23R/FOXP3/IFNG/HNF1A/IL12A/IL4R/IL4/LEP/IL10*	25
GO:0010575	positive regulation of vascular endothelial growth factor production	9/293	29/18670	3,89E-10	6,20E-09	2,73E-09	*IL1B/IL6/ARNT/HIF1A/BRCA1/C5AR1/TGFB1/SULF1/IL1A*	9
GO:0009615	response to virus	24/293	326/18670	3,93E-10	6,25E-09	2,76E-09	*SRC/CHUK/BAD/IKBKG/ACTA2/IL1B/TNF/IL6/FLNA/BCL2/BCL2L1/APOB/MMP12/LILRB1/LCN2/IL12B/EIF2AK2/IFI6/IFI44/FMR1/IL23R/FOXP3/IFNG/IL12A*	24
GO:0070372	regulation of ERK1 and ERK2 cascade	23/293	300/18670	4,18E-10	6,63E-09	2,92E-09	*SRC/TLR4/EGFR/FN1/GSTP1/BMP2/IL1B/TNF/FGG/FGB/FGA/APOE/TIMP3/HAVCR2/HMGB1/C5AR1/CEACAM1/FSHR/TGFB1/FBLN1/FGFR3/KDR/FLT1*	23
GO:0046209	nitric oxide metabolic process	13/293	82/18670	4,55E-10	7,19E-09	3,17E-09	*AIF1/AKT1/TLR4/NOS3/MTOR/JAK2/IL1B/TNF/AGT/INSR/IFNG/NOS2/IL10*	13
GO:0010951	negative regulation of endopeptidase activity	21/293	250/18670	4,70E-10	7,35E-09	3,24E-09	*SRC/AKT1/MDM2/AGT/HRG/SERPINA10/SERPINA6/SERPINC1/FETUB/SERPINA1/TIMP1/TIMP3/SERPINE1/MMP9/LTF/VEGFA/TIMP4/TIMP2/COL6A3/IFI6/TFPI*	21
GO:0030072	peptide hormone secretion	21/293	250/18670	4,70E-10	7,35E-09	3,24E-09	*EGFR/BAD/JAK2/ADRA2A/IL1B/TNF/IL6/FGG/RBP4/HIF1A/HNF4A/FGB/FGA/CFTR/INS/IFNG/HFE/HNF1A/IL1RN/LEP/NOS2*	21
GO:0071383	cellular response to steroid hormone stimulus	21/293	250/18670	4,70E-10	7,35E-09	3,24E-09	*SRC/THRB/NR3C1/EGFR/ESR2/AR/NR2F2/ESR1/PPARG/JAK2/GSTP1/RAN/TP63/HNF4A/CASP9/BRCA1/CNOT1/RXRB/TGFB1/PGR/TFPI*	21

ID	Description	GeneRatio	BgRatio	pvalue	p.adjust	qvalue	geneID	Count
GO:0045940	positive regulation of steroid metabolic process	9/293	30/18670	5,47E-10	8,54E-09	3,76E-09	*IL1B/AGTR1/TNF/AGT/APOE/CGA/FSHB/FGF1/IFNG*	9
GO:0002700	regulation of production of molecular mediator of immune response	16/293	139/18670	6,02E-10	9,35E-09	4,12E-09	*TLR4/IL1B/TNF/IL6/IL1R1/RBP4/HLA-E/HLA-G/LILRB1/TGFB1/FOXP3/HFE/TFRC/IL4R/IL4/IL10*	16
GO:0030100	regulation of endocytosis	22/293	281/18670	6,85E-10	1,06E-08	4,67E-09	*SRC/GATA2/LGALS3/PPARG/IL1B/TNF/MBL2/APOE/ITGB3/SERPINE1/HMGB1/SLC11A1/CD14/LILRB1/ITGA2/VEGFA/TGFB1/EGF/FMR1/IFNG/HFE/IL4*	22
GO:2001057	reactive nitrogen species metabolic process	13/293	85/18670	7,23E-10	1,12E-08	4,92E-09	*AIF1/AKT1/TLR4/NOS3/MTOR/JAK2/IL1B/TNF/AGT/INSR/IFNG/NOS2/IL10*	13
GO:0006509	membrane protein ectodomain proteolysis	10/293	42/18670	7,34E-10	1,13E-08	4,98E-09	*ADRA2A/IL1B/TNF/APOE/TIMP1/TIMP3/TIMP4/TIMP2/IFNG/IL10*	10
GO:0048017	inositol lipid-mediated signaling	18/293	184/18670	7,37E-10	1,13E-08	4,98E-09	*PLD1/SRC/AKT1/EGFR/FGFR1/IGF1R/JAK2/TNF/AGT/F2/CEACAM1/FSHR/INSR/INS/KDR/FLT1/IGF1/LEP*	18
GO:0031960	response to corticosteroid	17/293	162/18670	7,44E-10	1,14E-08	5,01E-09	*AIF1/SRC/NR3C1/EGFR/CASP3/BAD/GSTP1/TNF/IL6/CASP9/BCL2/TYMS/TGFB1/COL1A1/PAPPA/IL1RN/IL10*	17
GO:1902895	positive regulation of pri-miRNA transcription by RNA polymerase II	9/293	31/18670	7,61E-10	1,16E-08	5,11E-09	*TP53/STAT3/NR3C1/GATA2/BMP2/HIF1A/PRL/TGFB1/IL10*	9
GO:0042445	hormone metabolic process	20/293	232/18670	7,83E-10	1,19E-08	5,24E-09	*ACE/ACE2/NFKB1/FGFR1/ESR1/BMP2/IL1B/TNF/REN/SERPINA6/RBP4/ARNT/HIF1A/CGA/LHB/FSHB/DHCR7/IFNG/HFE/LEP*	20
GO:1903409	reactive oxygen species biosynthetic process	15/293	122/18670	8,24E-10	1,25E-08	5,50E-09	*AIF1/AKT1/STAT3/TLR4/NOS3/MTOR/JAK2/IL1B/TNF/AGT/INSR/INS/IFNG/NOS2/IL10*	15
GO:0071229	cellular response to acid chemical	19/293	209/18670	8,59E-10	1,30E-08	5,71E-09	*SRC/AKT1/EGFR/MTOR/PPARG/TNF/MMP2/BCL2L1/APOB/CEBPB/VEGFA/CREB1/COL1A1/COL5A2/COL6A1/COL1A2/KDR/HAMP/LEP*	19
GO:0014812	muscle cell migration	14/293	104/18670	9,05E-10	1,36E-08	5,98E-09	*ACE/AIF1/SRC/MDM2/IGFBP3/GSTP1/AGT/PRKG1/BCL2/PLAT/ITGB3/SERPINE1/ITGA2/IGF1*	14
GO:0071887	leukocyte apoptotic process	14/293	104/18670	9,05E-10	1,36E-08	5,98E-09	*TP53/AKT1/CASP3/LGALS3/ADA/CCR5/IL6/HIF1A/CASP9/BAX/LILRB1/FASLG/TSC22D3/IL10*	14
GO:0001503	ossification	26/293	398/18670	9,40E-10	1,40E-08	6,19E-09	*AKT1/EGFR/GATA1/IGFBP3/ACVR1/BMP2/ADRB2/TNF/IL6/TP63/HIF1A/MMP2/BCL2/LTF/CEBPA/CEBPB/TGFB1/COL1A1/COL5A2/COL6A1/COL1A2/IGF2/FGFR3/IGF1/LEP/IL6R*	26
GO:0045429	positive regulation of nitric oxide biosynthetic process	10/293	43/18670	9,44E-10	1,41E-08	6,20E-09	*AIF1/AKT1/TLR4/MTOR/JAK2/IL1B/TNF/AGT/INSR/IFNG*	10

ID	Description	GeneRatio	BgRatio	pvalue	p.adjust	qvalue	geneID	Count
GO:0035690	cellular response to drug	25/293	369/18670	9,48E-10	1,41E-08	6,21E-09	*NFKB1/TP53/AIF1/SRC/TRPC6/NR3C1/EGFR/MDM2/BAD/IL1B/TNF/IL6/REN/AHR/CASP9/MMP3/BRCA1/LCN2/CFTR/TGFB1/KDR/FMR1/TFRC/NOS2/IL10*	25
GO:0002690	positive regulation of leukocyte chemotaxis	13/293	87/18670	9,73E-10	1,44E-08	6,35E-09	*AIF1/IL6/F7/SERPINE1/HMGB1/C5AR1/VEGFA/CXCL8/VEGFC/VEGFB/PGF/IL12A/IL6R*	13
GO:0019218	regulation of steroid metabolic process	15/293	124/18670	1,04E-09	1,53E-08	6,75E-09	*NFKB1/BMP2/RAN/IL1B/AGTR1/TNF/AGT/APOB/APOE/CGA/FSHB/DHCR7/FGF1/IFNG/LEP*	15
GO:0010466	negative regulation of peptidase activity	21/293	262/18670	1,11E-09	1,63E-08	7,18E-09	*SRC/AKT1/MDM2/AGT/HRG/SERPINA10/SERPINA6/SERPINC1/FETUB/SERPINA1/TIMP1/TIMP3/SERPINE1/MMP9/LTF/VEGFA/TIMP4/TIMP2/COL6A3/IFI6/TFPI*	21
GO:0048002	antigen processing and presentation of peptide antigen	18/293	189/18670	1,14E-09	1,67E-08	7,37E-09	*ACE/CHUK/IKBKG/SLC11A1/HLA-E/HLA-C/HLA-G/HLA-A/HLA-B/TAP2/HFE/HLA-DQA1/HLA-DRB5/HLA-DRA/HLA-DQB2/HLA-DRB1/HLA-DQB1/HLA-DQA2*	18
GO:0042136	neurotransmitter biosynthetic process	14/293	106/18670	1,17E-09	1,71E-08	7,54E-09	*AIF1/AKT1/TLR4/NOS3/MTOR/JAK2/IL1B/TNF/AGT/PAH/INSR/IFNG/NOS2/IL10*	14
GO:1904407	positive regulation of nitric oxide metabolic process	10/293	44/18670	1,20E-09	1,76E-08	7,74E-09	*AIF1/AKT1/TLR4/MTOR/JAK2/IL1B/TNF/AGT/INSR/IFNG*	10
GO:0042063	gliogenesis	22/293	290/18670	1,24E-09	1,80E-08	7,94E-09	*AKT1/STAT3/TLR4/EGFR/MTOR/PPARG/GSTP1/BMP2/IL1B/TNF/IL6/TP73/F2/LEPR/C5AR1/CD9/CREB1/TGFB1/DICER1/IFNG/LTA/ROR1*	22
GO:0051250	negative regulation of lymphocyte activation	16/293	146/18670	1,25E-09	1,81E-08	7,98E-09	*CASP3/LGALS3/INHA/HAVCR2/HMGB1/HLA-G/LILRB1/CEACAM1/CEBPB/TGFB1/CTLA4/FOXP3/HFE/IL4R/PDE5A/IL10*	16
GO:0002483	antigen processing and presentation of endogenous peptide antigen	7/293	15/18670	1,26E-09	1,81E-08	7,98E-09	*HLA-E/HLA-C/HLA-G/HLA-A/HLA-B/TAP2/HFE*	7
GO:0019885	antigen processing and presentation of endogenous peptide antigen via MHC class I	7/293	15/18670	1,26E-09	1,81E-08	7,98E-09	*HLA-E/HLA-C/HLA-G/HLA-A/HLA-B/TAP2/HFE*	7
GO:0042976	activation of Janus kinase activity	7/293	15/18670	1,26E-09	1,81E-08	7,98E-09	*JAK2/AGT/PRLR/IL12B/IL23R/IL4/IL6R*	7
GO:0031348	negative regulation of defense response	20/293	239/18670	1,32E-09	1,89E-08	8,32E-09	*NFKB1/TNFRSF1A/PPARG/GSTP1/ADA/F2/PROC/APOE/MMP12/HAVCR2/HLA-E/HLA-G/LILRB1/CEACAM1/IL12B/INS/FOXP3/IGF1/IL4/IL10*	20
GO:0019229	regulation of vasoconstriction	11/293	58/18670	1,40E-09	1,99E-08	8,78E-09	*ACE/ACE2/AKT1/EGFR/ADRA2A/AGTR1/AGT/FGG/FGB/FGA/LEP*	11
GO:0070997	neuron death	24/293	348/18670	1,46E-09	2,08E-08	9,18E-09	*TP53/AKT1/STAT3/THRB/TLR4/CASP3/MTOR/BAD/IKBKG/JAK2/TNF/TP63/HIF1A/CASP9/BAX/BCL2/BCL2L1/APOE/C5AR1/CEBPB/CREB1/FASLG/IFNG/IL10*	24

ID	Description	GeneRatio	BgRatio	pvalue	p.adjust	qvalue	geneID	Count
GO:0007568	aging	23/293	321/18670	1,56E-09	2,22E-08	9,78E-09	*TP53/AKT1/STAT3/TRPC6/MTOR/ADA/TP63/AGT/IGFBP1/CASP9/BCL2/TYMS/TIMP1/SERPINE1/BRCA2/HLA-G/CREB1/MMP7/TGFB1/TIMP2/HAMP/LEP/IL10*	23
GO:0010001	glial cell differentiation	19/293	218/18670	1,75E-09	2,47E-08	1,09E-08	*AKT1/STAT3/TLR4/EGFR/MTOR/PPARG/GSTP1/BMP2/IL1B/TNF/IL6/TP73/F2/C5AR1/CD9/TGFB1/DICER1/IFNG/ROR1*	19
GO:0007584	response to nutrient	19/293	219/18670	1,89E-09	2,66E-08	1,17E-08	*PIM1/EGFR/MDM2/MTOR/PPARG/GSTP1/ADA/IL1B/SERPINC1/TYMS/F7/BRIP1/ITGA2/CREB1/TGFB1/COL1A1/HAMP/LEP/LTA*	19
GO:0010574	regulation of vascular endothelial growth factor production	9/293	34/18670	1,90E-09	2,67E-08	1,18E-08	*IL1B/IL6/ARNT/HIF1A/BRCA1/C5AR1/TGFB1/SULF1/IL1A*	9
GO:0030810	positive regulation of nucleotide biosynthetic process	10/293	46/18670	1,92E-09	2,69E-08	1,19E-08	*STAT3/NOS3/ARNT/HIF1A/INSR/INS/IFNG/IGF1/IL4/NOS2*	10
GO:1900373	positive regulation of purine nucleotide biosynthetic process	10/293	46/18670	1,92E-09	2,69E-08	1,19E-08	*STAT3/NOS3/ARNT/HIF1A/INSR/INS/IFNG/IGF1/IL4/NOS2*	10
GO:0150076	neuroinflammatory response	12/293	75/18670	1,93E-09	2,69E-08	1,19E-08	*AIF1/EGFR/JAK2/IL1B/TNF/IL6/MMP3/MMP9/C5AR1/IFNG/IGF1/IL4*	12
GO:0034116	positive regulation of heterotypic cell-cell adhesion	7/293	16/18670	2,21E-09	3,07E-08	1,35E-08	*IL1B/TNF/FGG/FGB/FGA/CEACAM6/IL10*	7
GO:0046887	positive regulation of hormone secretion	15/293	131/18670	2,25E-09	3,12E-08	1,37E-08	*EGFR/FGFR1/BAD/JAK2/FGG/RBP4/HIF1A/INHA/FGB/FGA/CREB1/CFTR/INS/HFE/LEP*	15
GO:0061614	pri-miRNA transcription by RNA polymerase II	10/293	47/18670	2,41E-09	3,33E-08	1,47E-08	*TP53/STAT3/NR3C1/GATA2/PPARG/BMP2/HIF1A/PRL/TGFB1/IL10*	10
GO:0090303	positive regulation of wound healing	11/293	61/18670	2,46E-09	3,38E-08	1,49E-08	*TLR4/MTOR/ADRA2A/F2/HRG/CPB2/F12/F7/SERPINE1/THBD/F3*	11
GO:0045807	positive regulation of endocytosis	16/293	153/18670	2,50E-09	3,43E-08	1,51E-08	*GATA2/PPARG/IL1B/TNF/MBL2/APOE/SERPINE1/SLC11A1/CD14/ITGA2/VEGFA/EGF/FMR1/IFNG/HFE/IL4*	16
GO:0042116	macrophage activation	13/293	95/18670	2,97E-09	4,05E-08	1,79E-08	*AIF1/TLR4/JAK2/TNF/IL6/HAVCR2/C5AR1/SLC11A1/CEBPA/IFNG/IL4R/TLR1/IL10*	13
GO:0050810	regulation of steroid biosynthetic process	13/293	95/18670	2,97E-09	4,05E-08	1,79E-08	*NFKB1/BMP2/RAN/IL1B/TNF/APOB/APOE/CGA/FSHB/DHCR7/FGF1/IFNG/LEP*	13
GO:0019882	antigen processing and presentation	19/293	226/18670	3,19E-09	4,34E-08	1,91E-08	*ACE/CHUK/IKBKG/SLC11A1/CD8A/HLA-E/HLA-C/HLA-G/HLA-A/HLA-B/TAP2/HFE/HLA-DQA1/HLA-DRB5/HLA-DRA/HLA-DQB2/HLA-DRB1/HLA-DQB1/HLA-DQA2*	19
GO:0001822	kidney development	21/293	278/18670	3,22E-09	4,37E-08	1,93E-08	*ACE/FGFR1/ACTA2/TGFBR1/BMP2/AGTR1/TP73/AGT/REN/CASP9/BAX/BCL2/MMP9/BAG6/VEGFA/TGFB1/FBN1/SULF1/FGF1/PGF/IL6R*	21

ID	Description	GeneRatio	BgRatio	pvalue	p.adjust	qvalue	geneID	Count
GO:0010573	vascular endothelial growth factor production	9/293	36/18670	3,32E-09	4,49E-08	1,98E-08	*IL1B/IL6/ARNT/HIF1A/BRCA1/C5AR1/TGFB1/SULF1/IL1A*	9
GO:0001889	liver development	15/293	135/18670	3,43E-09	4,63E-08	2,04E-08	*EGFR/ADA/HNF4A/TYMS/CPB2/ITGA2/CEACAM1/CEBPA/CEBPB/TGFB1/HAMP/HFE/HNF1A/IGF2R/IL10*	15
GO:0051099	positive regulation of binding	17/293	179/18670	3,48E-09	4,68E-08	2,06E-08	*ACE/LGALS3/PPARG/JAK2/BMP2/RAN/APOE/MMP9/HMGB1/ITGA2/TGFB1/EGF/FMR1/IFNG/HFE/IGF1/MAPRE3*	17
GO:2000027	regulation of animal organ morphogenesis	20/293	253/18670	3,54E-09	4,75E-08	2,09E-08	*PIM1/THRB/AR/FGFR1/ESR1/ACVR1/TGFBR1/BMP2/TNF/AGT/BAX/BCL2/CPB2/CEACAM1/VEGFA/TGFB1/SULF1/FGF1/FGF7/ROR1*	20
GO:0071260	cellular response to mechanical stimulus	12/293	79/18670	3,58E-09	4,79E-08	2,11E-08	*NFKB1/AKT1/TLR4/EGFR/BAD/TNFRSF1A/IL1B/AGT/ITGA2/MMP7/TGFB1/COL1A1*	12
GO:0001913	T cell mediated cytotoxicity	10/293	49/18670	3,72E-09	4,97E-08	2,19E-08	*HLA-E/HLA-C/HLA-G/HLA-A/HLA-B/LILRB1/CEACAM1/IL12B/IL23R/IL12A*	10
GO:0001916	positive regulation of T cell mediated cytotoxicity	8/293	26/18670	4,09E-09	5,40E-08	2,38E-08	*HLA-E/HLA-C/HLA-G/HLA-A/HLA-B/IL12B/IL23R/IL12A*	8
GO:0002360	T cell lineage commitment	8/293	26/18670	4,09E-09	5,40E-08	2,38E-08	*TP53/STAT3/MTOR/IL6/BCL2/IL12B/IL23R/FOXP3*	8
GO:0032800	receptor biosynthetic process	8/293	26/18670	4,09E-09	5,40E-08	2,38E-08	*ACE2/PPARG/JAK2/TNF/HIF1A/ITGB3/IFNG/IL10*	8
GO:0034114	regulation of heterotypic cell-cell adhesion	8/293	26/18670	4,09E-09	5,40E-08	2,38E-08	*IL1B/TNF/FGG/FGB/FGA/CEACAM6/IL1RN/IL10*	8
GO:0048015	phosphatidylinositol-mediated signaling	17/293	181/18670	4,12E-09	5,43E-08	2,39E-08	*SRC/AKT1/EGFR/FGFR1/IGF1R/JAK2/TNF/AGT/F2/CEACAM1/FSHR/INSR/INS/KDR/FLT1/IGF1/LEP*	17
GO:0022617	extracellular matrix disassembly	12/293	80/18670	4,15E-09	5,45E-08	2,40E-08	*TMPRSS6/IL6/MMP2/MMP12/MMP3/TIMP1/MMP9/MMP1/MMP7/TGFB1/TIMP2/MMP15*	12
GO:0032885	regulation of polysaccharide biosynthetic process	9/293	37/18670	4,32E-09	5,66E-08	2,49E-08	*NFKB1/AKT1/MTOR/TGFB1/EGF/INSR/INS/IGF2/IGF1*	9
GO:0010906	regulation of glucose metabolic process	14/293	117/18670	4,36E-09	5,69E-08	2,51E-08	*TP53/SRC/AKT1/MTOR/BAD/IGFBP3/LEPR/LCMT1/IGFBP4/INSR/INS/IGF2/IGF1/LEP*	14
GO:0008584	male gonad development	15/293	138/18670	4,65E-09	6,04E-08	2,66E-08	*AR/GATA1/ESR1/TGFBR1/REN/FLNA/BAX/BCL2/BCL2L1/INHA/BRIP1/LHB/FSHB/FSHR/INSR*	15
GO:0061008	hepaticobiliary system development	15/293	138/18670	4,65E-09	6,04E-08	2,66E-08	*EGFR/ADA/HNF4A/TYMS/CPB2/ITGA2/CEACAM1/CEBPA/CEBPB/TGFB1/HAMP/HFE/HNF1A/IGF2R/IL10*	15
GO:0110110	positive regulation of animal organ morphogenesis	12/293	81/18670	4,80E-09	6,22E-08	2,74E-08	*PIM1/AR/FGFR1/ACVR1/TGFBR1/BMP2/AGT/BAX/VEGFA/TGFB1/FGF1/FGF7*	12
GO:0034612	response to tumor necrosis factor	22/293	312/18670	4,82E-09	6,22E-08	2,74E-08	*NFKB1/AKT1/TRAF1/CASP3/CHUK/IKBKG/TNFRSF1A/HSPA1B/HSPA1A/JAK2/GSTP1/TNF/APOB/UBD/BRCA1/CD14/LCN2/CEBPA/COL1A1/CXCL8/HAMP/LTA*	22

ID	Description	GeneRatio	BgRatio	pvalue	p.adjust	qvalue	geneID	Count
GO:0072577	endothelial cell apoptotic process	11/293	65/18670	4,98E-09	6,41E-08	2,82E-08	*GATA2/TNF/FGG/FGB/FGA/SERPINE1/HLA-G/FASLG/KDR/IL4/IL10*	11
GO:1901214	regulation of neuron death	22/293	313/18670	5,11E-09	6,57E-08	2,89E-08	*TP53/AKT1/STAT3/TLR4/CASP3/MTOR/BAD/IKBKG/JAK2/TNF/HIF1A/CASP9/BAX/BCL2/BCL2L1/APOE/C5AR1/CEBPB/CREB1/FASLG/IFNG/IL10*	22
GO:0046546	development of primary male sexual characteristics	15/293	139/18670	5,15E-09	6,59E-08	2,90E-08	*AR/GATA1/ESR1/TGFBR1/REN/FLNA/BAX/BCL2/BCL2L1/INHA/BRIP1/LHB/FSHB/FSHR/INSR*	15
GO:0032635	interleukin-6 production	16/293	161/18670	5,26E-09	6,72E-08	2,96E-08	*AIF1/STAT3/TLR4/IL1B/TNF/IL6/HAVCR2/HMGB1/CEBPB/FOXP3/IL1RN/LEP/NOS2/TLR1/IL10/IL6R*	16
GO:0090276	regulation of peptide hormone secretion	18/293	208/18670	5,29E-09	6,74E-08	2,97E-08	*EGFR/BAD/JAK2/ADRA2A/IL1B/TNF/FGG/RBP4/HIF1A/HNF4A/FGB/FGA/CFTR/INS/IFNG/HFE/LEP/NOS2*	18
GO:0050999	regulation of nitric-oxide synthase activity	10/293	51/18670	5,63E-09	7,15E-08	3,15E-08	*AKT1/EGFR/NOS3/IL1B/TNF/HIF1A/APOE/DHFR/INS/LEP*	10
GO:0051701	interaction with host	18/293	209/18670	5,71E-09	7,23E-08	3,19E-08	*ACE2/SRC/EGFR/BAD/HSPA1B/NCAM1/HSPA1A/CCR5/BCL2L1/TYMS/ITGB6/ITGB3/ITGA2/TGFB1/CXCL8/EIF2AK2/INSR/TFRC*	18
GO:0002825	regulation of T-helper 1 type immune response	8/293	27/18670	5,73E-09	7,24E-08	3,19E-08	*IL1B/IL1R1/HAVCR2/SLC11A1/IL12B/IL23R/IL4R/IL6R*	8
GO:0045428	regulation of nitric oxide biosynthetic process	11/293	66/18670	5,89E-09	7,42E-08	3,27E-08	*AIF1/AKT1/TLR4/MTOR/JAK2/IL1B/TNF/AGT/INSR/IFNG/IL10*	11
GO:0051346	negative regulation of hydrolase activity	27/293	466/18670	5,92E-09	7,44E-08	3,28E-08	*TP53/SRC/AKT1/MDM2/NOS3/LGALS3/TNF/AGT/HRG/SERPINA10/SERPINA6/SERPINC1/LEPR/FETUB/SERPINA1/MAP2/TIMP1/TIMP3/SERPINE1/MMP9/LTF/VEGFA/TIMP4/TIMP2/COL6A3/IFI6/TFPI*	27
GO:0006898	receptor-mediated endocytosis	22/293	316/18670	6,09E-09	7,63E-08	3,36E-08	*ADRB2/CD163/APOB/APOE/ITGB3/SERPINE1/CD14/LILRB1/CD9/CEACAM1/VEGFA/CXCL8/CXCR1/EGF/INSR/FMR1/HBA1/HP/HFE/TFRC/IGF2R/IL4*	22
GO:0097529	myeloid leukocyte migration	18/293	210/18670	6,16E-09	7,70E-08	3,39E-08	*AIF1/LGALS3/IL1B/IL6/IL1R1/SERPINE1/HMGB1/C5AR1/CD9/VEGFA/CXCL8/CXCR1/VEGFC/FLT1/VEGFB/PGF/IL1RN/IL6R*	18
GO:1903426	regulation of reactive oxygen species biosynthetic process	13/293	101/18670	6,38E-09	7,94E-08	3,50E-08	*AIF1/AKT1/STAT3/TLR4/MTOR/JAK2/IL1B/TNF/AGT/INSR/INS/IFNG/IL10*	13
GO:0045913	positive regulation of carbohydrate metabolic process	12/293	83/18670	6,40E-09	7,94E-08	3,50E-08	*NFKB1/SRC/AKT1/BAD/ARNT/HIF1A/EGF/INSR/INS/IGF2/IFNG/IGF1*	12
GO:0048145	regulation of fibroblast proliferation	12/293	83/18670	6,40E-09	7,94E-08	3,50E-08	*TP53/EGFR/ESR1/PPARG/FN1/GSTP1/AGT/BAX/CREB1/TGFB1/IGF1/LTA*	12
GO:0035821	modification of morphology or	16/293	164/18670	6,88E-09	8,51E-08	3,75E-08	*BAD/F2/HRG/MBL2/BCL2L1/TYMS/APOE/LTF/TGFB1/EIF2AK2/INSR/FMR1/IFNG/HAMP/IGF2R/NOS2*	16

ID	Description	GeneRatio	BgRatio	pvalue	p.adjust	qvalue	geneID	Count
	physiology of other organism							
GO:0010675	regulation of cellular carbohydrate metabolic process	15/293	142/18670	6,91E-09	8,53E-08	3,76E-08	*TP53/SRC/AKT1/STAT3/MTOR/BAD/IGFBP3/LEPR/LCMT1/IGFBP4/INSR/INS/IGF2/IGF1/LEP*	15
GO:0002718	regulation of cytokine production involved in immune response	12/293	84/18670	7,36E-09	8,99E-08	3,96E-08	*TLR4/IL1B/TNF/IL6/IL1R1/HLA-E/HLA-G/LILRB1/TGFB1/FOXP3/HFE/IL10*	12
GO:0014910	regulation of smooth muscle cell migration	12/293	84/18670	7,36E-09	8,99E-08	3,96E-08	*ACE/AIF1/SRC/MDM2/IGFBP3/GSTP1/AGT/PRKG1/BCL2/SERPINE1/ITGA2/IGF1*	12
GO:0046889	positive regulation of lipid biosynthetic process	12/293	84/18670	7,36E-09	8,99E-08	3,96E-08	*AKT1/MTOR/TNFRSF1A/IL1B/TNF/APOE/CREB1/CGA/FSHB/INS/FGF1/IFNG*	12
GO:0048144	fibroblast proliferation	12/293	84/18670	7,36E-09	8,99E-08	3,96E-08	*TP53/EGFR/ESR1/PPARG/FN1/GSTP1/AGT/BAX/CREB1/TGFB1/IGF1/LTA*	12
GO:0032770	positive regulation of monooxygenase activity	8/293	28/18670	7,92E-09	9,64E-08	4,25E-08	*AKT1/IL1B/TNF/HIF1A/APOE/DHFR/INS/IFNG*	8
GO:1902106	negative regulation of leukocyte differentiation	13/293	103/18670	8,14E-09	9,89E-08	4,36E-08	*TLR4/GATA2/INHA/HMGB1/LTF/HLA-G/LILRB1/CEACAM1/FBN1/CTLA4/FOXP3/IL4R/IL4*	13
GO:0031343	positive regulation of cell killing	11/293	68/18670	8,17E-09	9,90E-08	4,36E-08	*BAD/HLA-E/HLA-C/HLA-G/HLA-A/HLA-B/IL12B/IL23R/IFNG/IL12A/NOS2*	11
GO:0060759	regulation of response to cytokine stimulus	17/293	190/18670	8,61E-09	1,04E-07	4,58E-08	*TRAF1/TLR4/CHUK/IKBKG/TNFRSF1A/HSPA1B/HSPA1A/PPARG/JAK2/GSTP1/TNF/IL6/IL1R1/HIF1A/MMP12/IFNG/IL1RN*	17
GO:0042176	regulation of protein catabolic process	24/293	381/18670	8,69E-09	1,05E-07	4,62E-08	*AKT1/EGFR/MDM2/HSPA1B/HSPA1A/ADRA2A/IL1B/TNF/FLNA/APC/APOE/TIMP1/TIMP3/BAG6/CEBPA/TIMP4/TIMP2/EGF/INS/FMR1/IFNG/HFE/NOS2/IL10*	24
GO:0002369	T cell cytokine production	9/293	40/18670	9,12E-09	1,10E-07	4,83E-08	*IL1B/IL6/IL1R1/SLC11A1/IL12B/FOXP3/HFE/IL12A/IL4*	9
GO:0008630	intrinsic apoptotic signaling pathway in response to DNA damage	13/293	104/18670	9,17E-09	1,10E-07	4,85E-08	*TP53/BAD/TNFRSF1A/TNF/TP63/TP73/CASP9/BAX/BCL2/BCL2L1/BAG6/BRCA1/BRCA2*	13
GO:0002363	alpha-beta T cell lineage commitment	7/293	19/18670	9,36E-09	1,12E-07	4,93E-08	*STAT3/MTOR/IL6/BCL2/IL12B/IL23R/FOXP3*	7
GO:0042698	ovulation cycle	11/293	69/18670	9,59E-09	1,14E-07	5,04E-08	*SRC/EGFR/CASP3/NOS3/ESR1/FSHB/FSHR/MMP7/TIMP4/PGR/LEP*	11
GO:1903829	positive regulation of cellular protein localization	22/293	324/18670	9,61E-09	1,14E-07	5,04E-08	*HSPA1L/TP53/SRC/AKT1/EGFR/MDM2/BAD/LGALS3/RAN/IL1B/TNF/TP63/TP73/F2/FLNA/BCL2/APC/TGFB1/EGF/INS/IFNG/LEP*	22

ID	Description	GeneRatio	BgRatio	pvalue	p.adjust	qvalue	geneID	Count
GO:0051353	positive regulation of oxidoreductase activity	10/293	54/18670	1,01E-08	1,20E-07	5,29E-08	*AKT1/IL1B/AGTR1/TNF/AGT/HIF1A/APOE/DHFR/INS/IFNG*	10
GO:0051384	response to glucocorticoid	15/293	146/18670	1,01E-08	1,20E-07	5,29E-08	*AIF1/NR3C1/EGFR/CASP3/BAD/GSTP1/TNF/IL6/CASP9/BCL2/TYMS/TGFB1/PAPPA/IL1RN/IL10*	15
GO:0045165	cell fate commitment	20/293	270/18670	1,08E-08	1,27E-07	5,60E-08	*TP53/STAT3/CASP3/GATA2/MTOR/AR/NR2F2/GATA1/FGFR1/PPARG/ACVR1/TGFBR1/BMP2/IL6/BCL2/APC/CEBPB/IL12B/IL23R/FOXP3*	20
GO:0007263	nitric oxide mediated signal transduction	8/293	29/18670	1,08E-08	1,27E-07	5,60E-08	*EGFR/NOS3/AGT/APOE/VEGFA/INS/NOS2/PDE5A*	8
GO:1902893	regulation of pri-miRNA transcription by RNA polymerase II	9/293	41/18670	1,15E-08	1,35E-07	5,97E-08	*TP53/STAT3/NR3C1/GATA2/BMP2/HIF1A/PRL/TGFB1/IL10*	9
GO:0016485	protein processing	22/293	328/18670	1,20E-08	1,41E-07	6,21E-08	*ACE/ACE2/SRC/MDM2/CASP3/BAD/IL1B/REN/FGG/F2/CPB2/F12/FGB/F7/FGA/PLAT/SERPINE1/C5AR1/CGA/LHB/FSHB/F3*	22
GO:0001541	ovarian follicle development	10/293	55/18670	1,22E-08	1,43E-07	6,28E-08	*SRC/ESR1/BAX/BCL2/BCL2L1/INHA/CEBPB/VEGFA/FSHB/FSHR*	10
GO:0043542	endothelial cell migration	20/293	273/18670	1,30E-08	1,51E-07	6,66E-08	*AKT1/NOS3/GATA2/NR2F2/FGFR1/PPARG/TGFBR1/AGT/HRG/HIF1A/APOE/ITGB3/HMGB1/CEACAM1/VEGFA/TGFB1/EPHB4/FGF1/KDR/VEGFC*	20
GO:0043369	CD4-positive or CD8-positive, alpha-beta T cell lineage commitment	7/293	20/18670	1,42E-08	1,65E-07	7,29E-08	*STAT3/MTOR/IL6/BCL2/IL12B/IL23R/FOXP3*	7
GO:0097421	liver regeneration	8/293	30/18670	1,45E-08	1,68E-07	7,42E-08	*EGFR/TYMS/CPB2/CEBPB/TGFB1/HAMP/HFE/IL10*	8
GO:0032615	interleukin-12 production	10/293	56/18670	1,46E-08	1,69E-07	7,45E-08	*NFKB1/TLR4/HMGB1/HLA-G/LILRB1/IL12B/IL23R/IFNG/LEP/IL10*	10
GO:0060688	regulation of morphogenesis of a branching structure	10/293	56/18670	1,46E-08	1,69E-07	7,45E-08	*AR/FGFR1/ESR1/TNF/AGT/VEGFA/TGFB1/SULF1/FGF7/PGF*	10
GO:0061180	mammary gland epithelium development	11/293	72/18670	1,52E-08	1,76E-07	7,74E-08	*SRC/AKT1/AR/ESR1/JAK2/HIF1A/BAX/BRCA2/CEBPB/TGFB1/PGR*	11
GO:0002695	negative regulation of leukocyte activation	16/293	175/18670	1,75E-08	1,99E-07	8,78E-08	*CASP3/LGALS3/INHA/HAVCR2/HMGB1/HLA-G/LILRB1/CEACAM1/CEBPB/TGFB1/CTLA4/FOXP3/HFE/IL4R/PDE5A/IL10*	16
GO:0046165	alcohol biosynthetic process	16/293	175/18670	1,75E-08	1,99E-07	8,78E-08	*NFKB1/BMP2/RAN/IL1B/TNF/APOB/APOE/NSDHL/CFTR/CNBP/DHCR7/DHFR/EBP/FGF1/IFNG/LEP*	16
GO:0045981	positive regulation of nucleotide metabolic process	10/293	57/18670	1,75E-08	1,99E-07	8,78E-08	*STAT3/NOS3/ARNT/HIF1A/INSR/INS/IFNG/IGF1/IL4/NOS2*	10

ID	Description	GeneRatio	BgRatio	pvalue	p.adjust	qvalue	geneID	Count
GO:1900544	positive regulation of purine nucleotide metabolic process	10/293	57/18670	1,75E-08	1,99E-07	8,78E-08	*STAT3/NOS3/ARNT/HIF1A/INSR/INS/IFNG/IGF1/IL4/NOS2*	10
GO:1903428	positive regulation of reactive oxygen species biosynthetic process	10/293	57/18670	1,75E-08	1,99E-07	8,78E-08	*AIF1/AKT1/TLR4/MTOR/JAK2/IL1B/TNF/AGT/INSR/IFNG*	10
GO:0032881	regulation of polysaccharide metabolic process	9/293	43/18670	1,80E-08	2,05E-07	9,03E-08	*NFKB1/AKT1/MTOR/TGFB1/EGF/INSR/INS/IGF2/IGF1*	9
GO:0007160	cell-matrix adhesion	18/293	225/18670	1,82E-08	2,06E-07	9,07E-08	*SRC/FN1/L1CAM/FGG/HRG/BCL2/FGB/FGA/MMP12/ITGB4/ITGB6/ITGA2B/ITGB3/SERPINE1/ITGA2/CEACAM6/VEGFA/KDR*	18
GO:0048568	embryonic organ development	25/293	428/18670	1,90E-08	2,14E-07	9,45E-08	*TP53/AKT1/EGFR/GATA2/NR2F2/GATA1/FGFR1/ACVR1/TGFBR1/ADA/TNF/RBP4/ARNT/HIF1A/NSDHL/CEBPA/CEBPB/VEGFA/TGFB1/FBN1/COL5A2/CXCL8/IGF2/KDR/IL10*	25
GO:0042133	neurotransmitter metabolic process	15/293	153/18670	1,92E-08	2,17E-07	9,54E-08	*AIF1/AKT1/TLR4/NOS3/MTOR/JAK2/IL1B/TNF/AGT/PAH/HTR1A/INSR/IFNG/NOS2/IL10*	15
GO:0045648	positive regulation of erythrocyte differentiation	8/293	31/18670	1,93E-08	2,17E-07	9,56E-08	*STAT3/STAT5B/GATA2/GATA1/HSPA1B/HSPA1A/ARNT/HIF1A*	8
GO:1903036	positive regulation of response to wounding	11/293	74/18670	2,05E-08	2,30E-07	1,01E-07	*TLR4/MTOR/ADRA2A/F2/HRG/CPB2/F12/F7/SERPINE1/THBD/F3*	11
GO:0001959	regulation of cytokine-mediated signaling pathway	16/293	177/18670	2,06E-08	2,30E-07	1,01E-07	*TRAF1/CHUK/IKBKG/TNFRSF1A/HSPA1B/HSPA1A/PPARG/JAK2/GSTP1/TNF/IL6/IL1R1/HIF1A/MMP12/IFNG/IL1RN*	16
GO:1902041	regulation of extrinsic apoptotic signaling pathway via death domain receptors	10/293	58/18670	2,09E-08	2,33E-07	1,03E-07	*NOS3/LGALS3/FGG/BCL2L1/FGB/FGA/TIMP3/SERPINE1/BRCA1/FASLG*	10
GO:0045995	regulation of embryonic development	14/293	132/18670	2,10E-08	2,35E-07	1,03E-07	*GATA2/AR/GATA1/FGFR1/HNF4A/BAG6/COL5A1/COL5A2/SULF1/INSR/IGF1/IL1RN/LAMA4/IL10*	14
GO:0010594	regulation of endothelial cell migration	18/293	229/18670	2,39E-08	2,65E-07	1,17E-07	*AKT1/NOS3/GATA2/NR2F2/FGFR1/PPARG/AGT/HRG/HIF1A/APOE/ITGB3/HMGB1/CEACAM1/VEGFA/TGFB1/FGF1/KDR/VEGFC*	18
GO:0046634	regulation of alpha-beta T cell activation	12/293	93/18670	2,39E-08	2,66E-07	1,17E-07	*ADA/HMGB1/HLA-E/LILRB1/IL12B/IL23R/FOXP3/IFNG/HFE/IL12A/IL4R/IL6R*	12
GO:0071347	cellular response to interleukin-1	16/293	179/18670	2,41E-08	2,67E-07	1,18E-07	*NFKB1/CHUK/IKBKG/IL1B/IL6/IL1R1/UBE2N/FGG/HIF1A/FGB/LCN2/CEBPB/CXCL8/TFPI/IL1RN/IL1A*	16

ID	Description	GeneRatio	BgRatio	pvalue	p.adjust	qvalue	geneID	Count
GO:0042130	negative regulation of T cell proliferation	10/293	59/18670	2,48E-08	2,72E-07	1,20E-07	*CASP3/HAVCR2/HLA-G/LILRB1/CEBPB/TGFB1/CTLA4/FOXP3/PDE5A/IL10*	10
GO:0070527	platelet aggregation	10/293	59/18670	2,48E-08	2,72E-07	1,20E-07	*GATA1/FGG/FLNA/PRKG1/FGB/FGA/ITGA2B/ITGB3/CD9/CEACAM1*	10
GO:2000351	regulation of endothelial cell apoptotic process	10/293	59/18670	2,48E-08	2,72E-07	1,20E-07	*GATA2/TNF/FGG/FGB/FGA/SERPINE1/HLA-G/FASLG/KDR/IL4*	10
GO:0021700	developmental maturation	20/293	284/18670	2,52E-08	2,76E-07	1,22E-07	*GATA2/MTOR/FGFR1/PPARG/BMP2/REN/FGG/HIF1A/MMP2/BCL2/TYMS/BRCA2/LTF/CEBPA/VEGFA/CFTR/PGR/FGFR3/IGF1/LEP*	20
GO:0043254	regulation of protein complex assembly	26/293	467/18670	2,58E-08	2,82E-07	1,24E-07	*TP53/SRC/TLR4/MTOR/ESR1/HSPA1B/HSPA1A/TNF/HRG/LCMT1/BAX/APOE/MAP2/MMP3/GTF2H4/HMGB1/MMP1/VEGFA/CREB1/CNOT1/TGFB1/EIF2AK2/INS/IFNG/HNF1A/MAPRE3*	26
GO:0002688	regulation of leukocyte chemotaxis	13/293	114/18670	2,83E-08	3,07E-07	1,35E-07	*AIF1/IL6/F7/SERPINE1/HMGB1/C5AR1/VEGFA/CXCL8/VEGFC/VEGFB/PGF/IL12A/IL6R*	13
GO:0030218	erythrocyte differentiation	13/293	114/18670	2,83E-08	3,07E-07	1,35E-07	*STAT3/STAT5B/CASP3/GATA2/GATA1/HSPA1B/HSPA1A/JAK2/ARNT/HIF1A/INHA/VEGFA/SLC4A1*	13
GO:0002440	production of molecular mediator of immune response	20/293	286/18670	2,83E-08	3,07E-07	1,35E-07	*TLR4/IL1B/TNF/IL6/IL1R1/RBP4/SLC11A1/HLA-E/HLA-G/LILRB1/TGFB1/IL12B/FOXP3/HFE/TFRC/HLA-DQB1/IL12A/IL4R/IL4/IL10*	20
GO:0019883	antigen processing and presentation of endogenous antigen	7/293	22/18670	3,04E-08	3,29E-07	1,45E-07	*HLA-E/HLA-C/HLA-G/HLA-A/HLA-B/TAP2/HFE*	7
GO:0008217	regulation of blood pressure	16/293	182/18670	3,05E-08	3,29E-07	1,45E-07	*ACE/ACE2/NOS3/AR/NR2F2/PPARG/ACTA2/ADRB2/AGTR1/AGT/REN/SGK1/COL1A2/VEGFC/LEP/NOS2*	16
GO:0002702	positive regulation of production of molecular mediator of immune response	12/293	95/18670	3,05E-08	3,29E-07	1,45E-07	*TLR4/IL1B/IL6/IL1R1/RBP4/HLA-E/HLA-G/LILRB1/TGFB1/TFRC/IL4R/IL4*	12
GO:0070555	response to interleukin-1	17/293	207/18670	3,10E-08	3,33E-07	1,47E-07	*NFKB1/SRC/CHUK/IKBKG/IL1B/IL6/IL1R1/UBE2N/FGG/HIF1A/FGB/LCN2/CEBPB/CXCL8/TFPI/IL1RN/IL1A*	17
GO:0097305	response to alcohol	18/293	233/18670	3,12E-08	3,35E-07	1,48E-07	*AKT1/STAT3/BAD/PPARG/GSTP1/TGFBR1/CCR5/RBP4/AHR/BCL2L1/TYMS/F7/BRCA1/CD14/CFTR/TGFB1/HAMP/LEP*	18
GO:0048010	vascular endothelial growth factor receptor signaling pathway	12/293	96/18670	3,44E-08	3,69E-07	1,63E-07	*SRC/IL1B/ARNT/HIF1A/ITGB3/VEGFA/SULF1/KDR/VEGFC/FLT1/VEGFB/PGF*	12
GO:0030888	regulation of B cell proliferation	10/293	61/18670	3,46E-08	3,70E-07	1,63E-07	*TLR4/CASP3/CD320/ADA/AHR/BCL2/CTLA4/TFRC/IL4/IL10*	10
GO:0050769	positive regulation of neurogenesis	26/293	474/18670	3,48E-08	3,71E-07	1,64E-07	*TRPC6/GATA2/MTOR/FGFR1/PPARG/FN1/L1CAM/BMP2/IL1B/TNF/IL6/TP73/AGT/HIF1A/FLNA/BCL2/APOE/VEG*	26

ID	Description	GeneRatio	BgRatio	pvalue	p.adjust	qvalue	geneID	Count
							FA/CRTC1/TGFB1/TIMP2/DICER1/VEGFC/FMR1/IFNG/LTA	
GO:0021782	glial cell development	13/293	116/18670	3,49E-08	3,71E-07	1,64E-07	*AKT1/TLR4/EGFR/GSTP1/IL1B/TNF/IL6/C5AR1/CD9/TGFB1/DICER1/IFNG/ROR1*	13
GO:0046661	male sex differentiation	15/293	160/18670	3,52E-08	3,73E-07	1,64E-07	*AR/GATA1/ESR1/TGFBR1/REN/FLNA/BAX/BCL2/BCL2L1/INHA/BRIP1/LHB/FSHB/FSHR/INSR*	15
GO:0046777	protein autophosphorylation	18/293	235/18670	3,56E-08	3,76E-07	1,66E-07	*PIM1/ACE/SRC/AKT1/EGFR/MTOR/FGFR1/IGF1R/JAK2/VEGFA/EIF2AK2/EPHB4/INSR/INS/FGFR3/KDR/VEGFC/FLT1*	18
GO:0043401	steroid hormone mediated signaling pathway	16/293	184/18670	3,56E-08	3,76E-07	1,66E-07	*SRC/THRB/NR3C1/ESR2/AR/NR2F2/ESR1/PPARG/JAK2/RAN/TP63/HNF4A/BRCA1/CNOT1/RXRB/PGR*	16
GO:0070664	negative regulation of leukocyte proliferation	11/293	78/18670	3,61E-08	3,81E-07	1,68E-07	*CASP3/GSTP1/HAVCR2/HLA-G/LILRB1/CEBPB/TGFB1/CTLA4/FOXP3/PDE5A/IL10*	11
GO:0002834	regulation of response to tumor cell	6/293	14/18670	3,84E-08	4,01E-07	1,77E-07	*HRG/HAVCR2/HMGB1/CEACAM1/IL12B/IL12A*	6
GO:0002837	regulation of immune response to tumor cell	6/293	14/18670	3,84E-08	4,01E-07	1,77E-07	*HRG/HAVCR2/HMGB1/CEACAM1/IL12B/IL12A*	6
GO:0050930	induction of positive chemotaxis	6/293	14/18670	3,84E-08	4,01E-07	1,77E-07	*IL16/VEGFA/CXCL8/VEGFC/VEGFB/PGF*	6
GO:0051917	regulation of fibrinolysis	6/293	14/18670	3,84E-08	4,01E-07	1,77E-07	*F2/HRG/CPB2/F12/SERPINE1/THBD*	6
GO:0007369	gastrulation	16/293	185/18670	3,84E-08	4,01E-07	1,77E-07	*TP53/FGFR1/FN1/ACVR1/HNF4A/MMP2/ITGB4/ITGB3/MMP9/ITGA2/COL5A1/COL5A2/COL6A1/IL1RN/MMP15/IL10*	16
GO:0120162	positive regulation of cold-induced thermogenesis	12/293	97/18670	3,87E-08	4,03E-07	1,78E-07	*IGF1R/JAK2/ADRB2/LEPR/PRLR/APC/LCN2/CEBPB/VEGFA/IL4R/IL4/LEP*	12
GO:0045089	positive regulation of innate immune response	23/293	381/18670	3,94E-08	4,09E-07	1,80E-07	*NFKB1/SRC/TLR4/CHUK/ESR1/IKBKG/HSPA1B/HSPA1A/UBE2N/FGG/FGB/FGA/APOB/MMP12/HAVCR2/HMGB1/CD14/LTF/HLA-E/HLA-G/IL12B/IL12A/TLR1*	23
GO:0031330	negative regulation of cellular catabolic process	19/293	264/18670	4,01E-08	4,16E-07	1,83E-07	*TP53/AKT1/STAT3/MTOR/IL10RA/LEPR/BCL2/TIMP1/TIMP3/BAG6/SLC11A1/TIMP4/TIMP2/INS/FMR1/HP/HFE/LEP/IL10*	19
GO:0032148	activation of protein kinase B activity	8/293	34/18670	4,26E-08	4,40E-07	1,94E-07	*SRC/AKT1/MTOR/ADRA2A/INSR/INS/FGF1/IGF1*	8
GO:0032660	regulation of interleukin-17 production	8/293	34/18670	4,26E-08	4,40E-07	1,94E-07	*TLR4/IL6/TGFB1/IL12B/IL23R/FOXP3/IFNG/IL12A*	8
GO:0031589	cell-substrate adhesion	22/293	354/18670	4,71E-08	4,85E-07	2,14E-07	*SRC/JAK2/FN1/L1CAM/FGG/HRG/FLNA/BCL2/FGB/FGA/MMP12/ITGB4/ITGB6/ITGA2B/ITGB3/SERPINE1/ITGA2/CEACAM6/VEGFA/COL1A1/FBLN1/KDR*	22

ID	Description	GeneRatio	BgRatio	pvalue	p.adjust	qvalue	geneID	Count
GO:0002637	regulation of immunoglobulin production	10/293	63/18670	4,77E-08	4,90E-07	2,16E-07	*TNF/IL6/RBP4/HLA-E/TGFB1/FOXP3/TFRC/IL4R/IL4/IL10*	10
GO:0007088	regulation of mitotic nuclear division	15/293	164/18670	4,90E-08	5,02E-07	2,21E-07	*HSPA1B/HSPA1A/IL1B/LCMT1/APC/BUB1/BUB1B/BUB3/TGFB1/EGF/INSR/INS/IGF2/IGF1/IL1A*	15
GO:0002698	negative regulation of immune effector process	13/293	120/18670	5,25E-08	5,37E-07	2,37E-07	*LGALS3/TNF/HAVCR2/HLA-E/HLA-G/LILRB1/CEACAM1/TGFB1/INS/FOXP3/HFE/IL4R/IL10*	13
GO:1903522	regulation of blood circulation	20/293	297/18670	5,29E-08	5,40E-07	2,38E-07	*ACE/ACE2/AKT1/THRB/EGFR/MDM2/NOS3/JAK2/ADA/ADRA2A/AGTR1/AGT/FGG/FLNA/FGB/FGA/DES/LEP/TRPC1/PDE5A*	20
GO:0034109	homotypic cell-cell adhesion	11/293	81/18670	5,40E-08	5,48E-07	2,42E-07	*GATA1/FGG/FLNA/PRKG1/FGB/FGA/ITGA2B/ITGB3/CD9/CEACAM1/CEACAM5*	11
GO:0048708	astrocyte differentiation	11/293	81/18670	5,40E-08	5,48E-07	2,42E-07	*STAT3/TLR4/EGFR/BMP2/IL1B/TNF/IL6/F2/C5AR1/IFNG/ROR1*	11
GO:0070374	positive regulation of ERK1 and ERK2 cascade	17/293	215/18670	5,41E-08	5,48E-07	2,42E-07	*SRC/TLR4/EGFR/BMP2/TNF/FGG/FGB/FGA/APOE/HAVCR2/HMGB1/C5AR1/FSHR/TGFB1/FGFR3/KDR/FLT1*	17
GO:0045927	positive regulation of growth	19/293	270/18670	5,74E-08	5,81E-07	2,56E-07	*PIM1/AKT1/EGFR/MTOR/FGFR1/FN1/L1CAM/TGFBR1/F2/IGFBP1/BCL2/VEGFA/CREB1/INSR/INS/IGF2/HAMP/IGF1/LEP*	19
GO:0045787	positive regulation of cell cycle	23/293	389/18670	5,76E-08	5,81E-07	2,56E-07	*TP53/AIF1/SRC/AKT1/EGFR/MDM2/FGFR1/IL1B/TP73/BAX/SPAG5/BRCA1/BRCA2/CNOT1/TGFB1/EGF/INSR/INS/IGF2/IGF1/IL1A/MAPRE3/IL10*	23
GO:0106106	cold-induced thermogenesis	14/293	143/18670	5,85E-08	5,89E-07	2,59E-07	*TLR4/IGF1R/JAK2/ADRB2/LEPR/PRLR/APC/LCN2/CEBPB/VEGFA/IL4R/IL4/LEP/LAMA4*	14
GO:0120161	regulation of cold-induced thermogenesis	14/293	143/18670	5,85E-08	5,89E-07	2,59E-07	*TLR4/IGF1R/JAK2/ADRB2/LEPR/PRLR/APC/LCN2/CEBPB/VEGFA/IL4R/IL4/LEP/LAMA4*	14
GO:1901617	organic hydroxy compound biosynthetic process	19/293	271/18670	6,09E-08	6,10E-07	2,69E-07	*NFKB1/TP53/BMP2/RAN/IL1B/TNF/PAH/APOB/APOE/NSDHL/CFTR/CNBP/DHCR7/DHFR/EBP/FGF1/IFNG/LEP/TRPC1*	19
GO:0032637	interleukin-8 production	11/293	82/18670	6,15E-08	6,15E-07	2,71E-07	*TLR4/HSPA1B/HSPA1A/IL1B/TNF/SERPINE1/CD14/LEP/NOS2/TLR1/IL10*	11
GO:0045342	MHC class II biosynthetic process	6/293	15/18670	6,31E-08	6,30E-07	2,78E-07	*TLR4/JAK2/SLC11A1/IFNG/IL4/IL10*	6
GO:1902107	positive regulation of leukocyte differentiation	14/293	144/18670	6,39E-08	6,37E-07	2,81E-07	*BAD/ADA/TNF/HMGB1/HLA-G/CREB1/TGFB1/IL12B/IL23R/FOXP3/IFNG/IL12A/IL4R/IL4*	14
GO:0034101	erythrocyte homeostasis	13/293	122/18670	6,40E-08	6,37E-07	2,81E-07	*STAT3/STAT5B/CASP3/GATA2/GATA1/HSPA1B/HSPA1A/JAK2/ARNT/HIF1A/INHA/VEGFA/SLC4A1*	13
GO:0034614	cellular response to reactive oxygen species	15/293	168/18670	6,75E-08	6,70E-07	2,95E-07	*SRC/AKT1/TRPC6/EGFR/MDM2/NOS3/CHUK/TNF/IL6/MMP2/MMP3/MMP9/LCN2/DHFR/IL10*	15

ID	Description	GeneRatio	BgRatio	pvalue	p.adjust	qvalue	geneID	Count
GO:2001171	positive regulation of ATP biosynthetic process	8/293	36/18670	6,91E-08	6,85E-07	3,02E-07	*STAT3/ARNT/HIF1A/INSR/INS/IFNG/IGF1/IL4*	8
GO:0045582	positive regulation of T cell differentiation	11/293	83/18670	6,99E-08	6,91E-07	3,04E-07	*BAD/ADA/HLA-G/TGFB1/IL12B/IL23R/FOXP3/IFNG/IL12A/IL4R/IL4*	11
GO:0006066	alcohol metabolic process	22/293	364/18670	7,69E-08	7,59E-07	3,34E-07	*NFKB1/FGFR1/BMP2/RAN/IL1B/TNF/RBP4/LEPR/APOB/APOE/NSDHL/CEBPA/CFTR/CNBP/DHCR7/DHFR/EBP/FGF1/IFNG/IGF1/IL4/LEP*	22
GO:0031639	plasminogen activation	7/293	25/18670	8,24E-08	8,11E-07	3,57E-07	*FGG/CPB2/F12/FGB/FGA/PLAT/SERPINE1*	7
GO:0051604	protein maturation	23/293	397/18670	8,33E-08	8,18E-07	3,61E-07	*ACE/ACE2/SRC/MDM2/CASP3/BAD/IL1B/REN/FGG/F2/CPB2/F12/FGB/F7/FGA/PLAT/SERPINE1/C5AR1/CGA/LHB/FSHB/F3/ISCA2*	23
GO:0046006	regulation of activated T cell proliferation	8/293	37/18670	8,70E-08	8,53E-07	3,76E-07	*CASP3/HMGB1/IL12B/IGF2/IL23R/FOXP3/IGF1/IL6R*	8
GO:0042108	positive regulation of cytokine biosynthetic process	10/293	67/18670	8,76E-08	8,57E-07	3,77E-07	*STAT3/TLR4/JAK2/IL1B/TNF/IL6/IL12B/IFNG/IL1A/TLR1*	10
GO:1904705	regulation of vascular smooth muscle cell proliferation	11/293	85/18670	8,98E-08	8,75E-07	3,86E-07	*MDM2/PPARG/JAK2/GSTP1/TNF/AGT/PRKG1/MMP2/MMP9/IGF1/IL10*	11
GO:1990874	vascular smooth muscle cell proliferation	11/293	85/18670	8,98E-08	8,75E-07	3,86E-07	*MDM2/PPARG/JAK2/GSTP1/TNF/AGT/PRKG1/MMP2/MMP9/IGF1/IL10*	11
GO:0030324	lung development	15/293	172/18670	9,23E-08	8,97E-07	3,95E-07	*THRB/EGFR/NOS3/FGFR1/ADA/TNF/RBP4/BAG6/CEBPA/VEGFA/CREB1/DHCR7/PGR/FGF1/FGF7*	15
GO:0002221	pattern recognition receptor signaling pathway	16/293	197/18670	9,25E-08	8,98E-07	3,96E-07	*TLR4/CHUK/ESR1/IKBKG/HSPA1B/HSPA1A/UBE2N/FGG/FGB/FGA/APOB/HAVCR2/HMGB1/CD14/LTF/TLR1*	16
GO:0072376	protein activation cascade	16/293	198/18670	9,93E-08	9,61E-07	4,24E-07	*IL1B/FGG/F2/MBL2/SERPINC1/CPB2/F12/FGB/F7/FGA/C5AR1/F13A1/FBLN1/F10/TFPI/F3*	16
GO:0002475	antigen processing and presentation via MHC class Ib	6/293	16/18670	9,96E-08	9,62E-07	4,24E-07	*HLA-E/HLA-C/HLA-G/HLA-A/HLA-B/TAP2*	6
GO:0071230	cellular response to amino acid stimulus	10/293	68/18670	1,01E-07	9,75E-07	4,30E-07	*EGFR/MTOR/TNF/MMP2/BCL2L1/CEBPB/COL1A1/COL5A2/COL6A1/COL1A2*	10
GO:0001776	leukocyte homeostasis	11/293	86/18670	1,02E-07	9,75E-07	4,30E-07	*AKT1/CASP3/ADA/IL6/HIF1A/BAX/BCL2/HMGB1/TGFB1/FOXP3/TSC22D3*	11
GO:0042446	hormone biosynthetic process	11/293	86/18670	1,02E-07	9,75E-07	4,30E-07	*NFKB1/BMP2/IL1B/TNF/ARNT/HIF1A/LHB/FSHB/DHCR7/IFNG/HFE*	11
GO:0048754	branching morphogenesis of an epithelial tube	14/293	150/18670	1,07E-07	1,03E-06	4,52E-07	*SRC/AR/ESR1/ACVR1/BMP2/TNF/AGT/BCL2/VEGFA/TGFB1/EGF/PGR/FGF1/PGF*	14

ID	Description	GeneRatio	BgRatio	pvalue	p.adjust	qvalue	geneID	Count
GO:0032620	interleukin-17 production	8/293	38/18670	1,09E-07	1,04E-06	4,58E-07	*TLR4/IL6/TGFB1/IL12B/IL23R/FOXP3/IFNG/IL12A*	8
GO:0043567	regulation of insulin-like growth factor receptor signaling pathway	7/293	26/18670	1,11E-07	1,06E-06	4,68E-07	*AR/IGFBP3/BMP2/IGFBP1/IGFBP4/IGF1/IGFBP6*	7
GO:0010595	positive regulation of endothelial cell migration	13/293	128/18670	1,13E-07	1,08E-06	4,76E-07	*AKT1/NOS3/GATA2/FGFR1/AGT/HIF1A/ITGB3/HMGB1/VEGFA/TGFB1/FGF1/KDR/VEGFC*	13
GO:0001892	embryonic placenta development	11/293	87/18670	1,15E-07	1,09E-06	4,80E-07	*AKT1/EGFR/GATA2/NR2F2/ARNT/HIF1A/NSDHL/CEBPA/CEBPB/IGF2/IL10*	11
GO:0002478	antigen processing and presentation of exogenous peptide antigen	15/293	175/18670	1,16E-07	1,10E-06	4,85E-07	*CHUK/IKBKG/HLA-E/HLA-C/HLA-G/HLA-A/HLA-B/TAP2/HLA-DQA1/HLA-DRB5/HLA-DRA/HLA-DQB2/HLA-DRB1/HLA-DQB1/HLA-DQA2*	15
GO:0030323	respiratory tube development	15/293	176/18670	1,25E-07	1,18E-06	5,21E-07	*THRB/EGFR/NOS3/FGFR1/ADA/TNF/RBP4/BAG6/CEBPA/VEGFA/CREB1/DHCR7/PGR/FGF1/FGF7*	15
GO:0001890	placenta development	14/293	152/18670	1,26E-07	1,19E-06	5,25E-07	*AKT1/EGFR/GATA2/NR2F2/PPARG/ADA/ARNT/HIF1A/NSDHL/CEBPA/CEBPB/IGF2/LEP/IL10*	14
GO:0032675	regulation of interleukin-6 production	14/293	152/18670	1,26E-07	1,19E-06	5,25E-07	*AIF1/STAT3/TLR4/IL1B/TNF/IL6/HAVCR2/HMGB1/CEBPB/FOXP3/IL1RN/TLR1/IL10/IL6R*	14
GO:0010721	negative regulation of cell development	21/293	344/18670	1,30E-07	1,22E-06	5,38E-07	*TP53/STAT3/TRPC6/THRB/MDM2/IL1B/TNF/IL6/TP73/F2/BCL2/APOE/MAP2/LTF/LILRB1/VEGFA/TGFB1/FBLN1/FBN1/DICER1/IGF1*	21
GO:0050852	T cell receptor signaling pathway	16/293	202/18670	1,31E-07	1,23E-06	5,42E-07	*NFKB1/CHUK/IKBKG/LGALS3/ADA/UBE2N/CEACAM1/CTLA4/FOXP3/HLA-DQA1/HLA-DRB5/HLA-DRA/HLA-DQB2/HLA-DRB1/HLA-DQB1/HLA-DQA2*	16
GO:1902042	negative regulation of extrinsic apoptotic signaling pathway via death domain receptors	8/293	39/18670	1,35E-07	1,26E-06	5,57E-07	*NOS3/FGG/BCL2L1/FGB/FGA/SERPINE1/BRCA1/FASLG*	8
GO:0032655	regulation of interleukin-12 production	9/293	54/18670	1,46E-07	1,37E-06	6,03E-07	*NFKB1/TLR4/HMGB1/HLA-G/LILRB1/IL12B/IL23R/IFNG/IL10*	9
GO:0046885	regulation of hormone biosynthetic process	7/293	27/18670	1,48E-07	1,38E-06	6,09E-07	*NFKB1/BMP2/IL1B/TNF/ARNT/HIF1A/IFNG*	7
GO:1990845	adaptive thermogenesis	14/293	154/18670	1,49E-07	1,39E-06	6,10E-07	*TLR4/IGF1R/JAK2/ADRB2/LEPR/PRLR/APC/LCN2/CEBPB/VEGFA/IL4R/IL4/LEP/LAMA4*	14
GO:0002418	immune response to tumor cell	6/293	17/18670	1,52E-07	1,41E-06	6,21E-07	*HRG/HAVCR2/HMGB1/CEACAM1/IL12B/IL12A*	6

ID	Description	GeneRatio	BgRatio	pvalue	p.adjust	qvalue	geneID	Count
GO:0043373	CD4-positive, alpha-beta T cell lineage commitment	6/293	17/18670	1,52E-07	1,41E-06	6,21E-07	*STAT3/MTOR/IL6/IL12B/IL23R/FOXP3*	6
GO:1902652	secondary alcohol metabolic process	14/293	155/18670	1,61E-07	1,49E-06	6,58E-07	*FGFR1/RAN/LEPR/APOB/APOE/NSDHL/CEBPA/CFTR/CNBP/DHCR7/EBP/FGF1/IL4/LEP*	14
GO:0045598	regulation of fat cell differentiation	13/293	132/18670	1,63E-07	1,51E-06	6,64E-07	*AKT1/GATA2/MTOR/PPARG/BMP2/TNF/IL6/CEBPA/CEBPB/CREB1/TGFB1/INS/LEP*	13
GO:0045778	positive regulation of ossification	11/293	90/18670	1,63E-07	1,51E-06	6,64E-07	*ACVR1/BMP2/ADRB2/IL6/TP63/LTF/CEBPA/CEBPB/TGFB1/IGF1/IL6R*	11
GO:1903556	negative regulation of tumor necrosis factor superfamily cytokine production	10/293	72/18670	1,77E-07	1,63E-06	7,17E-07	*TLR4/GSTP1/HAVCR2/LTF/LILRB1/DICER1/FOXP3/IGF1/IL4/IL10*	10
GO:0002064	epithelial cell development	16/293	207/18670	1,83E-07	1,68E-06	7,42E-07	*AR/FGFR1/BAD/ESR1/TNFRSF1A/ACTA2/IL1B/TNF/TP63/PROC/HIF1A/HNF4A/FLNA/TYMS/VEGFA/PGR*	16
GO:0071356	cellular response to tumor necrosis factor	19/293	291/18670	1,86E-07	1,71E-06	7,51E-07	*NFKB1/AKT1/TRAF1/CHUK/IKBKG/TNFRSF1A/HSPA1B/HSPA1A/JAK2/GSTP1/TNF/APOB/BRCA1/LCN2/CEBPA/COL1A1/CXCL8/HAMP/LTA*	19
GO:1905952	regulation of lipid localization	14/293	157/18670	1,89E-07	1,73E-06	7,62E-07	*NFKB1/AKT1/PPARG/IL1B/AGTR1/TNF/IL6/AGT/REN/APOB/APOE/ITGB3/EGF/LEP*	14
GO:0019884	antigen processing and presentation of exogenous antigen	15/293	182/18670	1,94E-07	1,77E-06	7,81E-07	*CHUK/IKBKG/HLA-E/HLA-C/HLA-G/HLA-A/HLA-B/TAP2/HLA-DQA1/HLA-DRB5/HLA-DRA/HLA-DQB2/HLA-DRB1/HLA-DQB1/HLA-DQA2*	15
GO:0051817	modification of morphology or physiology of other organism involved in symbiotic interaction	12/293	112/18670	1,95E-07	1,78E-06	7,83E-07	*BAD/F2/MBL2/BCL2L1/TYMS/APOE/LTF/TGFB1/EIF2AK2/INSR/FMR1/IGF2R*	12
GO:0090287	regulation of cellular response to growth factor stimulus	19/293	292/18670	1,96E-07	1,78E-06	7,86E-07	*TMPRSS6/TP53/FGFR1/HSPA1A/TGFBR1/IL1B/AGT/HRG/ARNT/HIF1A/ITGB3/VEGFA/TGFB1/FBN1/FST/SULF1/FGF1/VEGFC/FLT1*	19
GO:0032945	negative regulation of mononuclear cell proliferation	10/293	73/18670	2,02E-07	1,83E-06	8,07E-07	*CASP3/HAVCR2/HLA-G/LILRB1/CEBPB/TGFB1/CTLA4/FOXP3/PDE5A/IL10*	10
GO:0050672	negative regulation of lymphocyte proliferation	10/293	73/18670	2,02E-07	1,83E-06	8,07E-07	*CASP3/HAVCR2/HLA-G/LILRB1/CEBPB/TGFB1/CTLA4/FOXP3/PDE5A/IL10*	10
GO:0001912	positive regulation of leukocyte mediated cytotoxicity	9/293	56/18670	2,03E-07	1,84E-06	8,09E-07	*HLA-E/HLA-C/HLA-G/HLA-A/HLA-B/IL12B/IL23R/IL12A/NOS2*	9
GO:0050798	activated T cell proliferation	8/293	41/18670	2,04E-07	1,84E-06	8,12E-07	*CASP3/HMGB1/IL12B/IGF2/IL23R/FOXP3/IGF1/IL6R*	8
GO:0140014	mitotic nuclear division	18/293	264/18670	2,07E-07	1,87E-06	8,23E-07	*HSPA1B/HSPA1A/RAN/IL1B/LCMT1/FLNA/APC/BUB1/BUB1B/SPAG5/BUB3/TGFB1/EGF/INSR/INS/IGF2/IGF1/IL1A*	18

ID	Description	GeneRatio	BgRatio	pvalue	p.adjust	qvalue	geneID	Count
GO:0006006	glucose metabolic process	16/293	209/18670	2,09E-07	1,88E-06	8,29E-07	*TP53/SRC/AKT1/MTOR/BAD/IGFBP3/TNF/RBP4/LEPR/LCMT1/IGFBP4/INSR/INS/IGF2/IGF1/LEP*	16
GO:0045348	positive regulation of MHC class II biosynthetic process	5/293	10/18670	2,17E-07	1,95E-06	8,59E-07	*TLR4/JAK2/IFNG/IL4/IL10*	5
GO:0051918	negative regulation of fibrinolysis	5/293	10/18670	2,17E-07	1,95E-06	8,59E-07	*F2/HRG/CPB2/SERPINE1/THBD*	5
GO:0050864	regulation of B cell activation	15/293	184/18670	2,24E-07	2,00E-06	8,82E-07	*TLR4/CASP3/BAD/CD320/ADA/IL6/AHR/BCL2/INHA/TGFB1/CTLA4/FOXP3/TFRC/IL4/IL10*	15
GO:0014015	positive regulation of gliogenesis	10/293	74/18670	2,30E-07	2,06E-06	9,06E-07	*MTOR/PPARG/BMP2/IL1B/TNF/IL6/TP73/TGFB1/DICER1/LTA*	10
GO:0097028	dendritic cell differentiation	8/293	42/18670	2,48E-07	2,21E-06	9,76E-07	*GATA1/UBD/HMGB1/HLA-G/LILRB1/CEBPB/TGFB1/IL4*	8
GO:2000116	regulation of cysteine-type endopeptidase activity	17/293	239/18670	2,49E-07	2,22E-06	9,77E-07	*SRC/AKT1/MDM2/BAD/PPARG/JAK2/TNF/TP63/CASP9/BAX/MMP9/HMGB1/LTF/VEGFA/IFI6/FASLG/F3*	17
GO:0045621	positive regulation of lymphocyte differentiation	11/293	94/18670	2,57E-07	2,28E-06	1,00E-06	*BAD/ADA/HLA-G/TGFB1/IL12B/IL23R/FOXP3/IFNG/IL12A/IL4R/IL4*	11
GO:0031334	positive regulation of protein complex assembly	18/293	268/18670	2,59E-07	2,30E-06	1,01E-06	*TP53/SRC/TLR4/MTOR/ESR1/HSPA1B/HSPA1A/TNF/BAX/MMP3/GTF2H4/MMP1/VEGFA/CREB1/CNOT1/TGFB1/IFNG/HNF1A*	18
GO:0090068	positive regulation of cell cycle process	19/293	298/18670	2,68E-07	2,37E-06	1,05E-06	*TP53/AIF1/AKT1/EGFR/MDM2/FGFR1/IL1B/TP73/BAX/SPAG5/BRCA1/CNOT1/TGFB1/EGF/INSR/INS/IGF2/IGF1/IL1A*	19
GO:0042593	glucose homeostasis	17/293	241/18670	2,81E-07	2,48E-06	1,09E-06	*AKT1/STAT3/BAD/IGF1R/PPARG/ADRA2A/RBP4/LEPR/HIF1A/HNF4A/CPB2/CEBPA/CFTR/INSR/INS/HNF1A/LEP*	17
GO:0042100	B cell proliferation	11/293	95/18670	2,86E-07	2,52E-06	1,11E-06	*TLR4/CASP3/CD320/ADA/AHR/BAX/BCL2/CTLA4/TFRC/IL4/IL10*	11
GO:0044070	regulation of anion transport	11/293	95/18670	2,86E-07	2,52E-06	1,11E-06	*ABCB1/ACE2/AKT1/MTOR/FGFR1/IL1B/AGT/APOE/CEBPB/CFTR/LEP*	11
GO:0043687	post-translational protein modification	21/293	361/18670	2,90E-07	2,54E-06	1,12E-06	*VHL/IGFBP3/FN1/IL6/FGG/PROC/IGFBP1/SERPINA10/SERPINC1/HIF1A/LCMT1/FGA/APOB/APOE/SERPINA1/IGFBP4/TIMP1/UBD/FBN1/F5/FOLH1*	21
GO:0051235	maintenance of location	20/293	330/18670	2,92E-07	2,56E-06	1,13E-06	*NFKB1/AKT1/PPARG/IL1B/CCR5/TNF/IL6/F2/FLNA/BAX/APOB/APOE/ITGB3/LCN2/TGFB1/FBN1/FASLG/LEP/TRPC1/IL10*	20
GO:0006367	transcription initiation from RNA polymerase II promoter	15/293	188/18670	2,96E-07	2,58E-06	1,14E-06	*TP53/THRB/NR3C1/ESR2/AR/ESR1/PPARG/HNF4A/BAX/GTF2H4/HMGB1/CREB1/RXRB/PGR/HNF1A*	15
GO:0051783	regulation of nuclear division	15/293	188/18670	2,96E-07	2,58E-06	1,14E-06	*HSPA1B/HSPA1A/IL1B/LCMT1/APC/BUB1/BUB1B/BUB3/TGFB1/EGF/INSR/INS/IGF2/IGF1/IL1A*	15

ID	Description	GeneRatio	BgRatio	pvalue	p.adjust	qvalue	geneID	Count
GO:0033500	carbohydrate homeostasis	17/293	242/18670	2,98E-07	2,59E-06	1,14E-06	*AKT1/STAT3/BAD/IGF1R/PPARG/ADRA2A/RBP4/LEPR/HIF1A/HNF4A/CPB2/CEBPA/CFTR/INSR/INS/HNF1A/LEP*	17
GO:0045580	regulation of T cell differentiation	13/293	139/18670	2,99E-07	2,60E-06	1,15E-06	*BAD/ADA/HMGB1/HLA-G/TGFB1/IL12B/CTLA4/IL23R/FOXP3/IFNG/IL12A/IL4R/IL4*	13
GO:0031214	biomineral tissue development	14/293	163/18670	3,00E-07	2,60E-06	1,15E-06	*NOS3/GATA1/ACVR1/BMP2/ADRB2/HIF1A/LTF/CEBPB/TGFB1/COL1A1/COL1A2/FGFR3/IGF1/LEP*	14
GO:0045931	positive regulation of mitotic cell cycle	14/293	163/18670	3,00E-07	2,60E-06	1,15E-06	*AIF1/AKT1/EGFR/MDM2/FGFR1/IL1B/BRCA2/TGFB1/EGF/INSR/INS/IGF2/IGF1/IL1A*	14
GO:0071214	cellular response to abiotic stimulus	20/293	331/18670	3,07E-07	2,64E-06	1,16E-06	*NFKB1/TP53/AKT1/TLR4/EGFR/MDM2/CASP3/BAD/TNFRSF1A/IL1B/AGT/CASP9/BAX/BCL2L1/ITGA2/MMP7/TGFB1/COL1A1/FMR1/HAMP*	20
GO:0104004	cellular response to environmental stimulus	20/293	331/18670	3,07E-07	2,64E-06	1,16E-06	*NFKB1/TP53/AKT1/TLR4/EGFR/MDM2/CASP3/BAD/TNFRSF1A/IL1B/AGT/CASP9/BAX/BCL2L1/ITGA2/MMP7/TGFB1/COL1A1/FMR1/HAMP*	20
GO:0010810	regulation of cell-substrate adhesion	16/293	215/18670	3,07E-07	2,64E-06	1,16E-06	*SRC/JAK2/FN1/FGG/HRG/FLNA/BCL2/FGB/FGA/MMP12/SERPINE1/CEACAM6/VEGFA/COL1A1/FBLN1/KDR*	16
GO:0043281	regulation of cysteine-type endopeptidase activity involved in apoptotic process	16/293	215/18670	3,07E-07	2,64E-06	1,16E-06	*SRC/AKT1/MDM2/BAD/PPARG/JAK2/TNF/TP63/CASP9/BAX/MMP9/HMGB1/VEGFA/IFI6/FASLG/F3*	16
GO:0090277	positive regulation of peptide hormone secretion	11/293	96/18670	3,19E-07	2,74E-06	1,21E-06	*EGFR/BAD/JAK2/FGG/RBP4/HIF1A/FGB/FGA/CFTR/INS/HFE*	11
GO:0001836	release of cytochrome c from mitochondria	9/293	59/18670	3,23E-07	2,76E-06	1,22E-06	*TP53/AKT1/BAD/BAX/BCL2/BCL2L1/MMP9/IFI6/IGF1*	9
GO:0043388	positive regulation of DNA binding	9/293	59/18670	3,23E-07	2,76E-06	1,22E-06	*PPARG/JAK2/MMP9/HMGB1/ITGA2/TGFB1/EGF/IFNG/IGF1*	9
GO:0030949	positive regulation of vascular endothelial growth factor receptor signaling pathway	6/293	19/18670	3,24E-07	2,77E-06	1,22E-06	*IL1B/ARNT/HIF1A/ITGB3/VEGFA/FLT1*	6
GO:0032700	negative regulation of interleukin-17 production	6/293	19/18670	3,24E-07	2,77E-06	1,22E-06	*TLR4/TGFB1/IL12B/FOXP3/IFNG/IL12A*	6
GO:0010038	response to metal ion	21/293	364/18670	3,32E-07	2,83E-06	1,25E-06	*ACTA1/AKT1/EGFR/MDM2/CASP3/CHUK/BAD/FGG/HIF1A/CASP9/BCL2/FGB/FGA/MMP9/CD14/CEBPA/CREB1/HAMP/HFE/IL1A/TRPC1*	21
GO:0061448	connective tissue development	18/293	273/18670	3,40E-07	2,89E-06	1,27E-06	*FGFR1/ACTA2/TGFBR1/BMP2/MBL2/HIF1A/TYMS/TIMP1/TGFB1/COL1A1/TGFBI/COL5A1/COL6A3/COL6A1/SULF1/FGFR3/LEP/IL6R*	18

ID	Description	GeneRatio	BgRatio	pvalue	p.adjust	qvalue	geneID	Count
GO:0043393	regulation of protein binding	16/293	217/18670	3,48E-07	2,95E-06	1,30E-06	*ACE/SRC/AKT1/LGALS3/TGFBR1/BMP2/RAN/ADRB2/BAX/BCL2/APOE/MAP2/MMP9/HFE/MAPRE3/IL10*	16
GO:0043255	regulation of carbohydrate biosynthetic process	11/293	97/18670	3,54E-07	3,00E-06	1,32E-06	*NFKB1/AKT1/MTOR/LEPR/TGFB1/EGF/INSR/INS/IGF2/IGF1/LEP*	11
GO:0014002	astrocyte development	8/293	44/18670	3,63E-07	3,06E-06	1,35E-06	*TLR4/EGFR/IL1B/TNF/IL6/C5AR1/IFNG/ROR1*	8
GO:0042269	regulation of natural killer cell mediated cytotoxicity	8/293	44/18670	3,63E-07	3,06E-06	1,35E-06	*HAVCR2/HLA-E/HLA-G/LILRB1/CEACAM1/IL12B/IL12A/LEP*	8
GO:0003012	muscle system process	24/293	465/18670	3,64E-07	3,07E-06	1,35E-06	*ACE2/ACTA1/AIF1/NOS3/CHUK/MTOR/TNFRSF1A/ACTA2/ADA/ADRA2A/ADRB2/IL1B/AGT/FLNA/PRKG1/ITGA2/SULF1/DES/NEB/VEGFB/HAMP/IGF1/LEP/PDE5A*	24
GO:0002294	CD4-positive, alpha-beta T cell differentiation involved in immune response	9/293	60/18670	3,75E-07	3,14E-06	1,39E-06	*STAT3/MTOR/IL6/HMGB1/IL12B/IL23R/FOXP3/IFNG/IL4R*	9
GO:0042267	natural killer cell mediated cytotoxicity	9/293	60/18670	3,75E-07	3,14E-06	1,39E-06	*HAVCR2/HLA-E/HLA-G/LILRB1/CEACAM1/IL12B/KIR3DL1/IL12A/LEP*	9
GO:0043270	positive regulation of ion transport	18/293	275/18670	3,79E-07	3,18E-06	1,40E-06	*ABCB1/ACE2/AKT1/TRPC6/LGALS3/ADRA2A/ADRB2/IL1B/AGT/F2/FLNA/BAX/APOE/CEBPB/CFTR/IFNG/LEP/TRPC1*	18
GO:0046886	positive regulation of hormone biosynthetic process	5/293	11/18670	3,93E-07	3,29E-06	1,45E-06	*IL1B/TNF/ARNT/HIF1A/IFNG*	5
GO:0097237	cellular response to toxic substance	17/293	247/18670	3,97E-07	3,32E-06	1,46E-06	*NFKB1/SRC/TRPC6/MDM2/NOS3/BAD/GSTP1/TNF/IL6/HBE1/APOE/LCN2/DHFR/KDR/HBA1/HP/IL10*	17
GO:0072089	stem cell proliferation	12/293	120/18670	4,16E-07	3,46E-06	1,53E-06	*ABCB1/PIM1/ACE/TP53/FGFR1/HIF1A/ETV6/VEGFA/TGFB1/FBLN1/EIF2AK2/VEGFC*	12
GO:0043536	positive regulation of blood vessel endothelial cell migration	10/293	79/18670	4,31E-07	3,58E-06	1,58E-06	*AKT1/NOS3/GATA2/FGFR1/HIF1A/HMGB1/VEGFA/TGFB1/KDR/VEGFC*	10
GO:0002287	alpha-beta T cell activation involved in immune response	9/293	61/18670	4,33E-07	3,58E-06	1,58E-06	*STAT3/MTOR/IL6/HMGB1/IL12B/IL23R/FOXP3/IFNG/IL4R*	9
GO:0002293	alpha-beta T cell differentiation involved in immune response	9/293	61/18670	4,33E-07	3,58E-06	1,58E-06	*STAT3/MTOR/IL6/HMGB1/IL12B/IL23R/FOXP3/IFNG/IL4R*	9
GO:0006879	cellular iron ion homeostasis	9/293	61/18670	4,33E-07	3,58E-06	1,58E-06	*TMPRSS6/HEPH/HIF1A/SLC11A1/LTF/LCN2/HAMP/HFE/TFRC*	9

ID	Description	GeneRatio	BgRatio	pvalue	p.adjust	qvalue	geneID	Count
GO:0034113	heterotypic cell-cell adhesion	9/293	61/18670	4,33E-07	3,58E-06	1,58E-06	*IL1B/TNF/FGG/FGB/FGA/ITGB3/CEACAM6/IL1RN/IL10*	9
GO:0001974	blood vessel remodeling	8/293	45/18670	4,36E-07	3,59E-06	1,58E-06	*ACE/MDM2/NOS3/AGT/HRG/BAX/CEACAM1/TGFB1*	8
GO:0001704	formation of primary germ layer	12/293	121/18670	4,55E-07	3,75E-06	1,65E-06	*FGFR1/FN1/ACVR1/MMP2/ITGB4/ITGB3/MMP9/ITGA2/ COL5A1/COL5A2/COL6A1/MMP15*	12
GO:0002827	positive regulation of T-helper 1 type immune response	6/293	20/18670	4,57E-07	3,76E-06	1,66E-06	*IL1B/IL1R1/SLC11A1/IL12B/IL23R/IL6R*	6
GO:0045619	regulation of lymphocyte differentiation	14/293	169/18670	4,68E-07	3,83E-06	1,69E-06	*BAD/ADA/INHA/HMGB1/HLA-G/TGFB1/IL12B/CTLA4/IL23R/FOXP3/IFNG/IL12A/IL4R/IL4*	14
GO:0050728	negative regulation of inflammatory response	14/293	169/18670	4,68E-07	3,83E-06	1,69E-06	*NFKB1/TNFRSF1A/PPARG/GSTP1/ADA/F2/PROC/APOE/ IL12B/INS/FOXP3/IGF1/IL4/IL10*	14
GO:0043900	regulation of multi-organism process	22/293	405/18670	4,83E-07	3,95E-06	1,74E-06	*BAD/ADA/IL1B/TNF/MBL2/BCL2/APOE/MMP12/TIMP1/ HAVCR2/LTF/LILRB1/IL12B/CXCL8/EIF2AK2/FMR1/IL23R/IFNG/IGF1/IGF2R/NOS2/PDE5A*	22
GO:0055024	regulation of cardiac muscle tissue development	11/293	100/18670	4,84E-07	3,95E-06	1,74E-06	*PIM1/MTOR/FGFR1/TGFBR1/BMP2/TP73/RBP4/CREB1/ TGFB1/HAMP/IGF1*	11
GO:0002260	lymphocyte homeostasis	9/293	62/18670	5,00E-07	4,07E-06	1,79E-06	*AKT1/CASP3/ADA/HIF1A/BAX/BCL2/TGFB1/FOXP3/TSC22D3*	9
GO:0046635	positive regulation of alpha-beta T cell activation	9/293	62/18670	5,00E-07	4,07E-06	1,79E-06	*ADA/HLA-E/IL12B/IL23R/FOXP3/IFNG/IL12A/IL4R/IL6R*	9
GO:0002715	regulation of natural killer cell mediated immunity	8/293	46/18670	5,20E-07	4,20E-06	1,85E-06	*HAVCR2/HLA-E/HLA-G/LILRB1/CEACAM1/IL12B/IL12A/LEP*	8
GO:0022602	ovulation cycle process	8/293	46/18670	5,20E-07	4,20E-06	1,85E-06	*SRC/CASP3/NOS3/ESR1/FSHB/FSHR/PGR/LEP*	8
GO:0071354	cellular response to interleukin-6	8/293	46/18670	5,20E-07	4,20E-06	1,85E-06	*NFKB1/STAT3/JAK2/IL6/FGG/CEBPA/HAMP/IL6R*	8
GO:1904036	negative regulation of epithelial cell apoptotic process	8/293	46/18670	5,20E-07	4,20E-06	1,85E-06	*GATA2/MTOR/FGG/FGB/FGA/SERPINE1/KDR/IL4*	8
GO:0032814	regulation of natural killer cell activation	7/293	32/18670	5,25E-07	4,23E-06	1,86E-06	*HAVCR2/HLA-E/IL12B/IL23R/IL12A/LEP/IL6R*	7
GO:0002224	toll-like receptor signaling pathway	13/293	146/18670	5,29E-07	4,25E-06	1,87E-06	*TLR4/CHUK/ESR1/IKBKG/FGG/FGB/FGA/APOB/HAVCR2/HMGB1/CD14/LTF/TLR1*	13
GO:0042542	response to hydrogen peroxide	13/293	146/18670	5,29E-07	4,25E-06	1,87E-06	*SRC/TRPC6/MDM2/CASP3/BAD/ADA/IL6/BCL2/LCN2/COL1A1/HBA1/HP/IL10*	13
GO:0046632	alpha-beta T cell differentiation	11/293	101/18670	5,36E-07	4,30E-06	1,89E-06	*STAT3/MTOR/ADA/IL6/BCL2/HMGB1/IL12B/IL23R/FOXP3/IFNG/IL4R*	11

ID	Description	GeneRatio	BgRatio	pvalue	p.adjust	qvalue	geneID	Count
GO:1902930	regulation of alcohol biosynthetic process	10/293	81/18670	5,47E-07	4,38E-06	1,93E-06	*NFKB1/BMP2/RAN/IL1B/TNF/APOB/APOE/DHCR7/FGF1/IFNG*	10
GO:0060541	respiratory system development	15/293	198/18670	5,76E-07	4,60E-06	2,03E-06	*THRB/EGFR/NOS3/FGFR1/ADA/TNF/RBP4/BAG6/CEBPA/VEGFA/CREB1/DHCR7/PGR/FGF1/FGF7*	15
GO:0008637	apoptotic mitochondrial changes	12/293	124/18670	5,94E-07	4,73E-06	2,09E-06	*TP53/AKT1/BAD/HSPA1A/TP63/TP73/BAX/BCL2/BCL2L1/MMP9/IFI6/IGF1*	12
GO:0014013	regulation of gliogenesis	12/293	124/18670	5,94E-07	4,73E-06	2,09E-06	*MTOR/PPARG/BMP2/IL1B/TNF/IL6/TP73/F2/CREB1/TGFB1/DICER1/LTA*	12
GO:0030574	collagen catabolic process	8/293	47/18670	6,18E-07	4,89E-06	2,16E-06	*TMPRSS6/MMP2/MMP12/MMP3/MMP9/MMP1/MMP7/MMP15*	8
GO:0045646	regulation of erythrocyte differentiation	8/293	47/18670	6,18E-07	4,89E-06	2,16E-06	*STAT3/STAT5B/GATA2/GATA1/HSPA1B/HSPA1A/ARNT/HIF1A*	8
GO:0070231	T cell apoptotic process	8/293	47/18670	6,18E-07	4,89E-06	2,16E-06	*TP53/AKT1/LGALS3/ADA/HIF1A/BAX/FASLG/TSC22D3*	8
GO:1903580	positive regulation of ATP metabolic process	8/293	47/18670	6,18E-07	4,89E-06	2,16E-06	*STAT3/ARNT/HIF1A/INSR/INS/IFNG/IGF1/IL4*	8
GO:0010893	positive regulation of steroid biosynthetic process	6/293	21/18670	6,32E-07	4,98E-06	2,20E-06	*IL1B/TNF/CGA/FSHB/FGF1/IFNG*	6
GO:0035743	CD4-positive, alpha-beta T cell cytokine production	6/293	21/18670	6,32E-07	4,98E-06	2,20E-06	*IL1B/IL6/IL1R1/IL12B/IL12A/IL4*	6
GO:0001894	tissue homeostasis	16/293	227/18670	6,38E-07	5,03E-06	2,21E-06	*SRC/TLR4/EGFR/NOS3/GATA2/GATA1/ADRB2/IL6/RBP4/BAX/BCL2/ITGB3/LTF/VEGFA/FSHB/TFRC*	16
GO:2000352	negative regulation of endothelial cell apoptotic process	7/293	33/18670	6,57E-07	5,17E-06	2,28E-06	*GATA2/FGG/FGB/FGA/SERPINE1/KDR/IL4*	7
GO:0002228	natural killer cell mediated immunity	9/293	64/18670	6,61E-07	5,17E-06	2,28E-06	*HAVCR2/HLA-E/HLA-G/LILRB1/CEACAM1/IL12B/KIR3DL1/IL12A/LEP*	9
GO:0045669	positive regulation of osteoblast differentiation	9/293	64/18670	6,61E-07	5,17E-06	2,28E-06	*ACVR1/BMP2/IL6/TP63/LTF/CEBPA/CEBPB/IGF1/IL6R*	9
GO:2000378	negative regulation of reactive oxygen species metabolic process	9/293	64/18670	6,61E-07	5,17E-06	2,28E-06	*TP53/STAT3/HIF1A/BCL2/MMP3/BRCA1/INS/HP/IL10*	9
GO:0010623	programmed cell death involved in cell development	5/293	12/18670	6,66E-07	5,20E-06	2,29E-06	*IL1B/BAX/BCL2/FASLG/IL1A*	5
GO:0051403	stress-activated MAPK cascade	18/293	286/18670	6,73E-07	5,24E-06	2,31E-06	*NFKB1/AKT1/TLR4/EGFR/CHUK/IKBKG/IGF1R/GSTP1/BMP2/IL1B/TNF/UBE2N/AGT/HMGB1/VEGFA/EIF2AK2/IL1RN/LEP*	18

ID	Description	GeneRatio	BgRatio	pvalue	p.adjust	qvalue	geneID	Count
GO:0008203	cholesterol metabolic process	13/293	150/18670	7,21E-07	5,61E-06	2,47E-06	*RAN/LEPR/APOB/APOE/NSDHL/CEBPA/CFTR/CNBP/DHCR7/EBP/FGF1/IL4/LEP*	13
GO:0002762	negative regulation of myeloid leukocyte differentiation	8/293	48/18670	7,32E-07	5,68E-06	2,50E-06	*TLR4/GATA2/INHA/LTF/LILRB1/CEACAM1/FBN1/IL4*	8
GO:0046850	regulation of bone remodeling	8/293	48/18670	7,32E-07	5,68E-06	2,50E-06	*SRC/EGFR/IL6/LEPR/ITGB3/FSHB/TFRC/LEP*	8
GO:0050886	endocrine process	10/293	84/18670	7,71E-07	5,97E-06	2,63E-06	*ACE/ACE2/NOS3/FGFR1/IL1B/AGTR1/AGT/REN/INHA/LEP*	10
GO:0030278	regulation of ossification	15/293	203/18670	7,91E-07	6,12E-06	2,70E-06	*GATA1/ACVR1/BMP2/ADRB2/TNF/IL6/TP63/HIF1A/BCL2/LTF/CEBPA/CEBPB/TGFB1/IGF1/IL6R*	15
GO:0042533	tumor necrosis factor biosynthetic process	7/293	34/18670	8,17E-07	6,27E-06	2,76E-06	*TLR4/JAK2/LILRB1/IFNG/IL4/TLR1/IL10*	7
GO:0042534	regulation of tumor necrosis factor biosynthetic process	7/293	34/18670	8,17E-07	6,27E-06	2,76E-06	*TLR4/JAK2/LILRB1/IFNG/IL4/TLR1/IL10*	7
GO:0043276	anoikis	7/293	34/18670	8,17E-07	6,27E-06	2,76E-06	*SRC/AKT1/MTOR/IKBKG/BCL2/CEACAM6/CEACAM5*	7
GO:0043368	positive T cell selection	7/293	34/18670	8,17E-07	6,27E-06	2,76E-06	*STAT3/MTOR/IL6/BCL2/IL12B/IL23R/FOXP3*	7
GO:0048469	cell maturation	14/293	177/18670	8,20E-07	6,29E-06	2,77E-06	*GATA2/MTOR/FGFR1/PPARG/REN/FGG/HIF1A/BCL2/TYMS/BRCA2/CEBPA/VEGFA/CFTR/PGR*	14
GO:0031342	negative regulation of cell killing	6/293	22/18670	8,57E-07	6,55E-06	2,89E-06	*HAVCR2/HLA-E/HLA-G/LILRB1/CEACAM1/IL4*	6
GO:0051000	positive regulation of nitric-oxide synthase activity	6/293	22/18670	8,57E-07	6,55E-06	2,89E-06	*AKT1/TNF/HIF1A/APOE/DHFR/INS*	6
GO:0032757	positive regulation of interleukin-8 production	8/293	49/18670	8,63E-07	6,57E-06	2,89E-06	*TLR4/HSPA1B/HSPA1A/IL1B/TNF/SERPINE1/CD14/TLR1*	8
GO:0051205	protein insertion into membrane	8/293	49/18670	8,63E-07	6,57E-06	2,89E-06	*TP53/EGFR/BAD/TP63/TP73/BAX/BCL2/BAG6*	8
GO:1904707	positive regulation of vascular smooth muscle cell proliferation	8/293	49/18670	8,63E-07	6,57E-06	2,89E-06	*MDM2/JAK2/TNF/AGT/MMP2/MMP9/IGF1/IL10*	8
GO:0051146	striated muscle cell differentiation	18/293	293/18670	9,54E-07	7,25E-06	3,19E-06	*ACTA1/AKT1/CASP3/CHUK/MTOR/ACVR1/BMP2/AGT/BCL2/CD9/CEACAM5/VEGFA/TGFB1/NEB/IGF2/HAMP/IGF1/IL4R*	18
GO:0062014	negative regulation of small molecule metabolic process	11/293	107/18670	9,61E-07	7,27E-06	3,20E-06	*NFKB1/TP53/AKT1/STAT3/BMP2/LEPR/APOE/BRCA1/CEACAM1/TGFB1/INS*	11
GO:0032760	positive regulation of tumor necrosis factor production	10/293	86/18670	9,62E-07	7,27E-06	3,20E-06	*TLR4/JAK2/HAVCR2/HMGB1/CD14/HLA-E/IL12B/IFNG/LEP/TLR1*	10

ID	Description	GeneRatio	BgRatio	pvalue	p.adjust	qvalue	geneID	Count
GO:0090559	regulation of membrane permeability	10/293	86/18670	9,62E-07	7,27E-06	3,20E-06	*TP53/STAT3/MTOR/BAD/HSPA1A/TP63/TP73/BAX/BCL2/BCL2L1*	10
GO:0048662	negative regulation of smooth muscle cell proliferation	9/293	67/18670	9,84E-07	7,43E-06	3,27E-06	*AIF1/IGFBP3/PPARG/GSTP1/PRKG1/IL12B/IFNG/IL12A/IL10*	9
GO:0043534	blood vessel endothelial cell migration	14/293	180/18670	1,00E-06	7,57E-06	3,33E-06	*AKT1/NOS3/GATA2/FGFR1/PPARG/HRG/HIF1A/APOE/HMGB1/VEGFA/TGFB1/EPHB4/KDR/VEGFC*	14
GO:0032689	negative regulation of interferon-gamma production	7/293	35/18670	1,01E-06	7,57E-06	3,33E-06	*TLR4/INHA/HAVCR2/HMGB1/LILRB1/FOXP3/IL10*	7
GO:0060251	regulation of glial cell proliferation	7/293	35/18670	1,01E-06	7,57E-06	3,33E-06	*MTOR/IL1B/TNF/IL6/CREB1/DICER1/LTA*	7
GO:0010718	positive regulation of epithelial to mesenchymal transition	8/293	50/18670	1,01E-06	7,58E-06	3,34E-06	*MTOR/ACVR1/TGFBR1/BMP2/IL1B/IL6/TGFB1/COL1A1*	8
GO:0014009	glial cell proliferation	8/293	50/18670	1,01E-06	7,58E-06	3,34E-06	*MTOR/IL1B/TNF/IL6/LEPR/CREB1/DICER1/LTA*	8
GO:0070741	response to interleukin-6	8/293	50/18670	1,01E-06	7,58E-06	3,34E-06	*NFKB1/STAT3/JAK2/IL6/FGG/CEBPA/HAMP/IL6R*	8
GO:1903707	negative regulation of hemopoiesis	13/293	155/18670	1,05E-06	7,83E-06	3,45E-06	*TLR4/GATA2/INHA/HMGB1/LTF/HLA-G/LILRB1/CEACAM1/FBN1/CTLA4/FOXP3/IL4R/IL4*	13
GO:0030656	regulation of vitamin metabolic process	5/293	13/18670	1,07E-06	7,94E-06	3,50E-06	*NFKB1/CD320/IL1B/TNF/IFNG*	5
GO:1901550	regulation of endothelial cell development	5/293	13/18670	1,07E-06	7,94E-06	3,50E-06	*TNFRSF1A/IL1B/TNF/PROC/VEGFA*	5
GO:1903140	regulation of establishment of endothelial barrier	5/293	13/18670	1,07E-06	7,94E-06	3,50E-06	*TNFRSF1A/IL1B/TNF/PROC/VEGFA*	5
GO:0051781	positive regulation of cell division	10/293	87/18670	1,07E-06	7,95E-06	3,50E-06	*IL1B/VEGFA/TGFB1/IGF2/FGF1/FGF7/VEGFC/VEGFB/PGF/IL1A*	10
GO:0002292	T cell differentiation involved in immune response	9/293	68/18670	1,12E-06	8,27E-06	3,65E-06	*STAT3/MTOR/IL6/HMGB1/IL12B/IL23R/FOXP3/IFNG/IL4R*	9
GO:0019915	lipid storage	9/293	68/18670	1,12E-06	8,27E-06	3,65E-06	*NFKB1/PPARG/IL1B/TNF/IL6/APOB/APOE/ITGB3/LEP*	9
GO:0043535	regulation of blood vessel endothelial cell migration	13/293	156/18670	1,13E-06	8,33E-06	3,67E-06	*AKT1/NOS3/GATA2/FGFR1/PPARG/HRG/HIF1A/APOE/HMGB1/VEGFA/TGFB1/KDR/VEGFC*	13
GO:0043122	regulation of I-kappaB kinase/NF-kappaB signaling	16/293	237/18670	1,13E-06	8,34E-06	3,68E-06	*AKT1/TLR4/CHUK/ESR1/IKBKG/TNFRSF1A/GSTP1/IL1B/TNF/UBE2N/FLNA/UBD/LTF/FASLG/IL1RN/ROR1*	16

ID	Description	GeneRatio	BgRatio	pvalue	p.adjust	qvalue	geneID	Count
GO:0051216	cartilage development	15/293	209/18670	1,14E-06	8,40E-06	3,70E-06	*FGFR1/TGFBR1/BMP2/MBL2/HIF1A/TYMS/TIMP1/TGFB1/COL1A1/TGFBI/COL6A3/COL6A1/SULF1/FGFR3/LEP*	15
GO:0002347	response to tumor cell	6/293	23/18670	1,14E-06	8,40E-06	3,70E-06	*HRG/HAVCR2/HMGB1/CEACAM1/IL12B/IL12A*	6
GO:0045821	positive regulation of glycolytic process	6/293	23/18670	1,14E-06	8,40E-06	3,70E-06	*ARNT/HIF1A/INSR/INS/IFNG/IGF1*	6
GO:0010821	regulation of mitochondrion organization	14/293	182/18670	1,15E-06	8,40E-06	3,70E-06	*HSPA1L/TP53/AKT1/BAD/HSPA1A/TP63/TP73/HIF1A/BAX/BCL2/BCL2L1/MMP9/KDR/IGF1*	14
GO:0002712	regulation of B cell mediated immunity	8/293	51/18670	1,19E-06	8,66E-06	3,82E-06	*TNF/HLA-E/TGFB1/FOXP3/TFRC/IL4/LTA/IL10*	8
GO:0002889	regulation of immunoglobulin mediated immune response	8/293	51/18670	1,19E-06	8,66E-06	3,82E-06	*TNF/HLA-E/TGFB1/FOXP3/TFRC/IL4/LTA/IL10*	8
GO:0050829	defense response to Gram-negative bacterium	10/293	88/18670	1,19E-06	8,68E-06	3,83E-06	*TLR4/IL6/F2/SERPINE1/SLC11A1/LTF/IL12B/IL23R/NOS2/IL6R*	10
GO:1903557	positive regulation of tumor necrosis factor superfamily cytokine production	10/293	88/18670	1,19E-06	8,68E-06	3,83E-06	*TLR4/JAK2/HAVCR2/HMGB1/CD14/HLA-E/IL12B/IFNG/LEP/TLR1*	10
GO:0051048	negative regulation of secretion	16/293	238/18670	1,20E-06	8,70E-06	3,83E-06	*TNFRSF1A/FN1/ADA/ADRA2A/IL1B/TNF/INHA/APOE/LILRB1/CEACAM1/EGF/INS/FMR1/FOXP3/LEP/IL10*	16
GO:0009612	response to mechanical stimulus	15/293	210/18670	1,21E-06	8,81E-06	3,88E-06	*NFKB1/ACTA1/SRC/AKT1/TLR4/EGFR/BAD/TNFRSF1A/PPARG/IL1B/AGT/ITGA2/MMP7/TGFB1/COL1A1*	15
GO:0002758	innate immune response-activating signal transduction	18/293	298/18670	1,22E-06	8,82E-06	3,89E-06	*NFKB1/SRC/TLR4/CHUK/ESR1/IKBKG/HSPA1B/HSPA1A/UBE2N/FGG/FGB/FGA/APOB/HAVCR2/HMGB1/CD14/LTF/TLR1*	18
GO:0043123	positive regulation of I-kappaB kinase/NF-kappaB signaling	14/293	183/18670	1,22E-06	8,86E-06	3,90E-06	*AKT1/TLR4/CHUK/IKBKG/TNFRSF1A/IL1B/TNF/UBE2N/FLNA/UBD/LTF/FASLG/IL1RN/ROR1*	14
GO:0010742	macrophage derived foam cell differentiation	7/293	36/18670	1,23E-06	8,87E-06	3,91E-06	*NFKB1/PPARG/AGTR1/AGT/APOB/ITGB3/TGFB1*	7
GO:0030947	regulation of vascular endothelial growth factor receptor signaling pathway	7/293	36/18670	1,23E-06	8,87E-06	3,91E-06	*IL1B/ARNT/HIF1A/ITGB3/VEGFA/VEGFC/FLT1*	7
GO:0090077	foam cell differentiation	7/293	36/18670	1,23E-06	8,87E-06	3,91E-06	*NFKB1/PPARG/AGTR1/AGT/APOB/ITGB3/TGFB1*	7
GO:0097242	amyloid-beta clearance	7/293	36/18670	1,23E-06	8,87E-06	3,91E-06	*IGF1R/TNF/APOE/C5AR1/INSR/IFNG/IL4*	7

ID	Description	GeneRatio	BgRatio	pvalue	p.adjust	qvalue	geneID	Count
GO:0070227	lymphocyte apoptotic process	9/293	69/18670	1,27E-06	9,11E-06	4,02E-06	*TP53/AKT1/LGALS3/ADA/HIF1A/BAX/FASLG/TSC22D3/IL10*	9
GO:0002446	neutrophil mediated immunity	24/293	499/18670	1,28E-06	9,18E-06	4,04E-06	*ACE/NFKB1/PLD1/LGALS3/HSPA1B/HSPA1A/GSTP1/PA2G4/IL6/F2/SERPINA1/MMP9/HMGB1/C5AR1/SLC11A1/CD14/LTF/CEACAM1/CEACAM6/LCN2/TIMP2/CXCR1/HP/IGF2R*	24
GO:0043467	regulation of generation of precursor metabolites and energy	13/293	158/18670	1,30E-06	9,32E-06	4,11E-06	*TP53/AKT1/STAT3/MTOR/ARNT/HIF1A/INSR/INS/IGF2/IFNG/IGF1/IL4/NOS2*	13
GO:0007249	I-kappaB kinase/NF-kappaB signaling	17/293	269/18670	1,30E-06	9,32E-06	4,11E-06	*AKT1/TLR4/CHUK/ESR1/IKBKG/TNFRSF1A/GSTP1/IL1B/TNF/UBE2N/FLNA/UBD/CD14/LTF/FASLG/IL1RN/ROR1*	17
GO:0048639	positive regulation of developmental growth	14/293	184/18670	1,31E-06	9,32E-06	4,11E-06	*PIM1/AKT1/MTOR/FGFR1/FN1/L1CAM/BCL2/VEGFA/CREB1/INSR/IGF2/HAMP/IGF1/LEP*	14
GO:0030260	entry into host cell	12/293	134/18670	1,37E-06	9,70E-06	4,27E-06	*ACE2/SRC/EGFR/HSPA1B/NCAM1/HSPA1A/CCR5/ITGB6/ITGB3/ITGA2/CXCL8/TFRC*	12
GO:0044409	entry into host	12/293	134/18670	1,37E-06	9,70E-06	4,27E-06	*ACE2/SRC/EGFR/HSPA1B/NCAM1/HSPA1A/CCR5/ITGB6/ITGB3/ITGA2/CXCL8/TFRC*	12
GO:0051806	entry into cell of other organism involved in symbiotic interaction	12/293	134/18670	1,37E-06	9,70E-06	4,27E-06	*ACE2/SRC/EGFR/HSPA1B/NCAM1/HSPA1A/CCR5/ITGB6/ITGB3/ITGA2/CXCL8/TFRC*	12
GO:0051828	entry into other organism involved in symbiotic interaction	12/293	134/18670	1,37E-06	9,70E-06	4,27E-06	*ACE2/SRC/EGFR/HSPA1B/NCAM1/HSPA1A/CCR5/ITGB6/ITGB3/ITGA2/CXCL8/TFRC*	12
GO:0072132	mesenchyme morphogenesis	8/293	52/18670	1,38E-06	9,79E-06	4,32E-06	*ACTA1/MDM2/NOS3/FGFR1/ACTA2/ACVR1/TGFBR1/BMP2*	8
GO:0032720	negative regulation of tumor necrosis factor production	9/293	70/18670	1,44E-06	1,01E-05	4,47E-06	*TLR4/GSTP1/HAVCR2/LILRB1/DICER1/FOXP3/IGF1/IL4/IL10*	9
GO:0072091	regulation of stem cell proliferation	9/293	70/18670	1,44E-06	1,01E-05	4,47E-06	*PIM1/ACE/TP53/HIF1A/VEGFA/TGFB1/FBLN1/EIF2AK2/VEGFC*	9
GO:0046849	bone remodeling	10/293	90/18670	1,47E-06	1,04E-05	4,56E-06	*SRC/EGFR/ADRB2/IL6/LEPR/ITGB3/FSHB/TGFB1/TFRC/LEP*	10
GO:0071674	mononuclear cell migration	10/293	90/18670	1,47E-06	1,04E-05	4,56E-06	*AIF1/LGALS3/TNF/IL6/SERPINE1/HMGB1/C5AR1/TGFB1/FLT1/IL6R*	10
GO:0038083	peptidyl-tyrosine autophosphorylation	7/293	37/18670	1,50E-06	1,05E-05	4,64E-06	*ACE/SRC/EGFR/IGF1R/VEGFA/INSR/KDR*	7
GO:0043029	T cell homeostasis	7/293	37/18670	1,50E-06	1,05E-05	4,64E-06	*AKT1/CASP3/BAX/BCL2/TGFB1/FOXP3/TSC22D3*	7
GO:0042104	positive regulation of activated T cell proliferation	6/293	24/18670	1,51E-06	1,05E-05	4,64E-06	*HMGB1/IL12B/IGF2/IL23R/IGF1/IL6R*	6

ID	Description	GeneRatio	BgRatio	pvalue	p.adjust	qvalue	geneID	Count
GO:0060148	positive regulation of posttranscriptional gene silencing	6/293	24/18670	1,51E-06	1,05E-05	4,64E-06	*TP53/STAT3/EGFR/XPO5/TGFB1/FMR1*	6
GO:0071677	positive regulation of mononuclear cell migration	6/293	24/18670	1,51E-06	1,05E-05	4,64E-06	*AIF1/LGALS3/TNF/SERPINE1/HMGB1/TGFB1*	6
GO:1905475	regulation of protein localization to membrane	14/293	187/18670	1,58E-06	1,11E-05	4,87E-06	*TP53/AKT1/EGFR/AR/BAD/LGALS3/TNF/TP63/TP73/BCL2/BCL2L1/TGFB1/INS/IFNG*	14
GO:0007292	female gamete generation	12/293	136/18670	1,60E-06	1,12E-05	4,92E-06	*SRC/NOS3/BCL2/BRCA2/ETV6/FSHB/FSHR/PGR/IGF1/IL4R/LEP/PDE5A*	12
GO:0031638	zymogen activation	8/293	53/18670	1,61E-06	1,12E-05	4,92E-06	*BAD/FGG/CPB2/F12/FGB/FGA/PLAT/SERPINE1*	8
GO:0045840	positive regulation of mitotic nuclear division	8/293	53/18670	1,61E-06	1,12E-05	4,92E-06	*IL1B/TGFB1/EGF/INSR/INS/IGF2/IGF1/IL1A*	8
GO:0045346	regulation of MHC class II biosynthetic process	5/293	14/18670	1,64E-06	1,13E-05	5,00E-06	*TLR4/JAK2/IFNG/IL4/IL10*	5
GO:0045953	negative regulation of natural killer cell mediated cytotoxicity	5/293	14/18670	1,64E-06	1,13E-05	5,00E-06	*HAVCR2/HLA-E/HLA-G/LILRB1/CEACAM1*	5
GO:0060397	JAK-STAT cascade involved in growth hormone signaling pathway	5/293	14/18670	1,64E-06	1,13E-05	5,00E-06	*STAT3/STAT5B/JAK2/PRLR/PRL*	5
GO:0000075	cell cycle checkpoint	15/293	216/18670	1,73E-06	1,19E-05	5,26E-06	*TP53/MDM2/LCMT1/BAX/BCL2L1/APC/BUB1/BUB1B/DNA2/BUB3/BRIP1/BRCA1/MDC1/CNOT1/TGFB1*	15
GO:0035710	CD4-positive, alpha-beta T cell activation	10/293	92/18670	1,80E-06	1,24E-05	5,47E-06	*STAT3/MTOR/IL6/HMGB1/IL12B/IL23R/FOXP3/IFNG/IL12A/IL4R*	10
GO:0045833	negative regulation of lipid metabolic process	10/293	92/18670	1,80E-06	1,24E-05	5,47E-06	*NFKB1/AKT1/BMP2/ADRA2A/IL1B/TNF/APOE/BRCA1/CEACAM1/INS*	10
GO:0005976	polysaccharide metabolic process	11/293	114/18670	1,81E-06	1,24E-05	5,47E-06	*NFKB1/AKT1/MTOR/LEPR/CHIA/TGFB1/EGF/INSR/INS/IGF2/IGF1*	11
GO:0030282	bone mineralization	11/293	114/18670	1,81E-06	1,24E-05	5,47E-06	*GATA1/ACVR1/BMP2/ADRB2/HIF1A/LTF/TGFB1/COL1A2/FGFR3/IGF1/LEP*	11
GO:0002707	negative regulation of lymphocyte mediated immunity	7/293	38/18670	1,82E-06	1,24E-05	5,47E-06	*HAVCR2/HLA-E/HLA-G/LILRB1/CEACAM1/FOXP3/HFE*	7
GO:0032350	regulation of hormone metabolic process	7/293	38/18670	1,82E-06	1,24E-05	5,47E-06	*NFKB1/BMP2/IL1B/TNF/ARNT/HIF1A/IFNG*	7
GO:0060416	response to growth hormone	7/293	38/18670	1,82E-06	1,24E-05	5,47E-06	*AKT1/STAT3/STAT5B/JAK2/PRLR/F7/PRL*	7

ID	Description	GeneRatio	BgRatio	pvalue	p.adjust	qvalue	geneID	Count
GO:0060348	bone development	15/293	217/18670	1,83E-06	1,25E-05	5,51E-06	*TP53/SRC/BMP2/MBL2/LTF/LILRB1/TGFB1/COL1A1/FBN1/COL6A3/COL6A1/SULF1/FGFR3/IGF1/LEP*	15
GO:0019932	second-messenger-mediated signaling	22/293	439/18670	1,85E-06	1,26E-05	5,54E-06	*EGFR/NOS3/MTOR/ADA/ADRA2A/ADRB2/AGTR1/CCR5/TNF/AGT/AHR/PRKG1/APOE/VEGFA/FSHR/CXCL8/CXCR1/INS/KDR/IGF1/NOS2/PDE5A*	22
GO:1904645	response to amyloid-beta	8/293	54/18670	1,86E-06	1,27E-05	5,58E-06	*TLR4/IGF1R/ADRB2/MMP2/MMP12/MMP3/MMP9/IGF1*	8
GO:0002507	tolerance induction	6/293	25/18670	1,96E-06	1,32E-05	5,83E-06	*HAVCR2/HMGB1/HLA-E/HLA-G/TGFB1/FOXP3*	6
GO:0002719	negative regulation of cytokine production involved in immune response	6/293	25/18670	1,96E-06	1,32E-05	5,83E-06	*TNF/LILRB1/TGFB1/FOXP3/HFE/IL10*	6
GO:0009299	mRNA transcription	6/293	25/18670	1,96E-06	1,32E-05	5,83E-06	*TP53/STAT3/ARNT/HIF1A/FLNA/C5AR1*	6
GO:0030813	positive regulation of nucleotide catabolic process	6/293	25/18670	1,96E-06	1,32E-05	5,83E-06	*ARNT/HIF1A/INSR/INS/IFNG/IGF1*	6
GO:0051197	positive regulation of coenzyme metabolic process	6/293	25/18670	1,96E-06	1,32E-05	5,83E-06	*ARNT/HIF1A/INSR/INS/IFNG/IGF1*	6
GO:1901655	cellular response to ketone	10/293	93/18670	1,99E-06	1,34E-05	5,93E-06	*SRC/AKT1/NR3C1/EGFR/AR/PPARG/AHR/CASP9/CFTR/TGFB1*	10
GO:0000280	nuclear division	21/293	407/18670	2,01E-06	1,36E-05	5,98E-06	*HSPA1B/HSPA1A/RAN/IL1B/LCMT1/FLNA/APC/BUB1/BUB1B/SPAG5/BUB3/BAG6/BRIP1/BRCA2/TGFB1/EGF/INSR/INS/IGF2/IGF1/IL1A*	21
GO:0060485	mesenchyme development	17/293	278/18670	2,04E-06	1,38E-05	6,06E-06	*ACTA1/MDM2/NOS3/MTOR/FGFR1/FN1/ACTA2/ACVR1/TGFBR1/BMP2/IL1B/IL6/HIF1A/BCL2/TGFB1/COL1A1/EFNB1*	17
GO:0007093	mitotic cell cycle checkpoint	13/293	165/18670	2,12E-06	1,42E-05	6,27E-06	*TP53/MDM2/LCMT1/BAX/BCL2L1/APC/BUB1/BUB1B/BUB3/BRCA1/MDC1/CNOT1/TGFB1*	13
GO:0050680	negative regulation of epithelial cell proliferation	13/293	165/18670	2,12E-06	1,42E-05	6,27E-06	*AR/NR2F2/PPARG/TGFBR1/TNF/CPB2/APOE/BRCA2/CEACAM1/PRL/TGFB1/SULF1/FLT1*	13
GO:0071695	anatomical structure maturation	13/293	165/18670	2,12E-06	1,42E-05	6,27E-06	*MTOR/BMP2/FGG/HIF1A/MMP2/BCL2/TYMS/BRCA2/LTF/PGR/FGFR3/IGF1/LEP*	13
GO:0006352	DNA-templated transcription, initiation	16/293	249/18670	2,16E-06	1,44E-05	6,36E-06	*TP53/THRB/NR3C1/ESR2/AR/ESR1/PPARG/ZNRD1/HNF4A/BAX/GTF2H4/HMGB1/CREB1/RXRB/PGR/HNF1A*	16
GO:0019318	hexose metabolic process	16/293	249/18670	2,16E-06	1,44E-05	6,36E-06	*TP53/SRC/AKT1/MTOR/BAD/IGFBP3/TNF/RBP4/LEPR/LCMT1/IGFBP4/INSR/INS/IGF2/IGF1/LEP*	16
GO:0045926	negative regulation of growth	16/293	249/18670	2,16E-06	1,44E-05	6,36E-06	*TP53/ESR2/HSPA1B/HSPA1A/PPARG/ADRB2/TP73/AGT/HRG/RBP4/HIF1A/HNF4A/BCL2/MAP2/TGFB1/FGFR3*	16
GO:1901216	positive regulation of neuron death	10/293	94/18670	2,20E-06	1,47E-05	6,46E-06	*TP53/TLR4/CASP3/MTOR/BAD/TNF/CASP9/BAX/FASLG/IFNG*	10

ID	Description	GeneRatio	BgRatio	pvalue	p.adjust	qvalue	geneID	Count
GO:0016125	sterol metabolic process	13/293	166/18670	2,27E-06	1,51E-05	6,66E-06	*RAN/LEPR/APOB/APOE/NSDHL/CEBPA/CFTR/CNBP/DHCR7/EBP/FGF1/IL4/LEP*	13
GO:1901654	response to ketone	14/293	193/18670	2,30E-06	1,53E-05	6,74E-06	*SRC/AKT1/NR3C1/EGFR/AR/BAD/PPARG/AHR/CASP9/BCL2L1/TYMS/F7/CFTR/TGFB1*	14
GO:0032677	regulation of interleukin-8 production	9/293	74/18670	2,31E-06	1,53E-05	6,74E-06	*TLR4/HSPA1B/HSPA1A/IL1B/TNF/SERPINE1/CD14/TLR1/IL10*	9
GO:0043367	CD4-positive, alpha-beta T cell differentiation	9/293	74/18670	2,31E-06	1,53E-05	6,74E-06	*STAT3/MTOR/IL6/HMGB1/IL12B/IL23R/FOXP3/IFNG/IL4R*	9
GO:1900182	positive regulation of protein localization to nucleus	9/293	74/18670	2,31E-06	1,53E-05	6,74E-06	*SRC/AKT1/RAN/F2/FLNA/TGFB1/INS/IFNG/LEP*	9
GO:1900371	regulation of purine nucleotide biosynthetic process	11/293	117/18670	2,34E-06	1,55E-05	6,82E-06	*TP53/STAT3/NOS3/ARNT/HIF1A/INSR/INS/IFNG/IGF1/IL4/NOS2*	11
GO:0009411	response to UV	12/293	141/18670	2,34E-06	1,55E-05	6,82E-06	*TP53/AKT1/EGFR/MDM2/CASP3/CASP9/BAX/BCL2/BRCA2/IL12B/FMR1/IL12A*	12
GO:0002716	negative regulation of natural killer cell mediated immunity	5/293	15/18670	2,43E-06	1,60E-05	7,03E-06	*HAVCR2/HLA-E/HLA-G/LILRB1/CEACAM1*	5
GO:0032352	positive regulation of hormone metabolic process	5/293	15/18670	2,43E-06	1,60E-05	7,03E-06	*IL1B/TNF/ARNT/HIF1A/IFNG*	5
GO:0051044	positive regulation of membrane protein ectodomain proteolysis	5/293	15/18670	2,43E-06	1,60E-05	7,03E-06	*ADRA2A/IL1B/TNF/APOE/IFNG*	5
GO:0030808	regulation of nucleotide biosynthetic process	11/293	118/18670	2,55E-06	1,67E-05	7,37E-06	*TP53/STAT3/NOS3/ARNT/HIF1A/INSR/INS/IFNG/IGF1/IL4/NOS2*	11
GO:0006695	cholesterol biosynthetic process	9/293	75/18670	2,59E-06	1,70E-05	7,48E-06	*RAN/APOB/APOE/NSDHL/CFTR/CNBP/DHCR7/EBP/FGF1*	9
GO:0051302	regulation of cell division	13/293	168/18670	2,60E-06	1,70E-05	7,49E-06	*IL1B/TP63/BCL2L1/BRCA2/VEGFA/TGFB1/IGF2/FGF1/FGF7/VEGFC/VEGFB/PGF/IL1A*	13
GO:0002823	negative regulation of adaptive immune response based on somatic recombination of immune receptors built from immunoglobulin superfamily domains	7/293	40/18670	2,61E-06	1,70E-05	7,51E-06	*HAVCR2/HLA-G/LILRB1/CEACAM1/FOXP3/HFE/IL4R*	7

ID	Description	GeneRatio	BgRatio	pvalue	p.adjust	qvalue	geneID	Count
GO:0042036	negative regulation of cytokine biosynthetic process	7/293	40/18670	2,61E-06	1,70E-05	7,51E-06	*NFKB1/IL6/INHA/LILRB1/FOXP3/IL4/IL10*	7
GO:0048285	organelle fission	22/293	449/18670	2,66E-06	1,73E-05	7,64E-06	*HSPA1B/HSPA1A/RAN/IL1B/LCMT1/FLNA/APC/BUB1/BUB1B/SPAG5/BUB3/BAG6/BRIP1/BRCA2/TGFB1/EGF/INSR/INS/IGF2/KDR/IGF1/IL1A*	22
GO:0002474	antigen processing and presentation of peptide antigen via MHC class I	10/293	96/18670	2,67E-06	1,73E-05	7,64E-06	*ACE/CHUK/IKBKG/HLA-E/HLA-C/HLA-G/HLA-A/HLA-B/TAP2/HFE*	10
GO:0032755	positive regulation of interleukin-6 production	10/293	96/18670	2,67E-06	1,73E-05	7,64E-06	*AIF1/STAT3/TLR4/IL1B/TNF/IL6/HMGB1/IL1RN/TLR1/IL6R*	10
GO:0031098	stress-activated protein kinase signaling cascade	18/293	315/18670	2,68E-06	1,74E-05	7,66E-06	*NFKB1/AKT1/TLR4/EGFR/CHUK/IKBKG/IGF1R/GSTP1/BMP2/IL1B/TNF/UBE2N/AGT/HMGB1/VEGFA/EIF2AK2/IL1RN/LEP*	18
GO:0007173	epidermal growth factor receptor signaling pathway	11/293	119/18670	2,77E-06	1,79E-05	7,91E-06	*SRC/AKT1/EGFR/ADRA2A/AGT/MMP9/CEACAM1/TGFB1/IFI6/EGF/FASLG*	11
GO:0030307	positive regulation of cell growth	13/293	169/18670	2,77E-06	1,80E-05	7,91E-06	*AKT1/EGFR/MTOR/FN1/L1CAM/TGFBR1/F2/IGFBP1/BCL2/VEGFA/INS/HAMP/IGF1*	13
GO:0050851	antigen receptor-mediated signaling pathway	18/293	316/18670	2,80E-06	1,81E-05	7,97E-06	*NFKB1/CHUK/IKBKG/LGALS3/ADA/UBE2N/BAX/BCL2/CEACAM1/CTLA4/FOXP3/HLA-DQA1/HLA-DRB5/HLA-DRA/HLA-DQB2/HLA-DRB1/HLA-DQB1/HLA-DQA2*	18
GO:0001649	osteoblast differentiation	15/293	225/18670	2,87E-06	1,85E-05	8,16E-06	*AKT1/IGFBP3/ACVR1/BMP2/TNF/IL6/TP63/LTF/CEBPA/CEBPB/COL1A1/COL6A1/IGF2/IGF1/IL6R*	15
GO:2000146	negative regulation of cell motility	19/293	349/18670	2,87E-06	1,85E-05	8,16E-06	*AIF1/AKT1/STAT3/NR2F2/IGFBP3/PPARG/GSTP1/ADA/HRG/PRKG1/BCL2/APOE/TIMP1/SERPINE1/HMGB1/TGFB1/FBLN1/SULF1/IL4*	19
GO:0046902	regulation of mitochondrial membrane permeability	9/293	76/18670	2,90E-06	1,86E-05	8,21E-06	*TP53/STAT3/BAD/HSPA1A/TP63/TP73/BAX/BCL2/BCL2L1*	9
GO:1902653	secondary alcohol biosynthetic process	9/293	76/18670	2,90E-06	1,86E-05	8,21E-06	*RAN/APOB/APOE/NSDHL/CFTR/CNBP/DHCR7/EBP/FGF1*	9
GO:0051271	negative regulation of cellular component movement	20/293	384/18670	3,08E-06	1,98E-05	8,70E-06	*AIF1/AKT1/STAT3/NR2F2/IGFBP3/PPARG/GSTP1/ADA/HRG/PRKG1/BCL2/APOE/TIMP1/SERPINE1/HMGB1/CD9/TGFB1/FBLN1/SULF1/IL4*	20
GO:0150077	regulation of neuroinflammatory response	7/293	41/18670	3,11E-06	1,99E-05	8,78E-06	*IL1B/TNF/IL6/MMP3/MMP9/IGF1/IL4*	7
GO:0048143	astrocyte activation	6/293	27/18670	3,18E-06	2,04E-05	8,98E-06	*EGFR/IL1B/TNF/IL6/C5AR1/IFNG*	6

ID	Description	GeneRatio	BgRatio	pvalue	p.adjust	qvalue	geneID	Count
GO:0002218	activation of innate immune response	18/293	319/18670	3,20E-06	2,04E-05	9,01E-06	*NFKB1/SRC/TLR4/CHUK/ESR1/IKBKG/HSPA1B/HSPA1A/UBE2N/FGG/FGB/FGA/APOB/HAVCR2/HMGB1/CD14/LTF/TLR1*	18
GO:0050764	regulation of phagocytosis	10/293	98/18670	3,22E-06	2,06E-05	9,07E-06	*GATA2/PPARG/IL1B/TNF/MBL2/HMGB1/SLC11A1/ITGA2/TGFB1/IFNG*	10
GO:1901224	positive regulation of NIK/NF-kappaB signaling	9/293	77/18670	3,24E-06	2,06E-05	9,08E-06	*TLR4/EGFR/IL1B/TNF/HAVCR2/HMGB1/CD14/IL12B/EIF2AK2*	9
GO:0031663	lipopolysaccharide-mediated signaling pathway	8/293	58/18670	3,25E-06	2,06E-05	9,08E-06	*AKT1/TLR4/NOS3/IL1B/TNF/CD14/LTF/TGFB1*	8
GO:0042093	T-helper cell differentiation	8/293	58/18670	3,25E-06	2,06E-05	9,08E-06	*STAT3/MTOR/IL6/HMGB1/IL12B/IL23R/FOXP3/IL4R*	8
GO:0061900	glial cell activation	8/293	58/18670	3,25E-06	2,06E-05	9,08E-06	*AIF1/EGFR/JAK2/IL1B/TNF/IL6/C5AR1/IFNG*	8
GO:0035264	multicellular organism growth	12/293	146/18670	3,38E-06	2,14E-05	9,43E-06	*TP53/STAT3/STAT5B/MTOR/AR/ADRB2/BCL2/CREB1/PRL/DHCR7/IGF2/IGF1*	12
GO:0055123	digestive system development	12/293	146/18670	3,38E-06	2,14E-05	9,43E-06	*EGFR/ADA/TNF/TP63/HIF1A/BCL2/TYMS/ITGB4/TGFB1/CXCL8/INSR/IGF2*	12
GO:0002295	T-helper cell lineage commitment	5/293	16/18670	3,49E-06	2,21E-05	9,72E-06	*STAT3/MTOR/IL6/IL12B/IL23R*	5
GO:1905477	positive regulation of protein localization to membrane	11/293	122/18670	3,54E-06	2,23E-05	9,84E-06	*TP53/AKT1/EGFR/BAD/LGALS3/TNF/TP63/TP73/BCL2/TGFB1/IFNG*	11
GO:0000271	polysaccharide biosynthetic process	9/293	78/18670	3,61E-06	2,28E-05	1,00E-05	*NFKB1/AKT1/MTOR/TGFB1/EGF/INSR/INS/IGF2/IGF1*	9
GO:0034330	cell junction organization	17/293	290/18670	3,62E-06	2,28E-05	1,00E-05	*ACE/ACE2/SRC/FN1/TGFBR1/TNF/AGT/HRG/FLNA/BCL2/APC/ITGB4/CD9/ITGA2/VEGFA/TGFB1/KDR*	17
GO:0002639	positive regulation of immunoglobulin production	7/293	42/18670	3,68E-06	2,31E-05	1,02E-05	*IL6/RBP4/HLA-E/TGFB1/TFRC/IL4R/IL4*	7
GO:0010518	positive regulation of phospholipase activity	8/293	59/18670	3,70E-06	2,32E-05	1,02E-05	*EGFR/FGFR1/ESR1/AGTR1/AGT/C5AR1/FGFR3/FLT1*	8
GO:0045824	negative regulation of innate immune response	8/293	59/18670	3,70E-06	2,32E-05	1,02E-05	*PPARG/MMP12/HAVCR2/HLA-E/HLA-G/LILRB1/CEACAM1/INS*	8
GO:0097755	positive regulation of blood vessel diameter	8/293	59/18670	3,70E-06	2,32E-05	1,02E-05	*EGFR/NOS3/ADRB2/AGT/PRKG1/APOE/INS/LEP*	8
GO:0006959	humoral immune response	19/293	356/18670	3,83E-06	2,40E-05	1,06E-05	*IL1B/TNF/IL6/F2/HRG/MBL2/BCL2/CPB2/FGB/FGA/C5AR1/SLC11A1/LTF/HLA-E/LCN2/CXCL8/IFNG/HLA-DQB1/LTA*	19
GO:0022412	cellular process involved in reproduction in	19/293	357/18670	3,99E-06	2,49E-05	1,10E-05	*SRC/AKT1/MTOR/ACVR1/TGFBR1/BAX/BCL2/BCL2L1/BRIP1/BRCA2/ETV6/CTCFL/CD9/CFTR/FSHB/FSHR/TGFB1/IGF1/PDE5A*	19

ID	Description	GeneRatio	BgRatio	pvalue	p.adjust	qvalue	geneID	Count
	multicellular organism							
GO:0001773	myeloid dendritic cell activation	6/293	28/18670	4,00E-06	2,49E-05	1,10E-05	*HAVCR2/UBD/HMGB1/TGFB1/IL4/IL10*	6
GO:0032647	regulation of interferon-alpha production	6/293	28/18670	4,00E-06	2,49E-05	1,10E-05	*TLR4/CHUK/MMP12/HAVCR2/HMGB1/IL10*	6
GO:0055072	iron ion homeostasis	9/293	79/18670	4,02E-06	2,50E-05	1,10E-05	*TMPRSS6/HEPH/HIF1A/SLC11A1/LTF/LCN2/HAMP/HFE/TFRC*	9
GO:0015850	organic hydroxy compound transport	16/293	262/18670	4,17E-06	2,59E-05	1,14E-05	*NFKB1/PPARG/ADRA2A/AGTR1/AGT/REN/RBP4/HTR1A/APOB/APOE/ITGB3/LILRB1/CEACAM1/CFTR/EGF/LEP*	16
GO:0032459	regulation of protein oligomerization	7/293	43/18670	4,33E-06	2,69E-05	1,19E-05	*TP53/SRC/BAX/APOE/MMP3/MMP1/INS*	7
GO:0048738	cardiac muscle tissue development	15/293	233/18670	4,40E-06	2,73E-05	1,20E-05	*PIM1/MTOR/FGFR1/ACVR1/TGFBR1/BMP2/TP73/AGT/RBP4/VEGFA/CREB1/TGFB1/NEB/HAMP/IGF1*	15
GO:0034764	positive regulation of transmembrane transport	14/293	204/18670	4,40E-06	2,73E-05	1,20E-05	*ABCB1/AKT1/TRPC6/ADRB2/AGT/F2/FLNA/BAX/CFTR/INSR/INS/IFNG/IGF1/TRPC1*	14
GO:0010506	regulation of autophagy	18/293	327/18670	4,52E-06	2,79E-05	1,23E-05	*TP53/AKT1/STAT3/CASP3/MTOR/BAD/IKBKG/ADRB2/IL10RA/LEPR/HIF1A/BCL2/HMGB1/KDR/IFNG/IL4/LEP/IL10*	18
GO:0010959	regulation of metal ion transport	20/293	394/18670	4,53E-06	2,80E-05	1,23E-05	*AKT1/TRPC6/NOS3/LGALS3/ADRA2A/ADRB2/AGT/F2/IL16/FLNA/BAX/BCL2/LILRB1/TGFB1/EGF/FMR1/IFNG/HAMP/HFE/TRPC1*	20
GO:0019058	viral life cycle	18/293	328/18670	4,71E-06	2,91E-05	1,28E-05	*ACE2/EGFR/HSPA1B/NCAM1/HSPA1A/RAN/CCR5/TNF/BCL2/APOE/ITGB6/ITGB3/LTF/ITGA2/CXCL8/EIF2AK2/FMR1/TFRC*	18
GO:0010803	regulation of tumor necrosis factor-mediated signaling pathway	8/293	61/18670	4,79E-06	2,95E-05	1,30E-05	*TRAF1/CHUK/IKBKG/TNFRSF1A/HSPA1B/HSPA1A/GSTP1/TNF*	8
GO:1905953	negative regulation of lipid localization	8/293	61/18670	4,79E-06	2,95E-05	1,30E-05	*NFKB1/AKT1/PPARG/TNF/IL6/ITGB3/EGF/LEP*	8
GO:0045725	positive regulation of glycogen biosynthetic process	5/293	17/18670	4,88E-06	3,00E-05	1,32E-05	*AKT1/INSR/INS/IGF2/IGF1*	5
GO:0016126	sterol biosynthetic process	9/293	81/18670	4,96E-06	3,04E-05	1,34E-05	*RAN/APOB/APOE/NSDHL/CFTR/CNBP/DHCR7/EBP/FGF1*	9
GO:0005979	regulation of glycogen biosynthetic process	6/293	29/18670	4,98E-06	3,05E-05	1,34E-05	*AKT1/MTOR/INSR/INS/IGF2/IGF1*	6
GO:0010962	regulation of glucan biosynthetic process	6/293	29/18670	4,98E-06	3,05E-05	1,34E-05	*AKT1/MTOR/INSR/INS/IGF2/IGF1*	6

ID	Description	GeneRatio	BgRatio	pvalue	p.adjust	qvalue	geneID	Count
GO:0038084	vascular endothelial growth factor signaling pathway	7/293	44/18670	5,09E-06	3,10E-05	1,37E-05	*HRG/VEGFA/KDR/VEGFC/FLT1/VEGFB/PGF*	7
GO:0044060	regulation of endocrine process	7/293	44/18670	5,09E-06	3,10E-05	1,37E-05	*FGFR1/IL1B/AGTR1/AGT/REN/INHA/LEP*	7
GO:1901028	regulation of mitochondrial outer membrane permeabilization involved in apoptotic signaling pathway	7/293	44/18670	5,09E-06	3,10E-05	1,37E-05	*TP53/BAD/HSPA1A/TP63/TP73/BAX/BCL2*	7
GO:0000187	activation of MAPK activity	12/293	152/18670	5,14E-06	3,12E-05	1,38E-05	*TLR4/IKBKG/BMP2/IL1B/TNF/UBE2N/TP73/C5AR1/EGF/INSR/FGF1/IGF1*	12
GO:0016202	regulation of striated muscle tissue development	12/293	152/18670	5,14E-06	3,12E-05	1,38E-05	*PIM1/MTOR/FGFR1/TGFBR1/BMP2/TP73/RBP4/BCL2/CREB1/TGFB1/HAMP/IGF1*	12
GO:0030073	insulin secretion	14/293	207/18670	5,22E-06	3,17E-05	1,40E-05	*BAD/JAK2/ADRA2A/IL1B/TNF/RBP4/HIF1A/HNF4A/CFTR/IFNG/HNF1A/IL1RN/LEP/NOS2*	14
GO:0032368	regulation of lipid transport	11/293	127/18670	5,23E-06	3,17E-05	1,40E-05	*NFKB1/AKT1/PPARG/IL1B/AGTR1/AGT/REN/APOE/ITGB3/EGF/LEP*	11
GO:0035270	endocrine system development	11/293	127/18670	5,23E-06	3,17E-05	1,40E-05	*AKT1/THRB/GATA2/BAD/TGFBR1/BMP2/IL6/HNF4A/CREB1/INSR/IL6R*	11
GO:0007050	cell cycle arrest	15/293	237/18670	5,42E-06	3,27E-05	1,44E-05	*TP53/MDM2/MTOR/TGFBR1/TP73/BAX/INHA/APC/BRCA1/CNOT1/TGFB1/IL12B/CXCL8/IFNG/IL12A*	15
GO:0031640	killing of cells of other organism	8/293	62/18670	5,42E-06	3,27E-05	1,44E-05	*BAD/F2/HRG/MBL2/LTF/IFNG/HAMP/NOS2*	8
GO:0044364	disruption of cells of other organism	8/293	62/18670	5,42E-06	3,27E-05	1,44E-05	*BAD/F2/HRG/MBL2/LTF/IFNG/HAMP/NOS2*	8
GO:0046824	positive regulation of nucleocytoplasmic transport	8/293	62/18670	5,42E-06	3,27E-05	1,44E-05	*TP53/MDM2/RAN/IL1B/FLNA/TGFB1/IFNG/LEP*	8
GO:0007623	circadian rhythm	14/293	208/18670	5,52E-06	3,32E-05	1,46E-05	*TP53/EGFR/MTOR/PPARG/ADA/AHR/HNF4A/TYMS/F7/SERPINE1/CREB1/CRTC1/LEP/NOS2*	14
GO:0010876	lipid localization	20/293	400/18670	5,67E-06	3,41E-05	1,50E-05	*ABCB1/ACE/NFKB1/AKT1/PPARG/IL1B/AGTR1/TNF/IL6/AGT/REN/RBP4/APOB/APOE/ITGB3/CEACAM1/CFTR/EGF/LEP/NOS2*	20
GO:0002820	negative regulation of adaptive immune response	7/293	45/18670	5,94E-06	3,56E-05	1,57E-05	*HAVCR2/HLA-G/LILRB1/CEACAM1/FOXP3/HFE/IL4R*	7
GO:0035987	endodermal cell differentiation	7/293	45/18670	5,94E-06	3,56E-05	1,57E-05	*FN1/MMP2/MMP9/COL5A1/COL5A2/COL6A1/MMP15*	7
GO:0051402	neuron apoptotic process	15/293	239/18670	6,00E-06	3,59E-05	1,58E-05	*TP53/THRB/CASP3/JAK2/TNF/TP63/HIF1A/CASP9/BAX/BCL2/BCL2L1/APOE/C5AR1/CEBPB/FASLG*	15

ID	Description	GeneRatio	BgRatio	pvalue	p.adjust	qvalue	geneID	Count
GO:0030336	negative regulation of cell migration	18/293	334/18670	6,05E-06	3,62E-05	1,60E-05	*AIF1/AKT1/STAT3/NR2F2/IGFBP3/PPARG/GSTP1/ADA/HRG/PRKG1/BCL2/APOE/TIMP1/SERPINE1/HMGB1/TGFB1/SULF1/IL4*	18
GO:0006970	response to osmotic stress	9/293	83/18670	6,08E-06	3,63E-05	1,60E-05	*ABCB1/TP53/EGFR/CASP3/BAD/TNF/BAX/ITGA2/TSC22D3*	9
GO:2000106	regulation of leukocyte apoptotic process	9/293	83/18670	6,08E-06	3,63E-05	1,60E-05	*TP53/LGALS3/ADA/CCR5/HIF1A/BAX/LILRB1/TSC22D3/IL10*	9
GO:0060249	anatomical structure homeostasis	21/293	437/18670	6,09E-06	3,63E-05	1,60E-05	*SRC/TLR4/EGFR/NOS3/GATA2/GATA1/ADRB2/IL6/RBP4/HIF1A/BAX/BCL2/DNA2/ITGB3/BRCA2/RFC1/LTF/VEGFA/FSHB/TGFB1/TFRC*	21
GO:0001782	B cell homeostasis	6/293	30/18670	6,14E-06	3,64E-05	1,60E-05	*CASP3/ADA/HIF1A/BAX/BCL2/FOXP3*	6
GO:0001844	protein insertion into mitochondrial membrane involved in apoptotic signaling pathway	6/293	30/18670	6,14E-06	3,64E-05	1,60E-05	*TP53/BAD/TP63/TP73/BAX/BCL2*	6
GO:0010743	regulation of macrophage derived foam cell differentiation	6/293	30/18670	6,14E-06	3,64E-05	1,60E-05	*NFKB1/PPARG/AGTR1/AGT/APOB/ITGB3*	6
GO:0032607	interferon-alpha production	6/293	30/18670	6,14E-06	3,64E-05	1,60E-05	*TLR4/CHUK/MMP12/HAVCR2/HMGB1/IL10*	6
GO:1901861	regulation of muscle tissue development	12/293	155/18670	6,28E-06	3,72E-05	1,64E-05	*PIM1/MTOR/FGFR1/TGFBR1/BMP2/TP73/RBP4/BCL2/CREB1/TGFB1/HAMP/IGF1*	12
GO:1903531	negative regulation of secretion by cell	14/293	211/18670	6,51E-06	3,85E-05	1,70E-05	*TNFRSF1A/FN1/ADRA2A/IL1B/TNF/INHA/APOE/LILRB1/CEACAM1/INS/FMR1/FOXP3/LEP/IL10*	14
GO:0034329	cell junction assembly	15/293	241/18670	6,63E-06	3,91E-05	1,72E-05	*ACE/ACE2/SRC/FN1/TNF/AGT/HRG/FLNA/BCL2/APC/ITGB4/CD9/ITGA2/VEGFA/KDR*	15
GO:0002040	sprouting angiogenesis	13/293	183/18670	6,66E-06	3,91E-05	1,72E-05	*AKT1/GATA2/AGTR1/CEACAM1/VEGFA/EPHB4/FGF1/KDR/VEGFC/FLT1/VEGFB/PGF/IL10*	13
GO:0001911	negative regulation of leukocyte mediated cytotoxicity	5/293	18/18670	6,67E-06	3,91E-05	1,72E-05	*HAVCR2/HLA-E/HLA-G/LILRB1/CEACAM1*	5
GO:0060749	mammary gland alveolus development	5/293	18/18670	6,67E-06	3,91E-05	1,72E-05	*AR/ESR1/HIF1A/VEGFA/EGF*	5
GO:0061377	mammary gland lobule development	5/293	18/18670	6,67E-06	3,91E-05	1,72E-05	*AR/ESR1/HIF1A/VEGFA/EGF*	5
GO:0070875	positive regulation of glycogen metabolic process	5/293	18/18670	6,67E-06	3,91E-05	1,72E-05	*AKT1/INSR/INS/IGF2/IGF1*	5
GO:0032868	response to insulin	16/293	272/18670	6,71E-06	3,92E-05	1,73E-05	*SRC/AKT1/MTOR/IGF1R/PPARG/GSTP1/IL1B/AGT/IGFBP1/APC/CEACAM1/INSR/INS/IGF2/LEP/IL10*	16

ID	Description	GeneRatio	BgRatio	pvalue	p.adjust	qvalue	geneID	Count
GO:0010507	negative regulation of autophagy	9/293	84/18670	6,71E-06	3,92E-05	1,73E-05	*TP53/AKT1/STAT3/MTOR/IL10RA/LEPR/BCL2/LEP/IL10*	9
GO:0030856	regulation of epithelial cell differentiation	12/293	156/18670	6,71E-06	3,92E-05	1,73E-05	*BAD/TNFRSF1A/IL1B/TNF/TP63/PROC/SERPINE1/MMP9/CEACAM1/CEBPB/VEGFA/IFNG*	12
GO:0048634	regulation of muscle organ development	12/293	156/18670	6,71E-06	3,92E-05	1,73E-05	*PIM1/MTOR/FGFR1/TGFBR1/BMP2/TP73/RBP4/BCL2/CREB1/TGFB1/HAMP/IGF1*	12
GO:0060135	maternal process involved in female pregnancy	8/293	64/18670	6,91E-06	4,02E-05	1,77E-05	*AKT1/MTOR/AR/NR2F2/ESR1/HAVCR2/MMP7/PGR*	8
GO:0002437	inflammatory response to antigenic stimulus	7/293	46/18670	6,92E-06	4,02E-05	1,77E-05	*TNF/SERPINC1/HMGB1/HLA-E/IL12B/LTA/IL10*	7
GO:0010883	regulation of lipid storage	7/293	46/18670	6,92E-06	4,02E-05	1,77E-05	*NFKB1/PPARG/TNF/IL6/APOB/ITGB3/LEP*	7
GO:0071675	regulation of mononuclear cell migration	7/293	46/18670	6,92E-06	4,02E-05	1,77E-05	*AIF1/LGALS3/TNF/SERPINE1/HMGB1/C5AR1/TGFB1*	7
GO:0045930	negative regulation of mitotic cell cycle	18/293	338/18670	7,13E-06	4,14E-05	1,82E-05	*TP53/EGFR/MDM2/TNF/LCMT1/BAX/BCL2/BCL2L1/APC/BUB1/BUB1B/BUB3/BRCA1/MDC1/CNOT1/TGFB1/TIMP2/IL10*	18
GO:0045742	positive regulation of epidermal growth factor receptor signaling pathway	6/293	31/18670	7,51E-06	4,35E-05	1,92E-05	*AKT1/ADRA2A/AGT/MMP9/EGF/FASLG*	6
GO:0030518	intracellular steroid hormone receptor signaling pathway	11/293	132/18670	7,59E-06	4,39E-05	1,93E-05	*SRC/NR3C1/ESR2/AR/ESR1/JAK2/RAN/TP63/BRCA1/CNOT1/PGR*	11
GO:0042177	negative regulation of protein catabolic process	11/293	132/18670	7,59E-06	4,39E-05	1,93E-05	*EGFR/FLNA/TIMP1/TIMP3/BAG6/TIMP4/TIMP2/INS/HFE/NOS2/IL10*	11
GO:0045732	positive regulation of protein catabolic process	14/293	214/18670	7,65E-06	4,42E-05	1,95E-05	*AKT1/MDM2/HSPA1B/HSPA1A/ADRA2A/IL1B/TNF/APC/APOE/BAG6/CEBPA/EGF/FMR1/IFNG*	14
GO:0014911	positive regulation of smooth muscle cell migration	7/293	47/18670	8,02E-06	4,61E-05	2,03E-05	*AIF1/SRC/MDM2/AGT/BCL2/ITGA2/IGF1*	7
GO:0045058	T cell selection	7/293	47/18670	8,02E-06	4,61E-05	2,03E-05	*STAT3/MTOR/IL6/BCL2/IL12B/IL23R/FOXP3*	7
GO:0050435	amyloid-beta metabolic process	7/293	47/18670	8,02E-06	4,61E-05	2,03E-05	*ACE/CASP3/TNF/REN/APOE/IFNG/IGF1*	7
GO:0060986	endocrine hormone secretion	7/293	47/18670	8,02E-06	4,61E-05	2,03E-05	*FGFR1/IL1B/AGTR1/AGT/REN/INHA/LEP*	7
GO:0030101	natural killer cell activation	9/293	86/18670	8,16E-06	4,68E-05	2,06E-05	*BAG6/HAVCR2/HLA-E/IL12B/IL23R/HNF1A/IL12A/LEP/IL6R*	9

ID	Description	GeneRatio	BgRatio	pvalue	p.adjust	qvalue	geneID	Count
GO:0042058	regulation of epidermal growth factor receptor signaling pathway	9/293	86/18670	8,16E-06	4,68E-05	2,06E-05	*AKT1/EGFR/ADRA2A/AGT/MMP9/CEACAM1/IFI6/EGF/FASLG*	9
GO:0032869	cellular response to insulin stimulus	14/293	216/18670	8,51E-06	4,87E-05	2,15E-05	*SRC/AKT1/IGF1R/PPARG/GSTP1/IL1B/AGT/IGFBP1/APC/CEACAM1/INSR/INS/IGF2/LEP*	14
GO:0040014	regulation of multicellular organism growth	8/293	66/18670	8,73E-06	4,99E-05	2,20E-05	*STAT3/STAT5B/ADRB2/BCL2/CREB1/PRL/IGF2/IGF1*	8
GO:0051785	positive regulation of nuclear division	8/293	66/18670	8,73E-06	4,99E-05	2,20E-05	*IL1B/TGFB1/EGF/INSR/INS/IGF2/IGF1/IL1A*	8
GO:0042113	B cell activation	17/293	310/18670	8,76E-06	5,00E-05	2,20E-05	*TP53/TLR4/CASP3/BAD/CD320/ADA/IL6/AHR/BAX/BCL2/INHA/TGFB1/CTLA4/FOXP3/TFRC/IL4/IL10*	17
GO:0009314	response to radiation	21/293	448/18670	8,90E-06	5,07E-05	2,23E-05	*TP53/AKT1/EGFR/MDM2/CASP3/MTOR/HIF1A/CASP9/BAX/BCL2/BCL2L1/BRCA1/BRCA2/CREB1/CRTC1/TGFB1/IL12B/THBD/FMR1/HAMP/IL12A*	21
GO:0002710	negative regulation of T cell mediated immunity	5/293	19/18670	8,93E-06	5,07E-05	2,23E-05	*HLA-G/LILRB1/CEACAM1/FOXP3/HFE*	5
GO:0010888	negative regulation of lipid storage	5/293	19/18670	8,93E-06	5,07E-05	2,23E-05	*PPARG/TNF/IL6/ITGB3/LEP*	5
GO:0042789	mRNA transcription by RNA polymerase II	5/293	19/18670	8,93E-06	5,07E-05	2,23E-05	*STAT3/ARNT/HIF1A/FLNA/C5AR1*	5
GO:0060252	positive regulation of glial cell proliferation	5/293	19/18670	8,93E-06	5,07E-05	2,23E-05	*MTOR/IL1B/TNF/IL6/LTA*	5
GO:0071295	cellular response to vitamin	6/293	32/18670	9,12E-06	5,17E-05	2,28E-05	*PIM1/MDM2/PPARG/BRIP1/COL1A1/LEP*	6
GO:0035272	exocrine system development	7/293	48/18670	9,26E-06	5,23E-05	2,31E-05	*EGFR/FGFR1/TNF/TGFB1/INSR/IGF2/FGF7*	7
GO:0090199	regulation of release of cytochrome c from mitochondria	7/293	48/18670	9,26E-06	5,23E-05	2,31E-05	*TP53/AKT1/BAD/BAX/BCL2L1/MMP9/IGF1*	7
GO:0009791	post-embryonic development	9/293	88/18670	9,86E-06	5,57E-05	2,45E-05	*MTOR/TGFBR1/BAX/BCL2/APOB/VEGFA/FBN1/DHCR7/IGF2R*	9
GO:0031623	receptor internalization	10/293	111/18670	9,92E-06	5,59E-05	2,46E-05	*ITGB3/LILRB1/CD9/CEACAM1/VEGFA/CXCL8/CXCR1/EGF/FMR1/TFRC*	10
GO:0002704	negative regulation of leukocyte mediated immunity	7/293	49/18670	1,07E-05	6,00E-05	2,64E-05	*HAVCR2/HLA-E/HLA-G/LILRB1/CEACAM1/FOXP3/HFE*	7
GO:0003044	regulation of systemic arterial blood pressure	7/293	49/18670	1,07E-05	6,00E-05	2,64E-05	*ACE/ACE2/NOS3/ADRB2/AGTR1/AGT/REN*	7

ID	Description	GeneRatio	BgRatio	pvalue	p.adjust	qvalue	geneID	Count
	mediated by a chemical signal							
GO:0033627	cell adhesion mediated by integrin	8/293	68/18670	1,09E-05	6,13E-05	2,70E-05	*ADA/HRG/ITGB4/ITGB6/ITGB3/SERPINE1/ITGA2/FBN1*	8
GO:0035924	cellular response to vascular endothelial growth factor stimulus	8/293	68/18670	1,09E-05	6,13E-05	2,70E-05	*AKT1/HRG/VEGFA/KDR/VEGFC/FLT1/VEGFB/PGF*	8
GO:0016486	peptide hormone processing	6/293	33/18670	1,10E-05	6,15E-05	2,71E-05	*ACE/ACE2/REN/CGA/LHB/FSHB*	6
GO:0051194	positive regulation of cofactor metabolic process	6/293	33/18670	1,10E-05	6,15E-05	2,71E-05	*ARNT/HIF1A/INSR/INS/IFNG/IGF1*	6
GO:0051204	protein insertion into mitochondrial membrane	6/293	33/18670	1,10E-05	6,15E-05	2,71E-05	*TP53/BAD/TP63/TP73/BAX/BCL2*	6
GO:1901186	positive regulation of ERBB signaling pathway	6/293	33/18670	1,10E-05	6,15E-05	2,71E-05	*AKT1/ADRA2A/AGT/MMP9/EGF/FASLG*	6
GO:0001787	natural killer cell proliferation	4/293	10/18670	1,16E-05	6,44E-05	2,84E-05	*HLA-E/IL12B/IL23R/LEP*	4
GO:0010749	regulation of nitric oxide mediated signal transduction	4/293	10/18670	1,16E-05	6,44E-05	2,84E-05	*EGFR/VEGFA/INS/PDE5A*	4
GO:0051133	regulation of NK T cell activation	4/293	10/18670	1,16E-05	6,44E-05	2,84E-05	*IL12B/IL23R/IL12A/IL6R*	4
GO:0072641	type I interferon secretion	4/293	10/18670	1,16E-05	6,44E-05	2,84E-05	*CHUK/MMP12/HMGB1/LILRB1*	4
GO:0032816	positive regulation of natural killer cell activation	5/293	20/18670	1,18E-05	6,51E-05	2,87E-05	*HLA-E/IL12B/IL23R/IL12A/IL6R*	5
GO:0051767	nitric-oxide synthase biosynthetic process	5/293	20/18670	1,18E-05	6,51E-05	2,87E-05	*TLR4/JAK2/GSTP1/KDR/IFNG*	5
GO:0051769	regulation of nitric-oxide synthase biosynthetic process	5/293	20/18670	1,18E-05	6,51E-05	2,87E-05	*TLR4/JAK2/GSTP1/KDR/IFNG*	5
GO:2001242	regulation of intrinsic apoptotic signaling pathway	12/293	165/18670	1,19E-05	6,58E-05	2,90E-05	*TP53/SRC/AKT1/MDM2/BAD/HSPA1A/HIF1A/BAX/BCL2/ BCL2L1/MMP9/INS*	12
GO:0045444	fat cell differentiation	14/293	223/18670	1,22E-05	6,74E-05	2,97E-05	*AKT1/GATA2/MTOR/PPARG/BMP2/ADRB2/TNF/IL6/CEB PA/CEBPB/CREB1/TGFB1/INS/LEP*	14
GO:0001706	endoderm formation	7/293	50/18670	1,22E-05	6,74E-05	2,97E-05	*FN1/MMP2/MMP9/COL5A1/COL5A2/COL6A1/MMP15*	7
GO:1903793	positive regulation of anion transport	7/293	50/18670	1,22E-05	6,74E-05	2,97E-05	*ABCB1/ACE2/IL1B/AGT/APOE/CEBPB/CFTR*	7

ID	Description	GeneRatio	BgRatio	pvalue	p.adjust	qvalue	geneID	Count
GO:0002831	regulation of response to biotic stimulus	11/293	139/18670	1,24E-05	6,85E-05	3,02E-05	*IL1B/HRG/MMP12/HAVCR2/HMGB1/LTF/LILRB1/CEACAM1/IL12B/IL23R/IL12A*	11
GO:0032612	interleukin-1 production	10/293	115/18670	1,36E-05	7,45E-05	3,29E-05	*TLR4/JAK2/GSTP1/IL1B/HAVCR2/HMGB1/CEACAM1/IFNG/IGF1/IL10*	10
GO:0010517	regulation of phospholipase activity	8/293	70/18670	1,36E-05	7,45E-05	3,29E-05	*EGFR/FGFR1/ESR1/AGTR1/AGT/C5AR1/FGFR3/FLT1*	8
GO:0050766	positive regulation of phagocytosis	8/293	70/18670	1,36E-05	7,45E-05	3,29E-05	*GATA2/PPARG/IL1B/TNF/MBL2/SLC11A1/ITGA2/IFNG*	8
GO:0042119	neutrophil activation	22/293	498/18670	1,37E-05	7,53E-05	3,32E-05	*NFKB1/PLD1/LGALS3/HSPA1B/HSPA1A/GSTP1/PA2G4/SERPINA1/MMP9/HMGB1/C5AR1/SLC11A1/CD14/LTF/CEACAM1/CEACAM6/LCN2/TIMP2/CXCL8/CXCR1/HP/IGF2R*	22
GO:0008286	insulin receptor signaling pathway	11/293	141/18670	1,42E-05	7,80E-05	3,44E-05	*SRC/AKT1/IGF1R/IL1B/AGT/IGFBP1/APC/INSR/INS/IGF2/LEP*	11
GO:0038127	ERBB signaling pathway	11/293	142/18670	1,52E-05	8,31E-05	3,66E-05	*SRC/AKT1/EGFR/ADRA2A/AGT/MMP9/CEACAM1/TGFB1/IFI6/EGF/FASLG*	11
GO:1900542	regulation of purine nucleotide metabolic process	11/293	142/18670	1,52E-05	8,31E-05	3,66E-05	*TP53/STAT3/NOS3/ARNT/HIF1A/INSR/INS/IFNG/IGF1/IL4/NOS2*	11
GO:0035162	embryonic hemopoiesis	5/293	21/18670	1,52E-05	8,31E-05	3,66E-05	*GATA2/GATA1/HIF1A/VEGFA/KDR*	5
GO:0003073	regulation of systemic arterial blood pressure	9/293	93/18670	1,55E-05	8,44E-05	3,72E-05	*ACE/ACE2/NOS3/AR/NR2F2/ADRB2/AGTR1/AGT/REN*	9
GO:1901184	regulation of ERBB signaling pathway	9/293	93/18670	1,55E-05	8,44E-05	3,72E-05	*AKT1/EGFR/ADRA2A/AGT/MMP9/CEACAM1/IFI6/EGF/FASLG*	9
GO:0003007	heart morphogenesis	15/293	259/18670	1,57E-05	8,48E-05	3,74E-05	*PIM1/TP53/MDM2/NOS3/MTOR/ACVR1/TGFBR1/BMP2/RBP4/HIF1A/VEGFA/TGFB1/COL5A1/EPHB4/INSR*	15
GO:0090257	regulation of muscle system process	15/293	259/18670	1,57E-05	8,48E-05	3,74E-05	*ACE2/AIF1/NOS3/MTOR/TNFRSF1A/ADA/ADRA2A/ADRB2/AGT/FLNA/PRKG1/ITGA2/HAMP/IGF1/PDE5A*	15
GO:0002701	negative regulation of production of molecular mediator of immune response	6/293	35/18670	1,57E-05	8,48E-05	3,74E-05	*TNF/LILRB1/TGFB1/FOXP3/HFE/IL10*	6
GO:0032735	positive regulation of interleukin-12 production	6/293	35/18670	1,57E-05	8,48E-05	3,74E-05	*TLR4/HMGB1/HLA-G/IL12B/IL23R/IFNG*	6
GO:0070873	regulation of glycogen metabolic process	6/293	35/18670	1,57E-05	8,48E-05	3,74E-05	*AKT1/MTOR/INSR/INS/IGF2/IGF1*	6
GO:0110111	negative regulation of animal organ morphogenesis	6/293	35/18670	1,57E-05	8,48E-05	3,74E-05	*THRB/TNF/BCL2/CPB2/CEACAM1/SULF1*	6

ID	Description	GeneRatio	BgRatio	pvalue	p.adjust	qvalue	geneID	Count
GO:1901030	positive regulation of mitochondrial outer membrane permeabilization involved in apoptotic signaling pathway	6/293	35/18670	1,57E-05	8,48E-05	3,74E-05	*TP53/BAD/TP63/TP73/BAX/BCL2*	6
GO:0010822	positive regulation of mitochondrion organization	10/293	117/18670	1,58E-05	8,52E-05	3,76E-05	*HSPA1L/TP53/BAD/TP63/TP73/HIF1A/BAX/BCL2/MMP9/KDR*	10
GO:0031056	regulation of histone modification	11/293	143/18670	1,63E-05	8,75E-05	3,86E-05	*TP53/GATA2/IL1B/UBE2N/BRCA1/CTCFL/VEGFA/TGFB1/IGF2/FMR1/FOXP3*	11
GO:0005996	monosaccharide metabolic process	16/293	292/18670	1,63E-05	8,75E-05	3,86E-05	*TP53/SRC/AKT1/MTOR/BAD/IGFBP3/TNF/RBP4/LEPR/LCMT1/IGFBP4/INSR/INS/IGF2/IGF1/LEP*	16
GO:0009410	response to xenobiotic stimulus	16/293	292/18670	1,63E-05	8,75E-05	3,86E-05	*AIF1/STAT5B/NR3C1/EGFR/MDM2/MTOR/PPARG/GSTP1/ADA/AHR/ARNT/ARNT2/HNF4A/CASP9/F7/TGFB1*	16
GO:0002532	production of molecular mediator involved in inflammatory response	8/293	72/18670	1,68E-05	8,98E-05	3,96E-05	*TLR4/F2/SERPINE1/CHIA/INS/IL4R/LEP/NOS2*	8
GO:0051881	regulation of mitochondrial membrane potential	8/293	72/18670	1,68E-05	8,98E-05	3,96E-05	*SRC/AKT1/BAD/BAX/BCL2/BCL2L1/IFI6/KDR*	8
GO:0060193	positive regulation of lipase activity	8/293	72/18670	1,68E-05	8,98E-05	3,96E-05	*EGFR/FGFR1/ESR1/AGTR1/AGT/C5AR1/FGFR3/FLT1*	8
GO:0070167	regulation of biomineral tissue development	9/293	94/18670	1,69E-05	9,05E-05	3,99E-05	*NOS3/GATA1/ACVR1/BMP2/ADRB2/HIF1A/LTF/CEBPB/TGFB1*	9
GO:0009743	response to carbohydrate	14/293	230/18670	1,73E-05	9,25E-05	4,08E-05	*CASP3/BAD/IGF1R/GSTP1/ADRA2A/IL1B/HIF1A/HNF4A/CPB2/APOB/ITGA2/CFTR/TGFB1/LEP*	14
GO:0040013	negative regulation of locomotion	19/293	396/18670	1,74E-05	9,27E-05	4,09E-05	*AIF1/AKT1/STAT3/NR2F2/IGFBP3/PPARG/GSTP1/ADA/HRG/PRKG1/BCL2/APOE/TIMP1/SERPINE1/HMGB1/TGFB1/FBLN1/SULF1/IL4*	19
GO:0034504	protein localization to nucleus	15/293	262/18670	1,79E-05	9,57E-05	4,22E-05	*TP53/SRC/AKT1/STAT3/MDM2/RAN/AGT/F2/FLNA/MMP12/TGFB1/COL1A1/INS/IFNG/LEP*	15
GO:0032964	collagen biosynthetic process	7/293	53/18670	1,81E-05	9,65E-05	4,25E-05	*PPARG/IL6/F2/ITGA2/TGFB1/COL1A1/COL5A1*	7
GO:0070228	regulation of lymphocyte apoptotic process	7/293	53/18670	1,81E-05	9,65E-05	4,25E-05	*TP53/LGALS3/ADA/HIF1A/BAX/TSC22D3/IL10*	7
GO:0051193	regulation of cofactor metabolic process	10/293	119/18670	1,83E-05	9,75E-05	4,29E-05	*TP53/STAT3/ARNT/HIF1A/MMP3/INSR/INS/IFNG/HP/IGF1*	10
GO:1903076	regulation of protein localization to plasma membrane	9/293	95/18670	1,84E-05	9,79E-05	4,31E-05	*AKT1/EGFR/AR/LGALS3/TNF/BCL2L1/TGFB1/INS/IFNG*	9

ID	Description	GeneRatio	BgRatio	pvalue	p.adjust	qvalue	geneID	Count
GO:0006801	superoxide metabolic process	8/293	73/18670	1,86E-05	9,84E-05	4,34E-05	*EGFR/NOS3/GSTP1/TNF/AGT/TGFB1/DHFR/NOS2*	8
GO:0045685	regulation of glial cell differentiation	8/293	73/18670	1,86E-05	9,84E-05	4,34E-05	*MTOR/PPARG/BMP2/IL6/TP73/F2/TGFB1/DICER1*	8
GO:0090322	regulation of superoxide metabolic process	6/293	36/18670	1,86E-05	9,84E-05	4,34E-05	*EGFR/GSTP1/TNF/AGT/TGFB1/DHFR*	6
GO:0010469	regulation of signaling receptor activity	12/293	173/18670	1,92E-05	0,000101	4,46E-05	*SRC/ESR2/ADRA2A/ADRB2/SERPINE1/HMGB1/CGA/FSHB/EGF/IFNG/HFE/IL10*	12
GO:0010869	regulation of receptor biosynthetic process	5/293	22/18670	1,95E-05	0,000103	4,52E-05	*PPARG/JAK2/HIF1A/ITGB3/IFNG*	5
GO:0042359	vitamin D metabolic process	5/293	22/18670	1,95E-05	0,000103	4,52E-05	*NFKB1/FGFR1/IL1B/TNF/IFNG*	5
GO:0046628	positive regulation of insulin receptor signaling pathway	5/293	22/18670	1,95E-05	0,000103	4,52E-05	*SRC/AGT/INS/IGF2/LEP*	5
GO:0006140	regulation of nucleotide metabolic process	11/293	146/18670	1,98E-05	0,000104	4,58E-05	*TP53/STAT3/NOS3/ARNT/HIF1A/INSR/INS/IFNG/IGF1/IL4/NOS2*	11
GO:2001169	regulation of ATP biosynthetic process	9/293	96/18670	2,01E-05	0,000105	4,65E-05	*TP53/STAT3/ARNT/HIF1A/INSR/INS/IFNG/IGF1/IL4*	9
GO:0002429	immune response-activating cell surface receptor signaling pathway	21/293	473/18670	2,01E-05	0,000105	4,65E-05	*NFKB1/SRC/CHUK/IKBKG/LGALS3/ADA/UBE2N/BAX/BCL2/BAG6/C5AR1/CEACAM1/CTLA4/FOXP3/HLA-DQA1/HLA-DRB5/HLA-DRA/HLA-DQB2/HLA-DRB1/HLA-DQB1/HLA-DQA2*	21
GO:0006869	lipid transport	18/293	365/18670	2,01E-05	0,000106	4,65E-05	*ABCB1/ACE/NFKB1/AKT1/PPARG/IL1B/AGTR1/AGT/REN/RBP4/APOB/APOE/ITGB3/CEACAM1/CFTR/EGF/LEP/NOS2*	18
GO:0002720	positive regulation of cytokine production involved in immune response	7/293	54/18670	2,06E-05	0,000108	4,74E-05	*TLR4/IL1B/IL6/IL1R1/HLA-E/HLA-G/LILRB1*	7
GO:0097345	mitochondrial outer membrane permeabilization	7/293	54/18670	2,06E-05	0,000108	4,74E-05	*TP53/BAD/HSPA1A/TP63/TP73/BAX/BCL2*	7
GO:0071453	cellular response to oxygen levels	14/293	234/18670	2,10E-05	0,00011	4,83E-05	*TP53/SRC/AKT1/TRPC6/VHL/MDM2/MTOR/BAD/PPARG/ARNT/HIF1A/BCL2/BRIP1/VEGFA*	14
GO:0010212	response to ionizing radiation	11/293	147/18670	2,11E-05	0,00011	4,85E-05	*TP53/MDM2/CASP3/BAX/BCL2/BCL2L1/BRCA1/BRCA2/TGFB1/THBD/HAMP*	11
GO:0010811	positive regulation of cell-substrate adhesion	10/293	121/18670	2,12E-05	0,000111	4,87E-05	*JAK2/FN1/FGG/HRG/FLNA/FGB/FGA/CEACAM6/VEGFA/KDR*	10
GO:0046718	viral entry into host cell	10/293	121/18670	2,12E-05	0,000111	4,87E-05	*ACE2/EGFR/HSPA1B/NCAM1/HSPA1A/CCR5/ITGB6/ITGB3/ITGA2/TFRC*	10

ID	Description	GeneRatio	BgRatio	pvalue	p.adjust	qvalue	geneID	Count
GO:0002714	positive regulation of B cell mediated immunity	6/293	37/18670	2,19E-05	0,000114	5,01E-05	*TNF/HLA-E/TGFB1/TFRC/IL4/LTA*	6
GO:0002891	positive regulation of immunoglobulin mediated immune response	6/293	37/18670	2,19E-05	0,000114	5,01E-05	*TNF/HLA-E/TGFB1/TFRC/IL4/LTA*	6
GO:0003156	regulation of animal organ formation	6/293	37/18670	2,19E-05	0,000114	5,01E-05	*PIM1/AR/FGFR1/BMP2/SULF1/FGF1*	6
GO:0030212	hyaluronan metabolic process	6/293	37/18670	2,19E-05	0,000114	5,01E-05	*PIM1/NFKB1/AKT1/IL1B/TGFB1/EGF*	6
GO:0002479	antigen processing and presentation of exogenous peptide antigen via MHC class I, TAP-dependent	8/293	75/18670	2,27E-05	0,000118	5,18E-05	*CHUK/IKBKG/HLA-E/HLA-C/HLA-G/HLA-A/HLA-B/TAP2*	8
GO:0050796	regulation of insulin secretion	12/293	176/18670	2,27E-05	0,000118	5,19E-05	*BAD/JAK2/ADRA2A/IL1B/TNF/RBP4/HIF1A/HNF4A/CFTR/IFNG/LEP/NOS2*	12
GO:0043500	muscle adaptation	10/293	122/18670	2,28E-05	0,000118	5,19E-05	*ACTA1/AIF1/NOS3/MTOR/TNFRSF1A/IL1B/AGT/HAMP/IGF1/PDE5A*	10
GO:2001243	negative regulation of intrinsic apoptotic signaling pathway	9/293	98/18670	2,37E-05	0,000122	5,39E-05	*SRC/AKT1/MDM2/HSPA1A/HIF1A/BCL2/BCL2L1/MMP9/INS*	9
GO:0048592	eye morphogenesis	11/293	149/18670	2,39E-05	0,000123	5,44E-05	*STAT3/THRB/RBP4/HIF1A/BAX/BCL2/VEGFA/FBN1/COL5A1/COL5A2/FASLG*	11
GO:0002062	chondrocyte differentiation	10/293	123/18670	2,45E-05	0,000126	5,56E-05	*FGFR1/TGFBR1/BMP2/MBL2/TGFB1/TGFBI/COL6A3/COL6A1/SULF1/FGFR3*	10
GO:0050995	negative regulation of lipid catabolic process	5/293	23/18670	2,45E-05	0,000126	5,56E-05	*AKT1/ADRA2A/IL1B/TNF/INS*	5
GO:0070977	bone maturation	5/293	23/18670	2,45E-05	0,000126	5,56E-05	*BMP2/LTF/FGFR3/IGF1/LEP*	5
GO:2000637	positive regulation of gene silencing by miRNA	5/293	23/18670	2,45E-05	0,000126	5,56E-05	*TP53/STAT3/EGFR/TGFB1/FMR1*	5
GO:0071456	cellular response to hypoxia	13/293	207/18670	2,49E-05	0,000128	5,63E-05	*TP53/SRC/AKT1/TRPC6/VHL/MDM2/MTOR/BAD/ARNT/HIF1A/BCL2/BRIP1/VEGFA*	13
GO:0001937	negative regulation of endothelial cell proliferation	8/293	76/18670	2,50E-05	0,000128	5,64E-05	*NR2F2/PPARG/TGFBR1/TNF/APOE/PRL/SULF1/FLT1*	8
GO:0007492	endoderm development	8/293	76/18670	2,50E-05	0,000128	5,64E-05	*FN1/MMP2/MMP9/TGFB1/COL5A1/COL5A2/COL6A1/MMP15*	8
GO:0098586	cellular response to virus	7/293	56/18670	2,62E-05	0,000134	5,89E-05	*CHUK/BAD/BCL2L1/MMP12/IFI6/FMR1/IL12A*	7

ID	Description	GeneRatio	BgRatio	pvalue	p.adjust	qvalue	geneID	Count
GO:0009124	nucleoside monophosphate biosynthetic process	13/293	208/18670	2,62E-05	0,000134	5,89E-05	*TP53/STAT3/ADA/ARNT/HIF1A/TYMS/TGFB1/INSR/INS/IFNG/SLC4A1/IGF1/IL4*	13
GO:1901215	negative regulation of neuron death	13/293	208/18670	2,62E-05	0,000134	5,89E-05	*AKT1/STAT3/IKBKG/JAK2/HIF1A/BAX/BCL2/BCL2L1/APOE/C5AR1/CEBPB/CREB1/IL10*	13
GO:0051101	regulation of DNA binding	10/293	124/18670	2,63E-05	0,000134	5,90E-05	*GATA1/PPARG/JAK2/MMP9/HMGB1/ITGA2/TGFB1/EGF/IFNG/IGF1*	10
GO:0002551	mast cell chemotaxis	4/293	12/18670	2,66E-05	0,000134	5,93E-05	*VEGFA/VEGFC/VEGFB/PGF*	4
GO:0033197	response to vitamin E	4/293	12/18670	2,66E-05	0,000134	5,93E-05	*PPARG/ADA/COL1A1/LEP*	4
GO:0035630	bone mineralization involved in bone maturation	4/293	12/18670	2,66E-05	0,000134	5,93E-05	*BMP2/LTF/IGF1/LEP*	4
GO:0042368	vitamin D biosynthetic process	4/293	12/18670	2,66E-05	0,000134	5,93E-05	*NFKB1/IL1B/TNF/IFNG*	4
GO:0051132	NK T cell activation	4/293	12/18670	2,66E-05	0,000134	5,93E-05	*IL12B/IL23R/IL12A/IL6R*	4
GO:0072540	T-helper 17 cell lineage commitment	4/293	12/18670	2,66E-05	0,000134	5,93E-05	*STAT3/IL6/IL12B/IL23R*	4
GO:0090331	negative regulation of platelet aggregation	4/293	12/18670	2,66E-05	0,000134	5,93E-05	*FGG/PRKG1/CD9/CEACAM1*	4
GO:0097531	mast cell migration	4/293	12/18670	2,66E-05	0,000134	5,93E-05	*VEGFA/VEGFC/VEGFB/PGF*	4
GO:2001198	regulation of dendritic cell differentiation	4/293	12/18670	2,66E-05	0,000134	5,93E-05	*HMGB1/HLA-G/LILRB1/CEBPB*	4
GO:2001235	positive regulation of apoptotic signaling pathway	12/293	179/18670	2,69E-05	0,000136	5,98E-05	*TP53/BAD/JAK2/TGFBR1/TP63/TP73/AGT/BAX/BCL2/BCL2L1/TIMP3/MMP9*	12
GO:0070498	interleukin-1-mediated signaling pathway	9/293	100/18670	2,79E-05	0,00014	6,19E-05	*NFKB1/CHUK/IKBKG/IL1B/IL6/IL1R1/UBE2N/IL1RN/IL1A*	9
GO:1905330	regulation of morphogenesis of an epithelium	12/293	180/18670	2,84E-05	0,000143	6,30E-05	*MTOR/AR/FGFR1/ESR1/TNF/AGT/VEGFA/TGFB1/SULF1/FGF1/FGF7/ROR1*	12
GO:0043523	regulation of neuron apoptotic process	13/293	210/18670	2,90E-05	0,000146	6,42E-05	*TP53/CASP3/JAK2/TNF/HIF1A/CASP9/BAX/BCL2/BCL2L1/APOE/C5AR1/CEBPB/FASLG*	13
GO:0043312	neutrophil degranulation	21/293	485/18670	2,90E-05	0,000146	6,42E-05	*NFKB1/PLD1/LGALS3/HSPA1B/HSPA1A/GSTP1/PA2G4/SERPINA1/MMP9/HMGB1/C5AR1/SLC11A1/CD14/LTF/CEACAM1/CEACAM6/LCN2/TIMP2/CXCR1/HP/IGF2R*	21
GO:0010661	positive regulation of muscle cell apoptotic process	6/293	39/18670	3,00E-05	0,00015	6,61E-05	*TP53/PPARG/AGT/IL12B/IFNG/IL12A*	6

ID	Description	GeneRatio	BgRatio	pvalue	p.adjust	qvalue	geneID	Count
GO:0019048	modulation by virus of host morphology or physiology	6/293	39/18670	3,00E-05	0,00015	6,61E-05	*BAD/BCL2L1/TYMS/TGFB1/EIF2AK2/INSR*	6
GO:0033146	regulation of intracellular estrogen receptor signaling pathway	6/293	39/18670	3,00E-05	0,00015	6,61E-05	*SRC/AR/ESR1/TP63/BRCA1/CNOT1*	6
GO:0045667	regulation of osteoblast differentiation	10/293	126/18670	3,02E-05	0,000151	6,64E-05	*ACVR1/BMP2/TNF/IL6/TP63/LTF/CEBPA/CEBPB/IGF1/IL6R*	10
GO:0046031	ADP metabolic process	10/293	126/18670	3,02E-05	0,000151	6,64E-05	*TP53/STAT3/BAD/ARNT/HIF1A/INSR/INS/IFNG/SLC4A1/IGF1*	10
GO:0031016	pancreas development	8/293	78/18670	3,03E-05	0,000151	6,64E-05	*AKT1/BAD/IL6/HNF4A/INSR/IGF2/HNF1A/IL6R*	8
GO:0055021	regulation of cardiac muscle tissue growth	8/293	78/18670	3,03E-05	0,000151	6,64E-05	*PIM1/MTOR/FGFR1/TGFBR1/TP73/RBP4/HAMP/IGF1*	8
GO:0072332	intrinsic apoptotic signaling pathway by p53 class mediator	8/293	78/18670	3,03E-05	0,000151	6,64E-05	*TP53/MDM2/TP63/TP73/BAX/BCL2/BAG6/BRCA2*	8
GO:0002861	regulation of inflammatory response to antigenic stimulus	5/293	24/18670	3,06E-05	0,000152	6,68E-05	*TNF/HLA-E/IL12B/LTA/IL10*	5
GO:0042832	defense response to protozoan	5/293	24/18670	3,06E-05	0,000152	6,68E-05	*SLC11A1/IL12B/IL12A/IL4R/IL10*	5
GO:0060396	growth hormone receptor signaling pathway	5/293	24/18670	3,06E-05	0,000152	6,68E-05	*STAT3/STAT5B/JAK2/PRLR/PRL*	5
GO:1900078	positive regulation of cellular response to insulin stimulus	5/293	24/18670	3,06E-05	0,000152	6,68E-05	*SRC/AGT/INS/IGF2/LEP*	5
GO:2001026	regulation of endothelial cell chemotaxis	5/293	24/18670	3,06E-05	0,000152	6,68E-05	*FGFR1/HRG/VEGFA/FGF1/KDR*	5
GO:0033044	regulation of chromosome organization	17/293	342/18670	3,10E-05	0,000153	6,75E-05	*TP53/SRC/GATA2/IL1B/UBE2N/LCMT1/APC/BUB1/BUB1B/BUB3/BRCA1/CTCFL/VEGFA/TGFB1/IGF2/FMR1/FOXP3*	17
GO:0002283	neutrophil activation involved in immune response	21/293	488/18670	3,18E-05	0,000157	6,92E-05	*NFKB1/PLD1/LGALS3/HSPA1B/HSPA1A/GSTP1/PA2G4/SERPINA1/MMP9/HMGB1/C5AR1/SLC11A1/CD14/LTF/CEACAM1/CEACAM6/LCN2/TIMP2/CXCR1/HP/IGF2R*	21
GO:0001823	mesonephros development	9/293	102/18670	3,27E-05	0,000161	7,10E-05	*FGFR1/BMP2/AGT/REN/BCL2/VEGFA/TGFB1/FGF1/PGF*	9
GO:0030520	intracellular estrogen receptor signaling pathway	7/293	58/18670	3,31E-05	0,000163	7,18E-05	*SRC/ESR2/AR/ESR1/TP63/BRCA1/CNOT1*	7

ID	Description	GeneRatio	BgRatio	pvalue	p.adjust	qvalue	geneID	Count
GO:0035306	positive regulation of dephosphorylation	7/293	58/18670	3,31E-05	0,000163	7,18E-05	*SRC/MTOR/JAK2/BMP2/ITGA2/TGFB1/IFNG*	7
GO:0006110	regulation of glycolytic process	8/293	79/18670	3,32E-05	0,000163	7,20E-05	*TP53/STAT3/ARNT/HIF1A/INSR/INS/IFNG/IGF1*	8
GO:0042590	antigen processing and presentation of exogenous peptide antigen via MHC class I	8/293	79/18670	3,32E-05	0,000163	7,20E-05	*CHUK/IKBKG/HLA-E/HLA-C/HLA-G/HLA-A/HLA-B/TAP2*	8
GO:0009127	purine nucleoside monophosphate biosynthetic process	12/293	183/18670	3,35E-05	0,000164	7,23E-05	*TP53/STAT3/ADA/ARNT/HIF1A/TGFB1/INSR/INS/IFNG/SLC4A1/IGF1/IL4*	12
GO:0009168	purine ribonucleoside monophosphate biosynthetic process	12/293	183/18670	3,35E-05	0,000164	7,23E-05	*TP53/STAT3/ADA/ARNT/HIF1A/TGFB1/INSR/INS/IFNG/SLC4A1/IGF1/IL4*	12
GO:0038061	NIK/NF-kappaB signaling	12/293	183/18670	3,35E-05	0,000164	7,23E-05	*AKT1/TLR4/EGFR/CHUK/IL1B/TNF/HAVCR2/HMGB1/CD14/IL12B/DICER1/EIF2AK2*	12
GO:0034763	negative regulation of transmembrane transport	10/293	128/18670	3,45E-05	0,000169	7,45E-05	*AKT1/MTOR/ADRA2A/IL1B/TNF/MMP9/TGFB1/FMR1/HAMP/LEP*	10
GO:0001990	regulation of systemic arterial blood pressure by hormone	6/293	40/18670	3,48E-05	0,00017	7,49E-05	*ACE/ACE2/NOS3/AGTR1/AGT/REN*	6
GO:0045124	regulation of bone resorption	6/293	40/18670	3,48E-05	0,00017	7,49E-05	*SRC/EGFR/IL6/ITGB3/FSHB/TFRC*	6
GO:0007229	integrin-mediated signaling pathway	9/293	103/18670	3,53E-05	0,000172	7,58E-05	*SRC/FLNA/ITGB4/ITGB6/ITGA2B/ITGB3/TIMP1/ITGA2/CEACAM1*	9
GO:0032652	regulation of interleukin-1 production	9/293	103/18670	3,53E-05	0,000172	7,58E-05	*TLR4/JAK2/GSTP1/HAVCR2/HMGB1/CEACAM1/IFNG/IGF1/IL10*	9
GO:0032388	positive regulation of intracellular transport	13/293	215/18670	3,70E-05	0,00018	7,94E-05	*HSPA1L/TP53/MDM2/GATA2/RAN/IL1B/FLNA/SPAG5/MAP2/TGFB1/IFNG/IL4R/LEP*	13
GO:0001658	branching involved in ureteric bud morphogenesis	7/293	59/18670	3,70E-05	0,00018	7,94E-05	*BMP2/AGT/BCL2/VEGFA/TGFB1/FGF1/PGF*	7
GO:0001562	response to protozoan	5/293	25/18670	3,78E-05	0,000183	8,05E-05	*SLC11A1/IL12B/IL12A/IL4R/IL10*	5
GO:0045672	positive regulation of osteoclast differentiation	5/293	25/18670	3,78E-05	0,000183	8,05E-05	*TNF/CREB1/IL12B/IL23R/IFNG*	5
GO:0060444	branching involved in mammary gland duct morphogenesis	5/293	25/18670	3,78E-05	0,000183	8,05E-05	*SRC/AR/ESR1/TGFB1/PGR*	5

ID	Description	GeneRatio	BgRatio	pvalue	p.adjust	qvalue	geneID	Count
GO:0060571	morphogenesis of an epithelial fold	5/293	25/18670	3,78E-05	0,000183	8,05E-05	*EGFR/AR/TP63/HIF1A/SULF1*	5
GO:0071378	cellular response to growth hormone stimulus	5/293	25/18670	3,78E-05	0,000183	8,05E-05	*STAT3/STAT5B/JAK2/PRLR/PRL*	5
GO:1904385	cellular response to angiotensin	5/293	25/18670	3,78E-05	0,000183	8,05E-05	*NFKB1/SRC/AGTR1/AGT/BRIP1*	5
GO:0034374	low-density lipoprotein particle remodeling	4/293	13/18670	3,80E-05	0,000183	8,07E-05	*AGTR1/AGT/APOB/APOE*	4
GO:0043568	positive regulation of insulin-like growth factor receptor signaling pathway	4/293	13/18670	3,80E-05	0,000183	8,07E-05	*AR/IGFBP3/IGFBP4/IGF1*	4
GO:0090594	inflammatory response to wounding	4/293	13/18670	3,80E-05	0,000183	8,07E-05	*HIF1A/TIMP1/TGFB1/IL1A*	4
GO:0046822	regulation of nucleocytoplasmic transport	9/293	104/18670	3,81E-05	0,000184	8,09E-05	*TP53/MDM2/RAN/IL1B/FLNA/XPO5/TGFB1/IFNG/LEP*	9
GO:0043271	negative regulation of ion transport	11/293	157/18670	3,88E-05	0,000187	8,23E-05	*AKT1/NOS3/MTOR/ADRA2A/BCL2/MMP9/LILRB1/TGFB1/FMR1/HAMP/LEP*	11
GO:0009416	response to light stimulus	16/293	314/18670	3,94E-05	0,000189	8,34E-05	*TP53/AKT1/EGFR/MDM2/CASP3/MTOR/HIF1A/CASP9/BAX/BCL2/BRCA2/CREB1/CRTC1/IL12B/FMR1/IL12A*	16
GO:0030811	regulation of nucleotide catabolic process	8/293	81/18670	3,99E-05	0,000191	8,44E-05	*TP53/STAT3/ARNT/HIF1A/INSR/INS/IFNG/IGF1*	8
GO:0007178	transmembrane receptor protein serine/threonine kinase signaling pathway	17/293	349/18670	3,99E-05	0,000192	8,44E-05	*TMPRSS6/TP53/SRC/HSPA1A/ACVR1/TGFBR1/BMP2/HNF4A/INHA/CREB1/FSHB/TGFB1/FBN1/COL1A2/FST/SULF1/HFE*	17
GO:0030890	positive regulation of B cell proliferation	6/293	41/18670	4,02E-05	0,000192	8,48E-05	*TLR4/CD320/ADA/BCL2/TFRC/IL4*	6
GO:0050873	brown fat cell differentiation	6/293	41/18670	4,02E-05	0,000192	8,48E-05	*MTOR/ADRB2/CEBPA/CEBPB/INS/LEP*	6
GO:0042692	muscle cell differentiation	18/293	385/18670	4,06E-05	0,000194	8,56E-05	*ACTA1/AKT1/CASP3/CHUK/MTOR/ACVR1/BMP2/AGT/BCL2/CD9/CEACAM5/VEGFA/TGFB1/NEB/IGF2/HAMP/IGF1/IL4R*	18
GO:0036294	cellular response to decreased oxygen levels	13/293	217/18670	4,08E-05	0,000195	8,58E-05	*TP53/SRC/AKT1/TRPC6/VHL/MDM2/MTOR/BAD/ARNT/HIF1A/BCL2/BRIP1/VEGFA*	13
GO:0055017	cardiac muscle tissue growth	9/293	105/18670	4,11E-05	0,000196	8,65E-05	*PIM1/MTOR/FGFR1/TGFBR1/TP73/AGT/RBP4/HAMP/IGF1*	9

ID	Description	GeneRatio	BgRatio	pvalue	p.adjust	qvalue	geneID	Count
GO:1902110	positive regulation of mitochondrial membrane permeability involved in apoptotic process	7/293	60/18670	4,14E-05	0,000197	8,69E-05	*TP53/BAD/HSPA1A/TP63/TP73/BAX/BCL2*	7
GO:0009142	nucleoside triphosphate biosynthetic process	12/293	188/18670	4,36E-05	0,000207	9,14E-05	*TP53/STAT3/ARNT/HIF1A/TYMS/TGFB1/INSR/INS/IFNG/SLC4A1/IGF1/IL4*	12
GO:0002673	regulation of acute inflammatory response	11/293	159/18670	4,36E-05	0,000207	9,14E-05	*PPARG/GSTP1/IL1B/TNF/IL6/F2/CPB2/F12/C5AR1/HLA-E/INS*	11
GO:0048762	mesenchymal cell differentiation	13/293	219/18670	4,48E-05	0,000213	9,39E-05	*MTOR/FGFR1/FN1/ACVR1/TGFBR1/BMP2/IL1B/IL6/HIF1A/BCL2/TGFB1/COL1A1/EFNB1*	13
GO:0003158	endothelium development	10/293	132/18670	4,50E-05	0,000214	9,41E-05	*NR2F2/TNFRSF1A/ACVR1/IL1B/TNF/PROC/CEACAM1/VEGFA/FGF1/KDR*	10
GO:0003170	heart valve development	7/293	61/18670	4,61E-05	0,000217	9,58E-05	*MDM2/NOS3/MTOR/TNFRSF1A/ACVR1/BMP2/TGFB1*	7
GO:0003081	regulation of systemic arterial blood pressure by renin-angiotensin	5/293	26/18670	4,62E-05	0,000217	9,58E-05	*ACE/ACE2/AGTR1/AGT/REN*	5
GO:0006309	apoptotic DNA fragmentation	5/293	26/18670	4,62E-05	0,000217	9,58E-05	*CASP3/IL6/BAX/HMGB1/DICER1*	5
GO:0031954	positive regulation of protein autophosphorylation	5/293	26/18670	4,62E-05	0,000217	9,58E-05	*ACE/SRC/VEGFA/INS/VEGFC*	5
GO:0072539	T-helper 17 cell differentiation	5/293	26/18670	4,62E-05	0,000217	9,58E-05	*STAT3/IL6/IL12B/IL23R/FOXP3*	5
GO:1900739	regulation of protein insertion into mitochondrial membrane involved in apoptotic signaling pathway	5/293	26/18670	4,62E-05	0,000217	9,58E-05	*TP53/BAD/TP63/TP73/BCL2*	5
GO:1900740	positive regulation of protein insertion into mitochondrial membrane involved in apoptotic signaling pathway	5/293	26/18670	4,62E-05	0,000217	9,58E-05	*TP53/BAD/TP63/TP73/BCL2*	5
GO:1903959	regulation of anion transmembrane transport	5/293	26/18670	4,62E-05	0,000217	9,58E-05	*ABCB1/AKT1/MTOR/AGT/CFTR*	5

ID	Description	GeneRatio	BgRatio	pvalue	p.adjust	qvalue	geneID	Count
GO:0006754	ATP biosynthetic process	11/293	160/18670	4,62E-05	0,000217	9,58E-05	*TP53/STAT3/ARNT/HIF1A/TGFB1/INSR/INS/IFNG/SLC4A1/IGF1/IL4*	11
GO:0010470	regulation of gastrulation	6/293	42/18670	4,63E-05	0,000217	9,58E-05	*FGFR1/HNF4A/COL5A1/COL5A2/IL1RN/IL10*	6
GO:0010907	positive regulation of glucose metabolic process	6/293	42/18670	4,63E-05	0,000217	9,58E-05	*SRC/AKT1/INSR/INS/IGF2/IGF1*	6
GO:0007204	positive regulation of cytosolic calcium ion concentration	16/293	319/18670	4,76E-05	0,000223	9,84E-05	*TRPC6/GATA2/ESR1/JAK2/AGTR1/CCR5/AGT/F2/BAX/BCL2/HMGB1/C5AR1/TGFB1/CXCR1/FASLG/TRPC1*	16
GO:0001505	regulation of neurotransmitter levels	17/293	354/18670	4,77E-05	0,000224	9,85E-05	*AIF1/AKT1/TLR4/NOS3/MTOR/JAK2/IL1B/TNF/AGT/PAH/HTR1A/ITGB3/INSR/FMR1/IFNG/NOS2/IL10*	17
GO:0014706	striated muscle tissue development	18/293	390/18670	4,80E-05	0,000225	9,90E-05	*PIM1/ACTA1/MTOR/NR2F2/FGFR1/ACVR1/TGFBR1/BMP2/TP73/AGT/RBP4/BCL2/VEGFA/CREB1/TGFB1/NEB/HAMP/IGF1*	18
GO:0044262	cellular carbohydrate metabolic process	15/293	286/18670	4,95E-05	0,000231	0,000102	*TP53/SRC/AKT1/STAT3/MTOR/BAD/IGFBP3/LEPR/LCMT1/IGFBP4/INSR/INS/IGF2/IGF1/LEP*	15
GO:0048565	digestive tract development	10/293	134/18670	5,11E-05	0,000239	0,000105	*EGFR/ADA/TNF/TP63/HIF1A/BCL2/TYMS/ITGB4/TGFB1/CXCL8*	10
GO:0010676	positive regulation of cellular carbohydrate metabolic process	7/293	62/18670	5,13E-05	0,000239	0,000105	*SRC/AKT1/BAD/INSR/INS/IGF2/IGF1*	7
GO:0045453	bone resorption	7/293	62/18670	5,13E-05	0,000239	0,000105	*SRC/EGFR/ADRB2/IL6/ITGB3/FSHB/TFRC*	7
GO:0055025	positive regulation of cardiac muscle tissue development	7/293	62/18670	5,13E-05	0,000239	0,000105	*PIM1/MTOR/FGFR1/CREB1/TGFB1/HAMP/IGF1*	7
GO:0070059	intrinsic apoptotic signaling pathway in response to endoplasmic reticulum stress	7/293	62/18670	5,13E-05	0,000239	0,000105	*TP53/HSPA1A/BAX/BCL2/BCL2L1/BAG6/CEBPB*	7
GO:1902686	mitochondrial outer membrane permeabilization involved in programmed cell death	7/293	62/18670	5,13E-05	0,000239	0,000105	*TP53/BAD/HSPA1A/TP63/TP73/BAX/BCL2*	7
GO:0046916	cellular transition metal ion homeostasis	9/293	108/18670	5,14E-05	0,000239	0,000105	*TMPRSS6/HEPH/HIF1A/SLC11A1/LTF/LCN2/HAMP/HFE/TFRC*	9
GO:0051924	regulation of calcium ion transport	14/293	254/18670	5,16E-05	0,00024	0,000106	*TRPC6/NOS3/LGALS3/ADRA2A/AGT/F2/IL16/BAX/BCL2/LILRB1/TGFB1/EGF/FMR1/TRPC1*	14
GO:0060420	regulation of heart growth	8/293	84/18670	5,19E-05	0,000241	0,000106	*PIM1/MTOR/FGFR1/TGFBR1/TP73/RBP4/HAMP/IGF1*	8

ID	Description	GeneRatio	BgRatio	pvalue	p.adjust	qvalue	geneID	Count
GO:0010935	regulation of macrophage cytokine production	4/293	14/18670	5,25E-05	0,000243	0,000107	*TLR4/HLA-G/LILRB1/TGFB1*	4
GO:0030213	hyaluronan biosynthetic process	4/293	14/18670	5,25E-05	0,000243	0,000107	*NFKB1/IL1B/TGFB1/EGF*	4
GO:0045591	positive regulation of regulatory T cell differentiation	4/293	14/18670	5,25E-05	0,000243	0,000107	*HLA-G/TGFB1/FOXP3/IFNG*	4
GO:0010863	positive regulation of phospholipase C activity	6/293	43/18670	5,31E-05	0,000245	0,000108	*EGFR/FGFR1/ESR1/AGT/C5AR1/FLT1*	6
GO:0045687	positive regulation of glial cell differentiation	6/293	43/18670	5,31E-05	0,000245	0,000108	*MTOR/PPARG/BMP2/TP73/TGFB1/DICER1*	6
GO:0060562	epithelial tube morphogenesis	16/293	322/18670	5,32E-05	0,000245	0,000108	*SRC/CASP3/AR/ESR1/ACVR1/BMP2/TNF/AGT/HIF1A/BCL2/VEGFA/TGFB1/EGF/PGR/FGF1/PGF*	16
GO:0042326	negative regulation of phosphorylation	20/293	468/18670	5,40E-05	0,000249	0,00011	*AKT1/STAT3/TLR4/CASP3/MTOR/NR2F2/IGF1R/IGFBP3/GSTP1/IL1B/BAX/INHA/APC/APOE/TIMP3/CEACAM1/CEBPA/TGFB1/FBLN1/IFNG*	20
GO:0043032	positive regulation of macrophage activation	5/293	27/18670	5,59E-05	0,000257	0,000113	*TLR4/HAVCR2/CEBPA/IL4R/IL10*	5
GO:0045671	negative regulation of osteoclast differentiation	5/293	27/18670	5,59E-05	0,000257	0,000113	*TLR4/LTF/LILRB1/FBN1/IL4*	5
GO:0048799	animal organ maturation	5/293	27/18670	5,59E-05	0,000257	0,000113	*BMP2/LTF/FGFR3/IGF1/LEP*	5
GO:0046637	regulation of alpha-beta T cell differentiation	7/293	63/18670	5,69E-05	0,000261	0,000115	*ADA/HMGB1/IL12B/IL23R/FOXP3/IFNG/IL4R*	7
GO:0003015	heart process	15/293	290/18670	5,79E-05	0,000265	0,000117	*ACE/ACE2/SRC/THRB/MDM2/NOS3/MTOR/JAK2/ADA/AGT/FLNA/DES/VEGFB/TRPC1/PDE5A*	15
GO:0006936	muscle contraction	17/293	360/18670	5,87E-05	0,000269	0,000118	*ACE2/ACTA1/CHUK/MTOR/ACTA2/ADA/ADRA2A/ADRB2/AGT/FLNA/PRKG1/ITGA2/SULF1/DES/NEB/VEGFB/PDE5A*	17
GO:0030225	macrophage differentiation	6/293	44/18670	6,07E-05	0,000277	0,000122	*GATA2/INHA/MMP9/CEBPA/VEGFA/TGFB1*	6
GO:0034105	positive regulation of tissue remodeling	6/293	44/18670	6,07E-05	0,000277	0,000122	*EGFR/HRG/BAX/FSHB/IL12B/TFRC*	6
GO:0045601	regulation of endothelial cell differentiation	6/293	44/18670	6,07E-05	0,000277	0,000122	*TNFRSF1A/IL1B/TNF/PROC/CEACAM1/VEGFA*	6
GO:0006919	activation of cysteine-type endopeptidase	8/293	86/18670	6,15E-05	0,00028	0,000124	*BAD/PPARG/JAK2/TNF/CASP9/BAX/FASLG/F3*	8

ID	Description	GeneRatio	BgRatio	pvalue	p.adjust	qvalue	geneID	Count
	activity involved in apoptotic process							
GO:0009141	nucleoside triphosphate metabolic process	17/293	362/18670	6,29E-05	0,000287	0,000126	*TP53/STAT3/BAD/HSPA1B/HSPA1A/ADA/RAN/ARNT/HIF1A/TYMS/TGFB1/INSR/INS/IFNG/SLC4A1/IGF1/IL4*	17
GO:0035794	positive regulation of mitochondrial membrane permeability	7/293	64/18670	6,31E-05	0,000287	0,000126	*TP53/BAD/HSPA1A/TP63/TP73/BAX/BCL2*	7
GO:0045600	positive regulation of fat cell differentiation	7/293	64/18670	6,31E-05	0,000287	0,000126	*AKT1/PPARG/BMP2/CEBPA/CEBPB/CREB1/INS*	7
GO:0009135	purine nucleoside diphosphate metabolic process	10/293	138/18670	6,56E-05	0,000298	0,000131	*TP53/STAT3/BAD/ARNT/HIF1A/INSR/INS/IFNG/SLC4A1/IGF1*	10
GO:0009179	purine ribonucleoside diphosphate metabolic process	10/293	138/18670	6,56E-05	0,000298	0,000131	*TP53/STAT3/BAD/ARNT/HIF1A/INSR/INS/IFNG/SLC4A1/IGF1*	10
GO:0006112	energy reserve metabolic process	8/293	87/18670	6,68E-05	0,000302	0,000133	*AKT1/MTOR/LEPR/INSR/INS/IGF2/IGF1/LEP*	8
GO:0045844	positive regulation of striated muscle tissue development	8/293	87/18670	6,68E-05	0,000302	0,000133	*PIM1/MTOR/FGFR1/BCL2/CREB1/TGFB1/HAMP/IGF1*	8
GO:0048636	positive regulation of muscle organ development	8/293	87/18670	6,68E-05	0,000302	0,000133	*PIM1/MTOR/FGFR1/BCL2/CREB1/TGFB1/HAMP/IGF1*	8
GO:0033688	regulation of osteoblast proliferation	5/293	28/18670	6,72E-05	0,000303	0,000133	*GATA1/BMP2/BCL2/LTF/EIF2AK2*	5
GO:0045408	regulation of interleukin-6 biosynthetic process	5/293	28/18670	6,72E-05	0,000303	0,000133	*STAT3/IL1B/TNF/CEBPB/TLR1*	5
GO:0060260	regulation of transcription initiation from RNA polymerase II promoter	5/293	28/18670	6,72E-05	0,000303	0,000133	*TP53/ESR1/HMGB1/CREB1/HNF1A*	5
GO:1902003	regulation of amyloid-beta formation	5/293	28/18670	6,72E-05	0,000303	0,000133	*CASP3/TNF/APOE/IFNG/IGF1*	5
GO:1990776	response to angiotensin	5/293	28/18670	6,72E-05	0,000303	0,000133	*NFKB1/SRC/AGTR1/AGT/BRIP1*	5

ID	Description	GeneRatio	BgRatio	pvalue	p.adjust	qvalue	geneID	Count
GO:0051054	positive regulation of DNA metabolic process	13/293	228/18670	6,77E-05	0,000305	0,000134	*SRC/AKT1/EGFR/FGFR1/IL6/UBE2N/BAX/DNA2/BRCA1/HMGB1/TGFB1/TFRC/IL4*	13
GO:0000041	transition metal ion transport	9/293	112/18670	6,83E-05	0,000307	0,000135	*TRPC6/TCN2/HEPH/SLC11A1/IFNG/HAMP/HFE/TFRC/TRPC1*	9
GO:0002065	columnar/cuboidal epithelial cell differentiation	9/293	112/18670	6,83E-05	0,000307	0,000135	*FGFR1/BAD/BMP2/TP63/HIF1A/HNF4A/TYMS/SERPINE1/CEBPB*	9
GO:0060419	heart growth	9/293	112/18670	6,83E-05	0,000307	0,000135	*PIM1/MTOR/FGFR1/TGFBR1/TP73/AGT/RBP4/HAMP/IGF1*	9
GO:0009156	ribonucleoside monophosphate biosynthetic process	12/293	197/18670	6,85E-05	0,000307	0,000135	*TP53/STAT3/ADA/ARNT/HIF1A/TGFB1/INSR/INS/IFNG/SLC4A1/IGF1/IL4*	12
GO:0010952	positive regulation of peptidase activity	12/293	197/18670	6,85E-05	0,000307	0,000135	*STAT3/BAD/PPARG/JAK2/FN1/TNF/CASP9/BAX/HMGB1/FBLN1/FASLG/F3*	12
GO:0042771	intrinsic apoptotic signaling pathway in response to DNA damage by p53 class mediator	6/293	45/18670	6,91E-05	0,000309	0,000136	*TP53/TP63/TP73/BCL2/BAG6/BRCA2*	6
GO:1900274	regulation of phospholipase C activity	6/293	45/18670	6,91E-05	0,000309	0,000136	*EGFR/FGFR1/ESR1/AGT/C5AR1/FLT1*	6
GO:0002548	monocyte chemotaxis	7/293	65/18670	6,98E-05	0,000311	0,000137	*AIF1/LGALS3/IL6/SERPINE1/HMGB1/FLT1/IL6R*	7
GO:0014823	response to activity	7/293	65/18670	6,98E-05	0,000311	0,000137	*MTOR/AGT/HIF1A/ITGA2/CREB1/LEP/IL10*	7
GO:0060675	ureteric bud morphogenesis	7/293	65/18670	6,98E-05	0,000311	0,000137	*BMP2/AGT/BCL2/VEGFA/TGFB1/FGF1/PGF*	7
GO:0034111	negative regulation of homotypic cell-cell adhesion	4/293	15/18670	7,08E-05	0,000313	0,000138	*FGG/PRKG1/CD9/CEACAM1*	4
GO:0042362	fat-soluble vitamin biosynthetic process	4/293	15/18670	7,08E-05	0,000313	0,000138	*NFKB1/IL1B/TNF/IFNG*	4
GO:0045073	regulation of chemokine biosynthetic process	4/293	15/18670	7,08E-05	0,000313	0,000138	*IL1B/TNF/IL6/IFNG*	4
GO:0045410	positive regulation of interleukin-6 biosynthetic process	4/293	15/18670	7,08E-05	0,000313	0,000138	*STAT3/IL1B/TNF/TLR1*	4
GO:0045898	regulation of RNA polymerase II transcriptional preinitiation complex assembly	4/293	15/18670	7,08E-05	0,000313	0,000138	*TP53/ESR1/HMGB1/CREB1*	4

ID	Description	GeneRatio	BgRatio	pvalue	p.adjust	qvalue	geneID	Count
GO:0051770	positive regulation of nitric-oxide synthase biosynthetic process	4/293	15/18670	7,08E-05	0,000313	0,000138	*TLR4/JAK2/KDR/IFNG*	4
GO:0090197	positive regulation of chemokine secretion	4/293	15/18670	7,08E-05	0,000313	0,000138	*AIF1/TNF/CHIA/IL4R*	4
GO:0043470	regulation of carbohydrate catabolic process	8/293	88/18670	7,25E-05	0,000319	0,000141	*TP53/STAT3/ARNT/HIF1A/INSR/INS/IFNG/IGF1*	8
GO:0097194	execution phase of apoptosis	8/293	88/18670	7,25E-05	0,000319	0,000141	*TP53/AKT1/CASP3/IL6/BAX/BCL2L1/HMGB1/DICER1*	8
GO:1900407	regulation of cellular response to oxidative stress	8/293	88/18670	7,25E-05	0,000319	0,000141	*AKT1/TLR4/TNF/HIF1A/MMP3/DHFR/INS/IL10*	8
GO:1901863	positive regulation of muscle tissue development	8/293	88/18670	7,25E-05	0,000319	0,000141	*PIM1/MTOR/FGFR1/BCL2/CREB1/TGFB1/HAMP/IGF1*	8
GO:1901888	regulation of cell junction assembly	8/293	88/18670	7,25E-05	0,000319	0,000141	*ACE/ACE2/SRC/TNF/AGT/HRG/VEGFA/KDR*	8
GO:2000177	regulation of neural precursor cell proliferation	8/293	88/18670	7,25E-05	0,000319	0,000141	*TP53/GATA2/HIF1A/FLNA/VEGFA/TGFB1/EGF/VEGFC*	8
GO:0050890	cognition	15/293	296/18670	7,29E-05	0,000321	0,000141	*EGFR/CASP3/MTOR/TNF/AGT/HIF1A/SGK1/APOE/C5AR1/CEBPB/CREB1/CRTC1/INSR/INS/HLA-DRA*	15
GO:0046620	regulation of organ growth	9/293	113/18670	7,33E-05	0,000322	0,000142	*PIM1/AKT1/MTOR/FGFR1/TGFBR1/TP73/RBP4/HAMP/IGF1*	9
GO:0009185	ribonucleoside diphosphate metabolic process	10/293	140/18670	7,41E-05	0,000325	0,000143	*TP53/STAT3/BAD/ARNT/HIF1A/INSR/INS/IFNG/SLC4A1/IGF1*	10
GO:0072171	mesonephric tubule morphogenesis	7/293	66/18670	7,70E-05	0,000337	0,000149	*BMP2/AGT/BCL2/VEGFA/TGFB1/FGF1/PGF*	7
GO:1902108	regulation of mitochondrial membrane permeability involved in apoptotic process	7/293	66/18670	7,70E-05	0,000337	0,000149	*TP53/BAD/HSPA1A/TP63/TP73/BAX/BCL2*	7
GO:1905710	positive regulation of membrane permeability	7/293	66/18670	7,70E-05	0,000337	0,000149	*TP53/BAD/HSPA1A/TP63/TP73/BAX/BCL2*	7
GO:0005978	glycogen biosynthetic process	6/293	46/18670	7,84E-05	0,000342	0,000151	*AKT1/MTOR/INSR/INS/IGF2/IGF1*	6
GO:0009250	glucan biosynthetic process	6/293	46/18670	7,84E-05	0,000342	0,000151	*AKT1/MTOR/INSR/INS/IGF2/IGF1*	6
GO:0044003	modification by symbiont of host	6/293	46/18670	7,84E-05	0,000342	0,000151	*BAD/BCL2L1/TYMS/TGFB1/EIF2AK2/INSR*	6

ID	Description	GeneRatio	BgRatio	pvalue	p.adjust	qvalue	geneID	Count
	morphology or physiology							
GO:0060443	mammary gland morphogenesis	6/293	46/18670	7,84E-05	0,000342	0,000151	*SRC/AR/ESR1/BAX/TGFB1/PGR*	6
GO:0045446	endothelial cell differentiation	9/293	114/18670	7,85E-05	0,000342	0,000151	*NR2F2/TNFRSF1A/ACVR1/IL1B/TNF/PROC/CEACAM1/VEGFA/KDR*	9
GO:0006909	phagocytosis	17/293	369/18670	7,95E-05	0,000346	0,000153	*AIF1/SRC/TLR4/GATA2/PPARG/IL1B/TNF/MBL2/LEPR/ITGB3/HMGB1/SLC11A1/CD14/ITGA2/TGFB1/IFNG/LEP*	17
GO:0042226	interleukin-6 biosynthetic process	5/293	29/18670	8,02E-05	0,000348	0,000153	*STAT3/IL1B/TNF/CEBPB/TLR1*	5
GO:0070102	interleukin-6-mediated signaling pathway	5/293	29/18670	8,02E-05	0,000348	0,000153	*STAT3/JAK2/IL6/CEBPA/IL6R*	5
GO:0072538	T-helper 17 type immune response	5/293	29/18670	8,02E-05	0,000348	0,000153	*STAT3/IL6/IL12B/IL23R/FOXP3*	5
GO:0018105	peptidyl-serine phosphorylation	15/293	299/18670	8,17E-05	0,000354	0,000156	*SRC/AKT1/EGFR/CHUK/MTOR/TGFBR1/TNF/IL6/SGK1/PRKG1/BAX/BCL2/VEGFA/TGFB1/IFNG*	15
GO:0072006	nephron development	10/293	142/18670	8,35E-05	0,000362	0,000159	*ACTA2/BMP2/AGT/BCL2/VEGFA/TGFB1/SULF1/FGF1/PGF/IL6R*	10
GO:0006096	glycolytic process	9/293	115/18670	8,40E-05	0,000363	0,00016	*TP53/STAT3/ARNT/HIF1A/INSR/INS/IFNG/SLC4A1/IGF1*	9
GO:1904375	regulation of protein localization to cell periphery	9/293	115/18670	8,40E-05	0,000363	0,00016	*AKT1/EGFR/AR/LGALS3/TNF/BCL2L1/TGFB1/INS/IFNG*	9
GO:0009206	purine ribonucleoside triphosphate biosynthetic process	11/293	171/18670	8,44E-05	0,000365	0,000161	*TP53/STAT3/ARNT/HIF1A/TGFB1/INSR/INS/IFNG/SLC4A1/IGF1/IL4*	11
GO:0010717	regulation of epithelial to mesenchymal transition	8/293	90/18670	8,51E-05	0,000367	0,000162	*MTOR/ACVR1/TGFBR1/BMP2/IL1B/IL6/TGFB1/COL1A1*	8
GO:0031058	positive regulation of histone modification	8/293	90/18670	8,51E-05	0,000367	0,000162	*TP53/IL1B/UBE2N/BRCA1/VEGFA/TGFB1/FMR1/FOXP3*	8
GO:0060537	muscle tissue development	18/293	408/18670	8,55E-05	0,000368	0,000162	*PIM1/ACTA1/MTOR/NR2F2/FGFR1/ACVR1/TGFBR1/BMP2/TP73/AGT/RBP4/BCL2/VEGFA/CREB1/TGFB1/NEB/HAMP/IGF1*	18
GO:0006606	protein import into nucleus	10/293	143/18670	8,85E-05	0,000381	0,000168	*TP53/AKT1/STAT3/RAN/AGT/FLNA/MMP12/TGFB1/IFNG/LEP*	10
GO:0043370	regulation of CD4-positive, alpha-beta T cell differentiation	6/293	47/18670	8,87E-05	0,000381	0,000168	*HMGB1/IL12B/IL23R/FOXP3/IFNG/IL4R*	6
GO:0046638	positive regulation of alpha-beta T cell differentiation	6/293	47/18670	8,87E-05	0,000381	0,000168	*ADA/IL12B/IL23R/FOXP3/IFNG/IL4R*	6

ID	Description	GeneRatio	BgRatio	pvalue	p.adjust	qvalue	geneID	Count
GO:0070169	positive regulation of biomineral tissue development	6/293	47/18670	8,87E-05	0,000381	0,000168	*ACVR1/BMP2/ADRB2/LTF/CEBPB/TGFB1*	6
GO:0098868	bone growth	6/293	47/18670	8,87E-05	0,000381	0,000168	*MBL2/LEPR/COL6A3/COL6A1/FGFR3/LEP*	6
GO:0009145	purine nucleoside triphosphate biosynthetic process	11/293	172/18670	8,89E-05	0,000381	0,000168	*TP53/STAT3/ARNT/HIF1A/TGFB1/INSR/INS/IFNG/SLC4A1/IGF1/IL4*	11
GO:0006757	ATP generation from ADP	9/293	116/18670	8,98E-05	0,000384	0,000169	*TP53/STAT3/ARNT/HIF1A/INSR/INS/IFNG/SLC4A1/IGF1*	9
GO:1900180	regulation of protein localization to nucleus	9/293	116/18670	8,98E-05	0,000384	0,000169	*SRC/AKT1/RAN/F2/FLNA/TGFB1/INS/IFNG/LEP*	9
GO:0031668	cellular response to extracellular stimulus	14/293	268/18670	9,18E-05	0,000393	0,000173	*PIM1/TP53/AIF1/MDM2/MTOR/PPARG/GSTP1/BCL2/BRIP1/LCN2/COL1A1/EIF2AK2/HFE/LEP*	14
GO:0045638	negative regulation of myeloid cell differentiation	8/293	91/18670	9,20E-05	0,000393	0,000173	*TLR4/GATA2/INHA/LTF/LILRB1/CEACAM1/FBN1/IL4*	8
GO:0002643	regulation of tolerance induction	4/293	16/18670	9,32E-05	0,000396	0,000175	*HAVCR2/HMGB1/HLA-G/FOXP3*	4
GO:0010934	macrophage cytokine production	4/293	16/18670	9,32E-05	0,000396	0,000175	*TLR4/HLA-G/LILRB1/TGFB1*	4
GO:0042033	chemokine biosynthetic process	4/293	16/18670	9,32E-05	0,000396	0,000175	*IL1B/TNF/IL6/IFNG*	4
GO:0050755	chemokine metabolic process	4/293	16/18670	9,32E-05	0,000396	0,000175	*IL1B/TNF/IL6/IFNG*	4
GO:0060965	negative regulation of gene silencing by miRNA	4/293	16/18670	9,32E-05	0,000396	0,000175	*TP53/ESR1/PPARG/TGFB1*	4
GO:0046323	glucose import	7/293	68/18670	9,33E-05	0,000397	0,000175	*AKT1/TNF/INSR/INS/HNF1A/IGF1/LEP*	7
GO:0043372	positive regulation of CD4-positive, alpha-beta T cell differentiation	5/293	30/18670	9,50E-05	0,000402	0,000177	*IL12B/IL23R/FOXP3/IFNG/IL4R*	5
GO:0048147	negative regulation of fibroblast proliferation	5/293	30/18670	9,50E-05	0,000402	0,000177	*TP53/PPARG/GSTP1/BAX/LTA*	5
GO:0048873	homeostasis of number of cells within a tissue	5/293	30/18670	9,50E-05	0,000402	0,000177	*NOS3/GATA2/GATA1/BAX/BCL2*	5
GO:0050869	negative regulation of B cell activation	5/293	30/18670	9,50E-05	0,000402	0,000177	*CASP3/INHA/CTLA4/FOXP3/IL10*	5
GO:1901222	regulation of NIK/NF-kappaB signaling	9/293	117/18670	9,60E-05	0,000406	0,000179	*TLR4/EGFR/IL1B/TNF/HAVCR2/HMGB1/CD14/IL12B/EIF2AK2*	9

ID	Description	GeneRatio	BgRatio	pvalue	p.adjust	qvalue	geneID	Count
GO:0001774	microglial cell activation	6/293	48/18670	0,0001	0,000422	0,000186	*AIF1/JAK2/TNF/IL6/C5AR1/IFNG*	6
GO:0002269	leukocyte activation involved in inflammatory response	6/293	48/18670	0,0001	0,000422	0,000186	*AIF1/JAK2/TNF/IL6/C5AR1/IFNG*	6
GO:0007595	lactation	6/293	48/18670	0,0001	0,000422	0,000186	*SERPINC1/PRLR/HIF1A/VEGFA/CREB1/PRL*	6
GO:0010862	positive regulation of pathway-restricted SMAD protein phosphorylation	6/293	48/18670	0,0001	0,000422	0,000186	*ACVR1/TGFBR1/BMP2/INHA/TGFB1/HFE*	6
GO:0072604	interleukin-6 secretion	6/293	48/18670	0,0001	0,000422	0,000186	*AIF1/IL1B/TNF/HMGB1/LEP/NOS2*	6
GO:0032872	regulation of stress-activated MAPK cascade	13/293	237/18670	0,0001	0,000422	0,000186	*AKT1/TLR4/EGFR/IGF1R/GSTP1/BMP2/IL1B/TNF/HMGB 1/VEGFA/EIF2AK2/IL1RN/LEP*	13
GO:0032092	positive regulation of protein binding	8/293	93/18670	0,000107	0,000451	0,000199	*ACE/LGALS3/BMP2/RAN/APOE/MMP9/HFE/MAPRE3*	8
GO:0036473	cell death in response to oxidative stress	8/293	93/18670	0,000107	0,000451	0,000199	*AKT1/TLR4/JAK2/HIF1A/BCL2/MMP3/INS/IL10*	8
GO:0009144	purine nucleoside triphosphate metabolic process	16/293	342/18670	0,000108	0,000452	0,000199	*TP53/STAT3/BAD/HSPA1B/HSPA1A/ADA/RAN/ARNT/HIF1 A/TGFB1/INSR/INS/IFNG/SLC4A1/IGF1/IL4*	16
GO:0070302	regulation of stress-activated protein kinase signaling cascade	13/293	239/18670	0,000109	0,000457	0,000201	*AKT1/TLR4/EGFR/IGF1R/GSTP1/BMP2/IL1B/TNF/HMGB 1/VEGFA/EIF2AK2/IL1RN/LEP*	13
GO:0009408	response to heat	11/293	176/18670	0,000109	0,000457	0,000201	*HSPA1L/AKT1/NOS3/MTOR/HSPA1B/HSPA1A/CPB2/CD14 /FGF1/IGF1/IL1A*	11
GO:0042866	pyruvate biosynthetic process	9/293	119/18670	0,000109	0,000458	0,000202	*TP53/STAT3/ARNT/HIF1A/INSR/INS/IFNG/SLC4A1/IGF1*	9
GO:0034284	response to monosaccharide	12/293	207/18670	0,00011	0,00046	0,000203	*CASP3/BAD/IGF1R/GSTP1/ADRA2A/HIF1A/HNF4A/CPB2 /ITGA2/CFTR/TGFB1/LEP*	12
GO:0061351	neural precursor cell proliferation	10/293	147/18670	0,000111	0,000464	0,000205	*TP53/GATA2/FGFR1/HIF1A/FLNA/C5AR1/VEGFA/TGFB1 /EGF/VEGFC*	10
GO:0002675	positive regulation of acute inflammatory response	5/293	31/18670	0,000112	0,000464	0,000205	*IL1B/TNF/IL6/F12/HLA-E*	5
GO:0002724	regulation of T cell cytokine production	5/293	31/18670	0,000112	0,000464	0,000205	*IL1B/IL6/IL1R1/FOXP3/HFE*	5
GO:0010165	response to X-ray	5/293	31/18670	0,000112	0,000464	0,000205	*TP53/CASP3/BRCA2/THBD/HAMP*	5
GO:0033687	osteoblast proliferation	5/293	31/18670	0,000112	0,000464	0,000205	*GATA1/BMP2/BCL2/LTF/EIF2AK2*	5

ID	Description	GeneRatio	BgRatio	pvalue	p.adjust	qvalue	geneID	Count
GO:0035767	endothelial cell chemotaxis	5/293	31/18670	0,000112	0,000464	0,000205	*FGFR1/HRG/VEGFA/FGF1/KDR*	5
GO:0044319	wound healing, spreading of cells	5/293	31/18670	0,000112	0,000464	0,000205	*MTOR/FLNA/MMP12/CEACAM1/COL5A1*	5
GO:0090505	epiboly involved in wound healing	5/293	31/18670	0,000112	0,000464	0,000205	*MTOR/FLNA/MMP12/CEACAM1/COL5A1*	5
GO:0035094	response to nicotine	6/293	49/18670	0,000113	0,000467	0,000206	*NFKB1/CASP3/BAD/TNF/BCL2/CREB1*	6
GO:0097300	programmed necrotic cell death	6/293	49/18670	0,000113	0,000467	0,000206	*TP53/TLR4/TNF/BAX/CD14/FASLG*	6
GO:0009201	ribonucleoside triphosphate biosynthetic process	11/293	177/18670	0,000115	0,000475	0,000209	*TP53/STAT3/ARNT/HIF1A/TGFB1/INSR/INS/IFNG/SLC4A1/IGF1/IL4*	11
GO:0002792	negative regulation of peptide secretion	10/293	148/18670	0,000118	0,000488	0,000215	*FN1/ADRA2A/IL1B/TNF/APOE/LILRB1/INS/FOXP3/LEP/IL10*	10
GO:0006914	autophagy	20/293	496/18670	0,000119	0,000492	0,000217	*TP53/SRC/AKT1/STAT3/CASP3/MTOR/BAD/IKBKG/ADRB2/IL10RA/LEPR/HIF1A/BCL2/ITGB4/HMGB1/KDR/IFNG/IL4/LEP/IL10*	20
GO:0061919	process utilizing autophagic mechanism	20/293	496/18670	0,000119	0,000492	0,000217	*TP53/SRC/AKT1/STAT3/CASP3/MTOR/BAD/IKBKG/ADRB2/IL10RA/LEPR/HIF1A/BCL2/ITGB4/HMGB1/KDR/IFNG/IL4/LEP/IL10*	20
GO:0010744	positive regulation of macrophage derived foam cell differentiation	4/293	17/18670	0,00012	0,000494	0,000218	*NFKB1/AGTR1/AGT/APOB*	4
GO:0030540	female genitalia development	4/293	17/18670	0,00012	0,000494	0,000218	*ESR1/TP63/RBP4/BAX*	4
GO:0031649	heat generation	4/293	17/18670	0,00012	0,000494	0,000218	*ADRB2/IL1B/TNF/IL1A*	4
GO:0032740	positive regulation of interleukin-17 production	4/293	17/18670	0,00012	0,000494	0,000218	*IL6/TGFB1/IL12B/IL23R*	4
GO:0045072	regulation of interferon-gamma biosynthetic process	4/293	17/18670	0,00012	0,000494	0,000218	*INHA/LILRB1/IL12B/FOXP3*	4
GO:0070242	thymocyte apoptotic process	4/293	17/18670	0,00012	0,000494	0,000218	*TP53/ADA/HIF1A/BAX*	4
GO:2000811	negative regulation of anoikis	4/293	17/18670	0,00012	0,000494	0,000218	*SRC/BCL2/CEACAM6/CEACAM5*	4
GO:0050795	regulation of behavior	7/293	71/18670	0,000123	0,000505	0,000222	*STAT3/MTOR/ADA/LEPR/HTR1A/APOE/INS*	7
GO:0051196	regulation of coenzyme metabolic process	8/293	95/18670	0,000125	0,00051	0,000225	*TP53/STAT3/ARNT/HIF1A/INSR/INS/IFNG/IGF1*	8
GO:0060337	type I interferon signaling pathway	8/293	95/18670	0,000125	0,00051	0,000225	*MMP12/HLA-E/HLA-C/HLA-G/HLA-A/HLA-B/IFI6/IFI35*	8

ID	Description	GeneRatio	BgRatio	pvalue	p.adjust	qvalue	geneID	Count
GO:0071357	cellular response to type I interferon	8/293	95/18670	0,000125	0,00051	0,000225	*MMP12/HLA-E/HLA-C/HLA-G/HLA-A/HLA-B/IFI6/IFI35*	8
GO:0009266	response to temperature stimulus	13/293	243/18670	0,000129	0,000526	0,000232	*HSPA1L/AKT1/NOS3/MTOR/HSPA1B/HSPA1A/PPARG/ADRB2/CPB2/CD14/FGF1/IGF1/IL1A*	13
GO:0042311	vasodilation	5/293	32/18670	0,000131	0,000533	0,000235	*NOS3/ADRB2/AGT/PRKG1/APOE*	5
GO:0045589	regulation of regulatory T cell differentiation	5/293	32/18670	0,000131	0,000533	0,000235	*HLA-G/TGFB1/CTLA4/FOXP3/IFNG*	5
GO:0045907	positive regulation of vasoconstriction	5/293	32/18670	0,000131	0,000533	0,000235	*AKT1/EGFR/FGG/FGB/FGA*	5
GO:0055094	response to lipoprotein particle	5/293	32/18670	0,000131	0,000533	0,000235	*AKT1/TLR4/PPARG/APOE/CD9*	5
GO:0090504	epiboly	5/293	32/18670	0,000131	0,000533	0,000235	*MTOR/FLNA/MMP12/CEACAM1/COL5A1*	5
GO:0019730	antimicrobial humoral response	9/293	122/18670	0,000132	0,000538	0,000237	*F2/HRG/FGB/FGA/SLC11A1/LTF/HLA-E/LCN2/CXCL8*	9
GO:1903578	regulation of ATP metabolic process	9/293	122/18670	0,000132	0,000538	0,000237	*TP53/STAT3/ARNT/HIF1A/INSR/INS/IFNG/IGF1/IL4*	9
GO:0071466	cellular response to xenobiotic stimulus	11/293	180/18670	0,000133	0,000541	0,000238	*AIF1/STAT5B/NR3C1/EGFR/GSTP1/AHR/ARNT/ARNT2/HNF4A/CASP9/TGFB1*	11
GO:0010660	regulation of muscle cell apoptotic process	8/293	96/18670	0,000134	0,000544	0,00024	*TP53/PPARG/JAK2/AGT/IL12B/IFNG/IGF1/IL12A*	8
GO:0097327	response to antineoplastic agent	8/293	96/18670	0,000134	0,000544	0,00024	*NR3C1/EGFR/CASP9/F7/BRCA1/TGFB1/DHFR/FMR1*	8
GO:0007566	embryo implantation	6/293	51/18670	0,000141	0,000571	0,000252	*IL1B/PRLR/MMP2/TIMP1/MMP9/FBLN1*	6
GO:0048146	positive regulation of fibroblast proliferation	6/293	51/18670	0,000141	0,000571	0,000252	*EGFR/ESR1/FN1/AGT/TGFB1/IGF1*	6
GO:0048260	positive regulation of receptor-mediated endocytosis	6/293	51/18670	0,000141	0,000571	0,000252	*SERPINE1/VEGFA/EGF/FMR1/HFE/IL4*	6
GO:0001657	ureteric bud development	8/293	97/18670	0,000144	0,000582	0,000256	*FGFR1/BMP2/AGT/BCL2/VEGFA/TGFB1/FGF1/PGF*	8
GO:0007044	cell-substrate junction assembly	8/293	97/18670	0,000144	0,000582	0,000256	*SRC/FN1/HRG/BCL2/ITGB4/ITGA2/VEGFA/KDR*	8
GO:1902882	regulation of response to oxidative stress	8/293	97/18670	0,000144	0,000582	0,000256	*AKT1/TLR4/TNF/HIF1A/MMP3/DHFR/INS/IL10*	8
GO:0060047	heart contraction	14/293	280/18670	0,000146	0,000586	0,000258	*ACE/ACE2/THRB/MDM2/NOS3/MTOR/JAK2/ADA/AGT/FLNA/DES/VEGFB/TRPC1/PDE5A*	14
GO:0002312	B cell activation involved in immune response	7/293	73/18670	0,000147	0,00059	0,00026	*TLR4/ADA/TGFB1/FOXP3/TFRC/IL4/IL10*	7
GO:1903524	positive regulation of blood circulation	7/293	73/18670	0,000147	0,00059	0,00026	*ACE2/AKT1/EGFR/ADA/FGG/FGB/FGA*	7

ID	Description	GeneRatio	BgRatio	pvalue	p.adjust	qvalue	geneID	Count
GO:0009166	nucleotide catabolic process	11/293	182/18670	0,000147	0,00059	0,00026	*TP53/STAT3/ADA/ARNT/HIF1A/INSR/INS/IFNG/SLC4A1/IGF1/PDE5A*	11
GO:0016051	carbohydrate biosynthetic process	12/293	214/18670	0,00015	0,000603	0,000266	*NFKB1/AKT1/MTOR/RBP4/LEPR/TGFB1/EGF/INSR/INS/IGF2/IGF1/LEP*	12
GO:0010039	response to iron ion	5/293	33/18670	0,000152	0,000606	0,000267	*MDM2/HIF1A/BCL2/HAMP/HFE*	5
GO:0045191	regulation of isotype switching	5/293	33/18670	0,000152	0,000606	0,000267	*TGFB1/FOXP3/TFRC/IL4/IL10*	5
GO:0060603	mammary gland duct morphogenesis	5/293	33/18670	0,000152	0,000606	0,000267	*SRC/AR/ESR1/TGFB1/PGR*	5
GO:0070232	regulation of T cell apoptotic process	5/293	33/18670	0,000152	0,000606	0,000267	*TP53/LGALS3/ADA/HIF1A/TSC22D3*	5
GO:1901890	positive regulation of cell junction assembly	5/293	33/18670	0,000152	0,000606	0,000267	*ACE2/AGT/HRG/VEGFA/KDR*	5
GO:1902692	regulation of neuroblast proliferation	5/293	33/18670	0,000152	0,000606	0,000267	*TP53/HIF1A/VEGFA/TGFB1/VEGFC*	5
GO:0032693	negative regulation of interleukin-10 production	4/293	18/18670	0,000153	0,000606	0,000267	*LILRB1/IL12B/IL23R/FOXP3*	4
GO:0036035	osteoclast development	4/293	18/18670	0,000153	0,000606	0,000267	*SRC/LTF/LILRB1/FBN1*	4
GO:0042095	interferon-gamma biosynthetic process	4/293	18/18670	0,000153	0,000606	0,000267	*INHA/LILRB1/IL12B/FOXP3*	4
GO:0044320	cellular response to leptin stimulus	4/293	18/18670	0,000153	0,000606	0,000267	*STAT3/LEPR/FGB/LEP*	4
GO:0051023	regulation of immunoglobulin secretion	4/293	18/18670	0,000153	0,000606	0,000267	*TNF/IL6/RBP4/HLA-E*	4
GO:0090196	regulation of chemokine secretion	4/293	18/18670	0,000153	0,000606	0,000267	*AIF1/TNF/CHIA/IL4R*	4
GO:0090330	regulation of platelet aggregation	4/293	18/18670	0,000153	0,000606	0,000267	*FGG/PRKG1/CD9/CEACAM1*	4
GO:1900221	regulation of amyloid-beta clearance	4/293	18/18670	0,000153	0,000606	0,000267	*TNF/APOE/IFNG/IL4*	4
GO:0060191	regulation of lipase activity	8/293	98/18670	0,000155	0,000614	0,00027	*EGFR/FGFR1/ESR1/AGTR1/AGT/C5AR1/FGFR3/FLT1*	8
GO:0072163	mesonephric epithelium development	8/293	98/18670	0,000155	0,000614	0,00027	*FGFR1/BMP2/AGT/BCL2/VEGFA/TGFB1/FGF1/PGF*	8
GO:0072164	mesonephric tubule development	8/293	98/18670	0,000155	0,000614	0,00027	*FGFR1/BMP2/AGT/BCL2/VEGFA/TGFB1/FGF1/PGF*	8
GO:0003179	heart valve morphogenesis	6/293	52/18670	0,000158	0,000622	0,000274	*MDM2/NOS3/MTOR/ACVR1/BMP2/TGFB1*	6

ID	Description	GeneRatio	BgRatio	pvalue	p.adjust	qvalue	geneID	Count
GO:0032653	regulation of interleukin-10 production	6/293	52/18670	0,000158	0,000622	0,000274	*TLR4/HMGB1/LILRB1/IL12B/IL23R/FOXP3*	6
GO:0005977	glycogen metabolic process	7/293	74/18670	0,00016	0,00063	0,000278	*AKT1/MTOR/LEPR/INSR/INS/IGF2/IGF1*	7
GO:0072078	nephron tubule morphogenesis	7/293	74/18670	0,00016	0,00063	0,000278	*BMP2/AGT/BCL2/VEGFA/TGFB1/FGF1/PGF*	7
GO:0001933	negative regulation of protein phosphorylation	18/293	429/18670	0,00016	0,00063	0,000278	*AKT1/TLR4/CASP3/MTOR/NR2F2/IGF1R/IGFBP3/GSTP1/IL1B/BAX/APC/APOE/TIMP3/CEACAM1/CEBPA/TGFB1/FBLN1/IFNG*	18
GO:0051052	regulation of DNA metabolic process	18/293	429/18670	0,00016	0,00063	0,000278	*TP53/SRC/AKT1/EGFR/FGFR1/PPARG/IL6/UBE2N/BAX/DNA2/BRCA1/BRCA2/HMGB1/TGFB1/FOXP3/TFRC/IL4/IL10*	18
GO:0034340	response to type I interferon	8/293	99/18670	0,000166	0,000655	0,000289	*MMP12/HLA-E/HLA-C/HLA-G/HLA-A/HLA-B/IFI6/IFI35*	8
GO:0031647	regulation of protein stability	14/293	284/18670	0,000169	0,000663	0,000292	*PIM1/TP53/SRC/VHL/MDM2/CASP3/HSPA1B/HSPA1A/BMP2/FLNA/BCL2/BAG6/CREB1/IGF1*	14
GO:0032479	regulation of type I interferon production	9/293	126/18670	0,000169	0,000665	0,000293	*NFKB1/TLR4/CHUK/MMP12/HAVCR2/HMGB1/CD14/LILRB1/IL10*	9
GO:1902275	regulation of chromatin organization	11/293	185/18670	0,00017	0,000665	0,000293	*TP53/GATA2/IL1B/UBE2N/BRCA1/CTCFL/VEGFA/TGFB1/IGF2/FMR1/FOXP3*	11
GO:0006073	cellular glucan metabolic process	7/293	75/18670	0,000174	0,000682	0,0003	*AKT1/MTOR/LEPR/INSR/INS/IGF2/IGF1*	7
GO:0044042	glucan metabolic process	7/293	75/18670	0,000174	0,000682	0,0003	*AKT1/MTOR/LEPR/INSR/INS/IGF2/IGF1*	7
GO:0051348	negative regulation of transferase activity	14/293	285/18670	0,000175	0,000684	0,000301	*TP53/SRC/AKT1/CASP3/NR2F2/IGF1R/PPARG/GSTP1/IL1B/APC/APOE/CEACAM1/CEBPA/IFNG*	14
GO:0000737	DNA catabolic process, endonucleolytic	5/293	34/18670	0,000176	0,000684	0,000301	*CASP3/IL6/BAX/HMGB1/DICER1*	5
GO:0007094	mitotic spindle assembly checkpoint	5/293	34/18670	0,000176	0,000684	0,000301	*LCMT1/APC/BUB1/BUB1B/BUB3*	5
GO:0007435	salivary gland morphogenesis	5/293	34/18670	0,000176	0,000684	0,000301	*EGFR/FGFR1/TNF/TGFB1/FGF7*	5
GO:0010922	positive regulation of phosphatase activity	5/293	34/18670	0,000176	0,000684	0,000301	*MTOR/JAK2/BMP2/ITGA2/IFNG*	5
GO:0031577	spindle checkpoint	5/293	34/18670	0,000176	0,000684	0,000301	*LCMT1/APC/BUB1/BUB1B/BUB3*	5
GO:0034205	amyloid-beta formation	5/293	34/18670	0,000176	0,000684	0,000301	*CASP3/TNF/APOE/IFNG/IGF1*	5
GO:0045066	regulatory T cell differentiation	5/293	34/18670	0,000176	0,000684	0,000301	*HLA-G/TGFB1/CTLA4/FOXP3/IFNG*	5
GO:0071173	spindle assembly checkpoint	5/293	34/18670	0,000176	0,000684	0,000301	*LCMT1/APC/BUB1/BUB1B/BUB3*	5

ID	Description	GeneRatio	BgRatio	pvalue	p.adjust	qvalue	geneID	Count
GO:0071174	mitotic spindle checkpoint	5/293	34/18670	0,000176	0,000684	0,000301	*LCMT1/APC/BUB1/BUB1B/BUB3*	5
GO:0071402	cellular response to lipoprotein particle stimulus	5/293	34/18670	0,000176	0,000684	0,000301	*AKT1/TLR4/PPARG/APOE/CD9*	5
GO:0051480	regulation of cytosolic calcium ion concentration	16/293	357/18670	0,000176	0,000684	0,000301	*TRPC6/GATA2/ESR1/JAK2/AGTR1/CCR5/AGT/F2/BAX/BCL2/HMGB1/C5AR1/TGFB1/CXCR1/FASLG/TRPC1*	16
GO:0030308	negative regulation of cell growth	11/293	186/18670	0,000178	0,000689	0,000304	*TP53/ESR2/HSPA1B/HSPA1A/PPARG/AGT/HRG/HNF4A/BCL2/MAP2/TGFB1*	11
GO:0010657	muscle cell apoptotic process	8/293	100/18670	0,000178	0,000691	0,000304	*TP53/PPARG/JAK2/AGT/IL12B/IFNG/IGF1/IL12A*	8
GO:0034446	substrate adhesion-dependent cell spreading	8/293	100/18670	0,000178	0,000691	0,000304	*SRC/FN1/FGG/FLNA/FGB/FGA/ITGB3/FBLN1*	8
GO:0034767	positive regulation of ion transmembrane transport	10/293	156/18670	0,000182	0,000703	0,00031	*ABCB1/TRPC6/ADRB2/AGT/F2/FLNA/BAX/CFTR/IFNG/TRPC1*	10
GO:0018209	peptidyl-serine modification	15/293	322/18670	0,000185	0,000714	0,000314	*SRC/AKT1/EGFR/CHUK/MTOR/TGFBR1/TNF/IL6/SGK1/PRKG1/BAX/BCL2/VEGFA/TGFB1/IFNG*	15
GO:0030500	regulation of bone mineralization	7/293	76/18670	0,000189	0,00073	0,000322	*GATA1/ACVR1/BMP2/ADRB2/HIF1A/LTF/TGFB1*	7
GO:0072088	nephron epithelium morphogenesis	7/293	76/18670	0,000189	0,00073	0,000322	*BMP2/AGT/BCL2/VEGFA/TGFB1/FGF1/PGF*	7
GO:0032606	type I interferon production	9/293	128/18670	0,000191	0,000732	0,000323	*NFKB1/TLR4/CHUK/MMP12/HAVCR2/HMGB1/CD14/LILRB1/IL10*	9
GO:0002544	chronic inflammatory response	4/293	19/18670	0,000191	0,000732	0,000323	*TNF/FOXP3/LTA/IL10*	4
GO:0032930	positive regulation of superoxide anion generation	4/293	19/18670	0,000191	0,000732	0,000323	*EGFR/GSTP1/AGT/TGFB1*	4
GO:0034755	iron ion transmembrane transport	4/293	19/18670	0,000191	0,000732	0,000323	*SLC11A1/IFNG/HAMP/HFE*	4
GO:0060149	negative regulation of posttranscriptional gene silencing	4/293	19/18670	0,000191	0,000732	0,000323	*TP53/ESR1/PPARG/TGFB1*	4
GO:0060967	negative regulation of gene silencing by RNA	4/293	19/18670	0,000191	0,000732	0,000323	*TP53/ESR1/PPARG/TGFB1*	4
GO:1903798	regulation of production of miRNAs involved in	4/293	19/18670	0,000191	0,000732	0,000323	*TP53/EGFR/ESR1/TGFB1*	4

ID	Description	GeneRatio	BgRatio	pvalue	p.adjust	qvalue	geneID	Count
	gene silencing by miRNA							
GO:0007631	feeding behavior	8/293	101/18670	0,000191	0,000732	0,000323	*ACE2/STAT3/MTOR/AGT/REN/LEPR/INS/LEP*	8
GO:0050830	defense response to Gram-positive bacterium	8/293	101/18670	0,000191	0,000732	0,000323	*TNF/IL6/MBL2/HAVCR2/C5AR1/HLA-E/IL12A/LTA*	8
GO:0002763	positive regulation of myeloid leukocyte differentiation	6/293	54/18670	0,000195	0,000744	0,000328	*TNF/CREB1/TGFB1/IL12B/IL23R/IFNG*	6
GO:0050994	regulation of lipid catabolic process	6/293	54/18670	0,000195	0,000744	0,000328	*AKT1/MTOR/ADRA2A/IL1B/TNF/INS*	6
GO:1901292	nucleoside phosphate catabolic process	11/293	188/18670	0,000195	0,000745	0,000328	*TP53/STAT3/ADA/ARNT/HIF1A/INSR/INS/IFNG/SLC4A1/IGF1/PDE5A*	11
GO:0009132	nucleoside diphosphate metabolic process	10/293	158/18670	0,000202	0,000769	0,000339	*TP53/STAT3/BAD/ARNT/HIF1A/INSR/INS/IFNG/SLC4A1/IGF1*	10
GO:0019724	B cell mediated immunity	12/293	221/18670	0,000203	0,000771	0,00034	*TNF/MBL2/HLA-E/HLA-G/TGFB1/FOXP3/TFRC/HLA-DQB1/IL4R/IL4/LTA/IL10*	12
GO:0003203	endocardial cushion morphogenesis	5/293	35/18670	0,000203	0,000771	0,00034	*MDM2/NOS3/ACVR1/TGFBR1/BMP2*	5
GO:0030262	apoptotic nuclear changes	5/293	35/18670	0,000203	0,000771	0,00034	*CASP3/IL6/BAX/HMGB1/DICER1*	5
GO:0070423	nucleotide-binding oligomerization domain containing signaling pathway	5/293	35/18670	0,000203	0,000771	0,00034	*TLR4/IKBKG/HSPA1B/HSPA1A/UBE2N*	5
GO:1902991	regulation of amyloid precursor protein catabolic process	5/293	35/18670	0,000203	0,000771	0,00034	*CASP3/TNF/APOE/IFNG/IGF1*	5
GO:1905332	positive regulation of morphogenesis of an epithelium	5/293	35/18670	0,000203	0,000771	0,00034	*MTOR/AR/AGT/VEGFA/TGFB1*	5
GO:0000079	regulation of cyclin-dependent protein serine/threonine kinase activity	8/293	102/18670	0,000205	0,000776	0,000342	*SRC/AKT1/EGFR/CASP3/NR2F2/APC/CEBPA/MAPRE3*	8
GO:0098869	cellular oxidant detoxification	8/293	102/18670	0,000205	0,000776	0,000342	*NOS3/GSTP1/TNF/HBE1/APOE/DHFR/HBA1/HP*	8
GO:0090596	sensory organ morphogenesis	13/293	256/18670	0,000215	0,000815	0,000359	*STAT3/THRB/GATA2/FGFR1/RBP4/HIF1A/BAX/BCL2/VEGFA/FBN1/COL5A1/COL5A2/FASLG*	13
GO:0002011	morphogenesis of an epithelial sheet	6/293	55/18670	0,000216	0,000815	0,000359	*MTOR/FLNA/FLRT3/MMP12/CEACAM1/COL5A1*	6
GO:0032613	interleukin-10 production	6/293	55/18670	0,000216	0,000815	0,000359	*TLR4/HMGB1/LILRB1/IL12B/IL23R/FOXP3*	6

ID	Description	GeneRatio	BgRatio	pvalue	p.adjust	qvalue	geneID	Count
GO:0043030	regulation of macrophage activation	6/293	55/18670	0,000216	0,000815	0,000359	*TLR4/IL6/HAVCR2/CEBPA/IL4R/IL10*	6
GO:1905269	positive regulation of chromatin organization	8/293	103/18670	0,000219	0,000827	0,000365	*TP53/IL1B/UBE2N/BRCA1/VEGFA/TGFB1/FMR1/FOXP3*	8
GO:0010827	regulation of glucose transmembrane transport	7/293	78/18670	0,000223	0,000839	0,00037	*AKT1/IL1B/TNF/INSR/INS/IGF1/LEP*	7
GO:0061333	renal tubule morphogenesis	7/293	78/18670	0,000223	0,000839	0,00037	*BMP2/AGT/BCL2/VEGFA/TGFB1/FGF1/PGF*	7
GO:0072028	nephron morphogenesis	7/293	78/18670	0,000223	0,000839	0,00037	*BMP2/AGT/BCL2/VEGFA/TGFB1/FGF1/PGF*	7
GO:0048863	stem cell differentiation	13/293	257/18670	0,000223	0,000841	0,000371	*ACE/TP53/STAT3/GATA2/GATA1/ESR1/FN1/ACVR1/TP63/TP73/HIF1A/EIF2AK2/EFNB1*	13
GO:0055076	transition metal ion homeostasis	9/293	131/18670	0,000227	0,000854	0,000376	*TMPRSS6/HEPH/HIF1A/SLC11A1/LTF/LCN2/HAMP/HFE/TFRC*	9
GO:0034390	smooth muscle cell apoptotic process	5/293	36/18670	0,000233	0,000872	0,000384	*PPARG/IL12B/IFNG/IGF1/IL12A*	5
GO:0034391	regulation of smooth muscle cell apoptotic process	5/293	36/18670	0,000233	0,000872	0,000384	*PPARG/IL12B/IFNG/IGF1/IL12A*	5
GO:0035872	nucleotide-binding domain, leucine rich repeat containing receptor signaling pathway	5/293	36/18670	0,000233	0,000872	0,000384	*TLR4/IKBKG/HSPA1B/HSPA1A/UBE2N*	5
GO:0045841	negative regulation of mitotic metaphase/anaphase transition	5/293	36/18670	0,000233	0,000872	0,000384	*LCMT1/APC/BUB1/BUB1B/BUB3*	5
GO:0090218	positive regulation of lipid kinase activity	5/293	36/18670	0,000233	0,000872	0,000384	*SRC/F2/TGFB1/FGFR3/FLT1*	5
GO:2000516	positive regulation of CD4-positive, alpha-beta T cell activation	5/293	36/18670	0,000233	0,000872	0,000384	*IL12B/IL23R/FOXP3/IFNG/IL4R*	5
GO:0034393	positive regulation of smooth muscle cell apoptotic process	4/293	20/18670	0,000236	0,000879	0,000387	*PPARG/IL12B/IFNG/IL12A*	4
GO:0042535	positive regulation of tumor necrosis factor biosynthetic process	4/293	20/18670	0,000236	0,000879	0,000387	*TLR4/JAK2/IFNG/TLR1*	4
GO:0043931	ossification involved in bone maturation	4/293	20/18670	0,000236	0,000879	0,000387	*BMP2/LTF/IGF1/LEP*	4

ID	Description	GeneRatio	BgRatio	pvalue	p.adjust	qvalue	geneID	Count
GO:0060261	positive regulation of transcription initiation from RNA polymerase II promoter	4/293	20/18670	0,000236	0,000879	0,000387	*TP53/ESR1/CREB1/HNF1A*	4
GO:0090195	chemokine secretion	4/293	20/18670	0,000236	0,000879	0,000387	*AIF1/TNF/CHIA/IL4R*	4
GO:0097062	dendritic spine maintenance	4/293	20/18670	0,000236	0,000879	0,000387	*IGF1R/APOE/INSR/INS*	4
GO:0097709	connective tissue replacement	4/293	20/18670	0,000236	0,000879	0,000387	*HIF1A/TIMP1/TGFB1/IL1A*	4
GO:0002381	immunoglobulin production involved in immunoglobulin mediated immune response	6/293	56/18670	0,000238	0,000886	0,000391	*TGFB1/FOXP3/TFRC/HLA-DQB1/IL4/IL10*	6
GO:0010332	response to gamma radiation	6/293	56/18670	0,000238	0,000886	0,000391	*TP53/MDM2/BAX/BCL2/BCL2L1/BRCA2*	6
GO:0042476	odontogenesis	9/293	132/18670	0,00024	0,000891	0,000393	*SRC/BMP2/TP63/BAX/ITGB4/SERPINE1/TGFB1/COL1A1/COL1A2*	9
GO:0043280	positive regulation of cysteine-type endopeptidase activity involved in apoptotic process	9/293	132/18670	0,00024	0,000891	0,000393	*BAD/PPARG/JAK2/TNF/CASP9/BAX/HMGB1/FASLG/F3*	9
GO:0055007	cardiac muscle cell differentiation	9/293	132/18670	0,00024	0,000891	0,000393	*MTOR/ACVR1/BMP2/AGT/VEGFA/TGFB1/NEB/HAMP/IGF1*	9
GO:0002377	immunoglobulin production	11/293	193/18670	0,000245	0,000907	0,0004	*TNF/IL6/RBP4/HLA-E/TGFB1/FOXP3/TFRC/HLA-DQB1/IL4R/IL4/IL10*	11
GO:0007219	Notch signaling pathway	11/293	193/18670	0,000245	0,000907	0,0004	*AKT1/STAT3/EGFR/NOS3/GATA2/BMP2/TP63/CEBPA/TGFB1/TIMP4/EGF*	11
GO:0050768	negative regulation of neurogenesis	14/293	295/18670	0,000249	0,000923	0,000407	*TP53/STAT3/TRPC6/THRB/MDM2/IL1B/TNF/IL6/TP73/F2/APOE/MAP2/TGFB1/DICER1*	14
GO:0033138	positive regulation of peptidyl-serine phosphorylation	8/293	105/18670	0,00025	0,000924	0,000407	*AKT1/EGFR/TNF/IL6/BCL2/VEGFA/TGFB1/IFNG*	8
GO:0051170	import into nucleus	10/293	163/18670	0,000259	0,000958	0,000422	*TP53/AKT1/STAT3/RAN/AGT/FLNA/MMP12/TGFB1/IFNG/LEP*	10
GO:0042743	hydrogen peroxide metabolic process	6/293	57/18670	0,000263	0,000969	0,000427	*STAT3/EGFR/HBE1/MMP3/HBA1/HP*	6
GO:0043525	positive regulation of neuron apoptotic process	6/293	57/18670	0,000263	0,000969	0,000427	*TP53/CASP3/TNF/CASP9/BAX/FASLG*	6
GO:0048008	platelet-derived growth factor	6/293	57/18670	0,000263	0,000969	0,000427	*SRC/JAK2/F7/PLAT/VEGFA/F3*	6

ID	Description	GeneRatio	BgRatio	pvalue	p.adjust	qvalue	geneID	Count
	receptor signaling pathway							
GO:0060760	positive regulation of response to cytokine stimulus	6/293	57/18670	0,000263	0,000969	0,000427	*TLR4/HSPA1B/HSPA1A/IL1R1/HIF1A/MMP12*	6
GO:0006921	cellular component disassembly involved in execution phase of apoptosis	5/293	37/18670	0,000266	0,000973	0,000429	*CASP3/IL6/BAX/HMGB1/DICER1*	5
GO:0007431	salivary gland development	5/293	37/18670	0,000266	0,000973	0,000429	*EGFR/FGFR1/TNF/TGFB1/FGF7*	5
GO:0014912	negative regulation of smooth muscle cell migration	5/293	37/18670	0,000266	0,000973	0,000429	*AIF1/IGFBP3/GSTP1/PRKG1/SERPINE1*	5
GO:0042307	positive regulation of protein import into nucleus	5/293	37/18670	0,000266	0,000973	0,000429	*RAN/FLNA/TGFB1/IFNG/LEP*	5
GO:0050691	regulation of defense response to virus by host	5/293	37/18670	0,000266	0,000973	0,000429	*IL1B/MMP12/LILRB1/IL12B/IL23R*	5
GO:1902100	negative regulation of metaphase/anaphase transition of cell cycle	5/293	37/18670	0,000266	0,000973	0,000429	*LCMT1/APC/BUB1/BUB1B/BUB3*	5
GO:2000142	regulation of DNA-templated transcription, initiation	5/293	37/18670	0,000266	0,000973	0,000429	*TP53/ESR1/HMGB1/CREB1/HNF1A*	5
GO:2000273	positive regulation of signaling receptor activity	5/293	37/18670	0,000266	0,000973	0,000429	*ADRA2A/ADRB2/EGF/IFNG/HFE*	5
GO:1904029	regulation of cyclin-dependent protein kinase activity	8/293	106/18670	0,000267	0,000976	0,00043	*SRC/AKT1/EGFR/CASP3/NR2F2/APC/CEBPA/MAPRE3*	8
GO:0006165	nucleoside diphosphate phosphorylation	9/293	134/18670	0,000269	0,000982	0,000433	*TP53/STAT3/ARNT/HIF1A/INSR/INS/IFNG/SLC4A1/IGF1*	9
GO:0007006	mitochondrial membrane organization	9/293	134/18670	0,000269	0,000982	0,000433	*TP53/STAT3/BAD/HSPA1A/TP63/TP73/BAX/BCL2/BCL2L1*	9
GO:0072511	divalent inorganic cation transport	19/293	489/18670	0,000281	0,001026	0,000452	*TRPC6/NOS3/LGALS3/TCN2/ADRA2A/CCR5/AGT/F2/IL16/BAX/BCL2/SLC11A1/LILRB1/TGFB1/EGF/FASLG/FMR1/IFNG/TRPC1*	19

ID	Description	GeneRatio	BgRatio	pvalue	p.adjust	qvalue	geneID	Count
GO:0014855	striated muscle cell proliferation	7/293	81/18670	0,000282	0,001026	0,000452	*PIM1/STAT3/FGFR1/JAK2/TGFBR1/TP73/RBP4*	7
GO:0009205	purine ribonucleoside triphosphate metabolic process	15/293	335/18670	0,000283	0,001029	0,000454	*TP53/STAT3/BAD/HSPA1B/HSPA1A/RAN/ARNT/HIF1A/TGFB1/INSR/INS/IFNG/SLC4A1/IGF1/IL4*	15
GO:0043502	regulation of muscle adaptation	8/293	107/18670	0,000285	0,001036	0,000456	*AIF1/NOS3/MTOR/TNFRSF1A/AGT/HAMP/IGF1/PDE5A*	8
GO:0030728	ovulation	4/293	21/18670	0,000288	0,001045	0,000461	*NOS3/PGR/IL4R/LEP*	4
GO:0051900	regulation of mitochondrial depolarization	4/293	21/18670	0,000288	0,001045	0,000461	*SRC/BCL2/IFI6/KDR*	4
GO:0070920	regulation of production of small RNA involved in gene silencing by RNA	4/293	21/18670	0,000288	0,001045	0,000461	*TP53/EGFR/ESR1/TGFB1*	4
GO:1903055	positive regulation of extracellular matrix organization	4/293	21/18670	0,000288	0,001045	0,000461	*IL6/AGT/CPB2/TGFB1*	4
GO:1903749	positive regulation of establishment of protein localization to mitochondrion	6/293	58/18670	0,00029	0,00105	0,000463	*HSPA1L/TP53/BAD/TP63/TP73/BCL2*	6
GO:0006164	purine nucleotide biosynthetic process	14/293	300/18670	0,000296	0,001072	0,000473	*TP53/STAT3/NOS3/ADA/ARNT/HIF1A/TGFB1/INSR/INS/IFNG/SLC4A1/IGF1/IL4/NOS2*	14
GO:0046939	nucleotide phosphorylation	9/293	136/18670	0,0003	0,001087	0,000479	*TP53/STAT3/ARNT/HIF1A/INSR/INS/IFNG/SLC4A1/IGF1*	9
GO:0046636	negative regulation of alpha-beta T cell activation	5/293	38/18670	0,000302	0,001092	0,000481	*HMGB1/LILRB1/FOXP3/HFE/IL4R*	5
GO:1904037	positive regulation of epithelial cell apoptotic process	5/293	38/18670	0,000302	0,001092	0,000481	*BAD/JAK2/IL6/HLA-G/FASLG*	5
GO:1904659	glucose transmembrane transport	8/293	108/18670	0,000303	0,001095	0,000483	*AKT1/IL1B/TNF/INSR/INS/HNF1A/IGF1/LEP*	8
GO:0051702	interaction with symbiont	7/293	82/18670	0,000304	0,001095	0,000483	*FN1/F2/MBL2/APOE/LTF/FMR1/IGF2R*	7
GO:0071158	positive regulation of cell cycle arrest	7/293	82/18670	0,000304	0,001095	0,000483	*TP53/MDM2/TP73/BAX/BRCA1/CNOT1/TGFB1*	7
GO:0009123	nucleoside monophosphate metabolic process	16/293	375/18670	0,000306	0,001103	0,000486	*TP53/STAT3/BAD/HSPA1B/HSPA1A/ADA/ARNT/HIF1A/TYMS/TGFB1/INSR/INS/IFNG/SLC4A1/IGF1/IL4*	16

ID	Description	GeneRatio	BgRatio	pvalue	p.adjust	qvalue	geneID	Count
GO:0033209	tumor necrosis factor-mediated signaling pathway	10/293	167/18670	0,000315	0,001134	0,0005	*TRAF1/CHUK/IKBKG/TNFRSF1A/HSPA1B/HSPA1A/JAK2/GSTP1/TNF/LTA*	10
GO:0035051	cardiocyte differentiation	10/293	167/18670	0,000315	0,001134	0,0005	*EGFR/MTOR/ACVR1/BMP2/AGT/VEGFA/TGFB1/NEB/HAMP/IGF1*	10
GO:0031644	regulation of neurological system process	9/293	137/18670	0,000317	0,00114	0,000502	*SRC/NOS3/MTOR/ADRB2/AGT/ITGA2/DICER1/FMR1/IL10*	9
GO:0016052	carbohydrate catabolic process	11/293	199/18670	0,000319	0,001144	0,000504	*TP53/STAT3/BAD/ARNT/HIF1A/CHIA/INSR/INS/IFNG/SLC4A1/IGF1*	11
GO:0017038	protein import	11/293	199/18670	0,000319	0,001144	0,000504	*TP53/AKT1/STAT3/RAN/AGT/FLNA/APOE/MMP12/TGFB1/IFNG/LEP*	11
GO:0072331	signal transduction by p53 class mediator	13/293	267/18670	0,000323	0,001159	0,000511	*TP53/AKT1/MDM2/TP63/TP73/BAX/BCL2/DNA2/BAG6/BRIP1/BRCA1/BRCA2/CNOT1*	13
GO:0009167	purine ribonucleoside monophosphate metabolic process	15/293	340/18670	0,000331	0,001185	0,000522	*TP53/STAT3/BAD/HSPA1B/HSPA1A/ADA/ARNT/HIF1A/TGFB1/INSR/INS/IFNG/SLC4A1/IGF1/IL4*	15
GO:0009126	purine nucleoside monophosphate metabolic process	15/293	341/18670	0,000341	0,001221	0,000538	*TP53/STAT3/BAD/HSPA1B/HSPA1A/ADA/ARNT/HIF1A/TGFB1/INSR/INS/IFNG/SLC4A1/IGF1/IL4*	15
GO:0009199	ribonucleoside triphosphate metabolic process	15/293	341/18670	0,000341	0,001221	0,000538	*TP53/STAT3/BAD/HSPA1B/HSPA1A/RAN/ARNT/HIF1A/TGFB1/INSR/INS/IFNG/SLC4A1/IGF1/IL4*	15
GO:0030501	positive regulation of bone mineralization	5/293	39/18670	0,000342	0,001222	0,000538	*ACVR1/BMP2/ADRB2/LTF/TGFB1*	5
GO:1904591	positive regulation of protein import	5/293	39/18670	0,000342	0,001222	0,000538	*RAN/FLNA/TGFB1/IFNG/LEP*	5
GO:2000816	negative regulation of mitotic sister chromatid separation	5/293	39/18670	0,000342	0,001222	0,000538	*LCMT1/APC/BUB1/BUB1B/BUB3*	5
GO:0006939	smooth muscle contraction	8/293	110/18670	0,000343	0,001226	0,00054	*ACTA2/ADA/ADRA2A/ADRB2/AGT/PRKG1/ITGA2/SULF1*	8
GO:0001759	organ induction	4/293	22/18670	0,000348	0,001234	0,000544	*AR/FGFR1/BMP2/FGF1*	4
GO:0032727	positive regulation of interferon-alpha production	4/293	22/18670	0,000348	0,001234	0,000544	*TLR4/CHUK/MMP12/HMGB1*	4
GO:0032928	regulation of superoxide anion generation	4/293	22/18670	0,000348	0,001234	0,000544	*EGFR/GSTP1/AGT/TGFB1*	4
GO:0044321	response to leptin	4/293	22/18670	0,000348	0,001234	0,000544	*STAT3/LEPR/FGB/LEP*	4
GO:0048305	immunoglobulin secretion	4/293	22/18670	0,000348	0,001234	0,000544	*TNF/IL6/RBP4/HLA-E*	4

ID	Description	GeneRatio	BgRatio	pvalue	p.adjust	qvalue	geneID	Count
GO:0051195	negative regulation of cofactor metabolic process	4/293	22/18670	0,000348	0,001234	0,000544	*TP53/STAT3/MMP3/HP*	4
GO:0090312	positive regulation of protein deacetylation	4/293	22/18670	0,000348	0,001234	0,000544	*TP53/VEGFA/TGFB1/IFNG*	4
GO:1901522	positive regulation of transcription from RNA polymerase II promoter involved in cellular response to chemical stimulus	4/293	22/18670	0,000348	0,001234	0,000544	*TP53/BMP2/HIF1A/VEGFA*	4
GO:0046324	regulation of glucose import	6/293	60/18670	0,000349	0,001237	0,000545	*AKT1/TNF/INSR/INS/IGF1/LEP*	6
GO:0046034	ATP metabolic process	14/293	305/18670	0,00035	0,001239	0,000546	*TP53/STAT3/BAD/HSPA1B/HSPA1A/ARNT/HIF1A/TGFB1/INSR/INS/IFNG/SLC4A1/IGF1/IL4*	14
GO:0033135	regulation of peptidyl-serine phosphorylation	9/293	139/18670	0,000353	0,001249	0,00055	*AKT1/EGFR/TNF/IL6/BAX/BCL2/VEGFA/TGFB1/IFNG*	9
GO:0001837	epithelial to mesenchymal transition	9/293	140/18670	0,000372	0,001315	0,000579	*MTOR/ACVR1/TGFBR1/BMP2/IL1B/IL6/HIF1A/TGFB1/COL1A1*	9
GO:1903364	positive regulation of cellular protein catabolic process	9/293	140/18670	0,000372	0,001315	0,000579	*AKT1/MDM2/HSPA1B/HSPA1A/APOE/BAG6/CEBPA/EGF/FMR1*	9
GO:0007405	neuroblast proliferation	6/293	61/18670	0,000382	0,001347	0,000593	*TP53/FGFR1/HIF1A/VEGFA/TGFB1/VEGFC*	6
GO:0048645	animal organ formation	6/293	61/18670	0,000382	0,001347	0,000593	*PIM1/AR/FGFR1/TP63/SULF1/FGF1*	6
GO:2000514	regulation of CD4-positive, alpha-beta T cell activation	6/293	61/18670	0,000382	0,001347	0,000593	*HMGB1/IL12B/IL23R/FOXP3/IFNG/IL4R*	6
GO:0031669	cellular response to nutrient levels	12/293	237/18670	0,000384	0,001353	0,000596	*PIM1/TP53/MDM2/MTOR/PPARG/BCL2/BRIP1/LCN2/COL1A1/EIF2AK2/HFE/LEP*	12
GO:1905819	negative regulation of chromosome separation	5/293	40/18670	0,000386	0,001359	0,000599	*LCMT1/APC/BUB1/BUB1B/BUB3*	5
GO:0008645	hexose transmembrane transport	8/293	112/18670	0,000388	0,001364	0,000601	*AKT1/IL1B/TNF/INSR/INS/HNF1A/IGF1/LEP*	8
GO:1990748	cellular detoxification	8/293	112/18670	0,000388	0,001364	0,000601	*NOS3/GSTP1/TNF/HBE1/APOE/DHFR/HBA1/HP*	8
GO:0050709	negative regulation of protein secretion	9/293	141/18670	0,000392	0,001378	0,000607	*FN1/ADRA2A/IL1B/TNF/APOE/LILRB1/INS/FOXP3/IL10*	9

ID	Description	GeneRatio	BgRatio	pvalue	p.adjust	qvalue	geneID	Count
GO:0046394	carboxylic acid biosynthetic process	18/293	462/18670	0,000392	0,001378	0,000607	*NFKB1/TP53/STAT3/IL1B/PAH/ARNT/HIF1A/BRCA1/CEACAM1/TGFB1/DHFR/EGF/INSR/INS/FOLH1/IFNG/SLC4A1/IGF1*	18
GO:0016053	organic acid biosynthetic process	18/293	463/18670	0,000403	0,001412	0,000622	*NFKB1/TP53/STAT3/IL1B/PAH/ARNT/HIF1A/BRCA1/CEACAM1/TGFB1/DHFR/EGF/INSR/INS/FOLH1/IFNG/SLC4A1/IGF1*	18
GO:0001942	hair follicle development	7/293	86/18670	0,000407	0,001426	0,000628	*EGFR/TNF/TP63/BCL2/NSDHL/FST/FGF7*	7
GO:0034637	cellular carbohydrate biosynthetic process	7/293	86/18670	0,000407	0,001426	0,000628	*AKT1/MTOR/INSR/INS/IGF2/IGF1/LEP*	7
GO:0050871	positive regulation of B cell activation	9/293	142/18670	0,000413	0,001446	0,000637	*TLR4/BAD/CD320/ADA/IL6/BCL2/TGFB1/TFRC/IL4*	9
GO:0006359	regulation of transcription by RNA polymerase III	4/293	23/18670	0,000416	0,00145	0,000639	*MTOR/AR/BRCA1/CEBPA*	4
GO:0032753	positive regulation of interleukin-4 production	4/293	23/18670	0,000416	0,00145	0,000639	*HAVCR2/HLA-E/CEBPB/FOXP3*	4
GO:0036037	CD8-positive, alpha-beta T cell activation	4/293	23/18670	0,000416	0,00145	0,000639	*BCL2/HLA-E/LILRB1/HFE*	4
GO:0051882	mitochondrial depolarization	4/293	23/18670	0,000416	0,00145	0,000639	*SRC/BCL2/IFI6/KDR*	4
GO:0071425	hematopoietic stem cell proliferation	4/293	23/18670	0,000416	0,00145	0,000639	*PIM1/ACE/ETV6/EIF2AK2*	4
GO:0002562	somatic diversification of immune receptors via germline recombination within a single locus	6/293	62/18670	0,000417	0,001451	0,000639	*HMGB1/TGFB1/FOXP3/TFRC/IL4/IL10*	6
GO:0016444	somatic cell DNA recombination	6/293	62/18670	0,000417	0,001451	0,000639	*HMGB1/TGFB1/FOXP3/TFRC/IL4/IL10*	6
GO:0060393	regulation of pathway-restricted SMAD protein phosphorylation	6/293	62/18670	0,000417	0,001451	0,000639	*ACVR1/TGFBR1/BMP2/INHA/TGFB1/HFE*	6
GO:0070265	necrotic cell death	6/293	62/18670	0,000417	0,001451	0,000639	*TP53/TLR4/TNF/BAX/CD14/FASLG*	6
GO:0009165	nucleotide biosynthetic process	16/293	386/18670	0,000421	0,001457	0,000642	*TP53/STAT3/NOS3/ADA/ARNT/HIF1A/TYMS/RRM2/TGFB1/INSR/INS/IFNG/SLC4A1/IGF1/IL4/NOS2*	16
GO:0001660	fever generation	3/293	10/18670	0,000423	0,001457	0,000642	*IL1B/TNF/IL1A*	3
GO:0002291	T cell activation via T cell receptor contact with antigen bound to MHC	3/293	10/18670	0,000423	0,001457	0,000642	*LGALS3/HAVCR2/LILRB1*	3

ID	Description	GeneRatio	BgRatio	pvalue	p.adjust	qvalue	geneID	Count
	molecule on antigen presenting cell							
GO:0033210	leptin-mediated signaling pathway	3/293	10/18670	0,000423	0,001457	0,000642	*STAT3/LEPR/LEP*	3
GO:0033690	positive regulation of osteoblast proliferation	3/293	10/18670	0,000423	0,001457	0,000642	*GATA1/BMP2/LTF*	3
GO:0042536	negative regulation of tumor necrosis factor biosynthetic process	3/293	10/18670	0,000423	0,001457	0,000642	*LILRB1/IL4/IL10*	3
GO:0042756	drinking behavior	3/293	10/18670	0,000423	0,001457	0,000642	*ACE2/AGT/REN*	3
GO:0045945	positive regulation of transcription by RNA polymerase III	3/293	10/18670	0,000423	0,001457	0,000642	*MTOR/AR/CEBPA*	3
GO:0060068	vagina development	3/293	10/18670	0,000423	0,001457	0,000642	*ESR1/RBP4/BAX*	3
GO:0060513	prostatic bud formation	3/293	10/18670	0,000423	0,001457	0,000642	*AR/TP63/SULF1*	3
GO:1902033	regulation of hematopoietic stem cell proliferation	3/293	10/18670	0,000423	0,001457	0,000642	*PIM1/ACE/EIF2AK2*	3
GO:1903799	negative regulation of production of miRNAs involved in gene silencing by miRNA	3/293	10/18670	0,000423	0,001457	0,000642	*TP53/ESR1/TGFB1*	3
GO:1903800	positive regulation of production of miRNAs involved in gene silencing by miRNA	3/293	10/18670	0,000423	0,001457	0,000642	*TP53/EGFR/TGFB1*	3
GO:0006308	DNA catabolic process	5/293	41/18670	0,000434	0,001492	0,000657	*CASP3/IL6/BAX/HMGB1/DICER1*	5
GO:0061028	establishment of endothelial barrier	5/293	41/18670	0,000434	0,001492	0,000657	*TNFRSF1A/IL1B/TNF/PROC/VEGFA*	5
GO:1903053	regulation of extracellular matrix organization	5/293	41/18670	0,000434	0,001492	0,000657	*TNFRSF1A/IL6/AGT/CPB2/TGFB1*	5
GO:0015749	monosaccharide transmembrane transport	8/293	114/18670	0,000437	0,001501	0,000661	*AKT1/IL1B/TNF/INSR/INS/HNF1A/IGF1/LEP*	8
GO:0090092	regulation of transmembrane receptor protein	12/293	241/18670	0,000446	0,001531	0,000675	*TMPRSS6/TP53/HSPA1A/ACVR1/TGFBR1/BMP2/INHA/TGFB1/FBN1/FST/SULF1/HFE*	12

ID	Description	GeneRatio	BgRatio	pvalue	p.adjust	qvalue	geneID	Count
	serine/threonine kinase signaling pathway							
GO:0072522	purine-containing compound biosynthetic process	14/293	313/18670	0,000453	0,001554	0,000685	*TP53/STAT3/NOS3/ADA/ARNT/HIF1A/TGFB1/INSR/INS/IFNG/SLC4A1/IGF1/IL4/NOS2*	14
GO:0034394	protein localization to cell surface	6/293	63/18670	0,000455	0,001559	0,000687	*AKT1/TNF/FLNA/EGF/FGF7/HFE*	6
GO:0046622	positive regulation of organ growth	6/293	63/18670	0,000455	0,001559	0,000687	*PIM1/AKT1/MTOR/FGFR1/HAMP/IGF1*	6
GO:0022404	molting cycle process	7/293	88/18670	0,000468	0,001599	0,000704	*EGFR/TNF/TP63/BCL2/NSDHL/FST/FGF7*	7
GO:0022405	hair cycle process	7/293	88/18670	0,000468	0,001599	0,000704	*EGFR/TNF/TP63/BCL2/NSDHL/FST/FGF7*	7
GO:0045921	positive regulation of exocytosis	7/293	88/18670	0,000468	0,001599	0,000704	*GATA2/FGG/FGB/FGA/CFTR/IFNG/IL4R*	7
GO:0098773	skin epidermis development	7/293	88/18670	0,000468	0,001599	0,000704	*EGFR/TNF/TP63/BCL2/NSDHL/FST/FGF7*	7
GO:1901293	nucleoside phosphate biosynthetic process	16/293	390/18670	0,000471	0,001606	0,000708	*TP53/STAT3/NOS3/ADA/ARNT/HIF1A/TYMS/RRM2/TGFB1/INSR/INS/IFNG/SLC4A1/IGF1/IL4/NOS2*	16
GO:0051961	negative regulation of nervous system development	14/293	315/18670	0,000483	0,001645	0,000725	*TP53/STAT3/TRPC6/THRB/MDM2/IL1B/TNF/IL6/TP73/F2/APOE/MAP2/TGFB1/DICER1*	14
GO:0035303	regulation of dephosphorylation	11/293	209/18670	0,000483	0,001645	0,000725	*SRC/MTOR/LGALS3/IGFBP3/JAK2/BMP2/TNF/ITGA2/TGFB1/EIF2AK2/IFNG*	11
GO:0010613	positive regulation of cardiac muscle hypertrophy	5/293	42/18670	0,000486	0,001655	0,000729	*MTOR/AGT/HAMP/IGF1/PDE5A*	5
GO:0033048	negative regulation of mitotic sister chromatid segregation	5/293	42/18670	0,000486	0,001655	0,000729	*LCMT1/APC/BUB1/BUB1B/BUB3*	5
GO:0034219	carbohydrate transmembrane transport	8/293	116/18670	0,000491	0,00167	0,000736	*AKT1/IL1B/TNF/INSR/INS/HNF1A/IGF1/LEP*	8
GO:0002407	dendritic cell chemotaxis	4/293	24/18670	0,000493	0,001672	0,000737	*CCR5/HMGB1/CXCR1/IL12A*	4
GO:0009110	vitamin biosynthetic process	4/293	24/18670	0,000493	0,001672	0,000737	*NFKB1/IL1B/TNF/IFNG*	4
GO:2000209	regulation of anoikis	4/293	24/18670	0,000493	0,001672	0,000737	*SRC/BCL2/CEACAM6/CEACAM5*	4
GO:0006826	iron ion transport	6/293	64/18670	0,000496	0,00168	0,00074	*HEPH/SLC11A1/IFNG/HAMP/HFE/TFRC*	6
GO:1903672	positive regulation of sprouting angiogenesis	6/293	64/18670	0,000496	0,00168	0,00074	*GATA2/AGTR1/VEGFA/FGF1/KDR/IL10*	6

ID	Description	GeneRatio	BgRatio	pvalue	p.adjust	qvalue	geneID	Count
GO:0009161	ribonucleoside monophosphate metabolic process	15/293	354/18670	0,000505	0,001708	0,000752	*TP53/STAT3/BAD/HSPA1B/HSPA1A/ADA/ARNT/HIF1A/TGFB1/INSR/INS/IFNG/SLC4A1/IGF1/IL4*	15
GO:2000241	regulation of reproductive process	9/293	146/18670	0,000506	0,001711	0,000754	*SRC/AR/ESR1/ADA/TIMP1/SULF1/INSR/IGF1/PDE5A*	9
GO:0010508	positive regulation of autophagy	8/293	117/18670	0,00052	0,001755	0,000773	*BAD/IKBKG/ADRB2/HIF1A/HMGB1/KDR/IFNG/IL4*	8
GO:0051153	regulation of striated muscle cell differentiation	8/293	117/18670	0,00052	0,001755	0,000773	*MTOR/BMP2/BCL2/CEACAM5/TGFB1/HAMP/IGF1/IL4R*	8
GO:0060147	regulation of posttranscriptional gene silencing	8/293	117/18670	0,00052	0,001755	0,000773	*TP53/STAT3/EGFR/ESR1/PPARG/XPO5/TGFB1/FMR1*	8
GO:0060966	regulation of gene silencing by RNA	8/293	117/18670	0,00052	0,001755	0,000773	*TP53/STAT3/EGFR/ESR1/PPARG/XPO5/TGFB1/FMR1*	8
GO:0010950	positive regulation of endopeptidase activity	10/293	178/18670	0,000522	0,001759	0,000775	*STAT3/BAD/PPARG/JAK2/TNF/CASP9/BAX/HMGB1/FASLG/F3*	10
GO:0019359	nicotinamide nucleotide biosynthetic process	9/293	147/18670	0,000532	0,001788	0,000788	*TP53/STAT3/ARNT/HIF1A/INSR/INS/IFNG/SLC4A1/IGF1*	9
GO:0019363	pyridine nucleotide biosynthetic process	9/293	147/18670	0,000532	0,001788	0,000788	*TP53/STAT3/ARNT/HIF1A/INSR/INS/IFNG/SLC4A1/IGF1*	9
GO:0071236	cellular response to antibiotic	9/293	147/18670	0,000532	0,001788	0,000788	*TP53/SRC/TRPC6/MDM2/IL6/AHR/LCN2/CFTR/IL10*	9
GO:0060389	pathway-restricted SMAD protein phosphorylation	6/293	65/18670	0,000539	0,001812	0,000799	*ACVR1/TGFBR1/BMP2/INHA/TGFB1/HFE*	6
GO:0006775	fat-soluble vitamin metabolic process	5/293	43/18670	0,000543	0,001821	0,000803	*NFKB1/FGFR1/IL1B/TNF/IFNG*	5
GO:0008631	intrinsic apoptotic signaling pathway in response to oxidative stress	5/293	43/18670	0,000543	0,001821	0,000803	*AKT1/JAK2/HIF1A/BCL2/INS*	5
GO:0014742	positive regulation of muscle hypertrophy	5/293	43/18670	0,000543	0,001821	0,000803	*MTOR/AGT/HAMP/IGF1/PDE5A*	5
GO:0071364	cellular response to epidermal growth factor stimulus	5/293	43/18670	0,000543	0,001821	0,000803	*AKT1/STAT5B/EGFR/GSTP1/COL1A1*	5
GO:0002836	positive regulation of response to tumor cell	3/293	11/18670	0,000575	0,001912	0,000842	*HRG/IL12B/IL12A*	3
GO:0002839	positive regulation of immune response to tumor cell	3/293	11/18670	0,000575	0,001912	0,000842	*HRG/IL12B/IL12A*	3

ID	Description	GeneRatio	BgRatio	pvalue	p.adjust	qvalue	geneID	Count
GO:0016264	gap junction assembly	3/293	11/18670	0,000575	0,001912	0,000842	*ACE/ACE2/AGT*	3
GO:0033860	regulation of NAD(P)H oxidase activity	3/293	11/18670	0,000575	0,001912	0,000842	*AGTR1/AGT/INS*	3
GO:0045899	positive regulation of RNA polymerase II transcriptional preinitiation complex assembly	3/293	11/18670	0,000575	0,001912	0,000842	*TP53/ESR1/CREB1*	3
GO:0051024	positive regulation of immunoglobulin secretion	3/293	11/18670	0,000575	0,001912	0,000842	*IL6/RBP4/HLA-E*	3
GO:0051974	negative regulation of telomerase activity	3/293	11/18670	0,000575	0,001912	0,000842	*TP53/SRC/PPARG*	3
GO:0070278	extracellular matrix constituent secretion	3/293	11/18670	0,000575	0,001912	0,000842	*TNFRSF1A/AGT/CPB2*	3
GO:0070757	interleukin-35-mediated signaling pathway	3/293	11/18670	0,000575	0,001912	0,000842	*STAT3/JAK2/IL12A*	3
GO:1903011	negative regulation of bone development	3/293	11/18670	0,000575	0,001912	0,000842	*LTF/LILRB1/FBN1*	3
GO:2001204	regulation of osteoclast development	3/293	11/18670	0,000575	0,001912	0,000842	*LTF/LILRB1/FBN1*	3
GO:0000729	DNA double-strand break processing	4/293	25/18670	0,00058	0,001923	0,000847	*UBE2N/DNA2/BRIP1/BRCA1*	4
GO:0032461	positive regulation of protein oligomerization	4/293	25/18670	0,00058	0,001923	0,000847	*TP53/BAX/MMP3/MMP1*	4
GO:0034110	regulation of homotypic cell-cell adhesion	4/293	25/18670	0,00058	0,001923	0,000847	*FGG/PRKG1/CD9/CEACAM1*	4
GO:0001952	regulation of cell-matrix adhesion	8/293	119/18670	0,000582	0,001931	0,000851	*SRC/HRG/BCL2/MMP12/SERPINE1/CEACAM6/VEGFA/KDR*	8
GO:0046626	regulation of insulin receptor signaling pathway	6/293	66/18670	0,000585	0,001937	0,000854	*SRC/IL1B/AGT/INS/IGF2/LEP*	6
GO:0051926	negative regulation of calcium ion transport	6/293	66/18670	0,000585	0,001937	0,000854	*NOS3/ADRA2A/BCL2/LILRB1/TGFB1/FMR1*	6
GO:1905207	regulation of cardiocyte differentiation	6/293	66/18670	0,000585	0,001937	0,000854	*EGFR/MTOR/BMP2/TGFB1/HAMP/IGF1*	6

ID	Description	GeneRatio	BgRatio	pvalue	p.adjust	qvalue	geneID	Count
GO:2001056	positive regulation of cysteine-type endopeptidase activity	9/293	149/18670	0,000586	0,001939	0,000854	*BAD/PPARG/JAK2/TNF/CASP9/BAX/HMGB1/FASLG/F3*	9
GO:0007254	JNK cascade	11/293	214/18670	0,000588	0,001944	0,000857	*AKT1/TLR4/EGFR/IKBKG/IGF1R/GSTP1/IL1B/TNF/UBE2N/HMGB1/IL1RN*	11
GO:0071560	cellular response to transforming growth factor beta stimulus	12/293	249/18670	0,000597	0,00197	0,000868	*TP53/SRC/NR3C1/HSPA1A/ACVR1/TGFBR1/CREB1/FSHB/TGFB1/COL1A1/FBN1/COL1A2*	12
GO:0015980	energy derivation by oxidation of organic compounds	13/293	285/18670	0,000599	0,001977	0,000871	*TP53/AKT1/MTOR/LEPR/HIF1A/INSR/INS/IGF2/IFNG/IGF1/IL4/LEP/NOS2*	13
GO:0003197	endocardial cushion development	5/293	44/18670	0,000605	0,001989	0,000876	*MDM2/NOS3/ACVR1/TGFBR1/BMP2*	5
GO:0032965	regulation of collagen biosynthetic process	5/293	44/18670	0,000605	0,001989	0,000876	*PPARG/IL6/F2/ITGA2/TGFB1*	5
GO:0033046	negative regulation of sister chromatid segregation	5/293	44/18670	0,000605	0,001989	0,000876	*LCMT1/APC/BUB1/BUB1B/BUB3*	5
GO:0042987	amyloid precursor protein catabolic process	5/293	44/18670	0,000605	0,001989	0,000876	*CASP3/TNF/APOE/IFNG/IGF1*	5
GO:0070266	necroptotic process	5/293	44/18670	0,000605	0,001989	0,000876	*TP53/TLR4/TNF/CD14/FASLG*	5
GO:0090311	regulation of protein deacetylation	5/293	44/18670	0,000605	0,001989	0,000876	*TP53/VEGFA/TGFB1/FOXP3/IFNG*	5
GO:2000117	negative regulation of cysteine-type endopeptidase activity	7/293	92/18670	0,000613	0,002012	0,000887	*SRC/AKT1/MDM2/MMP9/LTF/VEGFA/IFI6*	7
GO:0072525	pyridine-containing compound biosynthetic process	9/293	150/18670	0,000615	0,002018	0,000889	*TP53/STAT3/ARNT/HIF1A/INSR/INS/IFNG/SLC4A1/IGF1*	9
GO:0003014	renal system process	8/293	120/18670	0,000616	0,002018	0,000889	*AGTR1/AGT/REN/SGK1/BCL2/SULF1/SLC4A1/HNF1A*	8
GO:1903052	positive regulation of proteolysis involved in cellular protein catabolic process	8/293	120/18670	0,000616	0,002018	0,000889	*AKT1/MDM2/HSPA1B/HSPA1A/BAG6/CEBPA/EGF/FMR1*	8
GO:0001654	eye development	15/293	362/18670	0,000635	0,00208	0,000917	*STAT3/THRB/EGFR/TGFBR1/RBP4/HIF1A/BAX/BCL2/VEGFA/TGFB1/FBN1/COL5A1/COL5A2/DCX/FASLG*	15
GO:0007589	body fluid secretion	7/293	93/18670	0,000654	0,002138	0,000942	*ADA/SERPINC1/PRLR/HIF1A/VEGFA/CREB1/PRL*	7
GO:0072080	nephron tubule development	7/293	93/18670	0,000654	0,002138	0,000942	*BMP2/AGT/BCL2/VEGFA/TGFB1/FGF1/PGF*	7

ID	Description	GeneRatio	BgRatio	pvalue	p.adjust	qvalue	geneID	Count
GO:0034248	regulation of cellular amide metabolic process	18/293	483/18670	0,00066	0,002156	0,00095	*AKT1/STAT3/CASP3/MTOR/TNFRSF1A/PA2G4/TNF/IL6/TYMS/APOE/ITGA2/CNBP/CNOT1/EIF2AK2/DHFR/FMR1/IFNG/IGF1*	18
GO:0070838	divalent metal ion transport	18/293	483/18670	0,00066	0,002156	0,00095	*TRPC6/NOS3/LGALS3/ADRA2A/CCR5/AGT/F2/IL16/BAX/BCL2/SLC11A1/LILRB1/TGFB1/EGF/FASLG/FMR1/IFNG/TRPC1*	18
GO:0031018	endocrine pancreas development	5/293	45/18670	0,000672	0,002193	0,000966	*AKT1/BAD/IL6/HNF4A/IL6R*	5
GO:0051985	negative regulation of chromosome segregation	5/293	45/18670	0,000672	0,002193	0,000966	*LCMT1/APC/BUB1/BUB1B/BUB3*	5
GO:0060740	prostate gland epithelium morphogenesis	4/293	26/18670	0,000677	0,002203	0,000971	*AR/ESR1/TP63/SULF1*	4
GO:1905048	regulation of metallopeptidase activity	4/293	26/18670	0,000677	0,002203	0,000971	*STAT3/TIMP1/TIMP3/TIMP2*	4
GO:0016064	immunoglobulin mediated immune response	11/293	218/18670	0,000686	0,002233	0,000984	*TNF/MBL2/HLA-E/TGFB1/FOXP3/TFRC/HLA-DQB1/IL4R/IL4/LTA/IL10*	11
GO:1901990	regulation of mitotic cell cycle phase transition	17/293	444/18670	0,000688	0,002238	0,000986	*TP53/AIF1/AKT1/EGFR/MDM2/LCMT1/BAX/BCL2/APC/BUB1/BUB1B/BUB3/UBD/BRCA1/TUBA1A/CNOT1/TGFB1*	17
GO:0060993	kidney morphogenesis	7/293	94/18670	0,000697	0,002266	0,000998	*BMP2/AGT/BCL2/VEGFA/TGFB1/FGF1/PGF*	7
GO:0034250	positive regulation of cellular amide metabolic process	9/293	153/18670	0,00071	0,002303	0,001015	*CASP3/MTOR/TNFRSF1A/TNF/IL6/ITGA2/CNBP/FMR1/IFNG*	9
GO:0090316	positive regulation of intracellular protein transport	9/293	153/18670	0,00071	0,002303	0,001015	*HSPA1L/TP53/MDM2/RAN/IL1B/FLNA/TGFB1/IFNG/LEP*	9
GO:0150063	visual system development	15/293	366/18670	0,000711	0,002305	0,001016	*STAT3/THRB/EGFR/TGFBR1/RBP4/HIF1A/BAX/BCL2/VEGFA/TGFB1/FBN1/COL5A1/COL5A2/DCX/FASLG*	15
GO:0071559	response to transforming growth factor beta	12/293	255/18670	0,000735	0,002383	0,00105	*TP53/SRC/NR3C1/HSPA1A/ACVR1/TGFBR1/CREB1/FSHB/TGFB1/COL1A1/FBN1/COL1A2*	12
GO:0061326	renal tubule development	7/293	95/18670	0,000743	0,002405	0,00106	*BMP2/AGT/BCL2/VEGFA/TGFB1/FGF1/PGF*	7
GO:0006090	pyruvate metabolic process	9/293	154/18670	0,000743	0,002406	0,00106	*TP53/STAT3/ARNT/HIF1A/INSR/INS/IFNG/SLC4A1/IGF1*	9
GO:0035196	production of miRNAs involved in gene silencing by miRNA	5/293	46/18670	0,000745	0,002409	0,001061	*TP53/EGFR/ESR1/TGFB1/DICER1*	5

ID	Description	GeneRatio	BgRatio	pvalue	p.adjust	qvalue	geneID	Count
GO:0002002	regulation of angiotensin levels in blood	3/293	12/18670	0,000758	0,002437	0,001074	*ACE/ACE2/REN*	3
GO:0002003	angiotensin maturation	3/293	12/18670	0,000758	0,002437	0,001074	*ACE/ACE2/REN*	3
GO:0002674	negative regulation of acute inflammatory response	3/293	12/18670	0,000758	0,002437	0,001074	*PPARG/GSTP1/INS*	3
GO:0002863	positive regulation of inflammatory response to antigenic stimulus	3/293	12/18670	0,000758	0,002437	0,001074	*TNF/HLA-E/LTA*	3
GO:0006828	manganese ion transport	3/293	12/18670	0,000758	0,002437	0,001074	*TRPC6/SLC11A1/TRPC1*	3
GO:0045080	positive regulation of chemokine biosynthetic process	3/293	12/18670	0,000758	0,002437	0,001074	*IL1B/TNF/IFNG*	3
GO:0051549	positive regulation of keratinocyte migration	3/293	12/18670	0,000758	0,002437	0,001074	*MTOR/MMP9/FGF7*	3
GO:0070243	regulation of thymocyte apoptotic process	3/293	12/18670	0,000758	0,002437	0,001074	*TP53/ADA/HIF1A*	3
GO:0032673	regulation of interleukin-4 production	4/293	27/18670	0,000784	0,002515	0,001108	*HAVCR2/HLA-E/CEBPB/FOXP3*	4
GO:0035902	response to immobilization stress	4/293	27/18670	0,000784	0,002515	0,001108	*MDM2/PPARG/REN/TGFB1*	4
GO:0036336	dendritic cell migration	4/293	27/18670	0,000784	0,002515	0,001108	*CCR5/HMGB1/CXCR1/IL12A*	4
GO:0060259	regulation of feeding behavior	4/293	27/18670	0,000784	0,002515	0,001108	*STAT3/MTOR/LEPR/INS*	4
GO:2000144	positive regulation of DNA-templated transcription, initiation	4/293	27/18670	0,000784	0,002515	0,001108	*TP53/ESR1/CREB1/HNF1A*	4
GO:0019217	regulation of fatty acid metabolic process	7/293	96/18670	0,000791	0,002532	0,001116	*AKT1/MTOR/PPARG/IL1B/BRCA1/CEACAM1/INS*	7
GO:0034121	regulation of toll-like receptor signaling pathway	6/293	70/18670	0,000802	0,002565	0,00113	*TLR4/ESR1/HMGB1/CD14/LTF/TLR1*	6
GO:0061515	myeloid cell development	6/293	70/18670	0,000802	0,002565	0,00113	*SRC/GATA1/LTF/LILRB1/FBN1/SLC4A1*	6

ID	Description	GeneRatio	BgRatio	pvalue	p.adjust	qvalue	geneID	Count
GO:0007517	muscle organ development	16/293	410/18670	0,000805	0,002572	0,001133	*PIM1/ACTA1/MTOR/NR2F2/FGFR1/TGFBR1/BMP2/TP73/RBP4/BCL2/CREB1/TGFB1/COL6A3/NEB/HAMP/IGF1*	16
GO:0045471	response to ethanol	8/293	125/18670	0,000806	0,002575	0,001135	*STAT3/BAD/GSTP1/RBP4/TYMS/CD14/HAMP/LEP*	8
GO:0048880	sensory system development	15/293	371/18670	0,000816	0,002605	0,001148	*STAT3/THRB/EGFR/TGFBR1/RBP4/HIF1A/BAX/BCL2/VEGFA/TGFB1/FBN1/COL5A1/COL5A2/DCX/FASLG*	15
GO:0002204	somatic recombination of immunoglobulin genes involved in immune response	5/293	47/18670	0,000823	0,002613	0,001151	*TGFB1/FOXP3/TFRC/IL4/IL10*	5
GO:0002208	somatic diversification of immunoglobulins involved in immune response	5/293	47/18670	0,000823	0,002613	0,001151	*TGFB1/FOXP3/TFRC/IL4/IL10*	5
GO:0031952	regulation of protein autophosphorylation	5/293	47/18670	0,000823	0,002613	0,001151	*ACE/SRC/VEGFA/INS/VEGFC*	5
GO:0035722	interleukin-12-mediated signaling pathway	5/293	47/18670	0,000823	0,002613	0,001151	*JAK2/IL12B/IFNG/IL12A/IL10*	5
GO:0045190	isotype switching	5/293	47/18670	0,000823	0,002613	0,001151	*TGFB1/FOXP3/TFRC/IL4/IL10*	5
GO:0048806	genitalia development	5/293	47/18670	0,000823	0,002613	0,001151	*AR/ESR1/TP63/RBP4/BAX*	5
GO:0070849	response to epidermal growth factor	5/293	47/18670	0,000823	0,002613	0,001151	*AKT1/STAT5B/EGFR/GSTP1/COL1A1*	5
GO:2000107	negative regulation of leukocyte apoptotic process	5/293	47/18670	0,000823	0,002613	0,001151	*ADA/CCR5/HIF1A/LILRB1/TSC22D3*	5
GO:0072503	cellular divalent inorganic cation homeostasis	18/293	493/18670	0,000836	0,002651	0,001168	*TRPC6/GATA2/ESR1/JAK2/AGTR1/CCR5/AGT/F2/BAX/BCL2/APOE/HMGB1/C5AR1/SLC11A1/TGFB1/CXCR1/FASLG/TRPC1*	18
GO:0015908	fatty acid transport	7/293	97/18670	0,000841	0,002666	0,001175	*ACE/AKT1/PPARG/IL1B/APOE/LEP/NOS2*	7
GO:0018107	peptidyl-threonine phosphorylation	8/293	126/18670	0,000849	0,002689	0,001185	*AKT1/TRPC6/MTOR/ACVR1/TGFBR1/BCL2/TGFB1/EGF*	8
GO:0090101	negative regulation of transmembrane receptor protein serine/threonine kinase signaling pathway	8/293	126/18670	0,000849	0,002689	0,001185	*TMPRSS6/TP53/HSPA1A/ACVR1/TGFBR1/TGFB1/FBN1/FST*	8
GO:0001764	neuron migration	9/293	157/18670	0,000853	0,0027	0,00119	*STAT3/GATA2/NR2F2/FGFR1/FLNA/PRKG1/BAX/VEGFA/DCX*	9

ID	Description	GeneRatio	BgRatio	pvalue	p.adjust	qvalue	geneID	Count
GO:0033692	cellular polysaccharide biosynthetic process	6/293	71/18670	0,000864	0,002734	0,001205	*AKT1/MTOR/INSR/INS/IGF2/IGF1*	6
GO:0072659	protein localization to plasma membrane	12/293	260/18670	0,000871	0,002753	0,001213	*AKT1/EGFR/AR/TNFRSF1A/LGALS3/TNF/FLNA/BCL2L1/TGFB1/INS/IFNG/SLC4A1*	12
GO:0019886	antigen processing and presentation of exogenous peptide antigen via MHC class II	7/293	98/18670	0,000894	0,002822	0,001244	*HLA-DQA1/HLA-DRB5/HLA-DRA/HLA-DQB2/HLA-DRB1/HLA-DQB1/HLA-DQA2*	7
GO:0033598	mammary gland epithelial cell proliferation	4/293	28/18670	0,000904	0,002844	0,001253	*ESR1/BAX/BRCA2/CEBPB*	4
GO:0034368	protein-lipid complex remodeling	4/293	28/18670	0,000904	0,002844	0,001253	*AGTR1/AGT/APOB/APOE*	4
GO:0034369	plasma lipoprotein particle remodeling	4/293	28/18670	0,000904	0,002844	0,001253	*AGTR1/AGT/APOB/APOE*	4
GO:0051123	RNA polymerase II preinitiation complex assembly	4/293	28/18670	0,000904	0,002844	0,001253	*TP53/ESR1/HMGB1/CREB1*	4
GO:0060512	prostate gland morphogenesis	4/293	28/18670	0,000904	0,002844	0,001253	*AR/ESR1/TP63/SULF1*	4
GO:1902932	positive regulation of alcohol biosynthetic process	4/293	28/18670	0,000904	0,002844	0,001253	*IL1B/TNF/FGF1/IFNG*	4
GO:0045912	negative regulation of carbohydrate metabolic process	5/293	48/18670	0,000907	0,002846	0,001254	*TP53/STAT3/LEPR/TGFB1/INS*	5
GO:0055023	positive regulation of cardiac muscle tissue growth	5/293	48/18670	0,000907	0,002846	0,001254	*PIM1/MTOR/FGFR1/HAMP/IGF1*	5
GO:0101023	vascular endothelial cell proliferation	5/293	48/18670	0,000907	0,002846	0,001254	*STAT3/FGFR1/PPARG/HMGB1/FLT1*	5
GO:1905562	regulation of vascular endothelial cell proliferation	5/293	48/18670	0,000907	0,002846	0,001254	*STAT3/FGFR1/PPARG/HMGB1/FLT1*	5
GO:0034404	nucleobase-containing small molecule biosynthetic process	11/293	226/18670	0,000922	0,002892	0,001274	*TP53/STAT3/ADA/ARNT/HIF1A/INSR/INS/IFNG/SLC4A1/IGF1/PDE5A*	11
GO:0007281	germ cell development	12/293	262/18670	0,000931	0,002913	0,001284	*SRC/AKT1/MTOR/ACVR1/BAX/BCL2/BCL2L1/BRIP1/BRCA2/CFTR/IGF1/PDE5A*	12
GO:0051155	positive regulation of striated muscle cell differentiation	6/293	72/18670	0,000931	0,002913	0,001284	*MTOR/BCL2/TGFB1/HAMP/IGF1/IL4R*	6

ID	Description	GeneRatio	BgRatio	pvalue	p.adjust	qvalue	geneID	Count
GO:1903747	regulation of establishment of protein localization to mitochondrion	6/293	72/18670	0,000931	0,002913	0,001284	*HSPA1L/TP53/BAD/TP63/TP73/BCL2*	6
GO:0006874	cellular calcium ion homeostasis	17/293	458/18670	0,000968	0,003023	0,001332	*TRPC6/GATA2/ESR1/JAK2/AGTR1/CCR5/AGT/F2/BAX/BCL2/APOE/HMGB1/C5AR1/TGFB1/CXCR1/FASLG/TRPC1*	17
GO:0007171	activation of transmembrane receptor protein tyrosine kinase activity	3/293	13/18670	0,000974	0,003023	0,001332	*ADRB2/PRLR/EGF*	3
GO:0010870	positive regulation of receptor biosynthetic process	3/293	13/18670	0,000974	0,003023	0,001332	*JAK2/HIF1A/IFNG*	3
GO:0010872	regulation of cholesterol esterification	3/293	13/18670	0,000974	0,003023	0,001332	*AGTR1/AGT/APOE*	3
GO:0032725	positive regulation of granulocyte macrophage colony-stimulating factor production	3/293	13/18670	0,000974	0,003023	0,001332	*IL1B/IL12B/IL23R*	3
GO:0033147	negative regulation of intracellular estrogen receptor signaling pathway	3/293	13/18670	0,000974	0,003023	0,001332	*TP63/BRCA1/CNOT1*	3
GO:0060100	positive regulation of phagocytosis, engulfment	3/293	13/18670	0,000974	0,003023	0,001332	*GATA2/PPARG/ITGA2*	3
GO:0060312	regulation of blood vessel remodeling	3/293	13/18670	0,000974	0,003023	0,001332	*HRG/CEACAM1/TGFB1*	3
GO:0060601	lateral sprouting from an epithelium	3/293	13/18670	0,000974	0,003023	0,001332	*AR/TP63/SULF1*	3
GO:0070208	protein heterotrimerization	3/293	13/18670	0,000974	0,003023	0,001332	*COL1A1/COL6A1/COL1A2*	3
GO:0070431	nucleotide-binding oligomerization domain containing 2 signaling pathway	3/293	13/18670	0,000974	0,003023	0,001332	*TLR4/HSPA1B/HSPA1A*	3
GO:1905155	positive regulation of membrane invagination	3/293	13/18670	0,000974	0,003023	0,001332	*GATA2/PPARG/ITGA2*	3
GO:0003206	cardiac chamber morphogenesis	8/293	129/18670	0,00099	0,003071	0,001353	*TP53/MDM2/NOS3/ACVR1/TGFBR1/RBP4/HIF1A/TGFB1*	8

ID	Description	GeneRatio	BgRatio	pvalue	p.adjust	qvalue	geneID	Count
GO:0010712	regulation of collagen metabolic process	5/293	49/18670	0,000997	0,00309	0,001362	*PPARG/IL6/F2/ITGA2/TGFB1*	5
GO:0071349	cellular response to interleukin-12	5/293	49/18670	0,000997	0,00309	0,001362	*JAK2/IL12B/IFNG/IL12A/IL10*	5
GO:0043627	response to estrogen	6/293	73/18670	0,001001	0,0031	0,001366	*MDM2/ESR1/PPARG/F7/BRCA1/IL4R*	6
GO:0030301	cholesterol transport	7/293	100/18670	0,001007	0,003116	0,001373	*NFKB1/PPARG/APOB/APOE/CFTR/EGF/LEP*	7
GO:0031063	regulation of histone deacetylation	4/293	29/18670	0,001036	0,00319	0,001406	*TP53/VEGFA/TGFB1/FOXP3*	4
GO:0034367	protein-containing complex remodeling	4/293	29/18670	0,001036	0,00319	0,001406	*AGTR1/AGT/APOB/APOE*	4
GO:0035666	TRIF-dependent toll-like receptor signaling pathway	4/293	29/18670	0,001036	0,00319	0,001406	*TLR4/CHUK/IKBKG/CD14*	4
GO:0044788	modulation by host of viral process	4/293	29/18670	0,001036	0,00319	0,001406	*APOE/LTF/FMR1/IGF2R*	4
GO:0070723	response to cholesterol	4/293	29/18670	0,001036	0,00319	0,001406	*TGFBR1/CCR5/F7/TGFB1*	4
GO:0072606	interleukin-8 secretion	4/293	29/18670	0,001036	0,00319	0,001406	*CD14/LEP/NOS2/TLR1*	4
GO:2000727	positive regulation of cardiac muscle cell differentiation	4/293	29/18670	0,001036	0,00319	0,001406	*MTOR/TGFB1/HAMP/IGF1*	4
GO:0002495	antigen processing and presentation of peptide antigen via MHC class II	7/293	101/18670	0,001068	0,003284	0,001447	*HLA-DQA1/HLA-DRB5/HLA-DRA/HLA-DQB2/HLA-DRB1/HLA-DQB1/HLA-DQA2*	7
GO:0032611	interleukin-1 beta production	7/293	101/18670	0,001068	0,003284	0,001447	*TLR4/JAK2/GSTP1/IL1B/HMGB1/IFNG/IGF1*	7
GO:0031670	cellular response to nutrient	6/293	74/18670	0,001075	0,003303	0,001456	*PIM1/MDM2/PPARG/BRIP1/COL1A1/LEP*	6
GO:1900076	regulation of cellular response to insulin stimulus	6/293	74/18670	0,001075	0,003303	0,001456	*SRC/IL1B/AGT/INS/IGF2/LEP*	6
GO:0030183	B cell differentiation	8/293	131/18670	0,001094	0,003346	0,001474	*TP53/BAD/ADA/BAX/BCL2/INHA/IL4/IL10*	8
GO:0098754	detoxification	8/293	131/18670	0,001094	0,003346	0,001474	*NOS3/GSTP1/TNF/HBE1/APOE/DHFR/HBA1/HP*	8
GO:0001961	positive regulation of cytokine-mediated signaling pathway	5/293	50/18670	0,001094	0,003346	0,001474	*HSPA1B/HSPA1A/IL1R1/HIF1A/MMP12*	5
GO:0045540	regulation of cholesterol biosynthetic process	5/293	50/18670	0,001094	0,003346	0,001474	*RAN/APOB/APOE/DHCR7/FGF1*	5

ID	Description	GeneRatio	BgRatio	pvalue	p.adjust	qvalue	geneID	Count
GO:0070671	response to interleukin-12	5/293	50/18670	0,001094	0,003346	0,001474	*JAK2/IL12B/IFNG/IL12A/IL10*	5
GO:0106118	regulation of sterol biosynthetic process	5/293	50/18670	0,001094	0,003346	0,001474	*RAN/APOB/APOE/DHCR7/FGF1*	5
GO:2000725	regulation of cardiac muscle cell differentiation	5/293	50/18670	0,001094	0,003346	0,001474	*MTOR/BMP2/TGFB1/HAMP/IGF1*	5
GO:0032386	regulation of intracellular transport	16/293	423/18670	0,001116	0,003411	0,001503	*HSPA1L/TP53/SRC/MDM2/GATA2/RAN/IL1B/FLNA/SPAG5/MAP2/XPO5/TGFB1/FMR1/IFNG/IL4R/LEP*	16
GO:0031346	positive regulation of cell projection organization	15/293	383/18670	0,001123	0,00343	0,001511	*SRC/MTOR/FGFR1/FN1/L1CAM/TGFBR1/AGT/APC/APOE/ITGA2/LCN2/VEGFA/CRTC1/INS/FMR1*	15
GO:0002504	antigen processing and presentation of peptide or polysaccharide antigen via MHC class II	7/293	102/18670	0,001131	0,003453	0,001521	*HLA-DQA1/HLA-DRB5/HLA-DRA/HLA-DQB2/HLA-DRB1/HLA-DQB1/HLA-DQA2*	7
GO:0002200	somatic diversification of immune receptors	6/293	75/18670	0,001154	0,003515	0,001549	*HMGB1/TGFB1/FOXP3/TFRC/IL4/IL10*	6
GO:0010611	regulation of cardiac muscle hypertrophy	6/293	75/18670	0,001154	0,003515	0,001549	*MTOR/TNFRSF1A/AGT/HAMP/IGF1/PDE5A*	6
GO:1903201	regulation of oxidative stress-induced cell death	6/293	75/18670	0,001154	0,003515	0,001549	*AKT1/TLR4/HIF1A/MMP3/INS/IL10*	6
GO:0010586	miRNA metabolic process	4/293	30/18670	0,001181	0,003582	0,001579	*NFKB1/RAN/XPO5/DICER1*	4
GO:0035066	positive regulation of histone acetylation	4/293	30/18670	0,001181	0,003582	0,001579	*IL1B/BRCA1/TGFB1/FOXP3*	4
GO:0061082	myeloid leukocyte cytokine production	4/293	30/18670	0,001181	0,003582	0,001579	*TLR4/HLA-G/LILRB1/TGFB1*	4
GO:0070168	negative regulation of biomineral tissue development	4/293	30/18670	0,001181	0,003582	0,001579	*NOS3/GATA1/HIF1A/TGFB1*	4
GO:0071549	cellular response to dexamethasone stimulus	4/293	30/18670	0,001181	0,003582	0,001579	*NR3C1/EGFR/CASP9/TGFB1*	4
GO:0090200	positive regulation of release of cytochrome c from mitochondria	4/293	30/18670	0,001181	0,003582	0,001579	*TP53/BAD/BAX/MMP9*	4
GO:0030071	regulation of mitotic metaphase/anaphase transition	5/293	51/18670	0,001198	0,003618	0,001594	*LCMT1/APC/BUB1/BUB1B/BUB3*	5

ID	Description	GeneRatio	BgRatio	pvalue	p.adjust	qvalue	geneID	Count
GO:0031050	dsRNA processing	5/293	51/18670	0,001198	0,003618	0,001594	*TP53/EGFR/ESR1/TGFB1/DICER1*	5
GO:0070918	production of small RNA involved in gene silencing by RNA	5/293	51/18670	0,001198	0,003618	0,001594	*TP53/EGFR/ESR1/TGFB1/DICER1*	5
GO:0032091	negative regulation of protein binding	7/293	103/18670	0,001198	0,003618	0,001594	*ACE/AKT1/ADRB2/BAX/MAP2/HFE/IL10*	7
GO:0034766	negative regulation of ion transmembrane transport	7/293	103/18670	0,001198	0,003618	0,001594	*AKT1/MTOR/ADRA2A/MMP9/TGFB1/FMR1/HAMP*	7
GO:0044264	cellular polysaccharide metabolic process	7/293	103/18670	0,001198	0,003618	0,001594	*AKT1/MTOR/LEPR/INSR/INS/IGF2/IGF1*	7
GO:0048259	regulation of receptor-mediated endocytosis	7/293	103/18670	0,001198	0,003618	0,001594	*ITGB3/SERPINE1/VEGFA/EGF/FMR1/HFE/IL4*	7
GO:0010755	regulation of plasminogen activation	3/293	14/18670	0,001225	0,003684	0,001623	*CPB2/F12/SERPINE1*	3
GO:0032310	prostaglandin secretion	3/293	14/18670	0,001225	0,003684	0,001623	*IL1B/LEP/NOS2*	3
GO:0035112	genitalia morphogenesis	3/293	14/18670	0,001225	0,003684	0,001623	*AR/TP63/RBP4*	3
GO:0051547	regulation of keratinocyte migration	3/293	14/18670	0,001225	0,003684	0,001623	*MTOR/MMP9/FGF7*	3
GO:0051712	positive regulation of killing of cells of other organism	3/293	14/18670	0,001225	0,003684	0,001623	*BAD/IFNG/NOS2*	3
GO:0061051	positive regulation of cell growth involved in cardiac muscle cell development	3/293	14/18670	0,001225	0,003684	0,001623	*MTOR/HAMP/IGF1*	3
GO:1905049	negative regulation of metallopeptidase activity	3/293	14/18670	0,001225	0,003684	0,001623	*TIMP1/TIMP3/TIMP2*	3
GO:0007179	transforming growth factor beta receptor signaling pathway	10/293	199/18670	0,001229	0,003693	0,001627	*TP53/SRC/HSPA1A/ACVR1/TGFBR1/CREB1/FSHB/TGFB1/FBN1/COL1A2*	10
GO:0002244	hematopoietic progenitor cell differentiation	9/293	166/18670	0,001264	0,003791	0,001671	*ACE/TP53/GATA2/GATA1/TP73/BCL2/TGFB1/FST/EIF2AK2*	9
GO:0090288	negative regulation of cellular response	9/293	166/18670	0,001264	0,003791	0,001671	*TMPRSS6/TP53/HSPA1A/TGFBR1/AGT/HRG/TGFB1/FBN1/SULF1*	9

ID	Description	GeneRatio	BgRatio	pvalue	p.adjust	qvalue	geneID	Count
	to growth factor stimulus							
GO:0006469	negative regulation of protein kinase activity	11/293	235/18670	0,001265	0,003791	0,001671	*AKT1/CASP3/NR2F2/IGF1R/GSTP1/IL1B/APC/APOE/CEACAM1/CEBPA/IFNG*	11
GO:0006766	vitamin metabolic process	8/293	134/18670	0,001265	0,003791	0,001671	*NFKB1/FGFR1/CD320/TCN2/IL1B/TNF/DHFR/IFNG*	8
GO:0018210	peptidyl-threonine modification	8/293	134/18670	0,001265	0,003791	0,001671	*AKT1/TRPC6/MTOR/ACVR1/TGFBR1/BCL2/TGFB1/EGF*	8
GO:0090100	positive regulation of transmembrane receptor protein serine/threonine kinase signaling pathway	7/293	104/18670	0,001267	0,003792	0,001671	*ACVR1/TGFBR1/BMP2/INHA/TGFB1/SULF1/HFE*	7
GO:1901800	positive regulation of proteasomal protein catabolic process	7/293	104/18670	0,001267	0,003792	0,001671	*AKT1/MDM2/HSPA1B/HSPA1A/BAG6/CEBPA/FMR1*	7
GO:0009620	response to fungus	5/293	52/18670	0,001308	0,0039	0,001719	*TLR4/HRG/LTF/TGFB1/HAMP*	5
GO:0016447	somatic recombination of immunoglobulin gene segments	5/293	52/18670	0,001308	0,0039	0,001719	*TGFB1/FOXP3/TFRC/IL4/IL10*	5
GO:0045839	negative regulation of mitotic nuclear division	5/293	52/18670	0,001308	0,0039	0,001719	*LCMT1/APC/BUB1/BUB1B/BUB3*	5
GO:0060421	positive regulation of heart growth	5/293	52/18670	0,001308	0,0039	0,001719	*PIM1/MTOR/FGFR1/HAMP/IGF1*	5
GO:1900024	regulation of substrate adhesion-dependent cell spreading	5/293	52/18670	0,001308	0,0039	0,001719	*FGG/FLNA/FGB/FGA/FBLN1*	5
GO:2000179	positive regulation of neural precursor cell proliferation	5/293	52/18670	0,001308	0,0039	0,001719	*HIF1A/FLNA/VEGFA/EGF/VEGFC*	5
GO:0055074	calcium ion homeostasis	17/293	471/18670	0,00131	0,003904	0,00172	*TRPC6/GATA2/ESR1/JAK2/AGTR1/CCR5/AGT/F2/BAX/BCL2/APOE/HMGB1/C5AR1/TGFB1/CXCR1/FASLG/TRPC1*	17
GO:0032481	positive regulation of type I interferon production	6/293	77/18670	0,001323	0,003935	0,001734	*NFKB1/TLR4/CHUK/MMP12/HMGB1/CD14*	6
GO:0048678	response to axon injury	6/293	77/18670	0,001323	0,003935	0,001734	*AIF1/JAK2/FLRT3/BAX/BCL2/DHFR*	6
GO:0051851	modification by host of symbiont	6/293	77/18670	0,001323	0,003935	0,001734	*F2/MBL2/APOE/LTF/FMR1/IGF2R*	6

ID	Description	GeneRatio	BgRatio	pvalue	p.adjust	qvalue	geneID	Count
	morphology or physiology							
GO:0007498	mesoderm development	8/293	135/18670	0,001327	0,003944	0,001738	*FGFR1/JAK2/ACVR1/TP63/ITGB4/ITGB3/ITGA2/VEGFA*	8
GO:0043457	regulation of cellular respiration	4/293	31/18670	0,001339	0,003974	0,001751	*HIF1A/IFNG/IL4/NOS2*	4
GO:0045737	positive regulation of cyclin-dependent protein serine/threonine kinase activity	4/293	31/18670	0,001339	0,003974	0,001751	*SRC/AKT1/EGFR/MAPRE3*	4
GO:0008593	regulation of Notch signaling pathway	7/293	105/18670	0,00134	0,003974	0,001751	*AKT1/STAT3/EGFR/NOS3/GATA2/TP63/EGF*	7
GO:0051607	defense response to virus	11/293	238/18670	0,0014	0,004149	0,001828	*IL1B/IL6/FLNA/BCL2/MMP12/LILRB1/IL12B/EIF2AK2/IFI6/IL23R/IFNG*	11
GO:0014743	regulation of muscle hypertrophy	6/293	78/18670	0,001415	0,004189	0,001846	*MTOR/TNFRSF1A/AGT/HAMP/IGF1/PDE5A*	6
GO:0033143	regulation of intracellular steroid hormone receptor signaling pathway	6/293	78/18670	0,001415	0,004189	0,001846	*SRC/AR/ESR1/TP63/BRCA1/CNOT1*	6
GO:0003300	cardiac muscle hypertrophy	7/293	106/18670	0,001416	0,004189	0,001846	*MTOR/TNFRSF1A/AGT/HAMP/IGF1/LEP/PDE5A*	7
GO:0000186	activation of MAPKK activity	5/293	53/18670	0,001426	0,004209	0,001855	*EGFR/JAK2/TGFBR1/EIF2AK2/EGF*	5
GO:0032715	negative regulation of interleukin-6 production	5/293	53/18670	0,001426	0,004209	0,001855	*TLR4/TNF/HAVCR2/FOXP3/IL10*	5
GO:0035065	regulation of histone acetylation	5/293	53/18670	0,001426	0,004209	0,001855	*GATA2/IL1B/BRCA1/TGFB1/FOXP3*	5
GO:1902099	regulation of metaphase/anaphase transition of cell cycle	5/293	53/18670	0,001426	0,004209	0,001855	*LCMT1/APC/BUB1/BUB1B/BUB3*	5
GO:0051100	negative regulation of binding	9/293	169/18670	0,001431	0,004221	0,00186	*ACE/AKT1/GATA1/JAK2/ADRB2/BAX/MAP2/HFE/IL10*	9
GO:0006816	calcium ion transport	16/293	434/18670	0,001454	0,004286	0,001889	*TRPC6/NOS3/LGALS3/ADRA2A/CCR5/AGT/F2/IL16/BAX/BCL2/LILRB1/TGFB1/EGF/FASLG/FMR1/TRPC1*	16
GO:0072655	establishment of protein localization to mitochondrion	8/293	137/18670	0,001458	0,004295	0,001892	*HSPA1L/TP53/AKT1/BAD/TP63/TP73/BAX/BCL2*	8
GO:0043552	positive regulation of phosphatidylinositol 3-kinase activity	4/293	32/18670	0,001511	0,004422	0,001949	*SRC/TGFB1/FGFR3/FLT1*	4

ID	Description	GeneRatio	BgRatio	pvalue	p.adjust	qvalue	geneID	Count
GO:0045922	negative regulation of fatty acid metabolic process	4/293	32/18670	0,001511	0,004422	0,001949	*AKT1/BRCA1/CEACAM1/INS*	4
GO:1900745	positive regulation of p38MAPK cascade	4/293	32/18670	0,001511	0,004422	0,001949	*BMP2/IL1B/VEGFA/LEP*	4
GO:2000778	positive regulation of interleukin-6 secretion	4/293	32/18670	0,001511	0,004422	0,001949	*AIF1/IL1B/TNF/HMGB1*	4
GO:0030730	sequestering of triglyceride	3/293	15/18670	0,001513	0,004422	0,001949	*PPARG/IL1B/TNF*	3
GO:0032645	regulation of granulocyte macrophage colony-stimulating factor production	3/293	15/18670	0,001513	0,004422	0,001949	*IL1B/IL12B/IL23R*	3
GO:0034349	glial cell apoptotic process	3/293	15/18670	0,001513	0,004422	0,001949	*TP53/CASP3/CASP9*	3
GO:0048569	post-embryonic animal organ development	3/293	15/18670	0,001513	0,004422	0,001949	*BAX/VEGFA/FBN1*	3
GO:0051883	killing of cells in other organism involved in symbiotic interaction	3/293	15/18670	0,001513	0,004422	0,001949	*BAD/F2/MBL2*	3
GO:0060099	regulation of phagocytosis, engulfment	3/293	15/18670	0,001513	0,004422	0,001949	*GATA2/PPARG/ITGA2*	3
GO:1900119	positive regulation of execution phase of apoptosis	3/293	15/18670	0,001513	0,004422	0,001949	*TP53/IL6/BAX*	3
GO:2001028	positive regulation of endothelial cell chemotaxis	3/293	15/18670	0,001513	0,004422	0,001949	*FGFR1/VEGFA/KDR*	3
GO:2001185	regulation of CD8-positive, alpha-beta T cell activation	3/293	15/18670	0,001513	0,004422	0,001949	*HLA-E/LILRB1/HFE*	3
GO:0007091	metaphase/anaphase transition of mitotic cell cycle	5/293	54/18670	0,001552	0,004526	0,001994	*LCMT1/APC/BUB1/BUB1B/BUB3*	5
GO:0030199	collagen fibril organization	5/293	54/18670	0,001552	0,004526	0,001994	*TGFBR1/COL1A1/COL5A1/COL5A2/COL1A2*	5
GO:0070206	protein trimerization	5/293	54/18670	0,001552	0,004526	0,001994	*HLA-G/LCN2/COL1A1/COL6A1/COL1A2*	5
GO:0016241	regulation of macroautophagy	9/293	171/18670	0,001553	0,004526	0,001994	*TP53/AKT1/CASP3/MTOR/IKBKG/ADRB2/HIF1A/KDR/IL4*	9

ID	Description	GeneRatio	BgRatio	pvalue	p.adjust	qvalue	geneID	Count
GO:0032526	response to retinoic acid	7/293	108/18670	0,001578	0,004593	0,002024	*PPARG/RBP4/CREB1/RXRB/COL1A1/IGF2R/LEP*	7
GO:0071156	regulation of cell cycle arrest	7/293	108/18670	0,001578	0,004593	0,002024	*TP53/MDM2/TP73/BAX/BRCA1/CNOT1/TGFB1*	7
GO:0000082	G1/S transition of mitotic cell cycle	12/293	279/18670	0,001591	0,004628	0,00204	*TP53/AIF1/AKT1/EGFR/MDM2/ACVR1/BAX/BCL2/TYMS/ RRM2/CNOT1/DHFR*	12
GO:1901987	regulation of cell cycle phase transition	17/293	480/18670	0,001603	0,004661	0,002054	*TP53/AIF1/AKT1/EGFR/MDM2/LCMT1/BAX/BCL2/APC/B UB1/BUB1B/BUB3/UBD/BRCA1/TUBA1A/CNOT1/TGFB1*	17
GO:0032874	positive regulation of stress-activated MAPK cascade	9/293	172/18670	0,001616	0,004697	0,00207	*TLR4/BMP2/IL1B/TNF/HMGB1/VEGFA/EIF2AK2/IL1RN/L EP*	9
GO:0014897	striated muscle hypertrophy	7/293	109/18670	0,001664	0,004829	0,002128	*MTOR/TNFRSF1A/AGT/HAMP/IGF1/LEP/PDE5A*	7
GO:0072009	nephron epithelium development	7/293	109/18670	0,001664	0,004829	0,002128	*BMP2/AGT/BCL2/VEGFA/TGFB1/FGF1/PGF*	7
GO:0072073	kidney epithelium development	8/293	140/18670	0,001673	0,004853	0,002138	*FGFR1/BMP2/AGT/BCL2/VEGFA/TGFB1/FGF1/PGF*	8
GO:0070304	positive regulation of stress-activated protein kinase signaling cascade	9/293	173/18670	0,001682	0,004874	0,002148	*TLR4/BMP2/IL1B/TNF/HMGB1/VEGFA/EIF2AK2/IL1RN/L EP*	9
GO:0010823	negative regulation of mitochondrion organization	5/293	55/18670	0,001686	0,004874	0,002148	*TP53/AKT1/HSPA1A/BCL2L1/IGF1*	5
GO:0042306	regulation of protein import into nucleus	5/293	55/18670	0,001686	0,004874	0,002148	*RAN/FLNA/TGFB1/IFNG/LEP*	5
GO:0045620	negative regulation of lymphocyte differentiation	5/293	55/18670	0,001686	0,004874	0,002148	*INHA/HMGB1/CTLA4/FOXP3/IL4R*	5
GO:0061098	positive regulation of protein tyrosine kinase activity	5/293	55/18670	0,001686	0,004874	0,002148	*ACE/SRC/ADRA2A/AGT/EGF*	5
GO:0002717	positive regulation of natural killer cell mediated immunity	4/293	33/18670	0,001699	0,004883	0,002152	*HLA-E/HLA-G/IL12B/IL12A*	4
GO:0002756	MyD88-independent toll-like receptor signaling pathway	4/293	33/18670	0,001699	0,004883	0,002152	*TLR4/CHUK/IKBKG/CD14*	4
GO:0014072	response to isoquinoline alkaloid	4/293	33/18670	0,001699	0,004883	0,002152	*AIF1/MDM2/MTOR/ADA*	4
GO:0030851	granulocyte differentiation	4/293	33/18670	0,001699	0,004883	0,002152	*GATA2/GATA1/CEACAM1/CEBPA*	4
GO:0032633	interleukin-4 production	4/293	33/18670	0,001699	0,004883	0,002152	*HAVCR2/HLA-E/CEBPB/FOXP3*	4

ID	Description	GeneRatio	BgRatio	pvalue	p.adjust	qvalue	geneID	Count
GO:0036003	positive regulation of transcription from RNA polymerase II promoter in response to stress	4/293	33/18670	0,001699	0,004883	0,002152	*TP53/HIF1A/CEBPB/VEGFA*	4
GO:0036314	response to sterol	4/293	33/18670	0,001699	0,004883	0,002152	*TGFBR1/CCR5/F7/TGFB1*	4
GO:0043278	response to morphine	4/293	33/18670	0,001699	0,004883	0,002152	*AIF1/MDM2/MTOR/ADA*	4
GO:0045736	negative regulation of cyclin-dependent protein serine/threonine kinase activity	4/293	33/18670	0,001699	0,004883	0,002152	*CASP3/NR2F2/APC/CEBPA*	4
GO:2000758	positive regulation of peptidyl-lysine acetylation	4/293	33/18670	0,001699	0,004883	0,002152	*IL1B/BRCA1/TGFB1/FOXP3*	4
GO:0034765	regulation of ion transmembrane transport	17/293	483/18670	0,001712	0,004917	0,002167	*ABCB1/AKT1/TRPC6/MTOR/ADRA2A/ADRB2/AGT/F2/FLNA/BAX/MMP9/CFTR/TGFB1/FMR1/IFNG/HAMP/TRPC1*	17
GO:0007045	cell-substrate adherens junction assembly	6/293	81/18670	0,00172	0,004934	0,002174	*SRC/HRG/BCL2/ITGA2/VEGFA/KDR*	6
GO:0048041	focal adhesion assembly	6/293	81/18670	0,00172	0,004934	0,002174	*SRC/HRG/BCL2/ITGA2/VEGFA/KDR*	6
GO:0070585	protein localization to mitochondrion	8/293	141/18670	0,00175	0,005018	0,002211	*HSPA1L/TP53/AKT1/BAD/TP63/TP73/BAX/BCL2*	8
GO:0032409	regulation of transporter activity	12/293	283/18670	0,001792	0,005135	0,002263	*ABCB1/TRPC6/PPARG/ADRA2A/ADRB2/SGK1/BCL2/MMP9/CFTR/INS/FMR1/IFNG*	12
GO:0010921	regulation of phosphatase activity	9/293	175/18670	0,00182	0,005212	0,002297	*MTOR/LGALS3/IGFBP3/JAK2/BMP2/TNF/ITGA2/EIF2AK2/IFNG*	9
GO:0044784	metaphase/anaphase transition of cell cycle	5/293	56/18670	0,001827	0,005223	0,002302	*LCMT1/APC/BUB1/BUB1B/BUB3*	5
GO:0090183	regulation of kidney development	5/293	56/18670	0,001827	0,005223	0,002302	*AGT/MMP9/VEGFA/TGFB1/IL6R*	5
GO:1903078	positive regulation of protein localization to plasma membrane	5/293	56/18670	0,001827	0,005223	0,002302	*AKT1/EGFR/LGALS3/TNF/IFNG*	5
GO:0002070	epithelial cell maturation	3/293	16/18670	0,001841	0,005225	0,002302	*HIF1A/TYMS/PGR*	3
GO:0002739	regulation of cytokine secretion involved in immune response	3/293	16/18670	0,001841	0,005225	0,002302	*TNF/LILRB1/IL10*	3
GO:0006837	serotonin transport	3/293	16/18670	0,001841	0,005225	0,002302	*HTR1A/ITGB3/LILRB1*	3

ID	Description	GeneRatio	BgRatio	pvalue	p.adjust	qvalue	geneID	Count
GO:0010225	response to UV-C	3/293	16/18670	0,001841	0,005225	0,002302	*TP53/MDM2/BRCA2*	3
GO:0030889	negative regulation of B cell proliferation	3/293	16/18670	0,001841	0,005225	0,002302	*CASP3/CTLA4/IL10*	3
GO:0032604	granulocyte macrophage colony-stimulating factor production	3/293	16/18670	0,001841	0,005225	0,002302	*IL1B/IL12B/IL23R*	3
GO:0032695	negative regulation of interleukin-12 production	3/293	16/18670	0,001841	0,005225	0,002302	*NFKB1/LILRB1/IL10*	3
GO:0060572	morphogenesis of an epithelial bud	3/293	16/18670	0,001841	0,005225	0,002302	*AR/TP63/SULF1*	3
GO:0090335	regulation of brown fat cell differentiation	3/293	16/18670	0,001841	0,005225	0,002302	*MTOR/INS/LEP*	3
GO:1902004	positive regulation of amyloid-beta formation	3/293	16/18670	0,001841	0,005225	0,002302	*CASP3/TNF/IFNG*	3
GO:1905153	regulation of membrane invagination	3/293	16/18670	0,001841	0,005225	0,002302	*GATA2/PPARG/ITGA2*	3
GO:2000846	regulation of corticosteroid hormone secretion	3/293	16/18670	0,001841	0,005225	0,002302	*AGTR1/AGT/REN*	3
GO:0014896	muscle hypertrophy	7/293	111/18670	0,001847	0,005238	0,002308	*MTOR/TNFRSF1A/AGT/HAMP/IGF1/LEP/PDE5A*	7
GO:0030111	regulation of Wnt signaling pathway	14/293	363/18670	0,001877	0,005321	0,002345	*NFKB1/SRC/EGFR/ESR1/BMP2/IGFBP1/APC/APOE/IGFBP4/COL1A1/SULF1/EGF/FOXP3/IGFBP6*	14
GO:0031128	developmental induction	4/293	34/18670	0,001902	0,005384	0,002373	*AR/FGFR1/BMP2/FGF1*	4
GO:1904030	negative regulation of cyclin-dependent protein kinase activity	4/293	34/18670	0,001902	0,005384	0,002373	*CASP3/NR2F2/APC/CEBPA*	4
GO:0044344	cellular response to fibroblast growth factor stimulus	8/293	143/18670	0,001913	0,005411	0,002385	*FGFR1/FLRT3/COL1A1/SULF1/CXCL8/FGF1/FGFR3/FGF7*	8
GO:0046434	organophosphate catabolic process	11/293	248/18670	0,001938	0,005477	0,002413	*TP53/STAT3/ADA/ARNT/HIF1A/INSR/INS/IFNG/SLC4A1/IGF1/PDE5A*	11
GO:1901991	negative regulation of mitotic cell cycle phase transition	11/293	248/18670	0,001938	0,005477	0,002413	*TP53/MDM2/LCMT1/BAX/BCL2/APC/BUB1/BUB1B/BUB3/BRCA1/CNOT1*	11
GO:0014902	myotube differentiation	7/293	112/18670	0,001944	0,005478	0,002414	*ACTA1/MTOR/BCL2/CD9/CEACAM5/IGF1/IL4R*	7

ID	Description	GeneRatio	BgRatio	pvalue	p.adjust	qvalue	geneID	Count
GO:0015918	sterol transport	7/293	112/18670	0,001944	0,005478	0,002414	*NFKB1/PPARG/APOB/APOE/CFTR/EGF/LEP*	7
GO:0042303	molting cycle	7/293	112/18670	0,001944	0,005478	0,002414	*EGFR/TNF/TP63/BCL2/NSDHL/FST/FGF7*	7
GO:0042633	hair cycle	7/293	112/18670	0,001944	0,005478	0,002414	*EGFR/TNF/TP63/BCL2/NSDHL/FST/FGF7*	7
GO:0043279	response to alkaloid	7/293	112/18670	0,001944	0,005478	0,002414	*AIF1/MDM2/CASP3/MTOR/PPARG/ADA/BCL2L1*	7
GO:0060218	hematopoietic stem cell differentiation	6/293	83/18670	0,001949	0,005487	0,002418	*ACE/TP53/GATA2/GATA1/TP73/EIF2AK2*	6
GO:0002066	columnar/cuboidal epithelial cell development	5/293	57/18670	0,001978	0,005549	0,002445	*FGFR1/BAD/HIF1A/HNF4A/TYMS*	5
GO:0010965	regulation of mitotic sister chromatid separation	5/293	57/18670	0,001978	0,005549	0,002445	*LCMT1/APC/BUB1/BUB1B/BUB3*	5
GO:0031294	lymphocyte costimulation	5/293	57/18670	0,001978	0,005549	0,002445	*SRC/AKT1/CD320/CTLA4/EFNB1*	5
GO:0051055	negative regulation of lipid biosynthetic process	5/293	57/18670	0,001978	0,005549	0,002445	*NFKB1/BMP2/APOE/BRCA1/CEACAM1*	5
GO:0060043	regulation of cardiac muscle cell proliferation	5/293	57/18670	0,001978	0,005549	0,002445	*PIM1/FGFR1/TGFBR1/TP73/RBP4*	5
GO:0071398	cellular response to fatty acid	5/293	57/18670	0,001978	0,005549	0,002445	*SRC/AKT1/PPARG/APOB/CREB1*	5
GO:0050821	protein stabilization	9/293	178/18670	0,002044	0,005732	0,002526	*PIM1/TP53/VHL/HSPA1B/HSPA1A/FLNA/BAG6/CREB1/IGF1*	9
GO:0060964	regulation of gene silencing by miRNA	7/293	113/18670	0,002045	0,005732	0,002526	*TP53/STAT3/EGFR/ESR1/PPARG/TGFB1/FMR1*	7
GO:0043154	negative regulation of cysteine-type endopeptidase activity involved in apoptotic process	6/293	84/18670	0,002071	0,005802	0,002557	*SRC/AKT1/MDM2/MMP9/VEGFA/IFI6*	6
GO:0071901	negative regulation of protein serine/threonine kinase activity	8/293	145/18670	0,002087	0,005841	0,002574	*AKT1/CASP3/NR2F2/GSTP1/IL1B/APC/APOE/CEBPA*	8
GO:1903050	regulation of proteolysis involved in cellular protein catabolic process	10/293	214/18670	0,002106	0,005888	0,002594	*AKT1/MDM2/HSPA1B/HSPA1A/APOE/BAG6/CEBPA/EGF/FMR1/HFE*	10
GO:2001020	regulation of response to DNA damage stimulus	10/293	214/18670	0,002106	0,005888	0,002594	*TP53/EGFR/MDM2/UBE2N/CASP9/BCL2/BCL2L1/BRCA1/HMGB1/FMR1*	10

ID	Description	GeneRatio	BgRatio	pvalue	p.adjust	qvalue	geneID	Count
GO:0042092	type 2 immune response	4/293	35/18670	0,002121	0,005909	0,002604	*IL6/IL4R/IL4/IL10*	4
GO:0045622	regulation of T-helper cell differentiation	4/293	35/18670	0,002121	0,005909	0,002604	*IL12B/IL23R/FOXP3/IL4R*	4
GO:0048333	mesodermal cell differentiation	4/293	35/18670	0,002121	0,005909	0,002604	*FGFR1/ITGB4/ITGB3/ITGA2*	4
GO:0071312	cellular response to alkaloid	4/293	35/18670	0,002121	0,005909	0,002604	*AIF1/MDM2/CASP3/BCL2L1*	4
GO:0098751	bone cell development	4/293	35/18670	0,002121	0,005909	0,002604	*SRC/LTF/LILRB1/FBN1*	4
GO:1904031	positive regulation of cyclin-dependent protein kinase activity	4/293	35/18670	0,002121	0,005909	0,002604	*SRC/AKT1/EGFR/MAPRE3*	4
GO:0008016	regulation of heart contraction	11/293	251/18670	0,002129	0,005928	0,002612	*ACE2/THRB/MDM2/NOS3/JAK2/ADA/AGT/FLNA/DES/TRPC1/PDE5A*	11
GO:0071385	cellular response to glucocorticoid stimulus	5/293	58/18670	0,002137	0,005943	0,002619	*NR3C1/EGFR/GSTP1/CASP9/TGFB1*	5
GO:1904589	regulation of protein import	5/293	58/18670	0,002137	0,005943	0,002619	*RAN/FLNA/TGFB1/IFNG/LEP*	5
GO:0010633	negative regulation of epithelial cell migration	7/293	114/18670	0,00215	0,00597	0,002631	*NR2F2/PPARG/HRG/APOE/HMGB1/TGFB1/IL4*	7
GO:0042752	regulation of circadian rhythm	7/293	114/18670	0,00215	0,00597	0,002631	*TP53/MTOR/PPARG/ADA/HNF4A/CREB1/CRTC1*	7
GO:0060349	bone morphogenesis	7/293	114/18670	0,00215	0,00597	0,002631	*MBL2/LTF/TGFB1/COL1A1/COL6A3/COL6A1/FGFR3*	7
GO:2001251	negative regulation of chromosome organization	8/293	146/18670	0,002178	0,006044	0,002663	*SRC/LCMT1/APC/BUB1/BUB1B/BUB3/BRCA1/FOXP3*	8
GO:1901796	regulation of signal transduction by p53 class mediator	9/293	180/18670	0,002205	0,006074	0,002677	*TP53/AKT1/MDM2/TP63/TP73/BCL2/DNA2/BRIP1/BRCA1*	9
GO:0006978	DNA damage response, signal transduction by p53 class mediator resulting in transcription of p21 class mediator	3/293	17/18670	0,00221	0,006074	0,002677	*TP53/BRCA1/BRCA2*	3
GO:0010224	response to UV-B	3/293	17/18670	0,00221	0,006074	0,002677	*BCL2/IL12B/IL12A*	3
GO:0015732	prostaglandin transport	3/293	17/18670	0,00221	0,006074	0,002677	*IL1B/LEP/NOS2*	3

ID	Description	GeneRatio	BgRatio	pvalue	p.adjust	qvalue	geneID	Count
GO:0034433	steroid esterification	3/293	17/18670	0,00221	0,006074	0,002677	*AGTR1/AGT/APOE*	3
GO:0034434	sterol esterification	3/293	17/18670	0,00221	0,006074	0,002677	*AGTR1/AGT/APOE*	3
GO:0034435	cholesterol esterification	3/293	17/18670	0,00221	0,006074	0,002677	*AGTR1/AGT/APOE*	3
GO:0035313	wound healing, spreading of epidermal cells	3/293	17/18670	0,00221	0,006074	0,002677	*MTOR/MMP12/COL5A1*	3
GO:0035930	corticosteroid hormone secretion	3/293	17/18670	0,00221	0,006074	0,002677	*AGTR1/AGT/REN*	3
GO:0051709	regulation of killing of cells of other organism	3/293	17/18670	0,00221	0,006074	0,002677	*BAD/IFNG/NOS2*	3
GO:0051818	disruption of cells of other organism involved in symbiotic interaction	3/293	17/18670	0,00221	0,006074	0,002677	*BAD/F2/MBL2*	3
GO:0051969	regulation of transmission of nerve impulse	3/293	17/18670	0,00221	0,006074	0,002677	*AGT/ITGA2/FMR1*	3
GO:0060644	mammary gland epithelial cell differentiation	3/293	17/18670	0,00221	0,006074	0,002677	*AKT1/HIF1A/CEBPB*	3
GO:0060850	regulation of transcription involved in cell fate commitment	3/293	17/18670	0,00221	0,006074	0,002677	*NR2F2/PPARG/CEBPB*	3
GO:1902931	negative regulation of alcohol biosynthetic process	3/293	17/18670	0,00221	0,006074	0,002677	*NFKB1/BMP2/APOE*	3
GO:2001267	regulation of cysteine-type endopeptidase activity involved in apoptotic signaling pathway	3/293	17/18670	0,00221	0,006074	0,002677	*JAK2/BAX/MMP9*	3
GO:0043524	negative regulation of neuron apoptotic process	8/293	147/18670	0,002273	0,006245	0,002752	*JAK2/HIF1A/BAX/BCL2/BCL2L1/APOE/C5AR1/CEBPB*	8
GO:0051147	regulation of muscle cell differentiation	9/293	181/18670	0,00229	0,006286	0,00277	*MTOR/BMP2/BCL2/CEACAM5/TGFB1/IGF2/HAMP/IGF1/IL4R*	9
GO:0045666	positive regulation of neuron differentiation	14/293	371/18670	0,002293	0,00629	0,002772	*TRPC6/GATA2/MTOR/FGFR1/FN1/L1CAM/BMP2/AGT/BCL2/APOE/VEGFA/CRTC1/TIMP2/FMR1*	14

ID	Description	GeneRatio	BgRatio	pvalue	p.adjust	qvalue	geneID	Count
GO:0001885	endothelial cell development	5/293	59/18670	0,002305	0,006311	0,002781	*TNFRSF1A/IL1B/TNF/PROC/VEGFA*	5
GO:0032732	positive regulation of interleukin-1 production	5/293	59/18670	0,002305	0,006311	0,002781	*TLR4/JAK2/HAVCR2/HMGB1/IFNG*	5
GO:0032890	regulation of organic acid transport	5/293	59/18670	0,002305	0,006311	0,002781	*ACE2/AKT1/IL1B/AGT/LEP*	5
GO:2000756	regulation of peptidyl-lysine acetylation	5/293	59/18670	0,002305	0,006311	0,002781	*GATA2/IL1B/BRCA1/TGFB1/FOXP3*	5
GO:0017157	regulation of exocytosis	10/293	217/18670	0,002331	0,006373	0,002808	*GATA2/ADRA2A/FGG/FGB/FGA/CEACAM1/CFTR/FMR1/IFNG/IL4R*	10
GO:0071241	cellular response to inorganic substance	10/293	217/18670	0,002331	0,006373	0,002808	*AKT1/EGFR/CHUK/BAD/MMP3/MMP9/CEBPA/CREB1/FMR1/HFE*	10
GO:0070542	response to fatty acid	6/293	86/18670	0,002335	0,00638	0,002811	*SRC/AKT1/BAD/PPARG/APOB/CREB1*	6
GO:0010453	regulation of cell fate commitment	4/293	36/18670	0,002357	0,006427	0,002832	*AR/FGFR1/IL12B/IL23R*	4
GO:0042554	superoxide anion generation	4/293	36/18670	0,002357	0,006427	0,002832	*EGFR/GSTP1/AGT/TGFB1*	4
GO:0071392	cellular response to estradiol stimulus	4/293	36/18670	0,002357	0,006427	0,002832	*EGFR/ESR1/ITGA2/IL10*	4
GO:0071634	regulation of transforming growth factor beta production	4/293	36/18670	0,002357	0,006427	0,002832	*HIF1A/ITGB6/CREB1/FOXP3*	4
GO:0008643	carbohydrate transport	8/293	148/18670	0,002371	0,006461	0,002847	*AKT1/IL1B/TNF/INSR/INS/HNF1A/IGF1/LEP*	8
GO:0007569	cell aging	7/293	116/18670	0,002373	0,006464	0,002848	*TP53/MTOR/TP63/BCL2/SERPINE1/BRCA2/HLA-G*	7
GO:0009566	fertilization	9/293	182/18670	0,002376	0,006468	0,00285	*HSPA1L/TRPC6/AR/NR2F2/BAX/BCL2L1/FETUB/APOB/CD9*	9
GO:0019722	calcium-mediated signaling	10/293	218/18670	0,00241	0,006555	0,002888	*EGFR/MTOR/ADA/AGTR1/CCR5/TNF/CXCL8/CXCR1/KDR/IGF1*	10
GO:0061136	regulation of proteasomal protein catabolic process	9/293	183/18670	0,002466	0,006704	0,002954	*AKT1/MDM2/HSPA1B/HSPA1A/APOE/BAG6/CEBPA/FMR1/HFE*	9
GO:2000058	regulation of ubiquitin-dependent protein catabolic process	8/293	149/18670	0,002472	0,006718	0,00296	*AKT1/MDM2/HSPA1B/HSPA1A/BAG6/CEBPA/EGF/HFE*	8
GO:0007611	learning or memory	11/293	256/18670	0,002481	0,006736	0,002968	*EGFR/CASP3/MTOR/AGT/HIF1A/SGK1/APOE/CEBPB/CREB1/CRTC1/INSR*	11
GO:0051306	mitotic sister chromatid separation	5/293	60/18670	0,002483	0,006736	0,002968	*LCMT1/APC/BUB1/BUB1B/BUB3*	5

ID	Description	GeneRatio	BgRatio	pvalue	p.adjust	qvalue	geneID	Count
GO:0051784	negative regulation of nuclear division	5/293	60/18670	0,002483	0,006736	0,002968	*LCMT1/APC/BUB1/BUB1B/BUB3*	5
GO:0007613	memory	7/293	117/18670	0,002491	0,006754	0,002976	*MTOR/SGK1/APOE/CEBPB/CREB1/CRTC1/INSR*	7
GO:0033673	negative regulation of kinase activity	11/293	257/18670	0,002557	0,006926	0,003052	*AKT1/CASP3/NR2F2/IGF1R/GSTP1/IL1B/APC/APOE/CEACAM1/CEBPA/IFNG*	11
GO:0071774	response to fibroblast growth factor	8/293	150/18670	0,002577	0,006978	0,003075	*FGFR1/FLRT3/COL1A1/SULF1/CXCL8/FGF1/FGFR3/FGF7*	8
GO:0034405	response to fluid shear stress	4/293	37/18670	0,002611	0,00699	0,00308	*SRC/AKT1/NOS3/TGFB1*	4
GO:0043114	regulation of vascular permeability	4/293	37/18670	0,002611	0,00699	0,00308	*SRC/CEACAM1/VEGFA/TGFB1*	4
GO:0043243	positive regulation of protein complex disassembly	4/293	37/18670	0,002611	0,00699	0,00308	*IGF1R/ADRB2/TNF/INSR*	4
GO:0071276	cellular response to cadmium ion	4/293	37/18670	0,002611	0,00699	0,00308	*AKT1/EGFR/CHUK/MMP9*	4
GO:1900026	positive regulation of substrate adhesion-dependent cell spreading	4/293	37/18670	0,002611	0,00699	0,00308	*FGG/FLNA/FGB/FGA*	4
GO:1990266	neutrophil migration	7/293	118/18670	0,002614	0,00699	0,00308	*LGALS3/IL1B/IL1R1/C5AR1/CXCL8/CXCR1/IL1RN*	7
GO:0002902	regulation of B cell apoptotic process	3/293	18/18670	0,002621	0,00699	0,00308	*ADA/BAX/IL10*	3
GO:0003414	chondrocyte morphogenesis involved in endochondral bone morphogenesis	3/293	18/18670	0,002621	0,00699	0,00308	*MBL2/COL6A3/COL6A1*	3
GO:0003429	growth plate cartilage chondrocyte morphogenesis	3/293	18/18670	0,002621	0,00699	0,00308	*MBL2/COL6A3/COL6A1*	3
GO:0006349	regulation of gene expression by genetic imprinting	3/293	18/18670	0,002621	0,00699	0,00308	*BRCA1/CTCFL/IGF2*	3
GO:0007252	I-kappaB phosphorylation	3/293	18/18670	0,002621	0,00699	0,00308	*AKT1/TLR4/CHUK*	3
GO:0007597	blood coagulation, intrinsic pathway	3/293	18/18670	0,002621	0,00699	0,00308	*F2/SERPINC1/F12*	3
GO:0031065	positive regulation of histone deacetylation	3/293	18/18670	0,002621	0,00699	0,00308	*TP53/VEGFA/TGFB1*	3
GO:0042772	DNA damage response, signal transduction	3/293	18/18670	0,002621	0,00699	0,00308	*TP53/BRCA1/BRCA2*	3

ID	Description	GeneRatio	BgRatio	pvalue	p.adjust	qvalue	geneID	Count
	resulting in transcription							
GO:0043011	myeloid dendritic cell differentiation	3/293	18/18670	0,002621	0,00699	0,00308	*UBD/TGFB1/IL4*	3
GO:0045780	positive regulation of bone resorption	3/293	18/18670	0,002621	0,00699	0,00308	*EGFR/FSHB/TFRC*	3
GO:0046852	positive regulation of bone remodeling	3/293	18/18670	0,002621	0,00699	0,00308	*EGFR/FSHB/TFRC*	3
GO:0070230	positive regulation of lymphocyte apoptotic process	3/293	18/18670	0,002621	0,00699	0,00308	*TP53/BAX/IL10*	3
GO:0070233	negative regulation of T cell apoptotic process	3/293	18/18670	0,002621	0,00699	0,00308	*ADA/HIF1A/TSC22D3*	3
GO:0072574	hepatocyte proliferation	3/293	18/18670	0,002621	0,00699	0,00308	*CPB2/CEACAM1/CEBPB*	3
GO:0072575	epithelial cell proliferation involved in liver morphogenesis	3/293	18/18670	0,002621	0,00699	0,00308	*CPB2/CEACAM1/CEBPB*	3
GO:0090026	positive regulation of monocyte chemotaxis	3/293	18/18670	0,002621	0,00699	0,00308	*AIF1/SERPINE1/HMGB1*	3
GO:0090171	chondrocyte morphogenesis	3/293	18/18670	0,002621	0,00699	0,00308	*MBL2/COL6A3/COL6A1*	3
GO:0090190	positive regulation of branching involved in ureteric bud morphogenesis	3/293	18/18670	0,002621	0,00699	0,00308	*AGT/VEGFA/TGFB1*	3
GO:0150078	positive regulation of neuroinflammatory response	3/293	18/18670	0,002621	0,00699	0,00308	*IL1B/TNF/IL6*	3
GO:2000319	regulation of T-helper 17 cell differentiation	3/293	18/18670	0,002621	0,00699	0,00308	*IL12B/IL23R/FOXP3*	3
GO:2000647	negative regulation of stem cell proliferation	3/293	18/18670	0,002621	0,00699	0,00308	*TP53/TGFB1/FBLN1*	3
GO:0046328	regulation of JNK cascade	9/293	185/18670	0,002653	0,007069	0,003115	*AKT1/TLR4/EGFR/IGF1R/GSTP1/IL1B/TNF/HMGB1/IL1RN*	9
GO:0071384	cellular response to corticosteroid stimulus	5/293	61/18670	0,002671	0,00711	0,003133	*NR3C1/EGFR/GSTP1/CASP9/TGFB1*	5

ID	Description	GeneRatio	BgRatio	pvalue	p.adjust	qvalue	geneID	Count
GO:2001244	positive regulation of intrinsic apoptotic signaling pathway	5/293	61/18670	0,002671	0,00711	0,003133	*TP53/BAD/BAX/BCL2/BCL2L1*	5
GO:0044843	cell cycle G1/S phase transition	12/293	298/18670	0,002743	0,007297	0,003215	*TP53/AIF1/AKT1/EGFR/MDM2/ACVR1/BAX/BCL2/TYMS/ RRM2/CNOT1/DHFR*	12
GO:0043903	regulation of symbiosis, encompassing mutualism through parasitism	10/293	222/18670	0,002748	0,007306	0,003219	*BAD/TNF/MBL2/BCL2/APOE/LTF/CXCL8/EIF2AK2/FMR 1/IGF2R*	10
GO:0055013	cardiac muscle cell development	6/293	89/18670	0,002775	0,007375	0,00325	*MTOR/AGT/VEGFA/NEB/HAMP/IGF1*	6
GO:0002753	cytoplasmic pattern recognition receptor signaling pathway	5/293	62/18670	0,002869	0,007611	0,003354	*TLR4/IKBKG/HSPA1B/HSPA1A/UBE2N*	5
GO:0032623	interleukin-2 production	5/293	62/18670	0,002869	0,007611	0,003354	*IL1B/HAVCR2/SLC11A1/FOXP3/IL1A*	5
GO:1905818	regulation of chromosome separation	5/293	62/18670	0,002869	0,007611	0,003354	*LCMT1/APC/BUB1/BUB1B/BUB3*	5
GO:0003298	physiological muscle hypertrophy	4/293	38/18670	0,002882	0,007617	0,003356	*MTOR/AGT/HAMP/IGF1*	4
GO:0003301	physiological cardiac muscle hypertrophy	4/293	38/18670	0,002882	0,007617	0,003356	*MTOR/AGT/HAMP/IGF1*	4
GO:0046326	positive regulation of glucose import	4/293	38/18670	0,002882	0,007617	0,003356	*AKT1/INSR/INS/IGF1*	4
GO:0046825	regulation of protein export from nucleus	4/293	38/18670	0,002882	0,007617	0,003356	*TP53/MDM2/IL1B/XPO5*	4
GO:0061049	cell growth involved in cardiac muscle cell development	4/293	38/18670	0,002882	0,007617	0,003356	*MTOR/AGT/HAMP/IGF1*	4
GO:0071604	transforming growth factor beta production	4/293	38/18670	0,002882	0,007617	0,003356	*HIF1A/ITGB6/CREB1/FOXP3*	4
GO:1904706	negative regulation of vascular smooth muscle cell proliferation	4/293	38/18670	0,002882	0,007617	0,003356	*PPARG/GSTP1/PRKG1/IL10*	4
GO:0032651	regulation of interleukin-1 beta production	6/293	90/18670	0,002935	0,007748	0,003414	*TLR4/JAK2/GSTP1/HMGB1/IFNG/IGF1*	6
GO:0034333	adherens junction assembly	6/293	90/18670	0,002935	0,007748	0,003414	*SRC/HRG/BCL2/ITGA2/VEGFA/KDR*	6
GO:0010769	regulation of cell morphogenesis	12/293	301/18670	0,002975	0,007848	0,003458	*TRPC6/FN1/L1CAM/FGG/FLNA/FGB/FGA/APOE/MAP2/ VEGFA/FBLN1/FMR1*	12

ID	Description	GeneRatio	BgRatio	pvalue	p.adjust	qvalue	geneID	Count
	involved in differentiation							
GO:0043618	regulation of transcription from RNA polymerase II promoter in response to stress	7/293	121/18670	0,003009	0,007933	0,003496	*TP53/VHL/HSPA1A/ARNT/HIF1A/CEBPB/VEGFA*	7
GO:0033157	regulation of intracellular protein transport	10/293	225/18670	0,003025	0,007972	0,003513	*HSPA1L/TP53/MDM2/RAN/IL1B/FLNA/XPO5/TGFB1/IFNG/LEP*	10
GO:0019362	pyridine nucleotide metabolic process	9/293	189/18670	0,00306	0,008015	0,003532	*TP53/STAT3/ARNT/HIF1A/INSR/INS/IFNG/SLC4A1/IGF1*	9
GO:0046496	nicotinamide nucleotide metabolic process	9/293	189/18670	0,00306	0,008015	0,003532	*TP53/STAT3/ARNT/HIF1A/INSR/INS/IFNG/SLC4A1/IGF1*	9
GO:0032371	regulation of sterol transport	5/293	63/18670	0,003077	0,008015	0,003532	*NFKB1/PPARG/APOE/EGF/LEP*	5
GO:0032374	regulation of cholesterol transport	5/293	63/18670	0,003077	0,008015	0,003532	*NFKB1/PPARG/APOE/EGF/LEP*	5
GO:0042982	amyloid precursor protein metabolic process	5/293	63/18670	0,003077	0,008015	0,003532	*CASP3/TNF/APOE/IFNG/IGF1*	5
GO:0090181	regulation of cholesterol metabolic process	5/293	63/18670	0,003077	0,008015	0,003532	*RAN/APOB/APOE/DHCR7/FGF1*	5
GO:1904377	positive regulation of protein localization to cell periphery	5/293	63/18670	0,003077	0,008015	0,003532	*AKT1/EGFR/LGALS3/TNF/IFNG*	5
GO:0001991	regulation of systemic arterial blood pressure by circulatory renin-angiotensin	3/293	19/18670	0,003077	0,008015	0,003532	*ACE/ACE2/REN*	3
GO:0002726	positive regulation of T cell cytokine production	3/293	19/18670	0,003077	0,008015	0,003532	*IL1B/IL6/IL1R1*	3
GO:0002922	positive regulation of humoral immune response	3/293	19/18670	0,003077	0,008015	0,003532	*IL1B/TNF/LTA*	3
GO:0003422	growth plate cartilage morphogenesis	3/293	19/18670	0,003077	0,008015	0,003532	*MBL2/COL6A3/COL6A1*	3
GO:0033189	response to vitamin A	3/293	19/18670	0,003077	0,008015	0,003532	*PPARG/TYMS/HAMP*	3

ID	Description	GeneRatio	BgRatio	pvalue	p.adjust	qvalue	geneID	Count
GO:0045063	T-helper 1 cell differentiation	3/293	19/18670	0,003077	0,008015	0,003532	*MTOR/HMGB1/IL4R*	3
GO:0045076	regulation of interleukin-2 biosynthetic process	3/293	19/18670	0,003077	0,008015	0,003532	*IL1B/FOXP3/IL1A*	3
GO:0051546	keratinocyte migration	3/293	19/18670	0,003077	0,008015	0,003532	*MTOR/MMP9/FGF7*	3
GO:0061081	positive regulation of myeloid leukocyte cytokine production involved in immune response	3/293	19/18670	0,003077	0,008015	0,003532	*TLR4/HLA-G/LILRB1*	3
GO:0072576	liver morphogenesis	3/293	19/18670	0,003077	0,008015	0,003532	*CPB2/CEACAM1/CEBPB*	3
GO:0090201	negative regulation of release of cytochrome c from mitochondria	3/293	19/18670	0,003077	0,008015	0,003532	*AKT1/BCL2L1/IGF1*	3
GO:1900409	positive regulation of cellular response to oxidative stress	3/293	19/18670	0,003077	0,008015	0,003532	*TLR4/TNF/MMP3*	3
GO:1902176	negative regulation of oxidative stress-induced intrinsic apoptotic signaling pathway	3/293	19/18670	0,003077	0,008015	0,003532	*AKT1/HIF1A/INS*	3
GO:2000831	regulation of steroid hormone secretion	3/293	19/18670	0,003077	0,008015	0,003532	*AGTR1/AGT/REN*	3
GO:0006913	nucleocytoplasmic transport	13/293	343/18670	0,003131	0,008151	0,003592	*TP53/AKT1/STAT3/MDM2/RAN/IL1B/AGT/FLNA/MMP12/XPO5/TGFB1/IFNG/LEP*	13
GO:0032434	regulation of proteasomal ubiquitin-dependent protein catabolic process	7/293	122/18670	0,00315	0,008196	0,003612	*AKT1/MDM2/HSPA1B/HSPA1A/BAG6/CEBPA/HFE*	7
GO:0060968	regulation of gene silencing	8/293	155/18670	0,003153	0,0082	0,003613	*TP53/STAT3/EGFR/ESR1/PPARG/XPO5/TGFB1/FMR1*	8
GO:0002251	organ or tissue specific immune response	4/293	39/18670	0,003172	0,008232	0,003627	*IL6/LTF/NOS2/IL6R*	4
GO:0032692	negative regulation of interleukin-1 production	4/293	39/18670	0,003172	0,008232	0,003627	*GSTP1/CEACAM1/IGF1/IL10*	4
GO:0060969	negative regulation of gene silencing	4/293	39/18670	0,003172	0,008232	0,003627	*TP53/ESR1/PPARG/TGFB1*	4

ID	Description	GeneRatio	BgRatio	pvalue	p.adjust	qvalue	geneID	Count
GO:2000826	regulation of heart morphogenesis	4/293	39/18670	0,003172	0,008232	0,003627	*PIM1/ACVR1/TGFBR1/BMP2*	4
GO:0045216	cell-cell junction organization	8/293	156/18670	0,00328	0,008505	0,003748	*ACE/ACE2/TGFBR1/TNF/AGT/APC/CD9/TGFB1*	8
GO:0016445	somatic diversification of immunoglobulins	5/293	64/18670	0,003295	0,008537	0,003762	*TGFB1/FOXP3/TFRC/IL4/IL10*	5
GO:0043550	regulation of lipid kinase activity	5/293	64/18670	0,003295	0,008537	0,003762	*SRC/F2/TGFB1/FGFR3/FLT1*	5
GO:0051169	nuclear transport	13/293	346/18670	0,003371	0,008727	0,003846	*TP53/AKT1/STAT3/MDM2/RAN/IL1B/AGT/FLNA/MMP12/ XPO5/TGFB1/IFNG/LEP*	13
GO:0031570	DNA integrity checkpoint	8/293	157/18670	0,00341	0,008824	0,003888	*TP53/MDM2/BAX/DNA2/BRIP1/BRCA1/MDC1/CNOT1*	8
GO:1901988	negative regulation of cell cycle phase transition	11/293	267/18670	0,003422	0,008849	0,003899	*TP53/MDM2/LCMT1/BAX/BCL2/APC/BUB1/BUB1B/BUB3 /BRCA1/CNOT1*	11
GO:0019835	cytolysis	4/293	40/18670	0,003482	0,008981	0,003957	*F2/HRG/LILRB1/TGFB1*	4
GO:0050832	defense response to fungus	4/293	40/18670	0,003482	0,008981	0,003957	*HRG/LTF/TGFB1/HAMP*	4
GO:0071548	response to dexamethasone	4/293	40/18670	0,003482	0,008981	0,003957	*NR3C1/EGFR/CASP9/TGFB1*	4
GO:0090184	positive regulation of kidney development	4/293	40/18670	0,003482	0,008981	0,003957	*AGT/VEGFA/TGFB1/IL6R*	4
GO:2000008	regulation of protein localization to cell surface	4/293	40/18670	0,003482	0,008981	0,003957	*AKT1/TNF/EGF/HFE*	4
GO:0051224	negative regulation of protein transport	9/293	193/18670	0,003515	0,009061	0,003993	*FN1/ADRA2A/IL1B/TNF/APOE/LILRB1/INS/FOXP3/IL10*	9
GO:0006940	regulation of smooth muscle contraction	5/293	65/18670	0,003525	0,009082	0,004002	*ADA/ADRA2A/ADRB2/PRKG1/ITGA2*	5
GO:0002374	cytokine secretion involved in immune response	3/293	20/18670	0,003578	0,009189	0,004049	*TNF/LILRB1/IL10*	3
GO:0030220	platelet formation	3/293	20/18670	0,003578	0,009189	0,004049	*CASP3/GATA1/CASP9*	3
GO:0033194	response to hydroperoxide	3/293	20/18670	0,003578	0,009189	0,004049	*AIF1/CHUK/JAK2*	3
GO:0043371	negative regulation of CD4-positive, alpha-beta T cell differentiation	3/293	20/18670	0,003578	0,009189	0,004049	*HMGB1/FOXP3/IL4R*	3
GO:0060602	branch elongation of an epithelium	3/293	20/18670	0,003578	0,009189	0,004049	*ESR1/TGFB1/FGF1*	3
GO:1902993	positive regulation of amyloid precursor	3/293	20/18670	0,003578	0,009189	0,004049	*CASP3/TNF/IFNG*	3

ID	Description	GeneRatio	BgRatio	pvalue	p.adjust	qvalue	geneID	Count
	protein catabolic process							
GO:0072524	pyridine-containing compound metabolic process	9/293	195/18670	0,003762	0,009655	0,004254	*TP53/STAT3/ARNT/HIF1A/INSR/INS/IFNG/SLC4A1/IGF1*	9
GO:1900117	regulation of execution phase of apoptosis	4/293	41/18670	0,003812	0,009772	0,004306	*TP53/IL6/BAX/BCL2L1*	4
GO:1905209	positive regulation of cardiocyte differentiation	4/293	41/18670	0,003812	0,009772	0,004306	*MTOR/TGFB1/HAMP/IGF1*	4
GO:0045069	regulation of viral genome replication	6/293	95/18670	0,00384	0,009829	0,004331	*TNF/BCL2/LTF/CXCL8/EIF2AK2/FMR1*	6
GO:0055006	cardiac cell development	6/293	95/18670	0,00384	0,009829	0,004331	*MTOR/AGT/VEGFA/NEB/HAMP/IGF1*	6
GO:2000060	positive regulation of ubiquitin-dependent protein catabolic process	6/293	95/18670	0,00384	0,009829	0,004331	*AKT1/MDM2/HSPA1B/HSPA1A/CEBPA/EGF*	6
GO:1990778	protein localization to cell periphery	12/293	311/18670	0,003866	0,009891	0,004358	*AKT1/EGFR/AR/TNFRSF1A/LGALS3/TNF/FLNA/BCL2L1/ TGFB1/INS/IFNG/SLC4A1*	12
GO:0050804	modulation of chemical synaptic transmission	15/293	436/18670	0,003886	0,009935	0,004378	*SRC/STAT3/EGFR/MTOR/JAK2/ADRB2/IL1B/TNF/AGT/AP OE/PLAT/CREB1/CRTC1/INS/FMR1*	15
