# Supplementary material for: From bench to in silico and backwards: What have we done on genetics of recurrent pregnancy loss and implantation failure and where should we go next?
Source: Genet Mol Biol. 2024 Aug 26;46(3 Suppl 1):e20230127. doi: 10.1590/1678-4685-GMB-2023-0127 (PMC11346592; doi:10.1590/1678-4685-GMB-2023-0127)
Supplement: Table S5 - [file 1415-4757-GMB-46-03-s1-e20230127-s5.pdf]

Genetics and Molecular Biology

DOI: https://doi.org/10.1590/1678-4685-GMB-2023-0127

Supplementary Material to "From bench to *in silico* and backwards: what have we done on genetics of recurrent pregnancy loss and implantation failure and where should we go next?"

Table S5 - Enriched KEGG pathways for recurrent pregnancy loss + implantation failure network.

ID	Description	GeneRatio	BgRatio	pvalue	p.adjust	qvalue	geneID	Count
hsa05200	Pathways in cancer	54/214	327/5894	1,85E-22	2,68E-20	1,56E-20	NFKB1/TP53/PLD1/AKT1/STAT3/STAT5B/TRAF1/VHL/EGFR/MDM2/CASP3/CHUK/MTOR/AR/FGFR1/BAD/IKBKG/IGF1R/PPARG/FN1/GSTP1/TGFBR1/BMP2/IL6/ARNT/ARNT2/HIF1A/CASP9/MMP2/BAX/BCL2/BCL2L1/APC/ITGA2B/MMP9/BRCA2/ITGA2/MMP1/CEBPA/VEGFA/RXRB/TGFB1/CXCL8/EGF/FASLG/FGF1/FGFR3/FGF7/VEGFC/VEGFB/PGF/IGF1/LAMA4/NOS2	54
hsa05332	Graft-versus-host disease	21/214	43/5894	1,13E-19	8,18E-18	4,75E-18	IL1B/TNF/IL6/HLA-E/HLA-C/HLA-G/HLA-A/HLA-B/KLRC1/KIR2DL3/FASLG/IFNG/KIR3DL1/HLA-DQA1/HLA-DRB5/HLA-DRA/HLA-DRB1/HLA-DQB1/HLA-DQA2/IL1A/KIR2DL1	21
hsa05145	Toxoplasmosis	32/214	133/5894	2,11E-18	1,02E-16	5,93E-17	HSPA1L/NFKB1/AKT1/STAT3/TLR4/CASP3/CHUK/BAD/IKBKG/TNFRSF1A/HSPA1B/HSPA1A/JAK2/CCR5/TNF/IL10RA/CASP9/BCL2/BCL2L1/TGFB1/IL12B/IFNG/HLA-DQA1/HLA-DRB5/HLA-DRA/HLA-DRB1/HLA-DQB1/HLA-DQA2/IL12A/LAMA4/NOS2/IL10	32
hsa04940	Type I diabetes mellitus	20/214	45/5894	9,33E-18	3,38E-16	1,96E-16	IL1B/TNF/HLA-E/HLA-C/HLA-G/HLA-A/HLA-B/IL12B/INS/FASLG/IFNG/HLA-DQA1/HLA-DRB5/HLA-DRA/HLA-	20

ID	Description	GeneRatio	BgRatio	pvalue	p.adjust	qvalue	geneID	Count
							DRB1/HLA-DQB1/HLA-DQA2/IL12A/IL1A/LTA	
hsa05330	Allograft rejection	18/214	39/5894	1,88E-16	5,45E-15	3,16E-15	TNF/HLA-E/HLA-C/HLA-G/HLA-A/HLA-B/IL12B/FASLG/IFNG/HLA-DQA1/HLA-DRB5/HLA-DRA/HLA-DRB1/HLA-DQB1/HLA-DQA2/IL12A/IL4/IL10	18
hsa04612	Antigen processing and presentation	23/214	78/5894	1,40E-15	3,38E-14	1,96E-14	HSPA1L/HSPA1B/HSPA1A/TNF/CD8A/HLA-E/HLA-C/HLA-G/HLA-A/HLA-B/KLRC1/TAP2/KIR2DL3/CREB1/IFNG/KIR3DL1/HLA-DQA1/HLA-DRB5/HLA-DRA/HLA-DRB1/HLA-DQB1/HLA-DQA2/KIR2DL1	23
hsa04610	Complement and coagulation cascades	20/214	69/5894	1,58E-13	3,27E-12	1,90E-12	FGG/F2/PROC/MBL2/SERPINC1/CPB2/F12/FGB/F7/FGA/SERPINA1/PLAT/SERPINE1/C5AR1/F13A1/THBD/F10/TFPI/F5/F3	20
hsa05212	Pancreatic cancer	19/214	70/5894	2,44E-12	4,42E-11	2,57E-11	NFKB1/TP53/PLD1/AKT1/STAT3/EGFR/CHUK/BAD/IKBKG/TGFBR1/CASP9/BCL2L1/BRCA2/VEGFA/TGFB1/EGF/VEGFC/VEGFB/PGF	19
hsa05140	Leishmaniasis	19/214	73/5894	5,55E-12	8,94E-11	5,19E-11	NFKB1/TLR4/JAK2/IL1B/TNF/TGFB1/IL12B/IFNG/HLA-DQA1/HLA-DRB5/HLA-DRA/HLA-DRB1/HLA-DQB1/HLA-DQA2/IL12A/IL1A/IL4/NOS2/IL10	19
hsa05323	Rheumatoid arthritis	21/214	92/5894	6,40E-12	9,27E-11	5,39E-11	TLR4/IL1B/TNF/IL6/MMP3/MMP1/VEGFA/TGFB1/CTLA4/CXCL8/FLT1/VEGFB/PGF/IFNG/HLA-DQA1/HLA-DRB5/HLA-DRA/HLA-DRB1/HLA-DQB1/HLA-DQA2/IL1A	21
hsa04060	Cytokine-cytokine receptor interaction	35/214	265/5894	1,21E-11	1,60E-10	9,28E-11	EGFR/TNFRSF1A/ACVR1/TGFBR1/BMP2/IL1B/CCR5/TNF/IL6/IL1R1/IL10RA/LEPR/PRLR/VEGFA/PRL/TGFB1/IL12B/CXCL8/CXCR1/EGF/FASLG/KDR/VEGFC/FLT1/VEGFB/IL23R/IFNG/IL12A/IL1A/IL4R/IL4/LEP/LTA/IL10/IL6R	35
hsa05320	Autoimmune thyroid disease	16/214	54/5894	3,25E-11	3,92E-10	2,28E-10	HLA-E/HLA-C/HLA-G/HLA-A/HLA-B/CGA/CTLA4/FASLG/HLA-DQA1/HLA-DRB5/HLA-DRA/HLA-DRB1/HLA-DQB1/HLA-DQA2/IL4/IL10	16

ID	Description	GeneRatio	BgRatio	pvalue	p.adjust	qvalue	geneID	Count
hsa05146	Amoebiasis	21/214	106/5894	1,14E-10	1,27E-09	7,36E-10	NFKB1/TLR4/CASP3/FN1/IL1B/TNF/IL6/IL1R1/CD14/TGFB1/COL1A1/COL5A1/COL5A2/COL1A2/IL12B/CXCL8/IFNG/IL12A/LAMA4/NOS2/IL10	21
hsa05215	Prostate cancer	19/214	89/5894	2,36E-10	2,44E-09	1,42E-09	NFKB1/TP53/AKT1/EGFR/MDM2/CHUK/MTOR/AR/FGFR1/BAD/IKBKG/IGF1R/GSTP1/CASP9/BCL2/CREB1/EGF/INS/IGF1	19
hsa04510	Focal adhesion	28/214	200/5894	4,38E-10	4,24E-09	2,46E-09	SRC/AKT1/EGFR/BAD/IGF1R/FN1/FLNA/BCL2/ITGB4/ITGB6/ITGA2B/ITGB3/ITGA2/VEGFA/COL1A1/COL5A1/COL6A3/COL5A2/COL6A1/COL1A2/EGF/KDR/VEGFC/FLT1/VEGFB/PGF/IGF1/LAMA4	28
hsa05142	Chagas disease (American trypanosomiasis)	20/214	104/5894	5,66E-10	5,13E-09	2,98E-09	ACE/NFKB1/AKT1/TLR4/CHUK/IKBKG/TNFRSF1A/TGFBR1/IL1B/TNF/IL6/SERPINE1/TGFB1/IL12B/CXCL8/FASLG/IFNG/IL12A/NOS2/IL10	20
hsa05219	Bladder cancer	13/214	42/5894	1,33E-09	1,14E-08	6,61E-09	TP53/EGFR/MDM2/MMP2/MMP9/MMP1/VEGFA/CXCL8/EGF/FGFR3/VEGFC/VEGFB/PGF	13
hsa04640	Hematopoietic cell lineage	18/214	88/5894	1,50E-09	1,21E-08	7,04E-09	IL1B/TNF/IL6/IL1R1/ITGA2B/ITGB3/CD14/CD8A/CD9/ITGA2/TFRC/HLA-DRB5/HLA-DRA/HLA-DRB1/IL1A/IL4R/IL4/IL6R	18
hsa04210	Apoptosis	17/214	89/5894	1,31E-08	1,00E-07	5,82E-08	NFKB1/TP53/AKT1/CASP3/CHUK/BAD/IKBKG/TNFRSF1A/IL1B/TNF/IL1R1/CASP9/BAX/BCL2/BCL2L1/FASLG/IL1A	17
hsa05143	African trypanosomiasis	10/214	35/5894	2,67E-07	1,94E-06	1,13E-06	IL1B/TNF/IL6/IL12B/FASLG/IFNG/HBA1/IL12A/LAMA4/IL10	10
hsa05222	Small cell lung cancer	15/214	85/5894	2,91E-07	2,01E-06	1,17E-06	NFKB1/TP53/AKT1/TRAF1/CHUK/IKBKG/FN1/CASP9/BCL2/BCL2L1/ITGA2B/ITGA2/RXRB/LAMA4/NOS2	15
hsa04630	Jak-STAT signaling pathway	20/214	155/5894	6,27E-07	4,13E-06	2,40E-06	PIM1/AKT1/STAT3/STAT5B/JAK2/IL6/IL10RA/LEPR/PRLR/BCL2L1/PRL/IL12B/IL23R/IFNG/IL12A/IL4R/IL4/LEP/IL10/IL6R	20
hsa04144	Endocytosis	23/214	203/5894	9,24E-07	5,63E-06	3,27E-06	HSPA1L/PLD1/SRC/EGFR/MDM2/IGF1R/HSPA1B/HSPA1A/TGFBR1/ADRB2/CCR5/HLA-E/HLA-C/HLA-G/HLA-A/HLA-B/TGFB1/CXCR1/EGF/FGFR3/KDR/FLT1/TFRC	23

ID	Description	GeneRatio	BgRatio	pvalue	p.adjust	qvalue	geneID	Count
hsa05310	Asthma	9/214	31/5894	9,32E-07	5,63E-06	3,27E-06	TNF/HLA-DQA1/HLA-DRB5/HLA-DRA/HLA-DRB1/HLA-DQB1/HLA-DQA2/IL4/IL10	9
hsa05416	Viral myocarditis	13/214	72/5894	1,41E-06	8,18E-06	4,75E-06	CASP3/CASP9/HLA-E/HLA-C/HLA-G/HLA-A/HLA-B/HLA-DQA1/HLA-DRB5/HLA-DRA/HLA-DRB1/HLA-DQB1/HLA-DQA2	13
hsa04145	Phagosome	19/214	156/5894	2,94E-06	1,64E-05	9,52E-06	TLR4/MBL2/ITGB3/CD14/HLA-E/HLA-C/HLA-G/HLA-A/HLA-B/TAP2/ITGA2/TUBA1A/TFRC/HLA-DQA1/HLA-DRB5/HLA-DRA/HLA-DRB1/HLA-DQB1/HLA-DQA2	19
hsa04920	Adipocytokine signaling pathway	12/214	68/5894	4,66E-06	2,50E-05	1,45E-05	NFKB1/AKT1/STAT3/CHUK/MTOR/IKBKG/TNFRSF1A/JAK2/TNF/LEPR/RXRB/LEP	12
hsa04672	Intestinal immune network for IgA production	10/214	49/5894	7,66E-06	3,97E-05	2,30E-05	IL6/TGFB1/HLA-DQA1/HLA-DRB5/HLA-DRA/HLA-DRB1/HLA-DQB1/HLA-DQA2/IL4/IL10	10
hsa04512	ECM-receptor interaction	13/214	85/5894	9,67E-06	4,84E-05	2,81E-05	FN1/ITGB4/ITGB6/ITGA2B/ITGB3/ITGA2/COL1A1/COL5A1/COL6A3/COL5A2/COL6A1/COL1A2/LAMA4	13
hsa04010	MAPK signaling pathway	25/214	268/5894	1,09E-05	5,23E-05	3,04E-05	HSPA1L/NFKB1/TP53/AKT1/EGFR/CASP3/CHUK/FGFR1/IKBKG/TNFRSF1A/HSPA1B/HSPA1A/TGFBR1/IL1B/TNF/IL1R1/FLNA/CD14/TGFB1/EGF/FASLG/FGF1/FGFR3/FGF7/IL1A	25
hsa05144	Malaria	10/214	51/5894	1,12E-05	5,23E-05	3,04E-05	TLR4/IL1B/TNF/IL6/TGFB1/CXCL8/IFNG/HBA1/IL12A/IL10	10
hsa04380	Osteoclast differentiation	16/214	128/5894	1,32E-05	5,97E-05	3,47E-05	NFKB1/AKT1/CHUK/IKBKG/TNFRSF1A/PPARG/TGFBR1/IL1B/TNF/IL1R1/ITGB3/LILRB1/CREB1/TGFB1/IFNG/IL1A	16
hsa05150	Staphylococcus aureus infection	10/214	56/5894	2,66E-05	0,000117	6,78E-05	FGG/MBL2/C5AR1/HLA-DQA1/HLA-DRB5/HLA-DRA/HLA-DRB1/HLA-DQB1/HLA-DQA2/IL10	10
hsa04115	p53 signaling pathway	11/214	69/5894	3,19E-05	0,000136	7,91E-05	TP53/MDM2/CASP3/IGFBP3/TP73/CASP9/BAX/RRM2/SERPINE1/CD82/IGF1	11
hsa05221	Acute myeloid leukemia	10/214	58/5894	3,65E-05	0,000151	8,79E-05	PIM1/NFKB1/AKT1/STAT3/STAT5B/CHUK/MTOR/BAD/IKBKG/CEBPA	10
hsa05218	Melanoma	11/214	71/5894	4,21E-05	0,000169	9,84E-05	TP53/AKT1/EGFR/MDM2/FGFR1/BAD/IGF1R/EGF/FGF1/FGF7/IGF1	11

ID	Description	GeneRatio	BgRatio	pvalue	p.adjust	qvalue	geneID	Count
hsa05220	Chronic myeloid leukemia	11/214	73/5894	5,48E-05	0,000215	0,000125	NFKB1/TP53/AKT1/STAT5B/MDM2/CHUK/BAD/IKBKG/TGFBR1/BCL2L1/TGFB1	11
hsa05210	Colorectal cancer	10/214	62/5894	6,63E-05	0,000253	0,000147	TP53/AKT1/CASP3/BAD/TGFBR1/CASP9/BAX/BCL2/APC/TGFB1	10
hsa04620	Toll-like receptor signaling pathway	13/214	102/5894	7,09E-05	0,000264	0,000153	NFKB1/AKT1/TLR4/CHUK/IKBKG/IL1B/TNF/IL6/CD14/IL12B/CXCL8/IL12A/TLR1	13
hsa04150	mTOR signaling pathway	9/214	52/5894	8,75E-05	0,000317	0,000184	AKT1/MTOR/HIF1A/VEGFA/INS/VEGFC/VEGFB/PGF/IGF1	9
hsa04514	Cell adhesion molecules (CAMs)	15/214	136/5894	0,000108	0,000382	0,000222	NCAM1/L1CAM/CD8A/HLA-E/HLA-C/HLA-G/HLA-A/HLA-B/CTLA4/HLA-DQA1/HLA-DRB5/HLA-DRA/HLA-DRB1/HLA-DQB1/HLA-DQA2	15
hsa05014	Amyotrophic lateral sclerosis (ALS)	9/214	54/5894	0,000119	0,00041	0,000238	TP53/CASP3/BAD/TNFRSF1A/TNF/CASP9/BAX/BCL2/BCL2L1	9
hsa05410	Hypertrophic cardiomyopathy (HCM)	11/214	83/5894	0,000181	0,00061	0,000354	ACE/TNF/IL6/ITGB4/ITGB6/ITGA2B/ITGB3/ITGA2/TGFB1/DES/IGF1	11
hsa05211	Renal cell carcinoma	10/214	70/5894	0,00019	0,000625	0,000363	AKT1/VHL/ARNT/ARNT2/HIF1A/VEGFA/TGFB1/VEGFC/VEGFB/PGF	10
hsa04614	Renin-angiotensin system	5/214	17/5894	0,000261	0,00084	0,000488	ACE/ACE2/AGTR1/AGT/REN	5
hsa05160	Hepatitis C	13/214	135/5894	0,001152	0,00363	0,002108	NFKB1/TP53/AKT1/STAT3/EGFR/CHUK/BAD/IKBKG/TNFRSF1A/TNF/CXCL8/EIF2AK2/EGF	13
hsa04650	Natural killer cell mediated cytotoxicity	13/214	136/5894	0,001233	0,003804	0,002209	CASP3/TNF/HLA-E/HLA-C/HLA-G/HLA-A/HLA-B/KLRC1/KIR2DL3/FASLG/IFNG/KIR3DL1/KIR2DL1	13
hsa05020	Prion diseases	6/214	36/5894	0,001669	0,005043	0,002929	NCAM1/HSPA1A/IL1B/IL6/BAX/IL1A	6
hsa05214	Glioma	8/214	65/5894	0,002249	0,006654	0,003864	TP53/AKT1/EGFR/MDM2/MTOR/IGF1R/EGF/IGF1	8
hsa05213	Endometrial cancer	7/214	52/5894	0,00251	0,007279	0,004227	TP53/AKT1/EGFR/BAD/CASP9/APC/EGF	7
hsa05120	Epithelial cell signaling in Helicobacter pylori infection	8/214	68/5894	0,003001	0,008531	0,004954	NFKB1/SRC/EGFR/CASP3/CHUK/IKBKG/CXCL8/CXCR1	8
hsa05223	Non-small cell lung cancer	7/214	54/5894	0,003125	0,008714	0,005061	TP53/AKT1/EGFR/BAD/CASP9/RXRB/EGF	7

ID	Description	GeneRatio	BgRatio	pvalue	p.adjust	qvalue	geneID	Count
hsa04810	Regulation of actin cytoskeleton	16/214	214/5894	0,004598	0,012578	0,007305	EGFR/FGFR1/FN1/F2/APC/ITGB4/ITGB6/ITGA2B/ITGB3/CD14/ITGA2/EGF/INS/FGF1/FGFR3/FGF7	16
hsa05414	Dilated cardiomyopathy	9/214	90/5894	0,005098	0,013621	0,007911	TNF/ITGB4/ITGB6/ITGA2B/ITGB3/ITGA2/TGFB1/DES/IGF1	9
hsa04621	NOD-like receptor signaling pathway	7/214	59/5894	0,005167	0,013621	0,007911	NFKB1/CHUK/IKBKG/IL1B/TNF/IL6/CXCL8	7
hsa04660	T cell receptor signaling pathway	10/214	108/5894	0,005583	0,014456	0,008396	NFKB1/AKT1/CHUK/IKBKG/TNF/CD8A/CTLA4/IFNG/IL4/IL10	10
hsa04974	Protein digestion and absorption	8/214	81/5894	0,008738	0,022228	0,012909	ACE2/CPB2/COL1A1/COL5A1/COL6A3/COL5A2/COL6A1/COL1A2	8
hsa04622	RIG-I-like receptor signaling pathway	7/214	71/5894	0,013981	0,034951	0,020298	NFKB1/CHUK/IKBKG/TNF/IL12B/CXCL8/IL12A	7
hsa04370	VEGF signaling pathway	7/214	76/5894	0,019755	0,04855	0,028196	SRC/AKT1/NOS3/BAD/CASP9/VEGFA/KDR	7
hsa00100	Steroid biosynthesis	3/214	19/5894	0,029769	0,071942	0,041781	NSDHL/DHCR7/EBP	3
hsa04350	TGF-beta signaling pathway	7/214	85/5894	0,034007	0,080837	0,046947	ACVR1/TGFBR1/BMP2/TNF/TGFB1/FST/IFNG	7
hsa05340	Primary immunodeficiency	4/214	35/5894	0,036689	0,085806	0,049833	IKBKG/ADA/CD8A/TAP2	4
hsa04080	Neuroactive ligand-receptor interaction	16/214	272/5894	0,037768	0,085899	0,049887	THRB/NR3C1/ADRA2A/ADRB2/AGTR1/F2/LEPR/PRLR/HTR1A/C5AR1/CGA/LHB/PRL/FSHB/FSHR/LEP	16
hsa04012	ErbB signaling pathway	7/214	87/5894	0,037914	0,085899	0,049887	SRC/AKT1/STAT5B/EGFR/MTOR/BAD/EGF	7
